# Comparative pathogenomic analysis reveals a highly tetanus toxin-producing clade of *Clostridium tetani* isolates in Japan

**DOI:** 10.1128/msphere.00369-23

**Published:** 2023-11-27

**Authors:** Chie Shitada, Tsuyoshi Sekizuka, Akihiko Yamamoto, Chiyomi Sakamoto, Masanori Hashino, Makoto Kuroda, Motohide Takahashi

**Affiliations:** 1 Toxin and Biologicals Research Laboratory, Kumamoto Health Science University, Kumamoto, Japan; 2 The Chemo-Sero-Therapeutic Research Institute, Kumamoto, Japan; 3 Pathogen Genomics Center, National Institute of Infectious Diseases, Tokyo, Japan; 4 Department of Bacteriology II, National Institute of Infectious Diseases, Tokyo, Japan; University of Maryland Medical Center, Baltimore, Maryland, USA

**Keywords:** tetanus toxin, *Clostridium tetani*, ELISA, mice experiment, genome analysis, transcriptome

## Abstract

*Clostridium tetani* is the etiological agent of the disease tetanus, resulting in various symptoms, including neck tension, tongue-twisting, a decline in walking ability, and death in severe cases. To date, publicly available genome sequences of *C. tetani* are limited. *C. tetani* was isolated from soil in Kumamoto Prefecture, Japan, between 2020 and 2022. Subsequently, bacteriological, biochemical, genetic, and immunological tests were conducted. Among the 46 soil sampling sites, 151 *C*. *tetani* strains were isolated from 33 sites, with a 71.7% positivity rate. Comparative genomic analysis subdivided clade 1 into clades 1-1, 1-2, and 1-3. The enzyme-linked immunosorbent assay revealed that partial clade 1-3 strains produced over sevenfold more tetanus neurotoxin (TeNT) than the reference strain (first isolate of *C. tetani* KHSU-154301-001). RNA-Seq analysis supported this elevated tetanus toxin *tetX* gene expression. Additionally, *tetX*-negative strains were isolated from the clade, indicating that TeNT may not be essential for ensuring the survival of their offspring without its representative toxin-causing tetanus. Five strains acquired the tetracycline resistance gene *tet*(M) in the *tetX* plasmid, resulting in significantly reduced susceptibility to tetracycline. In conclusion, although we investigated *C. tetani* isolates from a specific region of the Kumamoto Prefecture, we demonstrated that these isolates exhibit variable genomic features, toxin productivity, and substantial acquired antimicrobial resistance. These findings indicate that multiple *C. tetani* lineages have successfully adapted to the soil in Japan, as evidenced by the additional genome information and toxin productivity assays in this study.

**IMPORTANCE:**

*C. tetani* is a spore-forming, anaerobic bacterium that produces a toxin causing muscle stiffness and paralysis. Tetanus is preventable with the toxoid vaccine, but it remains a significant public health threat in regions with low vaccine coverage. However, there are relatively few isolates and limited genomic information available worldwide. In Japan, about 100 cases are reported each year, but there have been no nationwide surveys of isolates, and no genomic information from Japanese isolates has been published. In our study, we analyzed the genomes of 151 strains from a limited survey of soil in Kumamoto, Japan. Our findings revealed a high degree of genetic diversity, and we also identified a subset of strains that produced significantly more toxin, which provides new insights into the pathogenesis of tetanus. Our findings lay the foundation for future studies to investigate the distribution and evolution of *C. tetani* in Japan and neighboring countries.

## INTRODUCTION

Tetanus, a condition characterized by tonic spasm paralysis, stems from the toxin generated by *Clostridium tetani*, an ancient wound infection agent. *C. tetani* is a Gram-positive, anaerobic, spore-forming bacterium that is widely distributed in the soil. It is resistant to heat, desiccation, and alcohol-based disinfectants. In cases of wound infection in the body, an anaerobic environment is created, causing toxin production. Tetanus neurotoxin (TeNT) comprises two polypeptide chains: a light and heavy chain, with molecular weights of approximately 50 and 100 kDa. The mechanism of action of the toxin can be described as follows: initially, the heavy chain binds to gangliosides present on the nerve cell membrane, and subsequently, the toxin molecule enters the cell. After entry, the light chain, possessing zinc-dependent proteolytic capabilities, degrades vesicle associated membrane proteins (VAMPs), one of the SNARE proteins responsible for neurotransmitter exocytosis, thereby inhibiting neurotransmitter release. This results in spasmodic motor muscle paralysis (1). Disease onset typically occurs within 3–20 days (average: 7 days) after infection, with a reported incubation period of up to 120 days (2). Following symptoms, including neck tension, tongue-twisting, and injured limb prodding, the patient progressively loses the ability to walk, causing general paralysis. In severe cases, respiratory muscle paralysis can lead to death (2–4).

In Japan, the vaccination schedule involves four doses administered between 2 months and 2 years of age to establish basic immunity, followed by supplementary immunization at 11–12 years old. No neonatal tetanus cases have been reported since 2008. However, sporadic deaths occurred among adults between 2000 and 2020, averaging approximately 100 cases annually, with a few incidents reported in Kumamoto Prefecture. Tetanus risk stems from bacteria entering wounds caused by outdoor injuries. High-risk wounds include those contaminated with soil or feces, punctures, burns, and contusions. Additionally, the infection risk is notably high for individuals >50 years old. This is because the neutralizing antibodies can persist until age 40 years when the antibody retention rate is approximately 80% (5). An anti-tetanus antibody retention rate of ≥0.1 IU/mL is considered minimum for protection against the disease onset. Nevertheless, the rate decreases gradually in adults in their 50s and markedly drops to <30% for those over 55 years of age. Therefore, in Japan, despite the widespread vaccine availability, elderly individuals without vaccination or with lowered antibody levels may still be susceptible to tetanus infections.

To date, only a few nationwide environmental surveys for *C. tetani* in soil and other materials have been conducted (6, 7). *C. tetani* spores are frequently isolated from soil samples in natural environments (8, 9) and are in various animal intestinal tracts (4). *C. tetani* ecological role in soil remains poorly characterized. However, its strong swarming potential (10), lack of glucose fermentation, and asaccharolytic abilities may indicate traits common among environmental bacteria rather than host-associated *Clostridium* species, including *Clostridium botulinum* and *Clostridium perfringens*.

TeNT is similar to botulinum neurotoxin (BoNT) structurally and induces flaccid nerve paralysis. BoNTs are classified into seven serotypes (BoNT/A-G), with TeNTs exhibiting the highest structural similarity to BoNT/B (11). Only one TeNT has been characterized to date, and *C. tetani* displays significantly lower genetic diversity than *C. botulinum* strains (11). The reason TeNTs have not evolved into serotypes resembling BoNTs remains unclear. Comparative genome analysis is a direct method to reveal the possible pathways for TeNT acquisition and maintenance within a single serotype.

The initial complete genome sequence of TeNT-producing *C. tetani* strain E88 was published in 2003 (12). In 2021, only 43 *C*. *tetani* whole-genome sequences were available, and none originated from Japan at that time. Comparative and pan-genome analyses of *C. tetani* isolates have been conducted (13, 14). However, limited data are available regarding *C. tetani* isolate genomic diversity within specific countries or global regions (11, 12, 15, 16). To date, *C. tetani* isolates have been categorized into two clades, clades 1 and 2 (14), with clade 1 strains being more prevalent globally. While Japanese isolates have not undergone the sequencing mentioned above, metagenomic sequencing of ancient forensic DNA recently revealed the prevalence of the clade 2 genome sequence in tooth samples from the Jomon-era Sanganji Shell Mound in Japan (~1044 BCE) (17). This indicates the presence of the clade 2 strain in Japan during the BCE period.

Therefore, this study aims to characterize the features of *C. tetani* Japanese isolates. Initially, we examined the occurrence and prevalence of *C. tetani* in the soil of Kumamoto Prefecture. Subsequently, we conducted a whole-genome analysis of newly obtained Kumamoto Prefecture isolates and compared them with publicly available genome sequences. Additional experiments using enzyme-linked immunosorbent assay (ELISA) and immunological (neutralization) studies in mice clearly highlighted marked TeNT-producing *C. tetani* isolates associated with a distinct genomic clade.

## RESULTS AND DISCUSSION

### Presence/prevalence of *C. tetani* in Kumamoto Prefecture

**Fig 1:**
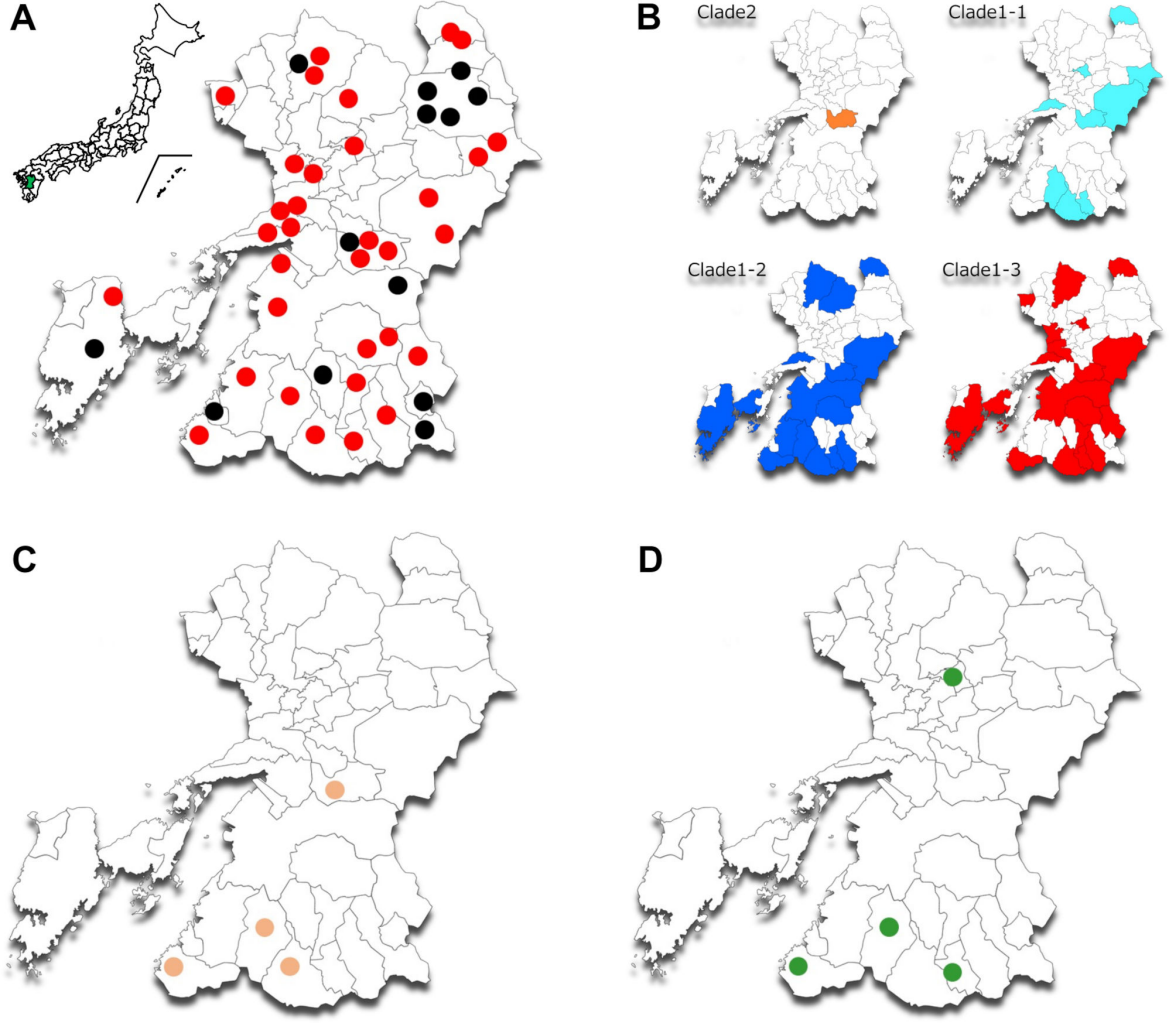
*Clostridium tetani* distribution in Kumamoto Prefecture. (**A**) Closed circles depict soil sampling sites in Kumamoto Prefecture. Red circles denote 151 *C*. *tetani* strains isolated from 33 of the 46 collection sites. The positivity and isolation rates were 71.7% and 57.1%, respectively. Tetanus was widely distributed in Kumamoto Prefecture, with no isolates obtained from the 13 locations denoted by black circles. (**B**) The prevalence of genomic clade features (**C**), tetanus toxin gene *tetX* negative (**D**), and tetracycline resistance gene *tet*(M) positive in *C. tetani* strains (Fig. 3).

**Fig 2:**
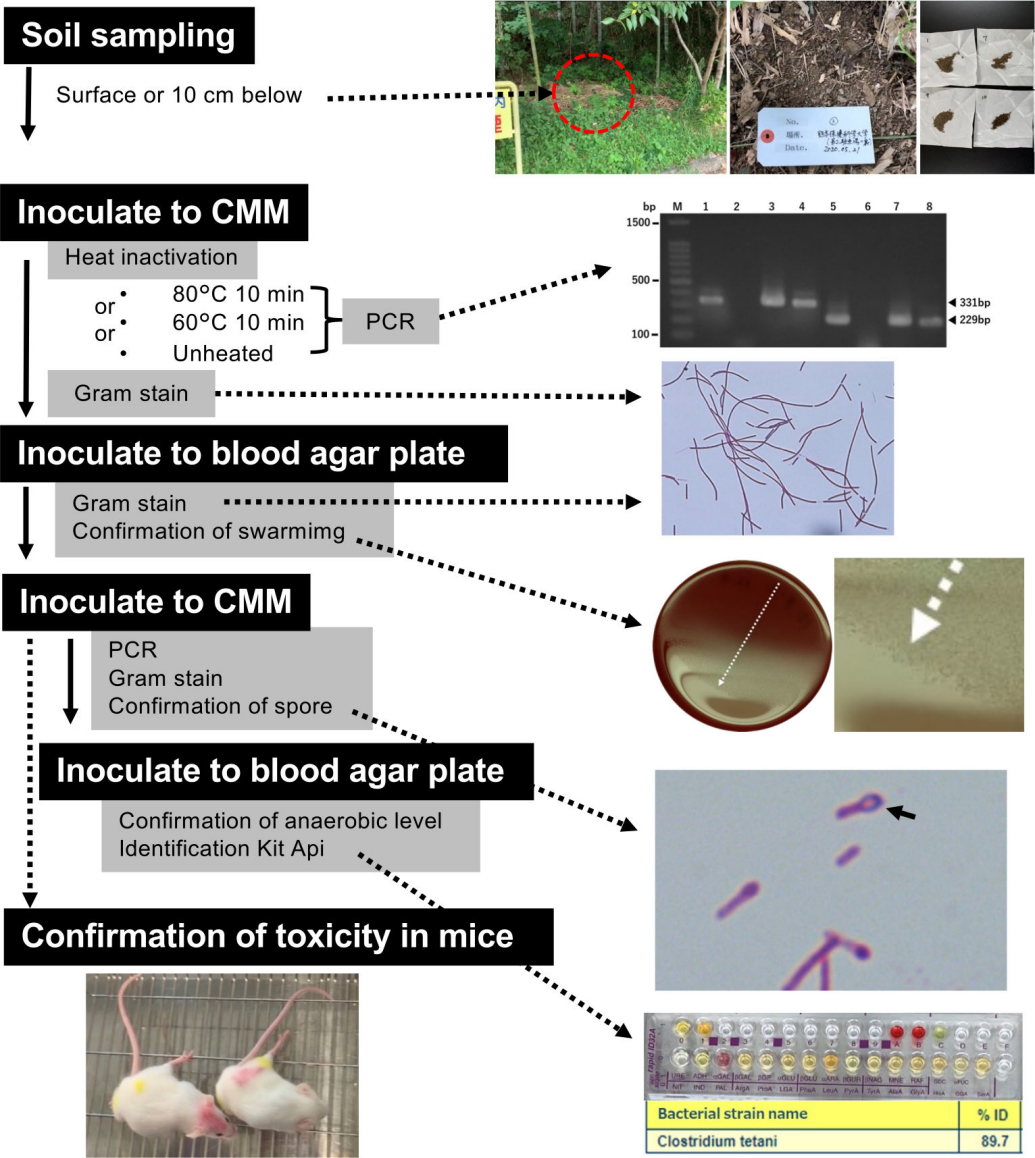
Isolation and identification process of *C. tetani*. The soil samples were initially added to cooked meat medium (CMM), a widely used culture medium for *Clostridium* spp., and subjected to incubation at 37°C for 2–3 days. This was performed after heated and unheated treatments to facilitate subsequent bacteriological, biochemical, genetic, and immunological testing. To isolate and identify *C. tetani*, a CM culture solution was inoculated dropwise onto the blood agar medium, followed by anaerobic incubation at 37°C for 18–24 h. *C. tetani* exhibited swarming behavior on the medium (arrow). Gram staining of the swarming tips on the medium revealed filamentous long rods. The swarm tip was introduced into fresh CMM; *C. tetani* was cultured in pure culture; and the spores were confirmed using Gram staining. The presence of the tetanus toxin gene was verified through PCR. M, DNA ladder; 1 and 5 indicate positive control; 2 and 6 indicate negative control; 3, 4, 7, and 8 denote isolates. If the tetanus toxin gene was present, PCR products were detected at 331 bp for GAT1/GAT2 and 229 bp for GAT5/GAT6 using the two sets of primers. For biochemical characterization, a pure culture of *C. tetani* from CMM was anaerobically incubated at 37°C for 18–24 h on a blood agar medium. The bacillus solution was then prepared and analyzed using API kit. The presence of tetanus toxin in the mice was confirmed by assessing its biological activity. The tetanus culture supernatant solution was subsequently injected into the inner left thigh of two mice, and their condition was observed for 4 days. The mice exhibited symptoms of tetanus, including characteristic left hind limb protrusion, tonic paralysis, limping, and gradual decline in walking ability.

The typical traits of *C. tetani* were assessed, including positive swarming, spore formation, and a filamentous long-rod morphology. Furthermore, polymerase chain reaction (PCR) tests and analytical profile index (API) biochemical identification were performed to identify *C. tetani* isolates. *C. tetani* was positively isolated from soil samples at 33 locations across 46 sampling sites (Fig. 1A; Table 1), with a 71.7% positivity rate. *C. tetani* was not found at the four sites in the northeastern part of the prefecture. This absence may be attributed to the soil samples having unfavorable conditions owing to their acidic volcanic content, which could inhibit the growth of *C. tetani,* which prefers an alkaline environment. Of the 264 samples collected, considering variations in soil depth and heat pretreatment, we identified 151 strains, resulting in an isolation rate of 57.1% (Fig. 2).

**TABLE 1:** The number of isolates undersampling and heating conditions per location

Place	Separation location/collection location	Isolation /sample	Sampling condition		Heating condition		
			Surface	10 cm below	80°C	60°C	Unheated
1	1/1	1/6	1	–^*a*^	–	1	–
2	2/3	4/18	3	1	1	1	2
3	1/1	1/6	–	1	1	–	–
4	3/3	5/18	4	1	1	1	3
5	3/3	13/18	6	7	3	6	4
6	2/3	4/18	3	1	2	–	2
7	1/2	2/12	2	–	–	1	1
8	1/1	13/6	1	12	4	8	1
9	1/1	14/6	9	5	7	3	4
10	1/1	3/6	–	3	1	1	1
11	1/1	6/6	3	3	2	2	2
12	1/1	3/6	2	1	2	–	1
13	2/2	5/12	2	3	1	2	2
14	2/2	10/12	5	5	4	4	2
15	3/4	25/24	18	7	4	3	18
16	1/1	15/6	13	2	8	7	–
17	1/1	6/6	1	5	3	–	3
18	1/1	1/6	–	1	–	–	1
19	1/1	15/6	11	4	10	4	1
20	1/1	1/6	1	–	–	1	–
21	2/2	3/12	1	2	1	–	2
22	1/1	1/6	–	1	–	1	–
23	0/5	0/30	–	–	–	–	–
24	0/1	0/6	–	–	–	–	–
25	0/1	0/6	–	–	–	–	–
26	0/1	0/6	–	–	–	–	–
27	0/1	0/6	–	–	–	–	–

^*a*^ "ー" is "not detected".

Under different sampling conditions, 86 and 65 isolates were identified at the ground surface and 10 cm below the surface, respectively. Regarding heat pretreatment selection, 55, 46, and 50 isolates were identified at 80°C and 60°C and no heating, respectively (Table 1). *C. tetani* isolates from the southern areas of Kumamoto Prefecture showed significant swarming potential; therefore, the agar concentration was increased to manage swarming during isolation. *C. tetani* was successfully isolated from various soil samples across Kumamoto Prefecture, with minimal variations based on the collection and heating conditions.

Based on the results of the Rapid 32A API Identification kit for *C. tetani*, using standard biochemical tests, out of the 29 test indices, a single positive marker of PAL (alkaline phosphatase) indicated an identification reliability of 89.7%. However, the reliability increased to 95.0% when two markers, PAL and ADH (arginine dihydrolase), were positive. Indole production was not observed based on the typical biochemical properties of *C. tetani*, such as motility (+), glucose degradation (−), and nitrate reduction (−). Several candidates of *C. tetani* isolates were found to be other *Clostridium* species, including *Clostridium glycoiycum*, *Clostridium septicum*, *Clostridium sprogenes*, and *C. perfringens*.

We speculate that conducting multiple cultivation trials from a single soil sample may enhance the successful isolation of *C. tetani*, especially when dealing with variable pretreatments (Fig. 2). This hypothesis arises from the absence of clear indications of optimal pretreatment conditions at the collection points (sites, surface, or underground) or heating conditions in this study. Additionally, we could not isolate *C. tetani* from the volcanic ash in this study (Fig. 1). *C. tetani* thrives in alkaline conditions, suggesting that soils near volcanoes and volcanic ash with acidic pH may not be suitable for most bacteria, including *C. tetani*.

Previous reports have suggested detection rates of 51.0% in the Tokyo area, 18.6% in the Okinawa and Southwest Islands, 34.4% in Kanazawa, and 22.9% in Sagamihara. The National Institute of Infectious Diseases reports an average isolation rate of 12% across 17 prefectures (8, 18). Therefore, we successfully isolated *C. tetani* with higher positivity and isolation rates than those previously reported. *C. tetani* was detected in the entire surveyed area, except in the volcanic ash region, indicating that *C. tetani* may be widespread nationwide rather than confined to a specific area.

### Mice experiment to confirm tetanus toxin

We injected the cultured supernatant from the *C. tetani* isolate into the left leg of the mice. In the presence of tetanus toxin production, the leg injection site exhibited proptosis. Strong toxin production induced left-sided body curvature and triggered tonic paralysis throughout the body of the mouse (Fig. 2). Considering the 3Rs, neutralization tests were performed on the selected 11 strains, which constituted the minimal set exhibiting tetanus toxin symptoms in the initial test. The symptoms of tetanus toxin were specific, and all mice injected with a mixture of the supernatant and antitoxin survived without any observed symptoms. Since the antitoxin effectively neutralized the toxin in all 11 strains, the 3R principles were upheld, and there was no need for further neutralization tests.

Tetanus toxin production has been an absolute criterion for the identification of *C. tetani*. Our laboratory is currently conducting mouse studies. In consideration of animal welfare guidelines such as those from the World Health Organization (WHO) and domestic animal welfare and management laws, there is a mandate to develop non-animal testing methods.

### Phylogenetic analysis based on draft genome sequences

**Fig 3:**
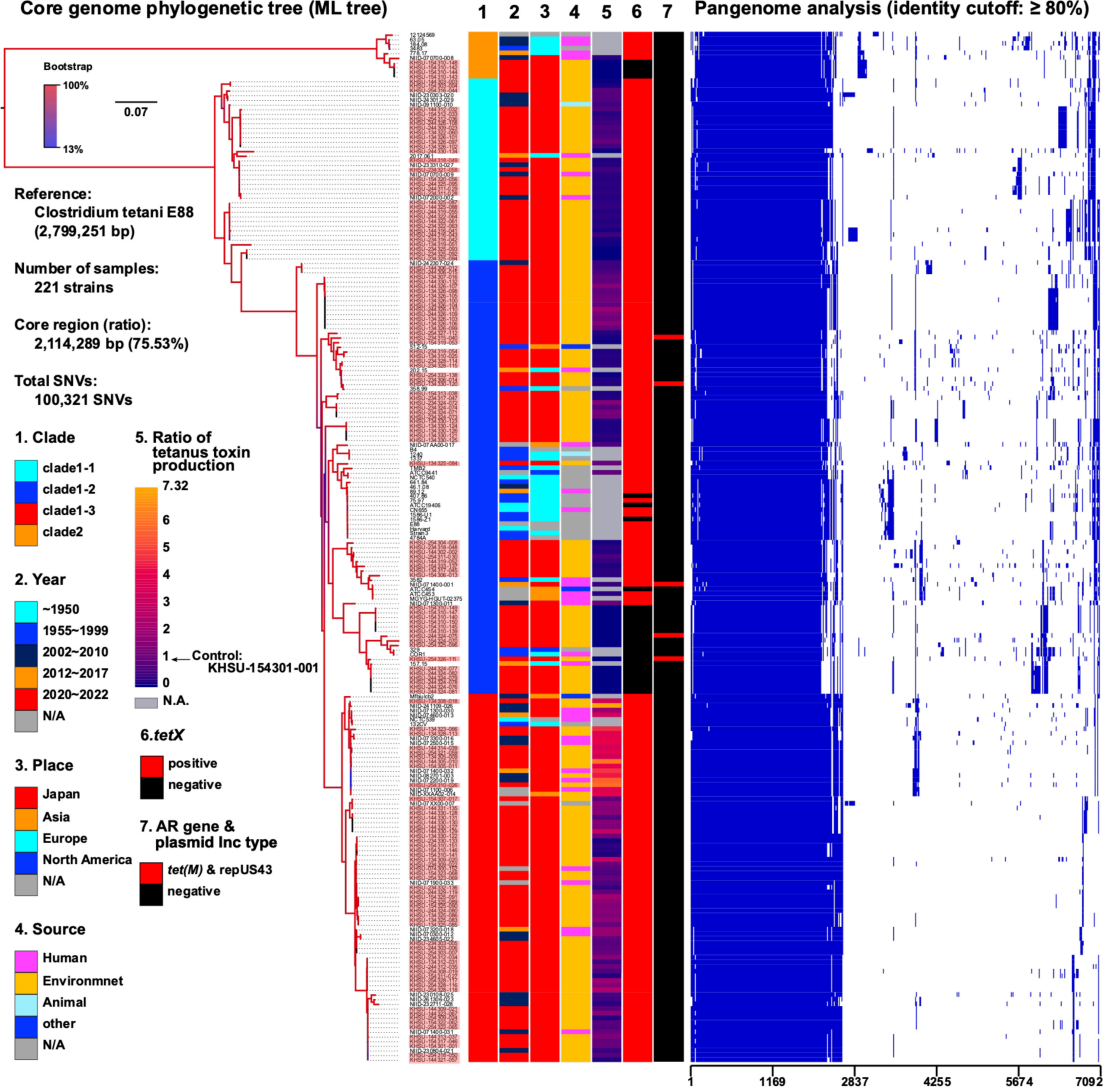
Core-genome phylogenetic analysis of *Clostridium tetani*. The core-genome region of 221 strains (185 and 36 strains in this study and published ones, respectively [Table S1]) was estimated to comprise 75.53% of the total genome. All strains were classified into clades 1 and 2. Clade 1 was subclassified into clades 1-1, 1-2, and 1-3. The Kumamoto strains (highlighted in red background) belonged to all clades (lane 1). *C. tetani* strains in clade 1-3 exhibited relatively high toxin production (lane 5), with the highest toxin-producing strains clustered together. Pangenome analysis revealed shared characteristic genes among the highly toxin-producing strains in clade 1-3, suggesting potential clonality. Clusters of *tetX*-negative strains were identified in clade 1-2 and clade 2 (lane 6), while five tetracycline resistance gene *tet*(M)-positive strains were identified (lane 7). N/A, not available.

We conducted whole-genome sequencing on all 151 *C*. *tetani* strains isolated from the soil in this study, including 34 previously reported isolates (Table S1). Single-nucleotide variations (SNVs) were extracted to analyze the mutational sites in the core-genome sequences of 221 strains, including 36 available *C. tetani* genome sequences (Fig. 3; Table S1). The genome size of *C. tetani* E88 (GenBank ID: NC_004557.1), which was used as the reference sequence, was 2,799,251 bp. The resulting core- genome sequence region accounted for 2,114,289 bp (75.53%). The total number of SNVs identified for all the strains was 100,321. Comprehensive core-genome SNV analysis showed that the domestic *C. tetani* and Kumamoto strains belong to most of the previously categorized clades 1 and 2 (14). In this study, we suggest further subdivision of clade 1 into three additional subclades (clades 1-1, 1-2, and 1-3) (Fig. 3). The 185 strains used in this study were composed of five in clade 2, 38 in clade 1-1, 66 in clade 1-2, and 76 in clade 1-3. In the Kumamoto Prefecture, clade 1-related strains were more prevalent (Fig. 1B) compared with clade 2.

Most European strains (see cyan squares in lane 3, Fig. 3) were isolated before 1999 (see lane 2 in Fig. 3), and most were genetically classified in the middle branch of clade 1-2 (see lane 1 in Fig. 3). A domestic Kumamoto isolate (KHSU-134325-084) was classified within the European isolates (clade 1-2), implying the possible presence of old European-related clones in Kumamoto Prefecture, Japan. Overall, these clades did not exhibit local specificity because they were isolated from Japan and other countries. Clade 1-3 isolates were frequently detected in clinical isolates (Table 2; see magenta square in lane 4, Fig. 3), and some of these clade 1-3 isolates exhibited relatively higher toxin-producing features than the other isolates (see orange/red square in lane 5; Fig. 3 and 5). TeNT expression was quantitatively determined using ELISA and RNA-Seq analyses, as further described (see Fig. 5 and 6).

**TABLE 2:** Correlations between clade 1 subclades and clinical isolates

	Human	Other	Total
Clade1-1	2	36	38
Other	16	110	126
*P*＝0.2494	18	146	164
Clade1-2	4	46	50
Other	14	100	114
*P*＝0.5890	18	146	164
Clade1-3	12	64	76
Other	6	82	88
*P*＝0.3184	18	146	164
Clade1-3 (toxin high production)	7	12	19
Other	11	134	145
*P*＝0.0013	18	146	164

Furthermore, 13 strains (KHSU-154310-139 to -151) were isolated from the same sampling site under identical selection conditions, whereas they belonged to different clades (clades 1-2, 1-3, and 2), indicating the coexistence of multiple lineages of *C. tetani* isolates at the same sampling site. Hence, it is plausible to observe diverse lineages of *C. tetani* strains even within a single sampling location, suggesting that these variations may not correlate with location-dependent lineages. Furthermore, we did not identify a country-specific clonal strain, as all strains obtained in Kumamoto Prefecture were closely related to other global strains (lane three in Fig. 3).

In this study, we identified 23 and 4 *tetX*-negative isolates in clades 1-2 and 2, respectively (Fig. 1C; black square in lane 6, Fig. 3). These strains have been found in both Japan and around the world, with five European isolates located close to the phylogenetic branch of the Japanese isolates and exhibiting *tetX*-negative traits. *C. tetani* carries the tetanus toxin gene *tetX*, which resides within the plasmid (12, 14). This study revealed that *tetX*-negative isolates seemed to also lack the *tetR* regulator gene, a component of the plasmid, as explained later in the findings (see Fig. 8). It remains uncertain whether the *tetR*/*tetX* genes can be deleted from the plasmid. Alternatively, these gene-negative plasmids might represent ancestral original plasmids that predate the acquisition of *tetR*/*tetX* genes in the *C. tetani* species. It remains unclear how these *tetX*-negative isolates lost the *tetX* gene from the plasmid. However, it appears that two separate deletion events occurred independently within clades 1-2 and 2, associated with distinct phylogenetic lineage.

### Determination of TeNT expression using ELISA and RNA-Seq analysis

**Fig 4:**
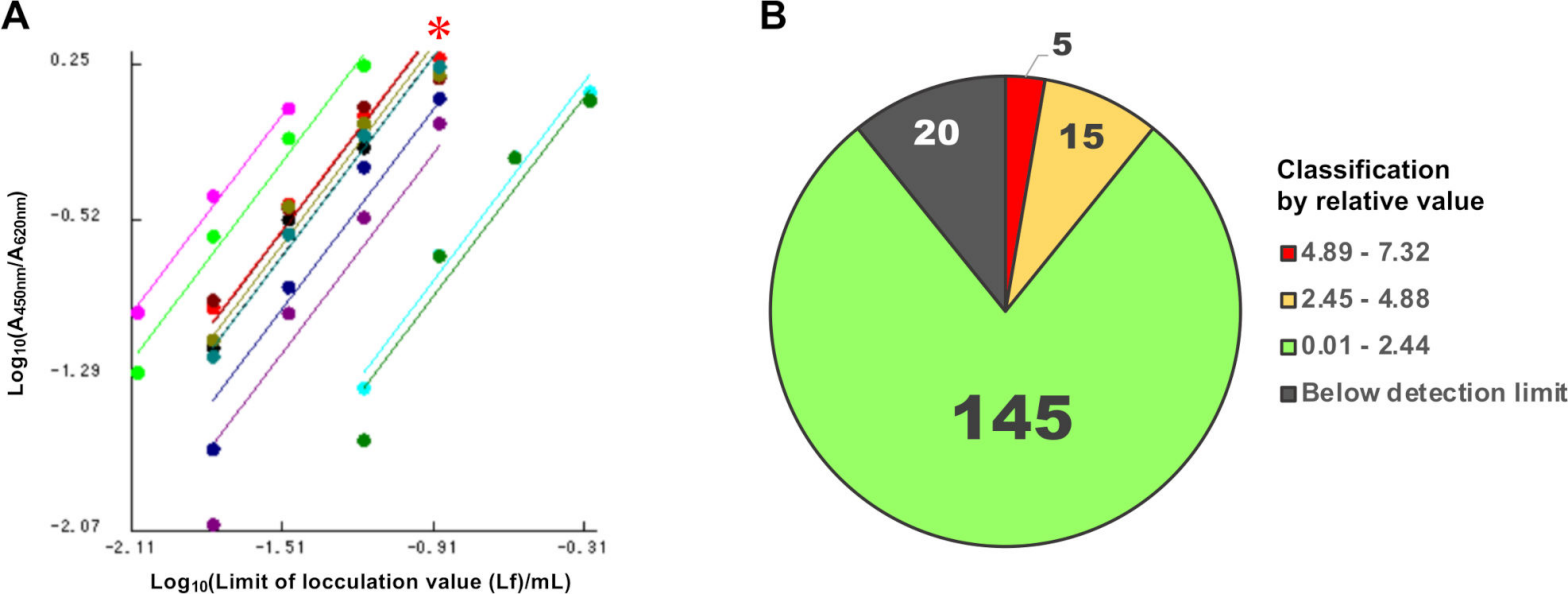
Comparative analysis using the parallel line quantification method. (**A**) Ten samples were partially selected from the pool of 185 strains for simultaneous measurement. The initial *C. tetani* isolate (KHSU-154301-001), marked with an asterisk, served as the representative standard among 151 identified standard strains. (**B**) Based on the results of 185 strains analyzed utilizing the parallel line quantification method, the results are categorized into four groups: high, medium, low, and below the detection limit. The results showed that the amount of toxin varied among the strains.

**Fig 5:**
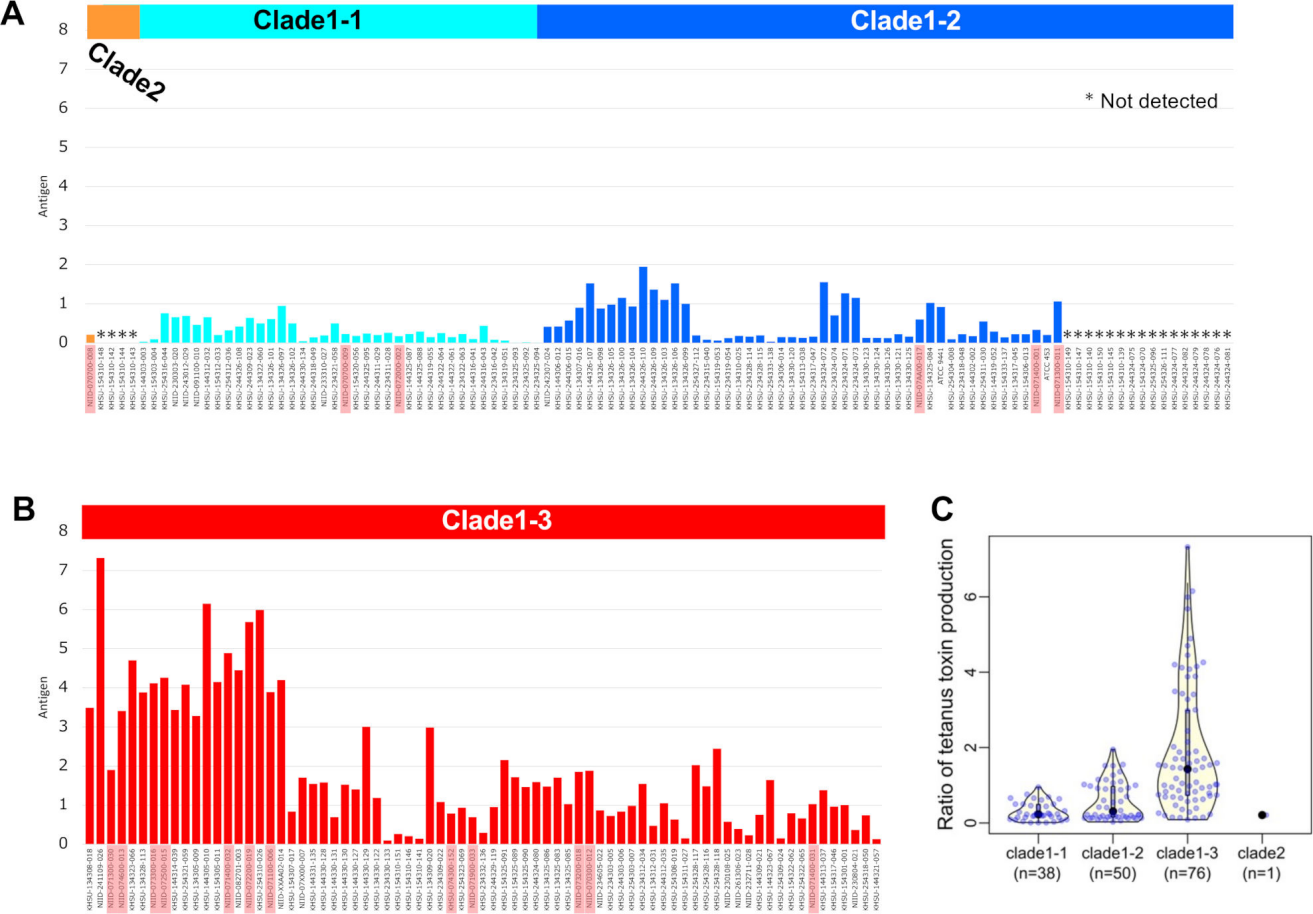
Comparison of antigen levels among the genomic clades.. Tetanus toxin levels were assessed via ELISA for the (**A**) clade 2, clade 1-1, clade 1-2, and (**B**) clade 1-3 strains. Red denotes clinical isolates. The asterisks (*) indicate values below the detection limit. Whole-genome sequencing revealed that all strains showing no detection (*) were *tetX* negative (lane 6 in Fig. 3). (**C**) Clade 1-3 strains exhibited relatively higher toxin production than the other clade strains, indicating an overall higher toxin production capacity.

The animal test, which employs paralysis and lethality as indicators of the biological activity of the toxin, is the international standard method for identifying *C. tetani*. In this study, we aimed to establish a test that eliminates the need for mice by performing bacteriological (biochemical properties, migration, and spore formation ability of bacteria), biochemical, and genetic tests to obtain a comprehensive judgment (Fig. 2). Furthermore, we aimed to improve the accuracy and sensitivity of the tetanus toxin test by combining PCR with a tetanus toxin-specific ELISA developed in our laboratory. The levels of toxin antigen in 183 isolates and 2 strains (ATCC 454 and ATCC 9441) were determined by comparing their expression to the reference strain (KHSU-154301-001), which was set at 1.0. After categorizing the values into low, medium, and high (Fig. 4), 145 strains fell within the 0.01–2.44 range, 15 in the 2.45–4.88 range, and 5 in the 4.89–7.32 range. Additionally, 20 strains were below the detection limit (indicated as “not detected” in Fig. 5). Further analysis revealed that all 20 strains showing no detection of the TeNT antigen were *tetX* negative according to whole-genome sequencing (Fig. 3). This suggests that our customized ELISA enabled the detection of TeNT expression in all *tetX*-positive isolates. However, we did not accurately evaluate the detection limit of the TeNT.

Most of the strains belonging to clades 1-1, 1-2, and 2 were below the standard strain (KHSU-154301-001), while 77 strains were higher than the standard strain, predominantly falling under clade 1-3. Additionally, clinical strains appeared to exhibit high toxin production, with 5 of the 19 strains (NIID-071100-006, NIID-074600-013, NIID-072500-015, NIID-072200-019, and NIID-071400-032) exhibiting toxin levels ranging from 2.45 to 7.32. By analyzing the ELISA antigen levels among the different genomic clades (Fig. 5C), a Mann–Whitney U test was conducted between the clade 1-3 group (*n* = 76) and the other groups (*n* = 109), showing a significant difference in toxin production (*P* < 0.01), although the amino acid sequence of *tetX* remained the same among the tested strains. In terms of genomic phylogeny, seven clinical isolates (NIID-071100-006, NIID-074600-013, NIID-072500-015, NIID-073300-016, NIID-072200-019, NIID-071300-030, and NIID-071400-032) were closely clustered together within clade 1-3, exhibiting a higher production of tetanus toxin (Fig. 5B). In addition, Fisher’s exact test was performed to determine whether the high toxin-producing strains were characteristic of the clinical isolates (Table 2). When comparing clades 1-1, 1-2, and 1-3 with the others, there were no significant differences (*P* = 0.2494, 0.5890, and 0.3184, respectively). However, there was a significant difference between the strains with high toxin production in clade 1-3 (>2.0) and the other strains (*P* = 0.0013). The minimum lethal doses (MLD) of KHSU-144303-003 and KHSU-144305-010 were 0.002 and 3.5, respectively, which were approximately equivalent to the ELISA results (0.001 and 3.46 for KHSU-144303-003 and KHSU-144305-010, respectively).

**Fig 6:**
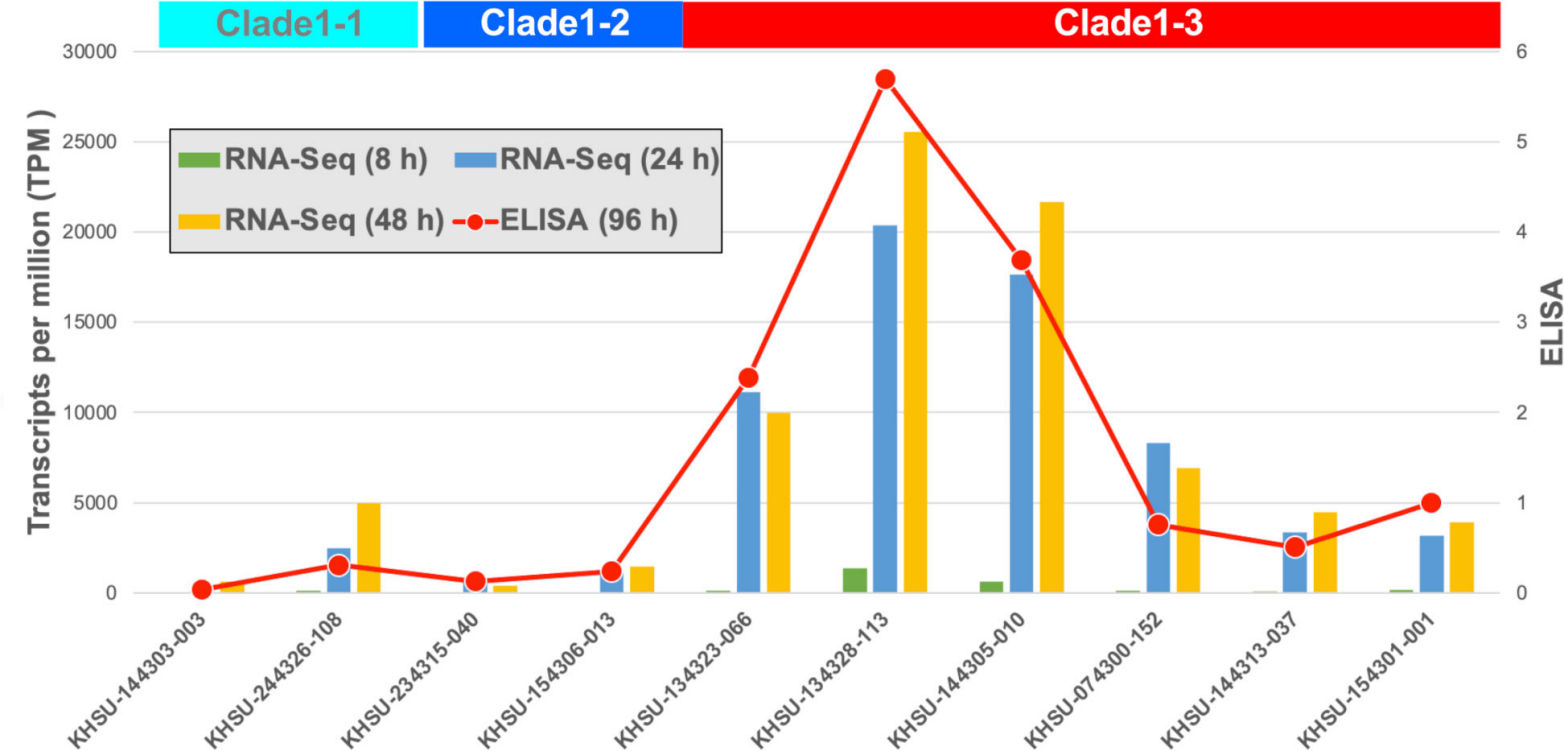
Comparison of RNA expression and toxin antigen levels. The *tetX* RNA expression levels in the tested isolates were compared after 8, 24, and 48 h of cultivation and the toxin antigen levels of 96-h culture supernatants. Expression levels were low at 8 h but increased at 24 and 48 h. The transcripts per million (TPM) value was correlated with the amount of toxin antigen at 96 h using ELISA. The results also showed that the high toxin-producing strains of clade1-3 lineages (KHSU-144305-010, KHSU-134323-066, and KHSU-134328-113) exhibited significantly higher mRNA expression levels than the other strains.

RNA-Seq analysis was performed to determine growth-dependent *tetX* mRNA expression after 8, 24, and 48 h of incubation (Fig. 6; Table S2), compared to toxin antigen levels determined by ELISA after 96 h of incubation (Fig. 5). Some of clade 1-3 (KHSU-134323-066, KHSU-134328-113, and KHSU-144305-010) exhibited significantly higher expression levels after 24 h of incubation, correlating with increased toxin production after 96 h of incubation. A comparison of the mRNA expression and ELISA results showed that both were positively correlated. The *tetX* mRNA was expressed at an early growth stage in the clade 1-3 strains (KHSU-134328-113 and KHSU-144305-010) (Fig. 6). Most of the clade 1-3 strains seem to exhibit higher tetanus toxin production compared to other subclades (lane 5 in Fig. 3 and 5). Additionally, clade 1-3 includes more clinical isolates (lane 5 in Fig. 3 and 5), suggesting that these highly toxin-producing strains have the potential to cause tetanus.

Pan-genome analysis suggested that highly toxin-producing strains shared certain common genes (phage-related and restriction-modification systems, methyltransferases, and transcriptional regulators) (Fig. 3); however, their direct involvement in *tetX* gene expression remains unclear. Based on the core-genome SNV analysis (Fig. 3), these strains seemed to be clonal, indicating similarities in pan-genome sequence elements among them. Therefore, we further investigated extracting a clade 1-3-specific nucleotide mutation by focusing on the regulatory systems for *tetX* gene expression (*tetR*, *codY*, RS04785, RS05745, RS07315, RS10155, and the promoter-binding site) (19). Significantly, the RS04785 amino acid sequence in clade 1-3 exhibited marked non-synonymous mutations: D222N (aspartic acid to asparagine at 222 amino acid positions) in the DNA-binding region and T45I (threonine to isoleucine at 45 amino acid positions) in the two-component system receiver domain. However, whether these non-synonymous mutations are involved in the high expression of *tetX* remains to be elucidated.

### Comparative genomic analysis based on complete genome sequences

**Fig 7:**
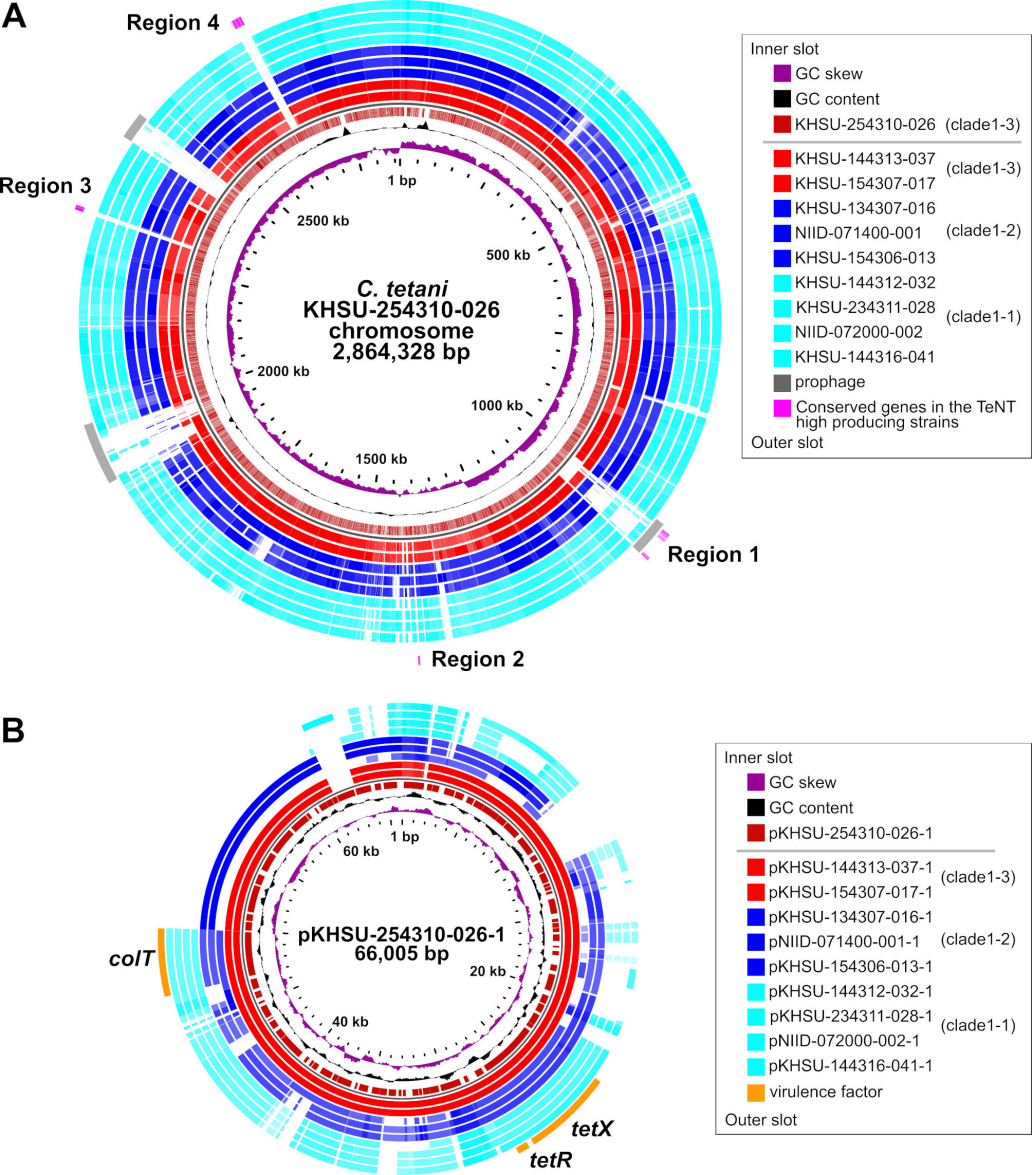
BLAST Atlas results for chromosomes and plasmids. Of the 10 strains with complete genome sequences, the high-TeNT-producing strain KHSU-254310-026 served as the reference, and it was compared using a BLASTn homology search for chromosome (**A**) and plasmid (**B**) sequences. The reference strain KHSU-254310-026 had three prophages, and four regions exhibited conservation among the TeNT high toxin-producing clade 1-3 strains: region 1 had 28 highly conserved genes in the terminal portion of the prophage; region 2 comprised 2 genes; region 3 contained 3 genes; and region 4 had 13 genes (Table S3).

Based on the basic information from the draft genome sequences (Fig. 3) and TeNT expression (Fig. 5), we selected 10 strains from clade 1 to complete the genome sequence (Table 3). Among the 10 complete genome sequences, KHSU-254310-026 was used as a reference for Basic Local Alignment Search Tool (BLAST) Atlas analysis (Fig. 7). This selection was based on its classification within clade 1-3 and its notable TeNT expression (5.99-fold compared with that of standard KHSU-154301-001). BLAST Atlas analysis identified four distinctive pan-genome regions within the chromosome, encoding most of the prophage sequences (Fig. 7A; Table S3) and the pan-genome region within the plasmid (Fig. 7B). No notable characteristic genes could predict why KHSU-254310-026 showed marked TeNT expression.

**TABLE 3:** Complete genomic data for 10 *C. tetani* strains isolated in Japan and putative replicon copy numbers

Clade	*C. tetani* strain name	Ratio of tetanus toxin production^*a*^	Replicon type	Complete sequence length (bp)	No. of CDS	Average read depth (Illumina short read)	Relative copy numbers to chromosome
Clade 1-1	KHSU-144316-041	0.1	Chromosome	2,863,790	2,780	349	1
			Plasmid	68,022	89	1,089	3
			Phage	138,965	179	943	3
	NIID-072000-002	0.17	Chromosome	2,761,480	2,632	577	1
			Plasmid	58,138	76	1,251	2
	KHSU-234311-028	0.26	Chromosome	2,814,725	2,687	297	1
			Plasmid	58,128	76	508	2
			Phage	45,581	72	928	3
	KHSU-144312-032	0.66	Chromosome	2,849,129	2,738	243	1
			Plasmid	61,222	78	622	3
Clade 1-2	KHSU-154306-013	0.22	Chromosome	2,801,574	2,609	2,401	1
			Plasmid	94,849	118	3,189	1
	NIID-071400-001	0.33	Chromosome	2,810,882	2,673	301	1
			Plasmid	112,887	134	1,455	5
	KHSU-134307-016	0.9	Chromosome	2,771,884	2,623	329	1
			Plasmid	67,592	83	1,205	4
Clade 1-3	KHSU-154307-017	0.83	Chromosome	2,751,438	2,607	1,521	1
			Plasmid	66,982	80	4,307	3
	KHSU-144313-037	1.38	Chromosome	2,734,951	2,545	322	1
			Plasmid	66,968	80	407	1
			Phage	51,784	81	257	1
	KHSU-254310-026	5.99	Chromosome	2,864,328	2,722	285	1
			Plasmid	66,005	81	1,126	4

^*a*^ The ratio is also shown in Fig. 5.

We also speculated that the copy number of each *tetX* plasmid might be involved in TeNT expression; however, we did not observe a positive correlation between them. For instance, KHSU-254310-026, which had four plasmid copies, exhibited the highest TeNT production (5.99-fold) compared to one chromosome copy. Conversely, KHSU-134307-017, with three plasmid copies, showed moderate TeNT production (0.83-fold) (Table 3). As described above, clade 1-3-specific nucleotide mutations may be involved in TeNT expression.

### Antimicrobial susceptibility to tetracycline

Antimicrobial resistance analysis revealed the presence of the tetracycline resistance gene *tet*(M) encoding the ribosomal protection protein in five isolates within clade 1-2: KHSU-234315-040, KHSU-244324-075, KHSU-254326-111, KHSU-134330-120, and NIID-071400-001 (Fig. 1D; see lane 7 in Fig. 3). The *tet*(M) gene was initially discovered in *Enterococcus* species through plasmid-mediated horizontal gene transfer (20). In addition to the findings based on whole-genome sequencing, antimicrobial susceptibility tests revealed that the *tet*(M)-positive isolates showed smaller inhibition zones for tetracycline, ranging from 9 to 30 mm, compared with wild-type isolates ranging from 45 to 50 mm (Table 4). This suggests that *tet*(M)-positive strains may be categorized as tetracycline-resistant, although no breakpoint for *C. tetani* has been suggested by the Clinical Laboratories Standards Institute (CLSI) and the European Committee on Antimicrobial Susceptibility Testing. Among the antimicrobial agents used in this study, gentamicin was not effective against *Clostridium* spp. because of the nature of obligate anaerobe. Penicillin, which is used to treat tetanus, exhibited a notably large inhibition zone, and ampicillin was equally effective.

**TABLE 4:** Zone of inhibition (mm) using antimicrobial agent disk (µg)^*a*^

Strain name	*tet*(M)	TC (30)	CP (30)	GM (10)	EM (15)	ABP (10)	PCG (10U)
KHSU-154301-001	−	50	38	−	40	43	46
KHSU-144303-003	−	46	33	−	30	35	43
KHSU-254325-096	−	49	32	−	34	42	43
KHSU-154310-142	−	45	36	−	36	45	47
KHSU-234315-040	+	22	34	−	30	38	38
KHSU-244324-075	+	30	33	−	40	45	49
KHSU-254326-111	+	10	34	−	38	46	51
KHSU-134330-120	+	9	31	−	34	47	48
NIID-071400-001	+	18	33	−	32	45	47

^*a*^ ABP, ampicillin; CP, chloramphenicol; EM, erythromycin; GM, gentamicin; PCG, penicillin G; TC, tetracycline.

**Fig 8:**
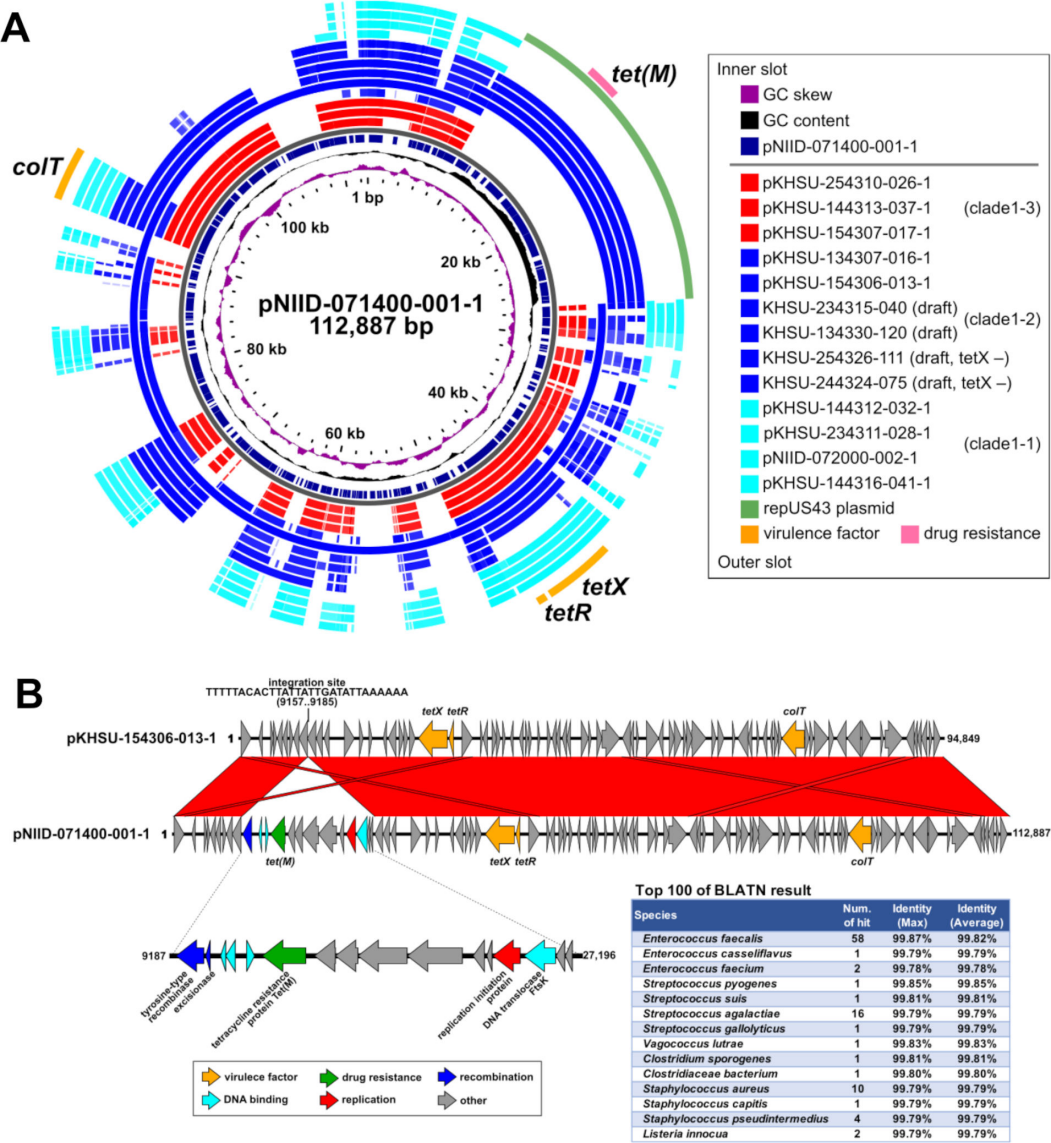
Horizontal acquisition of tetracycline resistance gene *tet*(M) in the *tetX* plasmid. (**A**) Compared to the complete 10 strains, five *tet*(M)-positive five strains (KHSU-234315-040, KHSU-244324-075, KHSU-254326-111, KHSU-134330-120, and NIID-071400-001) belonged to clade1-2; however, they did not exhibit clonality (lane 7, Fig. 3). Additionally, the *tet*(M)-positive plasmids were not identical, revealing different plasmid organization. (**B**) The acquired *tet*(M) element contains a repUS43 replicon, indicating that the *tet*(M) gene may be acquired independently through horizontal gene transfer. A homology search of the *tet*(M) element indicated high homology to Gram-positive bacteria, particularly *Enterococcus* and *Streptococcus*.

All five *tet*(M)-positive strains were isolated from various sampling sites, four of which were isolated from the Kumamoto Prefecture (Fig. 1D). The *tet*(M)-positive plasmid, pNIID-071400-001-1, served as the reference in BLAST analysis (Fig. 8A), revealing that the *tet*(M) element measured 18 kb in size (Fig. 8B) and likely integrated into the *tetX*-plasmid from the repUS43 replicon plasmid (see green bar in Fig. 8A). The *tet*(M) gene has been widely identified in various Gram-positive cocci (Fig. 8B), including *Enterococcus*, *Staphylococcus*, *Streptococcus*, *Lactobacillus*, and a part of the Gram-negative bacterium *Enterobacteriaceae* (CARD website: https://card.mcmaster.ca/ontology/36325). The occurrence of horizontal gene transfer among Gram-positive bacteria suggests that *C. tetani* may have the capacity to acquire relevant resistance genes from other Gram-positive bacteria when exposed to selective pressure from tetracycline in livestock, soil, or water environments. Two *tet*(M)-positive strains (KHSU-254326-111 and KHSU-244324-075; Fig. 3 and 8A) lacked the *tetX* gene in the plasmid, suggesting a potential prioritization of tetracycline resistance [*tet*(M)] over virulence (*tetX*) in these strains.

### Conclusions

We examined a large number of *C. tetani* isolates from a confined region in Kumamoto Prefecture, highlighting distinctive genomic characteristics. Our findings were further supported using ELISA, mouse experiments, and RNA-Seq analyses. *C. tetani* isolates in Kumamoto Prefecture exhibit genetic diversity and can be found in a relatively confined geographical area. This prompts the question of how global *C. tetani* isolates may spread throughout such a small area in Kumamoto Prefecture. Additionally, we identified a clonal clade of *C. tetani* characterized by high TeNT production, frequently found in clinical specimens. This leads to the second question: what mechanism drives high TeNT production in clade 1-3? In contrast, *tetX*-negative *C. tetani* isolates were found in clades 1-2 and 2, suggesting that TeNT may be dispensable for the life cycle of *C. tetani*. To the best of our knowledge, this is the first study to reveal horizontally acquired tetracycline resistance in *C. tetani*. However, antimicrobial treatment is not a crucial primary regimen for tetanus. The newly obtained whole-genome data and toxin productivity assessments presented in this study offer valuable insights into *C. tetani* pathogenesis. However, they also open up additional questions, particularly regarding how *C. tetani* thrives and effectively adapts within the soil ecosystem to trigger tetanus.

## MATERIALS AND METHODS

Soil sampling, *C. tetani* culture, isolation, and identification followed the techniques employed for laboratory tetanus diagnosis as previously described (8).

### Soil sampling

Soil was collected from the ground surface and 10 cm below the ground at 46 arbitrarily sites within Kumamoto Prefecture (Fig. 1). Furthermore, 10–15 g of soil was collected in 15-mL conical tubes (Labcon, North America) using sterile medicine spoons. Four of the sampled sites were on roads or rocky terrain that was too hard for digging. Consequently, only samples from the ground surface were collected. Overall, 264 soil samples were cultured and stored at 4°C for 1–2 months for subsequent use.

### Selective preculture treatment

*C. tetani* was isolated using a cooked meat medium (CMM; BD Difco, Tokyo, Japan). The soil was left to reach room temperature for approximately 30 min. Subsequently, 1 g of soil was weighed and added to each of the three CMMs, ensuring gentle handling to avoid vigorous shaking or introducing air to maintain anaerobic conditions. The CMMs underwent three different treatments: (i) heating to 80°C for 10 min, (i) heating to 60°C for 10 min, and (iii) unheating and incubating anaerobically at 37°C for 48–72 h for subsequent bacteriological, genetic, biochemical, and immunological tests.

### Isolation of *C. tetani*

The CMM culture was transferred onto a blood agar medium using an inoculation loop and then incubated anaerobically at 37°C for 18–24 h, utilizing an AnaeroPac Lab (SGI, Tokyo, Japan). When the bacterial preculture on the CMM contains numerous other soil-derived environmental bacteria, it could impede the growth or effective isolation of *C. tetani*. In such cases, to improve the isolation efficiency by utilizing the swarming potential of *C. tetani*, the preculture should be serially diluted (10^2^- to 10^6^-fold) and then cultivated on blood agar, resulting in swarming bacteria on the agar. Furthermore, raising the agar concentration to approximately 4% helped reduce excess migration, thereby creating better conditions for the swarming potential of each *C. tetani* isolate.

### Bacteriological, genetic, biochemical, and immunological tests

#### Bacteriological test

Given that *C. tetani* swarmed on the agar medium surface, resulting in a frizzled appearance at the tip, we collected the tip of potential *C. tetani* colonies with an inoculating loop for isolation. Subsequently, we transferred it to CMM, increased the bacteria population, and performed passages. The candidate *C. tetani* isolate was transferred to two blood agar media, and it was confirmed that only the anaerobic culture was collected, while the aerobic culture was discarded.

#### Species identification

The tetanus toxin gene *tetX* was detected using the PCR method described by Kato et al. The PCR primers for the gene encoding the l-chain of the tetanus toxin gene (accession no. X04436) included GAT1 (5′-GATGATACGTATGCCAATAACC-3′), GAT2 (5′-TAAGGCTTCACCTGCTACATTG-3′), GAT5 (5′-CTACATGGTTTATATACGGAATGCAGG-3′), and GAT6 (5′-GATCATTGCAGCTAGTGACTTGAC-3′) (21). The positive control used was the Harvard strain (Harvard A47), an internationally recognized vaccine production strain distributed by Osaka University. To prepare DNA extracts, 1 mL of the 48- to 72-h culture was collected using a Pasteur pipette and centrifuged at 6,000 rpm for 6 min. Sterile water (100 µL) was then added to the cell pellet and treated for 30 min at 100°C, followed by centrifugation at 6,000 rpm for 6 min. Finally, the supernatant was used as the cell lysate. PCR reagents were prepared following the manufacturer’s instructions. Subsequently, 2 µL of each sample, the positive and negative controls, was incorporated using the Emerald Amp PCR Master Mix (Takara Bio, Shiga, Japan). PCR was conducted through 35 cycles for 20 s at 95°C followed by 2 min at 55°C. The amplicon sizes were 331 and 229 bp for the GAT1/GAT2 and GAT5/GAT6 primer pairs, respectively. PCR products were confirmed through 2% agarose gel electrophoresis and ethidium bromide staining. Species identification tests for anaerobes were performed using a Rapid 32A Api (bioMérieux, Tokyo, Japan), following the manufacturer’s instructions.

#### Confirming toxicity and immunological (neutralization) studies in mice

Toxicity and immunological assessments were performed according to the *Pathogen Detection Manual* of the National Institute of Infectious Diseases (8, 22).

To prepare the sample, the CMM culture was centrifuged at 6,000 rpm for 6 min. The resulting supernatant was then filtered through a 0.2-µm membrane filter and subsequently diluted 100-fold with 0.2% gelatin and 17 mM phosphate-buffered saline (PBS) (pH 7.0). We subcutaneously injected filter-sterilized culture supernatant (0.5 mL) containing tetanus toxin into the left medial thigh of two mice per group and observed them for 4 days. If the sample contains active tetanus toxin, we observed tetanus-specific tonic paralysis in the mice. They succumbed to the toxin when exposed to a lethal dose.

In the neutralization test, equal quantities of horse serum-derived tetanus antitoxin (referred to as “antitoxin”) and the sample were mixed. The mixture was allowed to stand at room temperature for 1 h before injecting 0.5 mL subcutaneously into the inner left thigh of each mouse. The antitoxin (lyophilized, 1,200 IU/vial; supplied by KM Biologics) was dissolved in distilled water (Otsuka, Tokyo, Japan). It was diluted 60-fold to 20 IU/mL. Antitoxin neutralization was confirmed in surviving mice without exhibiting tetanus toxin symptoms. Laboratory animal handling for research and other purposes adhered to the reduction, replacement, and refinement (3Rs) principle and conformed to the Japanese Animal Welfare and Management Act and the International Organization for Animal Welfare and Management (WHO) (23).

### Determination of tetanus toxin antigen levels

We developed a sandwich ELISA for tetanus toxin to assess antigen levels quantitatively. The polyclonal anti-tetanus toxin antibody was generated by immunizing rabbits with the in-house tetanus toxoid, followed by affinity purification using tetanus toxin. An antibody-coated plate was created by immobilizing the affinity-purified anti-tetanus toxin polyclonal antibody on flat-bottomed Nunc Immuno Plates (Thermo Fisher Scientific, Tokyo, Japan) using 67 mM PBS and 0.1% sodium azide pH 7.4. It was allowed to stand for approximately 16 h at room temperature. Following three washes with 67 mM PBS (pH 7.4), we applied 320 µL of blocking reagent solution (1% bovine serum albumin, 5% lactose, 5% sucrose, 1% Ploclin300, and 2% Immunoblock [KAC, Kyoto, Japan] in 67 mM PBS). The solution was maintained at 25°C for 4 h and then at 4°C for at least 16 h. We removed the blocking reagent solution and desiccated the plates in a desiccator until the humidity was <35%. Subsequently, the plates were stored at 4°C in aluminum laminate with desiccant until they were needed.

Before utilization, the plate underwent three washes with 50 mM Tris-buffered saline and 0.05% Tween-20 pH7.5 (TBS-T). Tetanus toxin is mostly expressed during the initial *C. tetani* growth phase and subsequently secreted into the broth medium as an exotoxin. After ≥96-h incubation, the bacteria underwent autolysis (24).

For the antigen assay, *C. tetani* culture supernatant grown in brain heart infusion (BHI) medium at 37°C for 96 h was obtained. It was obtained following centrifugation at 3,500 rpm for 20 min and subsequently passed through a 0.2-µm membrane filter. The supernatants were serially diluted twofold with 67-mM PBS and 0.05% Tween-20. The horseradish peroxidase (Shigma, Tokyo, Japan)-labeled anti-tetanus toxin mouse monoclonal antibody (TH-11; Fujifilm, Tokyo, Japan) was diluted 1,000-fold using a conjugate diluent solution. The solution included 1% bovine serum albumin, 10% immobilized bovine serum, 1 mM ZnCl_2_, 1 mM MgCl_2_, 0.005% bromcresol purple, 1% ProClin 300, 50-µg/mL HAMA blocker, 1% Immunoblock, 0.02% Tween-20, 0.15 M NaCl, and 0.1 M Tris (pH7.2). Subsequently, 100 µL of diluted antibody solution was dispensed onto each plate. The diluted sample (50 µL) was added to each plate, and the plates were incubated for 2 h at 37°C. After five washes with TBS-T, 100-µL 3,3′,5,5′-tetramethylbenzidine (Nacalai, Kyoto, Japan) was added and incubated at 37°C for 30 min. Subsequently, 1 M sulfuric acid (Nacalai) was introduced to stop the reaction, and the absorbance was measured at A_450 nm_/A_620 nm_ within 15 min.

Since no suitable standard strains were available due to the new test method establishment, the initial *C. tetani* isolate strain, KHSU-154301-001, among the 151 isolates, was used as a positive control and standard reference, with a 1.0 expression value. The parallel line assay was utilized to assess the relative value of each strain compared to the standard KHSU-154301-001 using the optical density (OD) value endpoint. Raw data were evaluated using Bioassay Assist software for statistical analysis (provided by the National Institute of Infectious Diseases, Japan).

### Comparison of the toxin antigens in ELISA and toxicity in mouse

ELISA results were compared to mouse bioactivity. Samples served as standards for ELISA, and the three strains exhibited low and high toxin levels. Samples were prepared using 0.2% gelatin and 17-mM PBS (pH 7.0), diluted 5,000×, 10,000×, 20,000×, 40,000×, and 80,000× for standard strains; 5×, 10×, 20×, 40×, 80×, 100×, 200×, 400×, 800×, and 1,600× for low-value strains; and 17,500×, 50,000×, 70,000×, 140,000×, and 280,000× for high-value strain-fold dilutions. The endpoint was the MLD, where both mice succumbed. The relative value of each strain was determined using the strain used as the standard in ELISA as the positive control. The relative value for each strain was determined using the strain used as the standard in the ELISA as a positive control.

### Whole-genome *C. tetani* isolate sequencing

*C. tetani* culture and whole-genome sequencing were performed following the methods described by Kenri et al., Kato et al., and Sekizuka et al. (25–27).

Briefly, *C. tetani* isolate culture in BHI broth during 16- to 24-h incubation at 37°C was collected via centrifugation at 3,500 rpm for 20 min. The cell pellet was suspended in 450 µL of Tris-HCl EDTA (Nippon Gene, Tokyo, Japan), 50 µL of 10% sodium dodecyl sulfate SDS (Fujifilm, Osaka, Japan), and 500 µL of phenol chloroform (Nippon Gene). The phenol-inactivated mixture was transferred into a ZR BashingBead Lysis Tube (Zymo Research, Irvine, CA, USA). Bacterial cell disruption was achieved using the bead-beating method in a Geno Grinder at 1,500 rpm for 10 min. A bead-beating mixture was used to extract DNA and RNA. Genomic DNA was subsequently purified using the ZR-96 Zymoclean Gel DNA Recovery Kit (Zymo Research). DNA-Seq libraries were prepared using the QIAseq FX DNA Library Kit (QIAGEN, Tokyo, Japan). DNA-Seq was performed using the NextSeq 500/550 Mid Output Kit v2.5 (150-mer paired end, 300 cycles) and NextSeq 500 sequencer. Adapter and quality trimming of decoded reads were performed using Fastq-mcf v.1.04.636 and Sickle v.1.33 programs, respectively. Following read trimming, *de novo* assembly was performed using SKESA v.2.3.0, and gene annotation was conducted utilizing DFAST v.1.2.3, employing contig sequences obtained from the *de novo* assembly (28, 29).

### Comparative genome analysis

For core-genome SNV analysis, the *C. tetani* E88 chromosome (accession number NC_004557.1) was used as the reference sequence. Nucleotide substitutions were located through read mapping using VarScan v.2.3.4 (30, 31). The repeat regions of the chromosomal sequences were identified using NUCmer, followed by SNV elimination within these regions. SNVs within the common genomic region (core-genome region) of all tested strains were extracted and concatenated to form a pseudo-sequence. Core-genomic phylogenetic analysis was conducted using the maximum likelihood method with iqtree v.2.1.3 (32), and the resulting phylogenetic tree was created using FigTree v.1.4.4. TetX gene (*tetX*) multiple alignments were conducted using MAFFT v.7.475, followed by phylogenetic analysis using the iqTree v.2.1.3 (32, 33). For complete chromosome or plasmid sequences, BLAST analysis was performed using BLAST homology searches (34) and GView Server (https://server.gview.ca/) (35).

### RNA-Seq analysis

Total RNAs were purified from the bead-beating mixture using the miRNeasy Mini Kit (QIAGEN). Subsequently, RNA-Seq libraries were prepared utilizing the Zymo-Seq RiboFree Total RNA Library Kit (Zymo Research). RNA-Seq was conducted using NextSeq 1000/2000 P2 Reagents (150-mer paired end, 300 cycles), v3 cartridges, and a NextSeq 1000 sequencer. Following the core-genome phylogenetic analysis, 10 *C*. *tetani* strains were selected, representing each clade for further analysis. Cultures were prepared for 8, 24, and 48 h from the developmental stage to the spore formation stage and for 96 h to extract the exotoxin. RNA expression levels were evaluated as transcripts per million by read mapping to the coding sequences of *C. tetani* E88 (complete sequence, GenBank ID: NC_004557) using the CLC Genome Workbench v.22 software (QIAGEN).

### Antimicrobial agent susceptibility test

The Kirby–Bauer disk diffusion method was performed using KB Disk “Eiken” (Eiken Chemical, Tokyo, Japan)—a drug sensitivity kit—following the instructions of the manufacturer. Since no clear guidelines existed for antimicrobial susceptibility testing of *C. tetani*, we adapted the CLSI method with some modifications (36, 37). Following the inoculation of *C. tetani* into the blood agar medium via an inoculating loop, the culture was anaerobically incubated at 37°C for 18–24 h. Subsequently, the bacteria were adjusted to a McFarland turbidity of 0.5, using a turbidity standard solution, and they were evenly smeared over the entire blood agar medium using sterile swabs. Six antimicrobial disks (gentamicin, chloramphenicol, tetracycline, ampicillin, erythromycin, and penicillin) were positioned on the medium, ensuring a minimum spacing of 24 mm. The culture was subsequently incubated anaerobically at 37°C for 18–24 h. The bacteria were anaerobically incubated at 37°C for 18–24 h. The inhibition zone diameter was measured to confirm complete bacterial growth inhibition. Among the 151 isolates, *tet*(M)-negative strains were selected as potential tetracycline-susceptible strains.

## Data Availability

The BioSample IDs of the whole-genome sequences of 183 Kumamoto soil isolates, clinical isolates, and strains provided by the National Institute of Infectious Diseases, Japan, have been deposited in the DDBJ/EMBL/GenBank database (accession numbers: Table S1). All raw read sequence files can be accessed on the DDBJ Sequence Read Archive (DRA)/Sequence Read Archive (SRA) database (accession numbers: DRR403812–DRR404004 [whole-genome data; see Table S1]). Table 1 summarizes the whole-genome sequences of all *Clostridium tetani* strains used in this study.

## SUPPLEMENTAL MATERIAL

Table S1 msphere.00369-23-s0001.pdf Metadata and data deposition information of *Clostridium tetani* using this study.

Table S2 msphere.00369-23-s0002.pdf TPM gene expression determined by RNA-Seq analysis.

Table S3 msphere.00369-23-s0003.pdf Pan-genome regions and coding sequence list in the high toxin-producing strain KHSU-254310-026.

ASM does not own the copyrights to Supplemental Material that may be linked to, or accessed through, an article. The authors have granted ASM a non-exclusive, world-wide license to publish the Supplemental Material files. Please contact the corresponding author directly for reuse.
