# Supplementary material for: Comparative pathogenomic analysis reveals a highly tetanus toxin-producing clade of Clostridium tetani isolates in Japan
Source: mSphere. 2023 Nov 27;8(6):e00369-23. doi: 10.1128/msphere.00369-23 (PMC10732020; doi:10.1128/msphere.00369-23)
Supplement: Table S1 — Metadata and data deposition information of Clostridium tetani using this study. [file msphere.00369-23-s0001.pdf]

Table S1. Metadata and data deposition information of *Clostridium tetani* using this study

Strain name	Collection date (YYYY-MM-DD)	Location	Isolation source / Host	BioSample ID	Run ID	Assembly level	Sequence accession number	Description	clade	Ratio of tetanus toxin production (standard: KHSU-154301-001)	*tetX* gene	*tet(M)* gene & repUS43 plasmid
KHSU-154301-001	2020-08-28	Japan: Kumamoto	soil	SAMD00534290	DRR403812	raw read data	N/A	This study	clade1-3	1.00	+	—
KHSU-144302-002	2020-09-15	Japan: Kumamoto	soil	SAMD00534291	DRR403813	raw read data	N/A	This study	clade1-2	0.17	+	—
KHSU-144303-003	2020-09-15	Japan: Kumamoto	soil	SAMD00534292	DRR403814	raw read data	N/A	This study	clade1-1	0.03	+	—
KHSU-154303-004	2020-09-15	Japan: Kumamoto	soil	SAMD00534293	DRR403815	raw read data	N/A	This study	clade1-1	0.09	+	—
KHSU-234303-005	2020-09-15	Japan: Kumamoto	soil	SAMD00534294	DRR403816	raw read data	N/A	This study	clade1-3	0.72	+	—
KHSU-244303-006	2020-09-15	Japan: Kumamoto	soil	SAMD00534295	DRR403817	raw read data	N/A	This study	clade1-3	0.83	+	—
KHSU-254303-007	2020-09-15	Japan: Kumamoto	soil	SAMD00534296	DRR403818	raw read data	N/A	This study	clade1-3	0.98	+	—
KHSU-254304-008	2020-09-15	Japan: Kumamoto	soil	SAMD00534297	DRR403819	raw read data	N/A	This study	clade1-2	0.09	+	—
KHSU-134305-009	2020-09-15	Japan: Kumamoto	soil	SAMD00534298	DRR403820	raw read data	N/A	This study	clade1-3	3.28	+	—
KHSU-144305-010	2020-09-15	Japan: Kumamoto	soil	SAMD00534299	DRR403821	raw read data	N/A	This study	clade1-3	6.15	+	—
KHSU-154305-011	2020-09-15	Japan: Kumamoto	soil	SAMD00534300	DRR403822	raw read data	N/A	This study	clade1-3	4.15	+	—
KHSU-144306-012	2020-10-27	Japan: Kumamoto	soil	SAMD00534301	DRR403823	raw read data	N/A	This study	clade1-2	0.42	+	—
KHSU-154306-013	2020-10-27	Japan: Kumamoto	soil	SAMD00534302	DRR403824, DRR403997	complete	AP026814-AP026815 (INSD ID)	This study	clade1-2	0.22	+	—
KHSU-234306-014	2020-10-27	Japan: Kumamoto	soil	SAMD00534303	DRR403825	raw read data	N/A	This study	clade1-2	0.15	+	—
KHSU-244306-015	2020-10-27	Japan: Kumamoto	soil	SAMD00534304	DRR403826	raw read data	N/A	This study	clade1-2	0.57	+	—
KHSU-134307-016	2020-10-27	Japan: Kumamoto	soil	SAMD00534305	DRR403827, DRR403998	complete	AP026804-AP026805 (INSD ID)	This study	clade1-2	0.90	+	—
KHSU-154307-017	2020-10-27	Japan: Kumamoto	soil	SAMD00534306	DRR403828, DRR403999	complete	AP026816-AP026817 (INSD ID)	This study	clade1-3	0.83	+	—
KHSU-134308-018	2020-11-13	Japan: Kumamoto	soil	SAMD00534307	DRR403829	raw read data	N/A	This study	clade1-3	3.49	+	—
KHSU-254308-019	2020-11-13	Japan: Kumamoto	soil	SAMD00534308	DRR403830	raw read data	N/A	This study	clade1-3	0.63	+	—
KHSU-134309-020	2020-11-13	Japan: Kumamoto	soil	SAMD00534309	DRR403831	raw read data	N/A	This study	clade1-3	2.98	+	—
KHSU-144309-021	2020-11-13	Japan: Kumamoto	soil	SAMD00534310	DRR403832	raw read data	N/A	This study	clade1-3	0.75	+	—
KHSU-234309-022	2020-11-13	Japan: Kumamoto	soil	SAMD00534311	DRR403833	raw read data	N/A	This study	clade1-3	1.08	+	—
KHSU-244309-023	2020-11-13	Japan: Kumamoto	soil	SAMD00534312	DRR403834	raw read data	N/A	This study	clade1-1	0.64	+	—
KHSU-254309-024	2021-02-20	Japan: Kumamoto	soil	SAMD00534313	DRR403835	raw read data	N/A	This study	clade1-3	0.15	+	—
KHSU-134310-025	2020-12-14	Japan: Kumamoto	soil	SAMD00534314	DRR403836	raw read data	N/A	This study	clade1-2	0.18	+	—
KHSU-254310-026	2020-12-14	Japan: Kumamoto	soil	SAMD00534315	DRR403837, DRR404000	complete	AP026821-AP026822 (INSD ID)	This study	clade1-3	5.99	+	—
KHSU-154311-027	2020-12-14	Japan: Kumamoto	soil	SAMD00534316	DRR403838	raw read data	N/A	This study	clade1-3	0.15	+	—
KHSU-234311-028	2020-12-14	Japan: Kumamoto	soil	SAMD00534317	DRR403839, DRR404001	complete	AP026818-AP026820 (INSD ID)	This study	clade1-1	0.26	+	—
KHSU-244311-029	2020-12-14	Japan: Kumamoto	soil	SAMD00534318	DRR403840	raw read data	N/A	This study	clade1-1	0.20	+	—
KHSU-254311-030	2020-12-14	Japan: Kumamoto	soil	SAMD00534319	DRR403841	raw read data	N/A	This study	clade1-2	0.55	+	—
KHSU-134312-031	2021-02-16	Japan: Kumamoto	soil	SAMD00534320	DRR403842	raw read data	N/A	This study	clade1-3	0.47	+	—
KHSU-144312-032	2020-12-14	Japan: Kumamoto	soil	SAMD00534321	DRR403843, DRR404002	complete	AP026806-AP026807 (INSD ID)	This study	clade1-1	0.66	+	—
KHSU-154312-033	2021-03-22	Japan: Kumamoto	soil	SAMD00534322	DRR403844	raw read data	N/A	This study	clade1-1	0.20	+	—
KHSU-234312-034	2021-02-16	Japan: Kumamoto	soil	SAMD00534323	DRR403845	raw read data	N/A	This study	clade1-3	1.54	+	—
KHSU-244312-035	2020-12-14	Japan: Kumamoto	soil	SAMD00534324	DRR403846	raw read data	N/A	This study	clade1-3	1.05	+	—
KHSU-254312-036	2021-02-16	Japan: Kumamoto	soil	SAMD00534325	DRR403847	raw read data	N/A	This study	clade1-1	0.32	+	—
KHSU-144313-037	2020-12-14	Japan: Kumamoto	soil	SAMD00534326	DRR403848, DRR404003	complete	AP026808-AP026810 (INSD ID)	This study	clade1-3	1.38	+	—
KHSU-154313-038	2020-12-14	Japan: Kumamoto	soil	SAMD00534327	DRR403849	raw read data	N/A	This study	clade1-2	0.13	+	—
KHSU-144314-039	2021-02-25	Japan: Kumamoto	soil	SAMD00534328	DRR403850	raw read data	N/A	This study	clade1-3	3.43	+	—
KHSU-234315-040	2021-01-25	Japan: Kumamoto	soil	SAMD00534329	DRR403851	raw read data	N/A	This study	clade1-2	0.08	+	+
KHSU-144316-041	2021-01-25	Japan: Kumamoto	soil	SAMD00534330	DRR403852, DRR404004	complete	AP026811-AP026813 (INSD ID)	This study	clade1-1	0.10	+	—
KHSU-234316-042	2021-01-25	Japan: Kumamoto	soil	SAMD00534331	DRR403853	raw read data	N/A	This study	clade1-1	0.08	+	—
KHSU-244316-043	2021-01-25	Japan: Kumamoto	soil	SAMD00534332	DRR403854	raw read data	N/A	This study	clade1-1	0.44	+	—
KHSU-254316-044	2021-02-16	Japan: Kumamoto	soil	SAMD00534333	DRR403855	raw read data	N/A	This study	clade1-1	0.76	+	—
KHSU-134317-045	2021-02-16	Japan: Kumamoto	soil	SAMD00534334	DRR403856	raw read data	N/A	This study	clade1-2	0.22	+	—
KHSU-154317-046	2021-01-25	Japan: Kumamoto	soil	SAMD00534335	DRR403857	raw read data	N/A	This study	clade1-3	0.96	+	—
KHSU-234317-047	2021-01-25	Japan: Kumamoto	soil	SAMD00534336	DRR403858	raw read data	N/A	This study	clade1-2	0.16	+	—
KHSU-234318-048	2021-02-25	Japan: Kumamoto	soil	SAMD00534337	DRR403859	raw read data	N/A	This study	clade1-2	0.22	+	—
KHSU-244318-049	2021-02-25	Japan: Kumamoto	soil	SAMD00534338	DRR403860	raw read data	N/A	This study	clade1-1	0.14	+	—
KHSU-254318-050	2021-02-25	Japan: Kumamoto	soil	SAMD00534339	DRR403861	raw read data	N/A	This study	clade1-3	0.74	+	—
KHSU-134319-051	2021-02-25	Japan: Kumamoto	soil	SAMD00534340	DRR403862	raw read data	N/A	This study	clade1-1	0.06	+	—
KHSU-144319-052	2021-03-22	Japan: Kumamoto	soil	SAMD00534341	DRR403863	raw read data	N/A	This study	clade1-2	0.29	+	—
KHSU-154319-053	2021-02-25	Japan: Kumamoto	soil	SAMD00534342	DRR403864	raw read data	N/A	This study	clade1-2	0.06	+	—
KHSU-234319-054	2021-02-25	Japan: Kumamoto	soil	SAMD00534343	DRR403865	raw read data	N/A	This study	clade1-2	0.12	+	—
KHSU-244319-055	2021-02-25	Japan: Kumamoto	soil	SAMD00534344	DRR403866	raw read data	N/A	This study	clade1-1	0.15	+	—
KHSU-154320-056	2021-02-25	Japan: Kumamoto	soil	SAMD00534345	DRR403867	raw read data	N/A	This study	clade1-1	0.18	+	—
KHSU-144321-057	2021-03-22	Japan: Kumamoto	soil	SAMD00534346	DRR403868	raw read data	N/A	This study	clade1-3	0.13	+	—
KHSU-234321-058	2021-03-22	Japan: Kumamoto	soil	SAMD00534347	DRR403869	raw read data	N/A	This study	clade1-1	0.50	+	—
KHSU-254321-059	2021-03-22	Japan: Kumamoto	soil	SAMD00534348	DRR403870	raw read data	N/A	This study	clade1-3	4.08	+	—
KHSU-134322-060	2021-03-22	Japan: Kumamoto	soil	SAMD00534349	DRR403871	raw read data	N/A	This study	clade1-1	0.50	+	—
KHSU-144322-061	2021-04-02	Japan: Kumamoto	soil	SAMD00534350	DRR403872	raw read data	N/A	This study	clade1-1	0.15	+	—
KHSU-154322-062	2021-03-22	Japan: Kumamoto	soil	SAMD00534351	DRR403873	raw read data	N/A	This study	clade1-3	0.79	+	—
KHSU-234322-063	2021-03-22	Japan: Kumamoto	soil	SAMD00534352	DRR403874	raw read data	N/A	This study	clade1-1	0.23	+	—
KHSU-244322-064	2021-04-02	Japan: Kumamoto	soil	SAMD00534353	DRR403875	raw read data	N/A	This study	clade1-1	0.25	+	—
KHSU-254322-065	2021-04-05	Japan: Kumamoto	soil	SAMD00534354	DRR403876	raw read data	N/A	This study	clade1-3	0.66	+	—
KHSU-134323-066	2021-03-22	Japan: Kumamoto	soil	SAMD00534355	DRR403877	raw read data	N/A	This study	clade1-3	4.70	+	—
KHSU-144323-067	2021-03-22	Japan: Kumamoto	soil	SAMD00534356	DRR403878	raw read data	N/A	This study	clade1-3	1.64	+	—
KHSU-154323-068	2021-03-22	Japan: Kumamoto	soil	SAMD00534357	DRR403879	raw read data	N/A	This study	clade1-3	0.61	+	—
KHSU-254323-069	2021-03-22	Japan: Kumamoto	soil	SAMD00534358	DRR403880	raw read data	N/A	This study	clade1-3	0.93	+	—
KHSU-154324-070	2021-09-28	Japan: Kumamoto	soil	SAMD00534359	DRR403881	raw read data	N/A	This study	clade1-2	0.00	—	—
KHSU-234324-071	2021-11-02	Japan: Kumamoto	soil	SAMD00534360	DRR403882	raw read data	N/A	This study	clade1-2	1.27	+	—
KHSU-234324-072	2021-11-02	Japan: Kumamoto	soil	SAMD00534361	DRR403883	raw read data	N/A	This study	clade1-2	1.55	+	—
KHSU-234324-073	2021-11-02	Japan: Kumamoto	soil	SAMD00534362	DRR403884	raw read data	N/A	This study	clade1-2	1.15	+	—
KHSU-234324-074	2021-11-02	Japan: Kumamoto	soil	SAMD00534363	DRR403885	raw read data	N/A	This study	clade1-2	0.70	+	—
KHSU-244324-075	2021-11-02	Japan: Kumamoto	soil	SAMD00534364	DRR403886	raw read data	N/A	This study	clade1-2	0.00	—	+
KHSU-244324-076	2021-11-02	Japan: Kumamoto	soil	SAMD00534365	DRR403887	raw read data	N/A	This study	clade1-2	0.00	—	—
KHSU-244324-077	2021-11-02	Japan: Kumamoto	soil	SAMD00534366	DRR403888	raw read data	N/A	This study	clade1-2	0.00	—	—
KHSU-244324-078	2021-11-02	Japan: Kumamoto	soil	SAMD00534367	DRR403889	raw read data	N/A	This study	clade1-2	0.00	—	—
KHSU-244324-079	2021-11-02	Japan: Kumamoto	soil	SAMD00534368	DRR403890	raw read data	N/A	This study	clade1-2	0.00	—	—
KHSU-244324-080	2021-11-02	Japan: Kumamoto	soil	SAMD00534369	DRR403891	raw read data	N/A	This study	clade1-3	1.59	+	—
KHSU-244324-081	2021-11-02	Japan: Kumamoto	soil	SAMD00534370	DRR403892	raw read data	N/A	This study	clade1-2	0.00	—	—
KHSU-244324-082	2021-11-02	Japan: Kumamoto	soil	SAMD00534371	DRR403893	raw read data	N/A	This study	clade1-2	0.00	—	—
KHSU-134325-083	2021-11-02	Japan: Kumamoto	soil	SAMD00534372	DRR403894	raw read data	N/A	This study	clade1-3	1.70	+	—
KHSU-134325-084	2021-11-02	Japan: Kumamoto	soil	SAMD00534373	DRR403895	raw read data	N/A	This study	clade1-2	1.02	+	—
KHSU-134325-085	2021-11-02	Japan: Kumamoto	soil	SAMD00534374	DRR403896	raw read data	N/A	This study	clade1-3	1.02	+	—
KHSU-134325-086	2021-11-02	Japan: Kumamoto	soil	SAMD00534375	DRR403897	raw read data	N/A	This study	clade1-3	1.47	+	—
KHSU-144325-087	2021-11-05	Japan: Kumamoto	soil	SAMD00534376	DRR403898	raw read data	N/A	This study	clade1-1	0.23	+	—
KHSU-144325-088	2021-11-05	Japan: Kumamoto	soil	SAMD00534377	DRR403899	raw read data	N/A	This study	clade1-1	0.29	+	—
KHSU-154325-089	2021-11-05	Japan: Kumamoto	soil	SAMD00534378	DRR403900	raw read data	N/A	This study	clade1-3	1.71	+	—
KHSU-154325-090	2021-11-05	Japan: Kumamoto	soil	SAMD00534379	DRR403901	raw read data	N/A	This study	clade1-3	1.46	+	—
KHSU-154325-091	2021-11-05	Japan: Kumamoto	soil	SAMD00534380	DRR403902	raw read data	N/A	This study	clade1-3	2.15	+	—
KHSU-234325-092	2021-11-08	Japan: Kumamoto	soil	SAMD00534381	DRR403903	raw read data	N/A	This study	clade1-1	0.01	+	—
KHSU-234325-093	2021-11-08	Japan: Kumamoto	soil	SAMD00534382	DRR403904	raw read data	N/A	This study	clade1-1	0.01	+	—
KHSU-234325-094	2021-11-08	Japan: Kumamoto	soil	SAMD00534383	DRR403905	raw read data	N/A	This study	clade1-1	0.01	+	—
KHSU-244325-095	2021-10-10	Japan: Kumamoto	soil	SAMD00534384	DRR403906	raw read data	N/A	This study	clade1-1	0.24	+	—
KHSU-254325-096	2021-10-03	Japan: Kumamoto	soil	SAMD00534385	DRR403907	raw read data	N/A	This study	clade1-2	0.00	—	—
KHSU-134326-097	2021-11-05	Japan: Kumamoto	soil	SAMD00534386	DRR403908	raw read data	N/A	This study	clade1-1	0.95	+	—
KHSU-134326-098	2021-11-05	Japan: Kumamoto	soil	SAMD00534387	DRR403909	raw read data	N/A	This study	clade1-2	0.88	+	—
KHSU-134326-099	2021-11-05	Japan: Kumamoto	soil	SAMD00534388	DRR403910	raw read data	N/A	This study	clade1-2	1.00	+	—
KHSU-134326-100	2021-11-05	Japan: Kumamoto	soil	SAMD00534389	DRR403911	raw read data	N/A	This study	clade1-2	1.15	+	—
KHSU-134326-101	2021-11-05	Japan: Kumamoto	soil	SAMD00534390	DRR403912	raw read data	N/A	This study	clade1-1	0.61	+	—
KHSU-134326-102	2021-11-05	Japan: Kumamoto	soil	SAMD00534391	DRR403913	raw read data	N/A	This study	clade1-1	0.50	+	—
KHSU-134326-103	2021-11-05	Japan: Kumamoto	soil	SAMD00534392	DRR403914	raw read data	N/A	This study	clade1-2	1.10	+	—
KHSU-134326-104	2021-11-05	Japan: Kumamoto	soil	SAMD00534393	DRR403915	raw read data	N/A	This study	clade1-2	0.93	+	—
KHSU-134326-105	2021-11-05	Japan: Kumamoto	soil	SAMD00534394	DRR403916	raw read data	N/A	This study	clade1-2	0.98	+	—
KHSU-134326-106	2021-11-05	Japan: Kumamoto	soil	SAMD00534395	DRR403917	raw read data	N/A	This study	clade1-2	1.52	+	—
KHSU-144326-107	2021-10-02	Japan: Kumamoto	soil	SAMD00534396	DRR403918	raw read data	N/A	This study	clade1-2	1.52	+	—
KHSU-244326-108	2021-11-05	Japan: Kumamoto	soil	SAMD00534397	DRR403919	raw read data	N/A	This study	clade1-1	0.42	+	—
KHSU-244326-109	2021-11-05	Japan: Kumamoto	soil	SAMD00534398	DRR403920	raw read data	N/A	This study	clade1-2	1.36	+	—
KHSU-244326-110	2021-11-05	Japan: Kumamoto	soil	SAMD00534399	DRR403921	raw read data	N/A	This study	clade1-2	1.95	+	—
KHSU-254326-111	2021-10-02	Japan: Kumamoto	soil	SAMD00534400	DRR403922	raw read data	N/A	This study	clade1-2	0.00	—	+
KHSU-254327-112	2021-11-08	Japan: Kumamoto	soil	SAMD00534401	DRR403923	raw read data	N/A	This study	clade1-2	0.19	+	—
KHSU-134328-113	2021-11-08	Japan: Kumamoto	soil	SAMD00534402	DRR403924	raw read data	N/A	This study	clade1-3	3.88	+	—
KHSU-234328-114	2021-11-08	Japan: Kumamoto	soil	SAMD00534403	DRR403925	raw read data	N/A	This study	clade1-2	0.16	+	—
KHSU-234328-115	2021-11-08	Japan: Kumamoto	soil	SAMD00534404	DRR403926	raw read data	N/A	This study	clade1-2	0.19	+	—
KHSU-254328-116	2021-11-08	Japan: Kumamoto	soil	SAMD00534405	DRR403927	raw read data	N/A	This study	clade1-3	1.48	+	—
KHSU-254328-117	2021-11-08	Japan: Kumamoto	soil	SAMD00534406	DRR403928	raw read data	N/A	This study	clade1-3	2.02	+	—
KHSU-254328-118	2021-11-08	Japan: Kumamoto	soil	SAMD00534407	DRR403929	raw read data	N/A	This study	clade1-3	2.44	+	—
KHSU-244329-119	2021-11-29	Japan: Kumamoto	soil	SAMD00534408	DRR403930	raw read data	N/A	This study	clade1-3	0.95	+	—
KHSU-134330-120	2021-11-22	Japan: Kumamoto	soil	SAMD00534409	DRR403931	raw read data	N/A	This study	clade1-2	0.15	+	+
KHSU-134330-121	2021-11-22	Japan: Kumamoto	soil	SAMD00534410	DRR403932	raw read data	N/A	This study	clade1-2	0.22	+	—
KHSU-134330-122	2021-11-22	Japan: Kumamoto	soil	SAMD00534411	DRR403933	raw read data	N/A	This study	clade1-3	1.18	+	—
KHSU-134330-123	2021-11-22	Japan: Kumamoto	soil	SAMD00534412	DRR403934	raw read data	N/A	This study	clade1-2	0.13	+	—
KHSU-134330-124	2021-11-22	Japan: Kumamoto	soil	SAMD00534413	DRR403935	raw read data	N/A	This study	clade1-2	0.13	+	—
KHSU-134330-125	2021-11-22	Japan: Kumamoto	soil	SAMD00534414	DRR403936	raw read data	N/A	This study	clade1-2	0.16	+	—
KHSU-134330-126	2021-11-22	Japan: Kumamoto	soil	SAMD00534415	DRR403937	raw read data	N/A	This study	clade1-2	0.12	+	—
KHSU-144330-127	2021-11-22	Japan: Kumamoto	soil	SAMD00534416	DRR403938	raw read data	N/A	This study	clade1-3	1.40	+	—
KHSU-144330-128	2021-11-22	Japan: Kumamoto	soil	SAMD00534417	DRR403939	raw read data	N/A	This study	clade1-3	1.58	+	—
KHSU-144330-129	2021-11-22	Japan: Kumamoto	soil	SAMD00534418	DRR403940	raw read data	N/A	This study	clade1-3	3.00	+	—
KHSU-144330-130	2021-11-22	Japan: Kumamoto	soil	SAMD00534419	DRR403941	raw read data	N/A	This study	clade1-3	1.52	+	—
KHSU-144330-131	2021-11-22	Japan: Kumamoto	soil	SAMD00534420	DRR403942	raw read data	N/A	This study	clade1-3	0.69	+	—
KHSU-144330-132	2021-11-22	Japan: Kumamoto	soil	SAMD00534421	DRR403943	raw read data	N/A	This study	clade1-2	0.85	+	—
KHSU-234330-133	2021-11-22	Japan: Kumamoto	soil	SAMD00534422	DRR403944	raw read data	N/A	This study	clade1-3	0.09	+	—
KHSU-244330-134	2021-11-22	Japan: Kumamoto	soil	SAMD00534423	DRR403945	raw read data	N/A	This study	clade1-1	0.04	+	—
KHSU-144331-135	2021-11-22	Japan: Kumamoto	soil	SAMD00534424	DRR403946	raw read data	N/A	This study	clade1-3	1.54	+	—
KHSU-234332-136	2021-11-29	Japan: Kumamoto	soil	SAMD00534425	DRR403947	raw read data	N/A	This study	clade1-3	0.29	+	—
KHSU-154333-137	2021-11-29	Japan: Kumamoto	soil	SAMD00534426	DRR403948	raw read data	N/A	This study	clade1-2	0.14	+	—
KHSU-254333-138	2021-11-29	Japan: Kumamoto	soil	SAMD00534427	DRR403949	raw read data	N/A	This study	clade1-2	0.03	+	—
KHSU-154310-139	2022-04-10	Japan: Kumamoto	soil	SAMD00534428	DRR403950	raw read data	N/A	This study	clade1-2	0.00	—	—
KHSU-154310-140	2022-04-10	Japan: Kumamoto	soil	SAMD00534429	DRR403951	raw read data	N/A	This study	clade1-2	0.00	—	—
KHSU-154310-141	2022-04-10	Japan: Kumamoto	soil	SAMD00534430	DRR403952	raw read data	N/A	This study	clade1-3	0.14	+	—
KHSU-154310-142	2022-04-10	Japan: Kumamoto	soil	SAMD00534431	DRR403953	raw read data	N/A	This study	clade2	0.00	—	—
KHSU-154310-143	2022-04-10	Japan: Kumamoto	soil	SAMD00534432	DRR403954	raw read data	N/A	This study	clade2	0.00	—	—
KHSU-154310-144	2022-04-10	Japan: Kumamoto	soil	SAMD00534433	DRR403955	raw read data	N/A	This study	clade2	0.00	—	—
KHSU-154310-145	2022-04-10	Japan: Kumamoto	soil	SAMD00534434	DRR403956	raw read data	N/A	This study	clade1-2	0.00	—	—

KHSU-154310-146	2022-04-10	Japan: Kumamoto	soil	SAMD00534435	DRR403957	raw read data	N/A	This study	clade1-3	0.20	+	—
KHSU-154310-147	2022-04-10	Japan: Kumamoto	soil	SAMD00534436	DRR403958	raw read data	N/A	This study	clade1-2	0.00	—	—
KHSU-154310-148	2022-04-10	Japan: Kumamoto	soil	SAMD00534437	DRR403959	raw read data	N/A	This study	clade2	0.00	—	—
KHSU-154310-149	2022-04-10	Japan: Kumamoto	soil	SAMD00534438	DRR403960	raw read data	N/A	This study	clade1-2	0.00	—	—
KHSU-154310-150	2022-04-10	Japan: Kumamoto	soil	SAMD00534439	DRR403961	raw read data	N/A	This study	clade1-2	0.00	—	—
KHSU-154310-151	2022-04-10	Japan: Kumamoto	soil	SAMD00534440	DRR403962	raw read data	N/A	This study	clade1-3	0.26	+	—
KHSU-074300-152	N/A	Japan: Kumamoto	Homo sapiens	SAMD00534441	DRR403963	raw read data	N/A	This study	clade1-3	0.78	+	—
NIID-071400-001	2013	Japan: Kanagawa	Homo sapiens	SAMD00534442	DRR403964, DRR404005	complete	AP026823-AP026824 (INSD ID)	This study	clade1-2	0.33	+	+
NIID-072000-002	2009	Japan: Nagano	Homo sapiens	SAMD00534443	DRR403965, DRR404006	complete	AP026825-AP026826 (INSD ID)	This study	clade1-1	0.17	+	—
NIID-082701-003	2008	Japan: Osaka	yellow sand	SAMD00534444	DRR403966	raw read data	N/A	This study	clade1-3	4.45	+	—
NIID-071100-006	N/A	Japan: Saitama	Homo sapiens	SAMD00534445	DRR403967	raw read data	N/A	This study	clade1-3	3.89	+	—
NIID-07XX00-007	N/A	Japan	N/A	SAMD00534446	DRR403968	raw read data	N/A	This study	clade1-3	1.70	+	—
NIID-070700-008	2008	Japan: Fukushima	Homo sapiens	SAMD00534447	DRR403969	raw read data	N/A	This study	clade2	0.21	+	—
NIID-070700-009	2009	Japan: Fukushima	Homo sapiens	SAMD00534448	DRR403970	raw read data	N/A	This study	clade1-1	0.23	+	—
NIID-091100-010	2010	Japan: Saitama	Macaca fuscata	SAMD00534449	DRR403971	raw read data	N/A	This study	clade1-1	0.46	+	—
NIID-071300-011	2006	Japan: Tokyo	Homo sapiens	SAMD00534450	DRR403972	raw read data	N/A	This study	clade1-2	1.06	+	—
NIID-070300-012	2010	Japan: Iwate	Homo sapiens	SAMD00534451	DRR403973	raw read data	N/A	This study	clade1-3	1.88	+	—
NIID-074600-013	2012	Japan: Kagoshima	Homo sapiens	SAMD00534452	DRR403974	raw read data	N/A	This study	clade1-3	3.41	+	—
NIID-XXAA02-014	N/A	Mongolia	soil	SAMD00534453	DRR403975	raw read data	N/A	This study	clade1-3	4.20	+	—
NIID-072500-015	2008	Japan: Shiga	Homo sapiens	SAMD00534454	DRR403976	raw read data	N/A	This study	clade1-3	4.26	+	—
NIID-073300-016	2008	Japan: Okayama	Homo sapiens	SAMD00534455	DRR403977	raw read data	N/A	This study	clade1-3	4.12	+	—
NIID-07AA00-017	N/A	Pakistan	Homo sapiens	SAMD00534456	DRR403978	raw read data	N/A	This study	clade1-2	0.60	+	—
NIID-073200-018	2014	Japan: Shimane	Homo sapiens	SAMD00534457	DRR403979	raw read data	N/A	This study	clade1-3	1.85	+	—
NIID-072200-019	2008	Japan: Shizuoka	Homo sapiens	SAMD00534458	DRR403980	raw read data	N/A	This study	clade1-3	5.68	+	—
NIID-230303-020	2007	Japan: Iwate	soil	SAMD00534459	DRR403981	raw read data	N/A	This study	clade1-1	0.66	+	—
NIID-230804-021	2007	Japan: Ibaraki	soil	SAMD00534460	DRR403982	raw read data	N/A	This study	clade1-3	0.36	+	—
NIID-234605-022	2010	Japan: Kagoshima	soil	SAMD00534461	DRR403983	raw read data	N/A	This study	clade1-3	0.86	+	—
NIID-261306-023	2007	Japan: Tokyo	soil	SAMD00534462	DRR403984	raw read data	N/A	This study	clade1-3	0.39	+	—
NIID-242307-024	2007	Japan: Aichi	soil	SAMD00534463	DRR403985	raw read data	N/A	This study	clade1-2	0.41	+	—
NIID-230108-025	2009	Japan: Hokkaido	soil	SAMD00534464	DRR403986	raw read data	N/A	This study	clade1-3	0.57	+	—
NIID-241109-026	2008	Japan: Saitama	soil	SAMD00534465	DRR403987	raw read data	N/A	This study	clade1-3	7.32	+	—
NIID-233310-027	2007	Japan: Okayama	soil	SAMD00534466	DRR403988	raw read data	N/A	This study	clade1-1	0.19	+	—
NIID-232711-028	2007	Japan: Osaka	soil	SAMD00534467	DRR403989	raw read data	N/A	This study	clade1-3	0.23	+	—
NIID-243012-029	2007	Japan: Wakayama	soil	SAMD00534468	DRR403990	raw read data	N/A	This study	clade1-1	0.69	+	—
NIID-071300-030	2007	Japan: Tokyo	Homo sapiens	SAMD00534469	DRR403991	raw read data	N/A	This study	clade1-3	1.90	+	—
NIID-071400-031	2002	Japan: Kanagawa	Homo sapiens	SAMD00534470	DRR403992	raw read data	N/A	This study	clade1-3	1.02	+	—
NIID-071400-032	2012	Japan: Kanagawa	Homo sapiens	SAMD00534471	DRR403993	raw read data	N/A	This study	clade1-3	4.89	+	—
NIID-071900-033	N/A	Japan: Yamanashi	Homo sapiens	SAMD00534472	DRR403994	raw read data	N/A	This study	clade1-3	0.69	+	—
ATCC 453	N/A	China: Beijing	Homo sapiens	SAMD00534473	DRR403995	raw read data	N/A	This study	clade1-2	0.20	+	—
ATCC 9441	N/A	USA	N/A	SAMD00534474	DRR403996	raw read data	N/A	This study	clade1-2	0.92	+	—
63.05	2005	France	Homo sapiens	SAMN09469719	N/A	draft	GCA_004115605.1 (Assembly ID)	Download data	clade2	N/A	+	—
75.97	1997	France	N/A	SAMN09469717	N/A	draft	GCA_004115905.1 (Assembly ID)	Download data	clade1-2	N/A	+	—
89.12	2012	France	Homo sapiens	SAMN09469721	N/A	draft	GCA_004115505.1 (Assembly ID)	Download data	clade1-2	N/A	+	—
157.15	2014	France	Homo sapiens	SAMN09469722	N/A	draft	GCA_004119215.1 (Assembly ID)	Download data	clade1-2	N/A	—	—
184.08	2008	France: Toulouse	Homo sapiens	SAMN03145366	N/A	draft	GCA_000805775.1 (Assembly ID)	Download data	clade2	N/A	+	—
202.15	2015	France	Homo sapiens	SAMN09469723	N/A	draft	GCA_004115565.1 (Assembly ID)	Download data	clade1-2	N/A	+	—
329	1965	USSR	N/A	SAMN09469707	N/A	draft	GCA_004168545.1 (Assembly ID)	Download data	clade1-2	N/A	—	—
358.99	1999	France	N/A	SAMN09469718	N/A	draft	GCA_004115485.1 (Assembly ID)	Download data	clade1-2	N/A	+	—
407.86	1986	France	N/A	SAMN09469716	N/A	draft	GCA_004115585.1 (Assembly ID)	Download data	clade1-2	N/A	—	—
641.84	1984	France	N/A	SAMN09469715	N/A	draft	GCA_004115655.1 (Assembly ID)	Download data	clade1-2	N/A	+	—
778.17	2017	France	Homo sapiens	SAMN09469725	N/A	draft	GCA_004115665.1 (Assembly ID)	Download data	clade2	N/A	+	—
1240	1955	France	Felis catus	SAMN09469706	N/A	draft	GCA_004119335.1 (Assembly ID)	Download data	clade1-2	N/A	+	—
1337	1955	France	N/A	SAMN09469726	N/A	draft	GCA_004115515.1 (Assembly ID)	Download data	clade1-2	N/A	+	—
2017.061	2016	France	Homo sapiens	SAMN09469724	N/A	draft	GCA_004115495.1 (Assembly ID)	Download data	clade1-1	N/A	+	—
3483	1964	France	N/A	SAMN09469714	N/A	draft	GCA_004115705.1 (Assembly ID)	Download data	clade2	N/A	+	—
3582	1964	France	Homo sapiens	SAMN09469712	N/A	draft	GCA_004115575.1 (Assembly ID)	Download data	clade1-2	N/A	+	—
12124569	N/A	N/A	N/A	SAMEA3283140	N/A	complete	GCA_000967115.1 (Assembly ID)	Download data	clade2	N/A	+	—
132CV	1955	Germany	N/A	SAMN09469713	N/A	draft	GCA_004115645.1 (Assembly ID)	Download data	clade1-3	N/A	+	—
1586-U1	1969	France	N/A	SAMN09469704	N/A	draft	GCA_004119195.1 (Assembly ID)	Download data	clade1-2	N/A	+	—
1586-Z1	1969	France	N/A	SAMN09469705	N/A	draft	GCA_004119345.1 (Assembly ID)	Download data	clade1-2	N/A	—	—
46.1.08	2008	France	N/A	SAMN09469720	N/A	draft	GCA_004119225.1 (Assembly ID)	Download data	clade1-2	N/A	+	—
4784A	1968	N/A	N/A	SAMN09469703	N/A	draft	GCA_004119235.1 (Assembly ID)	Download data	clade1-2	N/A	+	—
512-15	1955	Viet Nam	cheese	SAMN09469708	N/A	draft	GCA_004119405.1 (Assembly ID)	Download data	clade1-2	N/A	+	—
ATCC 19406	1920	United Kingdom: London	N/A	SAMN02745112	N/A	draft	GCA_900167265.1 (Assembly ID)	Download data	clade1-2	N/A	—	—
ATCC 454	N/A	China; Beijing	feces	SAMN03579569	N/A	draft	GCA_000987095.1 (Assembly ID)	Download data	clade1-2	N/A	—	—
B4	1962	N/A	N/A	SAMN09469709	N/A	draft	GCA_004119395.1 (Assembly ID)	Download data	clade1-2	N/A	+	—
CN655	1929	France: Paris	Homo sapiens	SAMN03145081	N/A	draft	GCA_000805755.1 (Assembly ID)	Download data	clade1-2	N/A	+	—
COR1	1955	France	Homo sapiens	SAMN09469710	N/A	draft	GCA_004119295.1 (Assembly ID)	Download data	clade1-2	N/A	—	—
Massachusetts substr. E88	N/A	N/A	N/A	SAMN02603289	N/A	complete	GCA_000007625.1 (Assembly ID)	Download data	clade1-2	N/A	+	—
Harvard	1949	N/A	N/A	SAMN09469701	N/A	draft	GCA_004119355.1 (Assembly ID)	Download data	clade1-2	N/A	+	—
Mfbjulcb2	2004-10	India: Kerala, Cochin	retail fish market	SAMN08398331	N/A	complete	GCA_003013635.1 (Assembly ID)	Download data	clade1-3	N/A	+	—
MGYG-HGUT-02375	N/A	China	Homo sapiens	SAMEA5851879	N/A	draft	GCA_902386545.1 (Assembly ID)	Download data	clade1-2	N/A	+	—
NCTC539	1917	N/A	Homo sapiens	SAMEA4556074	ERR2125619	draft	GCA_900461425.1 (Assembly ID)	Download data	clade1-3	N/A	+	—
NCTC540	1917	United Kingdom: London	N/A	SAMEA37375918	ERR2125692	draft	GCA_900447145.1 (Assembly ID)	Download data	clade1-2	N/A	+	—
Strain 3	1955	Denmark	N/A	SAMN09469702	N/A	draft	GCA_004119205.1 (Assembly ID)	Download data	clade1-2	N/A	+	—
TMB2	1956	France	N/A	SAMN09469711	N/A	draft	GCA_004119305.1 (Assembly ID)	Download data	clade1-2	N/A	+	—

N/A, not available; INSD, the International Nucleotide Sequence Databases; +, positive; —, negative
