# Supplementary material for: Comparative pathogenomic analysis reveals a highly tetanus toxin-producing clade of Clostridium tetani isolates in Japan
Source: mSphere. 2023 Nov 27;8(6):e00369-23. doi: 10.1128/msphere.00369-23 (PMC10732020; doi:10.1128/msphere.00369-23)
Supplement: Table S2 — TPM gene expression determined by RNA-Seq analysis. [file msphere.00369-23-s0002.pdf]

Table S2. TPM gene expression determined by RNA-Seq analysis

	Strain	KHSU-134323-066			KHSU-134328-113			KHSU-074300-152			KHSU-234315-040			KHSU-154301-001			KHSU-144303-003			KHSU-154306-013			KHSU-144305-010			KHSU-144313-037			KHSU-244326-108		
	Cultivation (hour)	8h	24h	48h	8h	24h	48h	8h	24h	48h	8h	24h	48h	8h	24h	48h	8h	24h	48h	8h	24h	48h	8h	24h	48h	8h	24h	48h	8h	24h	48h
Gene ID	Region	TPM	TPM	TPM	TPM	TPM	TPM	TPM	TPM	TPM	TPM	TPM	TPM	TPM	TPM	TPM	TPM	TPM	TPM	TPM	TPM	TPM	TPM	TPM	TPM	TPM	TPM	TPM	TPM	TPM	TPM
CTC_RS00005	complement(1..2304)	35	113	69	109	139	105	57	171	70	223	98	155	72	89	118	77	88	115	94	84	104	48	113	106	48	107	101	104	75	175
cls	2493..3923	28	50	28	21	32	36	26	11	19	50	45	0	20	10	29	43	18	44	33	0	84	35	52	85	33	16	0	14	0	94
CTC_RS00015	3946..4548	22	39	0	76	190	143	62	78	89	29	53	79	36	0	0	68	126	42	28	0	119	62	62	0	26	37	77	23	41	32
CTC_RS14070	complement(4584..5102)	690	503	1204	706	662	1295	1204	1122	926	945	805	1104	654	846	1273	1273	729	1122	946	839	876	908	1294	1098	871	775	960	609	615	591
CTC_RS00045	10753..11397	0	0	0	0	0	0	0	0	0	18	25	0	0	0	0	0	0	0	7	0	0	0	0	0	0	0	0	0	0	0
CTC_RS00050	complement(11758..11946)	0	0	0	0	0	0	0	0	0	31	85	0	0	0	0	0	133	0	0	0	0	0	0	0	0	59	0	0	0	0
CTC_RS00055	complement(11960..12148)	0	0	0	0	0	0	0	0	0	31	0	0	0	0	0	0	0	0	0	0	0	0	0	0	0	0	0	0	0	0
CTC_RS14840	complement(12244..12399)	0	0	0	0	0	0	0	0	0	0	0	0	0	0	0	87	0	0	0	0	0	0	0	0	0	0	0	0	0	0
CTC_RS00065	complement(12432..13208)	0	31	0	10	0	0	12	0	0	23	0	61	0	0	0	0	65	81	5	0	31	32	48	0	0	29	30	18	0	0
CTC_RS00070	13557..14816	21	19	0	36	36	14	15	0	21	103	0	0	29	23	17	32	0	30	34	0	57	39	0	32	13	0	0	68	0	46
CTC_RS00075	complement(14871..15398)	230	90	50	87	43	261	88	119	101	112	152	136	191	0	119	77	1051	1055	201	641	317	70	212	771	90	148	220	104	93	182
sfsA	complement(15446..16138)	20	34	38	55	0	75	121	136	39	128	0	0	125	84	30	138	328	694	141	0	242	36	108	176	137	32	100	119	0	83
dpaL	complement(16325..17551)	0	0	22	6	0	0	0	0	0	14	79	97	0	0	0	67	617	712	3	0	0	0	30	33	0	0	19	17	20	31
CTC_RS00090	complement(17824..19581)	46	67	136	78	52	196	53	98	30	59	160	68	57	42	24	62	115	130	82	0	177	7	42	104	47	57	53	94	56	109
CTC_RS00095	complement(19603..19863)	104	0	0	117	88	66	36	0	0	45	185	91	111	168	0	209	0	49	212	185	0	48	0	156	91	86	89	26	94	73
CTC_RS00100	complement(19909..20178)	0	0	0	0	0	0	0	0	0	22	0	0	0	54	0	0	0	0	0	0	0	0	0	0	0	0	0	0	0	0
recR	complement(20790..21386)	985	2344	2461	1240	1229	1011	859	2900	1700	841	2019	1199	719	3457	1932	707	4057	2407	1243	810	1082	1018	1750	1023	717	3848	1495	921	3988	1991
CTC_RS00110	complement(21414..21755)	711	1040	2041	1071	1542	1008	600	1749	859	1036	1222	1047	433	2140	1349	896	5016	2721	1150	1273	769	798	764	1428	533	2386	1017	764	4665	2018
dnaX	complement(21830..23479)	74	86	121	150	222	230	79	105	81	322	88	152	44	98	25	116	107	269	142	117	333	23	90	123	34	115	84	177	119	319
CTC_RS00120	complement(23508..23711)	66	0	0	0	0	0	0	0	131	58	0	0	0	0	0	0	0	0	21	0	0	122	0	0	0	55	0	67	0	94
CTC_RS00130	complement(24390..24629)	225	1186	1385	445	1815	1005	39	1312	779	738	1339	2088	211	1098	524	369	526	686	230	1411	1595	207	622	848	231	605	580	286	716	2396
CTC_RS00135	complement(24825..25391)	48	42	0	13	0	30	0	28	0	31	0	0	25	52	74	12	0	22	30	0	84	0	66	0	0	0	0	12	0	0
CTC_RS00145	complement(25611..26051)	0	0	30	17	0	156	0	0	0	27	0	162	0	33	95	0	57	86	10	0	54	0	0	0	0	25	53	0	0	130
CTC_RS00155	complement(26462..27028)	72	0	70	94	121	61	82	83	47	135	85	84	64	26	0	36	89	45	82	0	42	44	66	0	42	39	82	73	43	68
CTC_RS00160	27169..28062	0	0	15	43	0	77	0	0	30	33	36	0	8	0	23	0	0	14	5	0	27	0	0	91	0	13	0	0	0	0
serS	complement(28683..29963)	369	222	156	494	161	175	564	246	83	454	213	466	501	274	65	521	118	257	541	340	355	581	175	318	415	297	181	392	211	135
CTC_RS00185	complement(30264..30641)	0	0	141	121	243	228	74	125	71	62	170	63	19	194	111	36	67	67	79	128	127	131	99	108	84	59	184	18	65	51
CTC_RS00190	complement(30658..32043)	29	68	86	96	149	149	54	170	58	102	104	258	57	116	76	59	127	146	141	0	242	63	135	88	46	89	84	69	106	97
CTC_RS00195	complement(32094..34310)	12	21	12	24	93	54	25	28	24	36	36	32	33	20	0	25	0	57	36	0	65	28	34	18	14	35	10	16	33	9
CTC_RS00200	complement(34405..37068)	5	27	25	30	17	32	11	18	10	22	72	36	5	38	8	5	19	14	18	18	36	0	14	15	6	29	9	5	9	22
CTC_RS00205	complement(37111..37623)	53	46	26	15	89	34	18	92	0	23	31	93	28	114	0	80	0	49	33	94	93	121	146	79	0	44	45	0	0	37
CTC_RS00210	complement(37641..38144)	0	0	0	0	0	34	19	0	0	12	0	0	0	0	0	0	100	0	8	0	95	49	0	81	0	0	0	14	0	38
gyrA	complement(43745..46177)	300	341	273	365	358	276	303	259	220	477	175	363	352	271	211	482	363	401	422	249	315	306	153	151	339	317	191	305	192	378
gyrB	complement(46198..48111)	268	174	125	273	323	207	324	222	126	413	84	299	294	283	142	356	145	318	413	227	175	194	117	149	282	274	133	302	180	130
CTC_RS00250	complement(48131..48394)	307	90	201	318	0	65	177	119	202	425	61	271	219	0	318	310	0	480	354	0	544	141	0	0	300	339	0	234	0	363
recF	complement(48425..49528)	61	64	72	83	83	94	68	114	24	404	44	86	52	53	95	136	23	103	112	0	65	11	68	111	65	71	95	149	44	208
yaaA	complement(49641..49847)	0	0	64	0	0	0	45	0	0	0	0	0	0	0	0	0	0	0	21	0	0	0	0	0	0	0	0	0	0	0
dnaN	complement(49861..50964)	12	43	24	28	104	125	93	43	24	118	87	86	78	66	38	148	23	115	85	44	87	45	34	111	65	51	63	112	0	122
dnaA	complement(51225..52589)	59	70	49	50	84	139	34	46	0	225	47	17	26	43	15	42	18	83	44	71	70	9	82	60	23	8	34	45	54	70
rpmH	53086..53220	200	176	99	170	170	0	138	233	0	87	0	177	160	108	0	404	0	94	220	0	0	460	276	0	0	166	0	153	0	142
rnpA	53277..53612	161	212	79	363	205	308	222	234	79	580	239	284	279	349	125	466	75	188	456	576	498	258	0	363	130	100	276	389	0	57
yidD	53609..53818	0	0	63	109	0	0	0	0	0	197	0	0	0	0	0	0	0	60	0	0	0	0	178	0	0	53	0	98	0	0
CTC_RS00290	53836..54498	408	394	201	460	380	1053	563	214	161	1514	267	648	272	177	221	390	266	535	879	584	974	262	450	583	108	219	210	912	296	405
CTC_RS00295	54512..55138	323	719	297	609	402	687	521	427	128	876	538	609	311	444	201	440	402	565	1173	386	458	218	417	584	266	437	555	718	548	397
mnmE	55193..56572	10	17	29	39	50	69	68	11	0	56	12	104	21	21	30	25	37	101	68	70	17	18	27	30	40	16	17	40	18	42
mnmG	56582..58453	79	63	57	82	37	101	85	34	29	91	120	153	108	31	45	84	81	81	143	78	89	46	100	109	76	78	37	59	131	92
rsmG	58475..59194	0	99	55	53	64	48	52	0	0	115	67	99	10	0	29	47	0	0	77	134	0	17	52	226	77	0	0	76	68	53
noc	59309..60088	277	258	239	294	411	442	359	343	411	220	433	490	134	150	188	210	194	390	84	248	399	143	287	104	112	150	149	141	79	467
CTC_RS00320	60188..60970	52	242	204	102	117	572	155	60	0	98	390	335	92	56	107	96	64	146	81	185	153	111	477	468	51	100	148	61	63	147
CTC_RS00325	60963..61820	32	111	201	125	374	623	163	110	125	83	543	501	118	34	73	143	265	236	159	226	279	58	304	332	65	65	135	56	57	223
CTC_RS00330	61860..62357	27	143	134	61	92	588	19	0	161	12	129	240	0	59	84	27	0	25	43	97	288	75	150	327	16	45	0	69	0	0
yyaC_1	complement(62379..62954)	0	0	46	0	0	0	0	0	0	0	112	0	0	76	36	0	44	0	0	0	0	0	0	0	0	39	0	12	0	33
ytvI	complement(63010..64065)	0	0	13	0	22	0	0	0	51	17	0	0	0	0	0	0	24	0	2	0	23	0	0	0	0	0	44	20	0	18
CTC_RS00345	64302..65186	15	0	15	26	0	0	21	18	0	40	91	108	41	17	0	38	0	0	10	55	0	14	42	46	27	0	0	16	0	22
CTC_RS00350	65179..65379	0	0	0	38	0	0	0	0	0	0	0	0	0	0	0	0	0	0	21	0	0	0	0	0	0	0	0	0	0	0
rpsF	65477..65761	2418	1997	1773	4445	3378	3084	3011	2596	2062	11201	2538	3099	3546	3339	3606	4756	797	4087	7526	2885	2686	4005	1833	3857	3033	3334	2522	15498	2670	2892
CTC_RS00360	65777..66214	2376	2437	1397	2997	2303	1220	2311	2084	1586	5198	1872	2234	3049	2340	2251	3172	749	1908	6577	1436	1802	3258	2343	2556	2100	2654	2435	9331	1177	1685
rpsR	66235..66501	962	888	647	1229	687	839	1118	1120	1100	2898	542	1252	1811	1371	1375	1403	189	1185	2309	544	1254	1301	978	1067	1634	1005	1737	3978	552	862
CTC_RS00370	66688..67005	85	298	209	120	216	217	293	198	0	409	202	75	159	92	264	214	0	119	267	152	602	117	0	0	150	88	0	303	309	241
CTC_RS00375	67019..68008	0	48	54	15	69	70	19	64	0	119	65	48	44	30	21	34	0	77	26	49	145	13	75	0	16	62	23	49	50	97
CTC_RS00380	68021..70015	68	65	87	88	92	156	84	55	67	105	73	96	51	66	42	125	38	184	140	97	120	62	112	102	32	81	12	72	98	173
rplI	70012..70455	304	160	30	395	258	78	420	106	180	213	145	54	455	33	236	460	256	342	326	218	377	391	168	183	321	101	52	217	111	259
lonC	70465..72360	285	263	238	441	435	532	347	332	141	422	280	579	343	259	166	568	612	594	410	434	517	425	236	301	272	248	122	285	272	576
CTC_RS00395	72416..73750	304	426	329	389	378	620	356	271	200	429	349	715	168	389	204	253	340	398	490	507	600	270	140	213	220	209	139	252	221	287
CTC_RS14075	73872..75389	151	594	456	176	687	341	132	643	633	206	572	660	171	463	491	184	316	363	262	510	441	192	639	268	167	530	473	263	340	1339
CTC_RS00405	75563..76000	0	54	61	0	105	79	21	36	61	108	37	54	0	33	48	31	58	87	87	0	164	0	0	186	0	153	0	47	112	88
CTC_RS00410	76027..76242	0	0	0	35	106	0	0	219	0	0	0	0	0	136	194	63	117	176	98	0	0	0	0	0	0	52	107	95	0	0
pgsA_1	complement(76265..76795)	0	0	50	14	0	32	18	0	0	44	0	0	14	28	0	0	0	0	8	0	0	0	0	0	0	0	0	0	0	0
CTC_RS00420	76996..78405	0	67	113	0	81	73	20	179	0	17	182	34	10	270	30	14	233	90	18	0	68	18	79	29	6	277	115	5	296	95
CTC_RS00425	78546..78728	591	259	509	167	125	283	153	172	584	387	351	522	0	80	115	112	0	277	139	0	131	271	612	445	130	244	0	113	402	105
CTC_RS00430	78851..79600	18	127	35	20	0	46	75	42	107	79	150	32	48	20	140	27	0	51	34	64	0	33	0	0	42	45	31	9	33	77
CTC_RS00435	complement(79615..80838)	0	0	11	25	56	84	8	0	44	152	13	0	18	12	0	28	0	21	24	40	59	10	61	67	13	27	0	79	40	63
CTC_RS00440	80972..81355	0	124	35	20	60	0	73	123	0	46	0	0	38	267	137	124	66	66	111	126	0	0	97	0	0	116	0	18	192	50
CTC_RS00445	81362..82177	99	160	81	94	169	42	183	212	65	123	177	0	159	126	103	184	62	264	146	0	117	76	0	150	136	205	114	51	241	82

CTC_RS00470	complement(82777..83766)	27	0	40	31	23	52	28	32	54	179	16	0	29	15	0	41	25	90	13	98	121	25	0	82	0	0	0	42	74	116
CTC_RS00475	complement(83763..84737)	55	0	68	39	47	35	38	48	27	176	66	122	30	45	43	35	0	39	39	99	0	38	115	0	33	23	24	95	101	98
CTC_RS00480	84888..85955	158	366	62	300	472	145	358	177	75	2171	181	89	257	233	98	953	71	605	1421	226	269	221	315	191	219	335	109	1101	253	413
CTC_RS00485	85948..86403	193	364	87	184	553	189	389	173	176	2921	211	0	158	96	46	597	55	389	951	212	472	82	655	536	122	123	305	1417	54	736
CTC_RS00490	86418..87422	350	425	225	706	547	326	571	681	478	4093	464	166	513	364	250	1095	126	731	1582	554	500	562	520	486	458	578	277	1669	317	744
CTC_RS00495	87442..87666	300	633	0	746	815	230	539	944	831	1969	1572	106	738	911	559	2210	449	450	2419	860	319	607	829	181	846	944	206	1619	218	256
fabK	87677..88618	402	806	254	725	803	238	555	902	510	1966	529	152	881	870	557	2563	348	1089	2959	719	813	790	792	281	775	908	517	1635	443	610
fabD	88611..89549	144	202	142	378	366	110	209	319	228	1516	377	76	430	218	201	1454	242	701	1404	824	560	410	159	260	253	345	74	1281	627	347
fabG	89557..90312	420	439	281	666	576	296	469	521	353	3168	361	126	678	349	305	1423	167	888	1347	640	918	739	741	215	698	621	276	1405	487	558
fabF	90342..91577	503	480	97	676	760	139	483	509	194	2083	403	77	911	681	272	1824	265	656	1772	861	474	753	453	527	1011	515	356	1299	377	434
accB	91580..92038	265	52	29	266	200	113	81	103	116	843	140	52	362	255	46	772	55	290	690	316	52	243	81	89	207	195	202	674	160	167
fabZ	92073..92501	425	221	46	409	427	121	391	220	187	1852	300	56	530	341	196	1032	118	295	852	226	112	434	87	0	555	208	108	897	114	402
CTC_RS00530	92509..93849	302	124	79	450	205	180	146	200	80	1513	198	53	606	246	141	1079	94	453	996	433	285	555	223	182	553	233	138	1115	201	214
CTC_RS00535	93861..95588	133	233	46	265	239	60	184	109	62	1123	121	14	294	152	158	493	73	234	674	112	152	280	173	71	317	210	107	557	170	122
CTC_RS00545	95696..97032	0	0	0	0	0	0	0	0	0	0	0	0	0	0	0	0	0	0	0	0	0	0	0	0	0	0	0	0	0	0
CTC_RS00550	complement(97100..97609)	3498	884	1486	1347	899	2433	2762	895	1047	1181	2017	2200	1472	832	1069	1462	2671	1192	1722	1423	3049	1606	1317	2954	1415	942	1000	1159	1781	1203
CTC_RS00555	complement(97682..98875)	0	40	50	0	19	14	0	26	0	10	135	20	0	123	53	0	21	53	7	41	0	0	0	0	0	66	39	0	62	48
CTC_RS00560	complement(98954..99388)	0	109	0	0	0	40	0	36	61	0	74	0	0	0	0	0	232	0	29	0	0	0	86	0	0	103	0	0	0	44
CTC_RS00565	complement(99484..99975)	714	241	419	768	606	806	578	320	271	1038	98	437	337	119	85	471	205	888	302	0	146	706	797	786	290	295	283	314	349	662
CTC_RS00570	100302..100904	0	39	0	0	0	29	0	0	0	10	0	0	0	0	0	0	0	0	0	0	0	0	0	0	13	0	0	11	0	0
asnS	complement(100960..102351)	476	204	191	315	313	272	466	294	96	549	104	274	347	116	60	313	54	164	293	243	327	446	375	205	376	120	133	242	106	193
CTC_RS00580	102883..103350	87	51	114	82	0	184	100	101	57	215	0	204	46	188	45	15	162	352	91	0	51	0	239	261	34	96	149	59	367	205
CTC_RS00585	103488..104177	20	0	39	0	83	125	27	0	77	60	70	0	0	21	0	10	37	37	12	0	69	18	162	59	0	0	34	10	0	28
CTC_RS00590	104193..104747	0	43	24	14	0	62	17	0	0	21	87	43	0	79	38	12	0	23	8	0	43	0	0	73	0	40	0	12	44	86
surE	complement(104842..105591)	18	0	0	20	0	46	25	63	0	118	0	0	0	39	56	9	34	68	23	0	64	50	0	54	21	15	31	55	65	51
CTC_RS00600	complement(105626..106525)	0	26	15	25	0	19	0	17	30	105	36	27	0	0	23	68	28	42	38	0	53	14	41	0	9	25	0	53	27	43
msrA	106685..107161	0	0	0	16	0	0	0	0	0	0	34	100	15	31	88	14	0	0	53	0	100	0	0	0	0	23	0	43	154	0
CTC_RS00610	complement(107204..108037)	0	0	0	0	0	0	0	0	0	7	19	0	0	0	0	4	30	15	0	0	0	0	0	0	0	0	0	0	0	0
bioB	complement(108080..109057)	0	0	68	23	0	70	0	16	0	48	131	49	0	15	0	28	0	13	22	0	0	13	76	42	0	23	24	7	50	39
CTC_RS00620	109200..110387	0	120	0	13	0	15	39	13	22	50	27	20	24	0	53	34	0	32	43	41	20	31	94	0	7	28	0	35	21	81
CTC_RS00625	complement(110388..111269)	15	27	0	0	0	20	0	0	0	0	0	54	8	0	0	0	0	57	0	0	0	0	0	0	0	0	0	0	0	0
CTC_RS00630	111391..112581	306	139	134	429	173	232	995	119	179	655	94	140	115	111	194	858	233	420	571	41	382	198	94	308	186	89	175	346	165	386
CTC_RS00635	complement(112660..113367)	0	67	56	43	32	146	26	133	38	83	45	0	0	41	30	19	143	54	18	205	68	0	53	115	34	16	33	136	35	108
CTC_RS00640	complement(113407..113967)	0	42	0	0	0	0	0	0	0	0	0	0	0	0	0	0	0	0	0	0	0	0	0	0	0	0	0	0	0	0
CTC_RS00645	complement(113969..114856)	0	27	0	17	26	78	0	0	0	80	18	0	0	0	0	15	28	14	5	0	0	0	0	0	0	0	0	15	0	0
CTC_RS14630	complement(114869..115027)	0	0	0	0	0	0	0	0	0	0	0	0	0	0	0	0	0	0	0	0	0	0	0	0	0	0	0	0	0	0
CTC_RS00650	115152..115748	0	795	223	13	346	87	16	712	537	0	633	80	0	2011	386	0	507	127	0	81	0	21	438	136	0	1638	272	0	925	161
CTC_RS00655	complement(115815..116735)	0	52	29	0	0	56	10	0	29	6	52	0	0	0	0	0	0	0	0	0	0	13	0	44	0	0	0	7	0	0
CTC_RS00660	complement(116754..117509)	0	0	0	0	0	23	0	0	0	0	21	0	0	0	0	9	67	33	0	0	0	0	49	0	0	0	0	18	0	25
CTC_RS00665	complement(117502..118425)	15	26	43	41	25	19	50	51	0	13	17	13	8	0	0	7	0	0	14	26	0	0	0	0	26	12	0	15	0	21
CTC_RS00670	complement(118508..119209)	19	34	76	43	65	147	66	0	76	42	46	0	31	0	60	29	0	18	24	0	34	18	0	116	11	0	33	0	0	0
CTC_RS00675	complement(119233..119418)	0	0	0	0	0	0	0	0	0	0	0	0	0	0	0	0	0	0	0	0	0	0	0	0	0	0	0	0	0	0
CTC_RS00680	119563..119841	194	85	48	41	0	247	0	169	191	275	461	428	78	105	0	98	90	91	152	173	86	356	268	438	114	120	83	74	264	275
CTC_RS00685	120086..120745	0	0	0	12	35	0	28	95	81	9	49	36	0	22	0	10	38	0	32	147	0	19	57	0	12	34	0	21	37	0
rlmH	complement(120820..121299)	0	49	0	32	0	0	0	0	0	74	0	99	0	0	0	43	0	53	22	0	100	52	0	0	17	0	0	14	0	40
CTC_RS00695	complement(121371..121871)	189	47	159	259	229	413	130	126	160	796	160	524	115	0	209	299	201	202	140	0	239	124	298	325	158	89	46	309	0	383
CTC_RS00700	complement(121902..122081)	0	0	0	42	0	0	104	0	0	0	0	0	0	0	0	0	0	0	24	0	0	0	0	0	0	0	0	115	0	0
CTC_RS00705	complement(122065..122631)	191	84	152	155	283	243	197	111	188	547	57	84	89	52	240	234	44	324	262	85	464	66	0	180	56	217	123	521	238	372
CTC_RS00710	complement(122647..123432)	0	30	0	10	0	44	24	20	0	192	20	0	0	0	0	13	0	113	76	0	91	0	0	0	20	14	29	74	0	49
CTC_RS00715	complement(123445..124701)	161	94	185	219	237	137	230	88	64	543	102	95	189	82	117	298	80	322	396	38	228	99	148	97	151	53	111	317	98	290
CTC_RS14390	complement(124938..125096)	0	0	0	0	0	0	0	0	0	0	0	0	0	0	0	0	0	0	0	0	0	0	0	0	0	0	0	0	0	0
CTC_RS00720	125222..125773	24	43	72	55	125	312	118	86	145	610	102	86	52	53	0	185	91	252	85	263	217	45	135	221	115	101	42	374	0	278
CTC_RS00725	complement(125817..127715)	128	75	28	68	60	27	34	25	14	143	17	25	53	54	22	75	0	57	74	0	50	59	0	64	109	35	49	147	26	20
CTC_RS14080	127891..128241	347	203	38	152	327	344	346	90	228	202	504	136	62	0	60	175	0	36	133	413	0	141	106	0	113	127	66	59	350	109
CTC_RS00735	128258..128917	328	180	141	277	382	340	580	310	121	421	536	181	241	133	127	279	115	96	155	220	399	207	226	370	288	135	211	151	186	174
CTC_RS00740	129072..130868	466	132	155	285	370	556	410	70	89	873	197	359	165	33	128	182	183	240	274	323	386	186	415	272	154	62	90	402	96	203
CTC_RS00750	130992..132328	0	0	10	0	0	0	0	0	0	0	0	0	0	0	0	0	0	0	0	0	0	0	0	0	0	0	0	0	0	0
CTC_RS00755	complement(132395..133444)	0	0	0	0	0	33	0	0	0	22	15	0	0	0	20	6	0	12	0	0	23	0	0	0	0	0	0	0	0	0
CTC_RS00760	133569..134684	36	21	48	14	0	93	33	42	0	74	158	86	0	66	19	6	45	68	11	0	107	0	33	73	0	65	21	25	0	17
CTC_RS00765	134764..135708	0	25	84	16	121	36	30	33	57	41	68	51	46	77	44	22	53	27	31	51	51	0	0	0	8	47	49	51	78	0
CTC_RS00770	135779..136480	116	34	0	22	65	25	66	22	0	25	69	34	72	21	15	68	36	180	73	69	68	35	106	0	85	0	0	69	0	55
CTC_RS00775	complement(136637..136969)	0	0	80	0	0	0	0	0	0	35	145	0	0	0	126	0	0	0	0	0	72	0	0	0	0	0	0	21	0	0
CTC_RS00780	complement(137119..137772)	21	36	0	23	0	0	14	24	0	18	49	0	0	67	0	0	0	19	13	0	0	0	57	124	0	34	0	11	75	88
CTC_RS00785	complement(137800..139146)	40	53	59	45	34	51	21	82	59	132	143	18	21	65	31	66	94	94	73	72	107	55	83	60	35	41	17	87	91	57
CTC_RS00790	complement(139252..139557)	0	0	0	0	0	0	0	0	87	0	0	0	0	0	0	0	82	0	0	0	0	0	0	0	0	73	0	0	0	125
murC	complement(139628..140998)	49	52	68	89	33	25	48	34	19	271	70	122	37	43	15	60	18	74	43	0	52	45	109	0	64	33	34	40	36	98
purR	141177..141995	165	261	195	56	140	631	376	269	261	562	373	189	132	375	282	408	216	402	618	354	438	91	410	298	136	478	142	302	420	398
spoVG	142101..142391	13885	7458	9597	10752	9807	8293	14458	7492	17761	4059	12869	10171	11868	6992	14019	12311	5809	12117	7590	8145	12742	12450	11800	11052	13653	7144	16412	3863	6326	9123
glmU	142543..143910	59	104	146	50	218	239	68	173	59	138	258	419	58	171	153	60	240	120	211	248	332	0	246	223	35	114	85	151	179	182
CTC_RS00815	143935..144891	141	644	584	175	563	558	58	641	1005	247	907	723	238	505	570	327	685	351	344	809	675	201	1014	510	232	479	581	284	385	341
CTC_RS00820	145035..145724	118	138	77	100	100	300	122	183	116	128	116	277	84	85	152	207	183	312	210	70	243	36	0	118	69	146	235	179	462	278
CTC_RS00825	145728..147134	38	17	57	49	16	110	53	22	38	59	11	34	26	10	30	29	54	72	57	0	34	18	27	0	11	24	0	39	0	41
CTC_RS00830	147221..148396	23	222	170	104	195	147	71	335	136	186	150	284	61	162	321	127	451	366	148	370	549	42	95	35	47	238	296	123	376	399
pth	148498..149064	0	42	23	13	0	91	0	83	94	52	28	210	38	26	0	48	0	22	22	171	84	66	66	144	56	39	82	61	130	0
mfd	149090..152170	26	46	47	32	22	62	61	26	26	61	78	139	45	38	61	51	49	95	41	31	47	24	12	53	33	40	38	29	32	50
CTC_RS00845	152208..153227	702	837	1063	438	966	845	366	988	890	533	488	655	506	969	637	501	717	1043	509	142	305	462	787	878	397	822	818	482	734	883
spoVT	153345..153896	0	258	0	0	0	31	0	314	48	0	1194	86	0	875	190	0	411	0	0	175	43	0	0	0	0	648	42	0	356	35
CTC_RS00855	154026..155579	0	0	9	0	15	0	0	0	0	4	31	0	0	19	13	4	0	0	0	0	0	0	0	0	0	32	0	4	0	12
mazG	155576..157024	65	98	46	58	47	167	103	141	55	77	122	115	95	51	72	75	87	61	91	67	33	77	77	28	115	177	96	52	34	40
CTC_RS00865	157135..157410	3134	3180	1735	2765	2076	2217	2400	2452	2710	2579	3261	1730	2864	2811	1672	3035	1371	1284	2434	1227	2167	2922	2569	1770	3419	2005	2772	2416	1423	2535

CTC_RS00870	157483..157728	0	96	0	31	0	0	0	0	0	0	131	0	0	60	0	0	410	0	0	0	0	0	0	0	64	0	0	0	0	0
yabP	157840..158130	46	82	0	0	79	0	0	54	0	0	276	0	0	302	216	0	173	0	15	0	0	0	0	0	0	77	0	0	0	0
yabQ	158139..158561	0	168	16	0	0	41	0	74	0	14	38	0	0	104	50	0	60	60	0	0	57	0	0	0	0	53	27	0	0	0
CTC_RS00885	158632..158904	693	3128	1559	755	2519	2336	957	2220	2055	3115	4622	4765	661	3271	1959	873	3234	3107	919	1240	3417	682	2051	1342	378	2682	1614	1750	4405	2949
CTC_RS00890	158961..159371	1743	3463	1181	1829	2175	1636	1793	3121	2307	1775	2542	3426	1387	2921	2322	1723	1964	1494	2959	1295	1746	1585	2906	1783	1457	2611	1862	2292	3225	2518
spoIIE	160188..162575	6	924	863	48	1200	440	35	873	1275	136	330	740	18	1048	1129	26	222	684	52	182	521	10	438	273	10	850	1000	58	545	3235
tilS	162670..164073	39	68	104	60	49	123	66	101	95	164	34	102	31	83	45	78	90	135	218	69	170	18	53	116	23	115	50	69	35	246
hpt_1	164060..164605	99	348	219	175	336	253	222	86	147	465	236	262	152	214	230	374	323	672	300	266	307	295	205	224	160	184	255	214	315	491
ftsH_1	164704..166515	820	1008	844	1074	835	875	904	916	811	1151	674	1317	924	901	810	1430	794	1251	1531	801	885	719	577	517	919	759	960	1286	718	1423
CTC_RS00940	166633..167016	141	618	519	457	239	449	316	963	209	569	502	1181	489	648	901	585	197	495	543	252	530	808	486	106	413	611	725	322	64	499
CTC_RS00945	167196..167984	137	90	34	87	116	87	71	100	68	165	61	30	110	74	53	129	96	209	291	184	212	79	189	52	70	128	118	113	124	170
dusB	167984..168952	42	98	69	165	71	53	116	276	69	253	133	86	119	45	22	183	78	196	222	100	198	51	193	210	106	92	0	234	101	119
CTC_RS00955	169006..169842	0	85	16	27	27	41	22	56	32	71	0	29	9	140	25	24	0	15	41	0	57	44	89	0	0	27	0	66	59	46
greA_1	169936..170418	476	393	220	395	285	71	386	456	55	562	233	99	374	333	174	423	52	210	537	0	149	244	309	0	345	440	96	512	152	238
lysS	170435..171943	233	204	97	286	273	206	278	250	106	779	234	245	306	146	111	427	351	436	510	224	214	321	420	162	276	141	138	718	260	235
CTC_RS00975	172508..173899	476	315	48	274	99	12	201	79	77	314	92	69	358	74	15	387	18	55	425	104	17	285	54	29	285	120	33	341	53	96
murD	174280..175656	108	103	145	116	117	125	112	160	39	122	70	17	126	122	168	158	18	156	153	70	226	72	81	89	75	195	101	190	160	376
CTC_RS01005	181171..181911	36	32	18	165	31	93	25	21	0	191	22	32	29	20	28	64	0	17	149	65	32	151	101	55	64	30	0	65	0	26
CTC_RS01010	181997..182740	291	64	161	364	200	209	119	148	108	524	238	225	126	138	141	92	34	238	349	228	225	284	401	876	43	135	156	185	0	129
CTC_RS01020	183147..183644	0	143	107	15	0	69	0	63	0	59	0	48	0	29	42	0	0	0	17	0	0	0	0	163	0	45	47	0	0	0
CTC_RS01025	complement(183701..184852)	0	21	12	0	0	0	0	0	0	5	14	0	0	0	0	0	44	0	0	0	42	0	0	35	0	0	0	0	0	0
CTC_RS01030	185007..185219	0	223	125	0	0	0	0	148	0	28	151	0	0	137	0	0	237	119	20	0	0	0	175	0	37	52	0	0	230	0
CTC_RS01035	185198..186613	0	17	56	16	32	37	0	33	38	38	102	17	15	72	15	5	71	40	36	0	68	18	0	29	17	79	33	15	69	54
CTC_RS01040	186642..187328	0	0	19	0	33	25	0	0	0	26	23	0	0	43	0	20	37	18	43	0	70	18	0	0	23	16	0	0	0	28
CTC_RS01045	187399..187728	737	1617	1370	486	834	1097	1074	1240	2266	125	4018	3870	744	1952	2288	475	3211	921	593	1759	1667	414	1923	493	505	2134	2037	250	1413	1046
CTC_RS01050	187744..188697	113	323	585	112	384	1355	230	280	1064	180	977	1276	129	330	440	157	423	518	67	659	777	104	313	811	100	281	741	130	334	844
CTC_RS01055	188703..189611	505	757	1536	529	908	1659	842	658	1646	448	2626	3597	405	886	946	472	1110	1407	454	1596	1882	396	1150	1388	401	873	1505	333	756	1624
CTC_RS01060	189622..189825	530	349	1108	524	1011	2535	412	617	2095	637	1773	2808	212	789	1028	367	989	1241	156	237	1055	122	732	998	155	822	682	270	842	1973
CTC_RS01065	189916..190086	112339	64918	31574	64195	45710	21370	92035	107840	69510	14714	72423	37058	87034	75249	78932	60543	30244	14734	93201	85856	28679	71544	62750	40235	87975	77132	79857	44846	47796	23876
CTC_RS01070	complement(190156..191492)	0	0	0	0	0	0	0	0	0	0	0	0	0	0	0	0	0	0	0	0	0	0	0	0	0	0	0	0	0	0
CTC_RS01080	191678..192433	36	0	0	10	30	0	0	0	0	0	21	63	10	39	0	9	0	50	45	0	0	0	49	0	10	15	0	27	0	0
rsmI	192436..193281	112	42	31	54	54	61	55	56	0	112	38	0	68	35	124	209	0	75	103	0	28	88	88	48	103	53	110	57	0	45
CTC_RS01090	193426..194520	716	282	437	666	649	724	980	633	293	1408	264	698	761	254	278	1079	323	844	1477	618	1049	414	477	521	826	510	423	860	247	788
CTC_RS01095	complement(194726..194968)	0	0	0	0	0	0	0	0	0	0	0	98	0	0	0	0	0	0	0	0	0	0	0	0	0	0	0	0	0	0
fsa_1	195206..195850	241	37	103	112	142	214	347	98	41	142	374	259	274	91	33	264	78	275	86	75	334	154	58	126	221	87	252	117	114	386
CTC_RS01105	complement(196197..197072)	0	0	0	0	0	39	0	0	0	13	18	0	0	0	0	0	0	0	0	0	27	0	0	46	0	0	0	8	0	0
CTC_RS01110	197483..198436	0	0	28	40	48	0	0	16	28	12	84	100	15	31	0	7	0	40	22	0	50	0	0	0	8	23	24	0	77	20
CTC_RS14845	198553..198714	0	0	0	0	0	0	0	0	0	0	298	0	0	0	0	0	0	0	0	0	0	0	0	0	0	0	0	0	0	0
CTC_RS01115	198823..200634	22	26	22	29	13	38	36	26	0	59	62	40	28	8	0	23	56	70	45	67	66	34	0	90	4	6	77	27	14	63
ispF	200659..201135	0	0	28	0	0	0	20	66	56	0	0	0	30	0	44	14	53	27	36	0	0	0	0	0	50	23	49	29	0	0
CTC_RS01125	201266..201649	2921	1483	3082	2504	3462	2289	2041	1640	3756	461	5232	2984	2115	1296	2513	2395	1248	1780	1129	2142	2741	2262	3110	2969	2540	1601	3260	591	1598	3993
CTC_RS01130	201870..203345	586	836	1009	579	1041	1600	904	970	1222	388	773	655	579	665	952	891	1402	1291	557	1180	1929	496	1264	1848	527	750	895	522	1197	1260
CTC_RS01135	complement(203391..203627)	0	0	56	0	0	0	0	0	0	75	0	0	0	0	0	29	0	0	0	0	0	0	0	0	0	47	0	0	0	81
CTC_RS01140	complement(203720..205174)	9	0	18	16	32	24	0	11	0	45	0	0	0	10	29	0	0	17	18	0	33	0	0	0	0	8	0	0	17	53
trkA	complement(205125..206531)	0	17	9	0	0	0	7	0	19	25	11	34	0	0	0	0	36	18	12	0	17	0	0	0	0	0	0	5	0	14
CTC_RS01150	206889..207464	0	41	0	0	40	0	0	27	0	0	0	0	0	51	36	0	0	44	0	0	0	43	0	0	0	0	0	0	0	33
CTC_RS01155	207507..207986	0	49	0	0	48	0	0	0	0	25	33	0	0	0	0	0	0	26	0	0	0	0	0	0	0	0	0	0	0	0
CTC_RS01160	208056..208781	74	196	128	137	284	214	103	152	221	122	244	33	89	242	173	178	104	227	217	267	297	34	257	224	55	154	96	28	203	317
CTC_RS01165	208774..210420	49	94	48	42	125	372	96	38	16	174	146	116	53	84	76	70	61	208	72	29	290	60	227	222	39	68	56	96	246	198
CTC_RS01170	210458..211024	238	920	586	162	970	1049	165	361	565	333	1106	379	76	465	259	288	623	603	285	1024	970	109	658	1292	168	444	572	109	952	541
metG	211425..213350	519	148	104	293	196	107	279	123	69	202	342	285	304	236	109	258	262	151	248	226	161	309	116	148	280	281	12	159	89	129
CTC_RS01180	213502..214269	70	185	17	0	0	22	36	0	0	23	42	0	28	57	0	27	66	33	61	0	0	0	49	0	21	58	0	18	64	25
CTC_RS01185	214543..215583	351	3008	1597	1210	1916	1921	663	2314	2232	1841	3829	1295	534	3902	1733	1155	3478	1885	1238	2091	1643	906	2617	1095	488	3162	1514	1691	3796	2762
rnmV	215650..216207	0	43	0	41	0	31	0	0	48	64	115	43	0	26	38	0	0	45	61	0	129	111	0	0	14	40	42	0	0	137
rsmA	216194..217033	32	56	32	18	55	185	33	37	32	141	38	114	26	52	50	32	30	75	35	115	114	15	222	97	19	53	0	25	117	46
CTC_RS01200	217116..218405	42	1710	1866	106	515	321	43	1586	1108	224	2417	407	45	2831	878	90	2063	957	110	600	315	67	723	379	80	2378	1123	107	2131	951
CTC_RS01205	218519..219109	0	80	23	0	0	29	0	27	45	10	0	0	0	25	0	0	85	0	0	82	0	0	0	0	0	76	0	0	0	32
CTC_RS01210	219201..219950	180	411	213	142	336	299	236	336	71	291	343	191	77	351	587	136	572	203	272	129	223	50	224	271	63	328	185	137	295	256
CTC_RS01215	219980..220819	145	198	237	145	191	267	222	206	159	204	344	256	172	331	275	195	375	286	251	288	285	177	178	339	104	346	110	221	321	342
CTC_RS01220	220842..221684	946	1435	978	1059	1387	879	863	1550	1077	886	1125	2378	826	1381	1480	1204	2125	1524	1301	1549	1703	736	1417	966	800	1512	1265	966	1747	1387
CTC_RS01225	221802..221993	0	494	277	0	358	90	0	656	696	31	1381	497	0	1220	328	0	131	0	0	252	0	0	194	0	0	1223	121	0	639	100
thiT	complement(222035..222682)	21	37	82	47	106	213	0	49	82	46	50	0	0	0	129	53	117	39	7	0	148	0	0	63	0	17	0	11	38	89
CTC_RS01235	complement(222915..224252)	0	0	0	0	0	0	0	0	0	0	0	0	0	0	0	0	19	0	0	36	0	0	0	0	0	0	17	0	0	0
CTC_RS01240	224794..226908	128	247	107	72	266	122	185	164	51	159	38	56	72	69	40	116	60	198	137	23	68	41	212	77	116	53	22	133	93	181
nrdG	226913..227422	79	186	156	180	157	169	73	309	52	139	32	47	156	144	0	214	297	261	150	95	0	97	73	319	124	22	0	135	144	150
CTC_RS01250	227491..227856	332	583	727	417	564	706	204	710	839	871	1537	717	217	760	1175	437	655	1280	459	529	850	305	1530	222	282	611	633	676	872	655
murI	228081..228863	863	30	34	68	146	22	346	40	0	68	349	30	69	19	80	139	32	65	92	0	61	16	191	52	91	43	0	97	0	24
CTC_RS01260	229023..229358	80	0	79	0	205	51	83	141	0	88	144	213	21	44	125	0	0	38	13	0	285	37	222	242	24	100	69	102	0	57
CTC_RS01265	229373..231163	566	490	275	430	518	472	771	611	224	448	547	700	520	405	410	635	859	502	806	729	668	339	500	591	549	549	298	309	754	471
ileS	231549..234659	313	152	77	282	37	89	256	111	34	475	52	100	260	122	81	343	259	326	366	124	131	231	84	65	277	83	15	298	174	148
CTC_RS01275	234852..235850	568	380	466	294	390	1070	392	457	267	443	708	1242	296	264	252	484	530	494	283	339	431	248	411	285	349	324	371	509	541	537
CTC_RS01280	236036..240094	3	281	355	8	333	221	11	349	484	9	315	423	7	382	543	27	221	459	2	131	306	0	101	70	0	329	554	5	97	1237
CTC_RS01285	240171..241238	13	1110	1127	0	687	226	17	1776	1050	11	1023	548	7	2467	1689	6	697	901	20	408	336	12	367	76	0	2507	2095	6	1287	503
CTC_RS01290	241365..242747	0	566	740	22	414	162	0	933	676	26	721	207	5	937	629	0	620	568	6	0	173	18	243	59	6	1059	671	15	550	277
CTC_RS01295	242883..244109	0	0	22	0	0	14	0	0	22	24	0	0	12	6	17	11	0	31	28	0	19	0	0	33	6	9	0	14	40	62
CTC_RS01300	complement(244146..245270)	0	21	95	7	20	0	0	14	0	0	100	42	0	13	37	6	45	45	0	0	43	0	66	36	4	30	41	6	44	51
CTC_RS01305	245444..246469	0	0	39	0	22	0	0	31	0	0	16	0	0	86	20	0	123	86	2	0	0	0	0	40	0	44	0	0	120	75
CTC_RS01310	246462..247214	0	63	71	0	61	0	0	146	106	0	64	32	5	78	56	9	235	67	11	64	0	0	50	0	0	59	62	0	98	0
CTC_RS01315	247225..248241	0	70	216	15	23	34	0	186	79	6	205	117	7	202	82	0	422	249	0	0	0	12	110	0	8	324	182	0	434	113
CTC_RS01320	complement(248280..249434)	0	41	207	0	0	15	8	89	93	0	70	62	6	89	54	0	175	241	0	0	41	0	0	70	0	68	60	0	43	116

CTC_RS01325	249573..250577	0	260	93	0	182	34	0	219	27	0	400	47	0	459	313	0	402	151	0	0	71	0	149	41	0	323	92	0	171	172
CTC_RS01330	250732..251769	299	1211	564	66	1457	598	135	1592	592	91	759	1127	125	1410	667	256	656	378	279	2517	1982	131	1798	941	153	1206	570	533	3358	425
CTC_RS01335	251831..253513	8	42	8	5	82	113	6	65	16	28	57	28	4	78	12	12	0	30	18	29	256	7	133	24	0	40	41	57	284	57
CTC_RS01340	253525..254604	50	132	74	0	85	64	35	58	0	5	45	44	33	81	19	0	47	106	28	134	222	23	69	38	11	41	21	76	318	35
CTC_RS14395	254876..256828	7	0	0	39	35	26	24	24	7	73	0	12	18	0	21	24	13	104	28	0	49	6	38	21	0	17	24	67	0	20
CTC_RS01355	256816..257112	46	0	0	0	0	58	31	0	0	0	0	80	24	0	0	23	0	0	14	0	0	0	0	0	0	0	78	46	83	65
CTC_RS01360	257099..257383	0	0	0	80	0	0	82	55	0	41	0	42	0	0	74	0	0	0	15	0	0	0	0	0	0	0	0	84	86	0
CTC_RS01365	complement(257329..257571)	0	0	0	0	0	0	0	0	0	0	0	0	0	0	0	0	0	0	0	0	0	0	0	0	0	0	0	0	0	0
fsa_2	257809..258291	0	0	0	0	0	0	0	0	0	0	0	0	0	0	0	0	0	0	0	0	0	0	0	0	0	0	0	0	0	0
CTC_RS01375	258330..260339	34	47	7	8	46	26	23	23	106	32	32	53	7	73	52	47	25	31	27	0	12	12	19	0	8	39	23	65	61	57
yabG	260455..261342	15	508	629	9	77	116	0	709	331	20	217	81	8	1162	425	0	710	741	19	218	108	28	252	0	9	1032	496	39	995	216
CTC_RS01385	261616..261849	3147	1318	1193	3555	882	1031	3030	1076	1199	1943	1992	4947	2313	2502	2689	1397	1725	1353	2353	5168	716	2121	1754	1740	2677	1815	1288	2762	1259	1802
CTC_RS01390	262220..263782	0	607	145	39	147	55	24	453	85	57	977	15	5	1400	174	13	597	138	19	263	0	0	0	0	0	851	74	35	644	270
CTC_RS01395	263894..264691	0	30	33	0	0	0	0	0	0	0	40	30	0	92	26	0	0	16	0	0	0	0	0	0	0	42	0	0	0	0
cphA	264695..267319	5	36	0	3	35	0	0	12	10	4	73	0	0	45	0	3	67	14	2	0	9	5	0	0	0	38	9	0	28	15
ispE	267405..268247	0	56	0	18	27	41	0	75	0	42	133	0	9	35	75	40	0	15	30	0	0	29	0	48	9	80	0	33	87	68
CTC_RS01410	268353..268553	0	0	0	0	0	0	0	0	0	0	0	0	0	0	0	0	0	0	0	0	0	0	0	0	0	0	0	0	0	0
CTC_RS01415	268571..270043	0	16	0	21	0	12	0	0	18	0	33	0	5	50	14	5	0	9	0	0	0	0	0	0	5	38	16	0	0	0
CTC_RS01420	270067..271167	0	0	0	7	0	0	8	0	24	5	0	0	0	0	0	0	0	0	0	0	0	11	0	0	0	30	0	0	0	0
CTC_RS01425	271160..272344	0	60	0	19	19	0	12	40	0	0	14	20	6	49	0	0	43	11	4	0	0	0	0	34	0	47	78	12	41	0
spoIIR	272424..273059	0	0	21	24	0	27	0	25	0	0	0	0	0	23	0	0	0	0	0	0	0	0	0	0	0	18	0	0	0	0
CTC_RS01435	complement(273074..274243)	23	20	102	46	59	177	32	54	46	222	27	20	6	13	0	82	65	76	62	0	82	0	64	35	34	67	99	112	21	66
ypeB	274467..275834	0	52	49	6	17	0	0	115	0	4	482	52	0	353	46	5	129	19	6	35	35	0	0	0	0	327	34	0	108	0
CTC_RS01445	275976..277001	184	69	26	216	201	168	191	169	130	161	78	186	204	157	204	232	123	234	215	189	163	181	146	357	255	185	158	127	48	131
CTC_RS01450	277022..278002	393	314	203	334	491	228	247	401	245	283	328	219	537	433	246	496	180	374	416	247	341	341	380	290	356	422	47	252	175	391
CTC_RS01455	278023..278445	96	168	63	198	54	41	110	149	189	140	76	56	239	242	99	161	60	180	141	0	0	147	176	0	187	225	55	179	0	45
CTC_RS01460	278818..279120	0	0	0	0	0	0	0	0	0	0	53	0	0	48	0	0	0	42	0	0	0	41	0	0	0	0	0	0	0	0
CTC_RS01465	279260..279607	39	204	0	0	0	0	0	0	0	17	0	0	21	0	0	20	72	146	49	0	0	0	107	0	23	32	0	20	0	0
rho	279807..281276	653	468	280	786	452	399	685	546	345	534	328	276	840	339	371	853	326	547	836	362	545	772	406	249	777	228	315	533	117	613
phnD	281385..283265	57	38	28	93	61	64	60	33	14	25	137	76	119	16	33	51	67	108	49	26	13	66	79	22	55	30	0	26	39	41
rpmE	complement(283341..283553)	1554	1225	1935	1469	1560	2347	876	813	1630	2496	1811	3362	1355	1581	1674	1599	1421	1724	2076	1136	3706	1340	1665	2102	1080	1574	2177	3421	1037	1980
CTC_RS01485	283757..284335	0	0	0	66	40	30	48	54	46	82	28	41	0	0	36	59	0	22	103	0	41	21	64	70	68	77	160	107	0	66
CTC_RS01490	284345..285253	15	0	0	17	50	0	10	0	0	143	0	0	64	16	0	37	0	14	70	0	53	55	0	0	9	25	0	38	27	42
prmC	285272..286150	31	27	91	87	52	78	21	54	30	74	37	0	74	50	24	39	0	58	87	55	54	28	0	46	27	140	26	55	84	109
prfA	286215..287282	392	689	797	529	665	678	480	604	563	807	497	1117	338	473	628	593	520	996	625	498	952	349	734	686	327	628	499	708	781	978
CTC_RS01505	287305..287901	0	40	22	64	38	0	16	0	0	49	27	40	24	12	0	23	42	21	36	0	40	0	63	68	0	0	0	12	0	128
CTC_RS01510	287983..288684	0	101	0	33	65	0	53	90	0	50	69	68	21	188	60	29	108	18	12	0	34	0	0	58	23	96	0	20	0	55
CTC_RS01515	288708..289760	39	248	101	94	65	65	71	135	76	129	336	181	41	334	40	129	216	228	69	0	91	24	142	155	75	149	44	46	233	255
CTC_RS01520	289846..290298	0	52	29	17	0	38	0	0	0	176	35	211	0	32	0	30	0	140	38	0	0	55	165	0	18	25	0	91	108	42
rpiB	290354..290806	119	209	147	270	202	190	144	139	177	150	284	105	207	162	232	165	111	224	178	320	158	110	247	449	105	173	51	167	54	212
upp	290828..291457	450	565	317	285	291	246	237	325	382	197	281	227	458	441	233	335	320	322	560	691	38	335	533	129	290	328	147	404	351	91
CTC_RS01535	291586..292074	28	146	82	62	47	0	143	193	55	60	66	49	44	120	172	293	103	155	148	297	196	25	229	167	114	160	190	225	251	0
CTC_RS01540	292088..293122	52	46	90	66	133	100	72	30	52	131	78	69	70	57	0	224	244	220	94	93	185	108	36	118	38	32	0	166	142	259
wecB	293150..294298	47	103	69	83	100	195	85	164	23	116	70	291	107	25	0	148	176	154	104	84	146	140	195	177	62	68	40	191	150	50
CTC_RS01550	294440..295615	45408	10670	8990	52039	9054	7834	57806	8573	6950	3980	14201	19190	60844	4369	6662	52994	11971	2013	37573	38954	12317	53588	7776	5504	65078	6097	7610	6810	15590	9747
CTC_RS01555	295823..296473	0	182	654	0	176	132	0	508	123	0	148	73	0	427	548	0	233	778	7	0	73	0	115	0	0	652	534	5	302	412
murA	296500..297753	0	170	339	0	73	82	0	176	277	5	90	19	0	321	201	0	262	525	3	0	38	0	30	65	0	432	259	5	372	122
spoIID	297829..298887	0	157	308	7	0	65	0	268	76	17	197	90	0	290	158	6	298	395	0	0	0	0	70	77	0	222	44	26	324	145
CTC_RS01570	299052..299765	0	498	93	11	64	0	0	331	0	0	1058	67	0	1435	88	0	212	35	6	203	34	0	0	57	11	697	162	0	172	81
spoIIID	299875..300129	0	186	0	0	0	0	0	0	0	0	63	94	0	172	0	0	99	0	0	0	0	0	0	0	0	197	0	0	96	75
CTC_RS01580	300216..301247	223	713	387	222	311	367	280	381	362	252	467	532	161	489	396	406	342	607	593	516	556	120	705	394	231	672	348	373	404	316
yyaC_2	complement(301269..301826)	0	85	48	0	0	0	0	56	0	32	403	0	0	275	38	0	226	136	15	173	0	0	134	0	0	80	42	12	88	206
CTC_RS01590	complement(301892..302602)	0	0	19	0	0	73	0	44	38	33	0	34	0	0	0	0	0	36	18	0	67	0	0	0	0	31	33	48	35	0
metK	303526..304701	368	282	339	214	351	425	294	415	318	369	431	365	282	710	473	513	536	560	524	699	386	106	349	242	314	960	276	804	209	456
CTC_RS01620	304873..307104	36	85	36	58	10	124	46	85	36	103	58	139	29	66	28	108	11	102	133	43	171	39	33	82	18	90	73	105	11	120
CTC_RS01625	307118..308149	13	0	13	15	67	33	27	0	0	52	31	0	7	0	20	26	24	37	4	47	23	0	0	0	0	22	0	40	0	74
CTC_RS01630	308122..308796	20	70	59	68	68	51	14	0	40	83	71	0	11	0	0	20	75	56	13	0	142	18	0	0	23	66	0	31	73	28
raiA	309207..309737	3957	3708	4783	1681	3712	3733	8811	3468	4452	2341	10715	7754	2766	5734	6399	3534	10547	4852	2194	4418	4280	1495	4006	4677	3360	5673	5021	3230	9360	4909
CTC_RS01640	complement(309757..310533)	0	92	0	20	0	111	0	0	0	15	0	0	9	19	54	0	32	16	5	0	31	16	0	0	10	14	0	0	63	25
secA	310734..313244	129	359	410	240	256	563	195	257	266	387	506	993	227	257	342	342	502	424	350	511	667	230	282	251	174	256	471	298	147	420
prfB	313313..314417	110	258	361	145	83	265	144	157	326	192	524	713	176	384	569	277	616	355	279	350	541	213	169	368	115	223	420	352	422	173
CTC_RS01655	complement(314463..315806)	0	18	20	0	0	26	0	0	0	4	24	18	0	11	16	0	0	9	0	0	18	0	0	0	0	0	0	5	18	29
CTC_RS01660	315916..318072	169	176	160	188	181	160	143	219	161	159	320	177	228	217	243	259	105	223	214	135	266	230	121	208	283	249	183	84	68	151
CTC_RS01665	318625..319239	242	309	627	223	727	1009	91	640	1433	643	601	1164	141	405	853	210	902	679	691	708	856	202	607	1324	103	518	1018	369	718	1683
CTC_RS01670	319315..320841	212	16	17	30	45	45	266	10	52	97	21	141	80	10	69	67	50	41	72	0	16	16	49	0	83	22	46	104	32	25
CTC_RS01675	320867..321130	512	449	353	491	434	522	954	417	405	872	670	1085	383	444	636	464	573	671	596	550	181	282	141	925	511	339	878	417	465	363
CTC_RS01680	321139..321825	256	311	116	300	167	464	367	92	117	421	234	347	257	256	183	248	367	258	285	282	70	199	109	356	185	179	101	380	179	432
CTC_RS01685	complement(321872..322651)	17	91	34	117	118	221	72	161	68	288	103	214	93	66	108	79	65	130	144	124	123	95	144	157	102	72	59	106	31	98
CTC_RS01690	322793..323581	240	150	169	155	203	262	201	120	169	150	244	182	101	186	133	224	192	353	280	123	364	142	355	413	181	198	176	157	342	170
leuS	complement(323610..326048)	100	58	60	167	47	35	107	84	22	446	33	20	139	66	69	293	52	122	152	0	29	168	77	50	189	37	10	309	50	39
CTC_RS01705	complement(326500..327060)	0	0	24	0	0	61	0	0	0	11	0	0	0	0	0	0	0	0	0	0	0	22	0	0	0	0	0	0	0	34
CTC_RS01710	327205..327411	0	344	385	0	0	167	0	380	1807	0	155	115	0	354	811	0	488	245	0	234	116	60	0	393	0	540	336	0	356	0
CTC_RS01715	327433..327711	0	425	667	0	164	371	0	508	1484	0	115	0	0	944	601	0	452	1225	0	0	86	0	134	876	0	801	1080	0	352	893
CTC_RS01720	complement(327781..328680)	0	0	30	0	25	0	0	52	0	7	0	0	0	16	47	0	0	0	0	0	0	0	0	0	0	12	0	0	27	0
CTC_RS01725	328825..329859	26	46	26	92	22	150	36	15	155	120	124	92	63	57	101	86	0	86	66	0	23	84	0	79	46	22	112	53	47	111
fba	complement(329898..330848)	1648	898	713	802	482	961	1118	910	871	1050	1538	1305	960	508	419	849	902	519	796	1144	1333	828	1295	578	1117	588	658	716	723	1129
CTC_RS01735	complement(331126..331506)	0	0	0	0	0	0	0	0	0	0	127	0	0	0	0	0	0	0	0	0	0	0	0	0	0	0	0	0	0	50
CTC_RS01740	complement(331525..332193)	0	0	60	0	0	0	0	24	0	9	84	36	0	0	31	0	38	38	0	0	0	0	112	0	0	50	0	0	220	29
CTC_RS01745	332318..333976	65	164	120	53	111	94	112	171	161	50	136	72	100	115	190	45	30	126	94	58	115	30	45	49	105	148	119	29	89	173
CTC_RS01750	334054..334392	0	175	118	68	135	102	28	232	79	52	237	0	43	216	0	60	74	168	50	0	141	37	110	0	70	33	0	41	72	57

pssA_1	complement(334431..334886)	59	52	146	134	201	189	0	0	59	298	176	52	0	32	46	119	0	83	37	0	472	54	164	893	35	25	102	166	54	126
CTC_RS01760	335150..335581	0	494	431	0	0	160	0	838	556	0	335	111	0	1288	777	32	993	59	0	112	55	0	0	283	0	1552	805	0	966	266
CTC_RS01765	335684..336631	43	25	28	80	97	73	39	17	28	255	119	50	15	31	22	36	27	80	166	102	76	0	276	43	25	12	0	87	78	142
sleB	336944..337642	77	34	57	87	131	74	27	158	38	34	506	68	21	251	30	10	361	18	207	138	0	36	214	58	11	256	66	10	351	82
cysE	338007..338567	145	127	119	204	41	92	482	196	48	84	57	43	270	157	112	279	405	135	538	172	43	177	67	0	254	139	124	159	350	103
queG	338583..339566	55	0	0	147	116	88	133	48	54	102	33	49	117	119	64	42	51	64	78	0	0	50	38	0	81	68	0	70	0	58
CTC_RS01785	339601..340308	95	34	131	140	162	195	132	111	38	100	91	135	51	83	119	144	71	268	60	68	34	70	0	58	118	95	131	97	243	190
nth	340305..340943	106	186	42	215	179	189	204	99	42	120	377	374	192	206	164	320	158	297	146	227	374	78	350	64	68	175	181	108	230	120
CTC_RS01795	341149..342318	4077	1237	977	3255	1362	958	4334	982	1119	1035	1140	1530	4451	1214	1533	6747	1013	1098	1931	889	1206	2911	829	626	4124	1213	1744	993	902	1401
CTC_RS01800	complement(342374..343603)	44	77	238	53	93	210	76	38	65	91	78	39	82	131	85	33	103	82	66	354	78	91	61	364	84	45	94	17	20	125
CTC_RS01805	343749..344324	23	124	162	93	119	30	73	191	278	174	112	41	100	102	146	71	0	22	185	0	208	108	194	141	110	175	161	48	43	100
CTC_RS01810	344328..345149	279	491	534	288	809	168	136	833	536	111	391	145	184	855	536	83	399	231	362	824	378	106	272	149	96	857	508	84	328	47
CTC_RS01815	345426..346805	59	69	58	61	100	87	68	34	97	223	47	130	58	32	15	84	18	165	176	35	121	36	0	89	40	89	134	147	160	125
CTC_RS01820	346876..347826	14	25	28	8	0	18	10	0	56	12	17	25	0	0	0	0	27	0	18	0	0	0	0	0	0	0	0	0	0	40
CTC_RS01825	347823..348983	0	0	0	0	0	30	8	0	0	0	0	21	0	0	0	0	11	11	4	0	0	0	0	0	0	0	0	6	0	0
tnpA_1	349100..349498	0	0	0	0	0	0	0	0	0	0	0	0	0	0	0	0	0	0	0	0	0	0	0	0	0	0	0	0	0	0
tnpB_1	349508..350593	0	0	0	0	0	0	0	0	0	0	0	0	0	0	0	0	0	0	0	0	0	0	0	0	0	0	0	0	0	0
CTC_RS01840	350732..352954	12	21	6	2	10	16	0	7	0	13	22	11	0	0	0	0	11	0	11	22	0	6	0	18	11	5	0	9	0	9
CTC_RS01845	353039..353737	19	102	114	44	98	74	67	405	153	84	184	137	41	147	135	49	108	109	91	69	274	53	267	175	34	192	66	39	35	137
CTC_RS01850	353817..354638	16	115	129	37	279	84	102	153	260	108	117	87	35	125	77	149	31	92	119	177	233	15	136	50	39	122	141	50	30	93
trmL	354716..355183	0	203	114	65	147	184	80	303	114	151	69	51	62	219	90	160	0	149	291	103	665	0	0	174	51	96	50	29	52	82
CTC_RS01860	355199..356041	0	169	110	27	299	143	111	187	32	98	172	283	43	174	75	97	209	421	171	344	284	0	44	193	28	133	138	90	233	296
CTC_RS01865	356136..357215	5987	3833	2869	6052	3916	3256	4867	3644	3462	837	6809	4033	8133	3185	2894	8076	3901	2386	4915	5420	4164	5928	4596	3053	7894	2888	3209	1610	3591	2396
CTC_RS01870	357281..358825	586	215	77	309	148	45	459	148	52	126	52	124	432	142	14	571	49	107	671	219	186	189	217	53	400	137	15	280	64	62
CTC_RS01875	358818..359906	323	131	37	175	42	63	146	87	25	239	30	44	182	40	19	306	0	47	279	44	66	68	137	75	284	10	0	196	68	35
CTC_RS01880	359899..360819	968	129	130	447	473	131	344	171	203	612	87	26	533	127	91	673	27	179	632	53	299	498	122	133	629	97	76	515	400	250
CTC_RS01885	complement(360892..362430)	79	77	156	99	134	280	121	41	87	180	115	124	84	48	95	106	115	222	41	31	311	32	121	331	72	22	136	80	72	187
CTC_RS01890	complement(362449..363555)	98	129	108	124	166	93	206	28	24	144	44	86	91	13	38	92	23	217	27	44	130	45	135	147	36	10	105	87	0	277
CTC_RS01895	complement(363628..364614)	233	216	296	186	209	838	298	128	298	545	195	169	102	163	404	221	307	385	82	49	436	226	227	660	121	215	446	286	124	777
rpoN	364944..366329	29	51	19	17	50	50	40	45	19	72	139	69	31	11	15	5	91	55	28	70	121	36	54	88	17	48	17	5	18	14
CTC_RS01905	366477..367499	793	93	143	321	112	388	903	169	131	869	141	93	339	57	41	273	74	705	432	47	164	364	73	199	295	186	45	706	48	375
gap	367595..368602	10792	871	1801	9376	1228	2308	14114	828	2411	3155	797	1444	12216	523	916	6463	701	3423	5226	768	1661	9484	1333	3352	11703	632	1311	2073	219	1883
CTC_RS01915	368711..369907	3974	436	333	3698	517	403	4937	171	223	715	222	299	5828	330	280	6037	685	1883	2490	323	360	4333	390	408	5797	364	165	936	123	576
tpiA	369938..370687	4838	569	231	4782	428	345	7413	231	178	1445	343	573	5254	351	196	6707	505	1933	2551	193	191	5848	299	136	5525	298	124	720	164	741
gpmI	370698..372239	4952	323	280	5369	431	525	5215	235	329	994	229	341	5753	247	156	7502	491	2139	2633	188	419	6031	266	198	5760	275	150	972	223	609
eno	372258..373553	7164	558	451	7649	601	519	8757	413	505	1554	347	681	11617	587	356	14403	837	2452	4959	448	554	9062	590	503	11244	655	286	1464	208	740
secG	373742..373966	60	211	355	136	306	153	207	70	0	551	500	318	225	520	746	30	112	338	246	215	744	165	498	1267	211	149	206	92	218	85
CTC_RS01940	374053..376074	819	1003	908	1034	1315	1023	704	817	773	1355	779	850	1023	666	840	1408	774	1500	1014	1435	1432	859	997	483	961	727	837	1268	692	1147
rnr	376266..378392	19	123	75	83	54	122	53	107	75	111	212	79	68	138	69	125	148	173	86	148	135	35	123	96	41	184	196	102	115	72
CTC_RS01950	378641..378940	0	633	44	127	229	115	93	52	89	266	54	159	48	98	140	114	0	464	28	0	239	41	249	543	132	37	155	23	82	192
smpB	379026..379496	86	101	226	49	389	274	79	201	170	596	137	355	61	155	134	260	161	296	181	205	305	0	79	346	51	142	295	204	313	448
CTC_RS01960	380624..381466	0	0	0	0	0	0	0	0	0	0	0	0	0	0	0	0	0	0	0	0	0	0	0	0	0	0	0	0	0	0
CTC_RS01965	381459..382745	0	0	0	0	0	0	0	0	0	0	0	0	0	0	0	0	0	0	0	0	0	0	0	0	0	0	0	0	0	0
CTC_RS01970	complement(382957..383979)	0	0	0	0	0	0	0	0	0	0	0	12	0	0	0	0	0	0	0	0	0	0	0	0	0	0	0	0	0	0
CTC_RS01975	384103..385182	0	0	0	0	0	0	0	0	0	0	0	0	0	0	0	0	0	0	0	0	0	0	0	0	0	0	0	0	0	0
CTC_RS01980	385442..385852	0	58	32	19	167	168	23	191	65	0	39	0	0	142	255	50	123	31	10	0	116	30	91	99	0	82	282	50	0	140
CTC_RS01985	385970..386656	20	0	39	0	100	75	0	0	0	34	23	0	0	0	0	0	0	18	19	0	70	36	54	59	12	33	0	10	71	56
CTC_RS01990	386678..387727	0	23	38	22	22	131	9	60	0	51	92	23	7	42	20	13	48	48	16	0	46	12	36	0	0	21	0	13	23	55
CTC_RS01995	387847..388617	0	31	138	0	89	425	0	20	0	0	21	0	9	0	54	9	0	0	6	0	0	16	145	53	0	14	30	0	0	0
CTC_RS02000	388628..390595	7	12	27	6	58	175	0	8	14	3	8	12	0	0	11	3	0	6	0	0	12	0	38	83	0	23	0	10	0	0
CTC_RS02005	390730..391404	0	141	197	34	441	996	14	23	0	35	0	35	0	65	0	66	112	169	0	72	248	18	276	302	0	33	34	20	182	227
CTC_RS02010	391418..391651	116	0	0	0	98	0	0	67	0	0	0	0	0	0	0	0	0	0	0	0	204	0	0	0	0	48	0	29	105	0
CTC_RS02015	391663..392190	0	45	50	29	217	718	0	60	51	22	0	0	27	0	40	26	0	72	20	92	136	0	212	347	0	0	0	78	139	73
CTC_RS02020	complement(392204..393094)	15	27	45	26	26	0	10	0	0	93	18	40	0	33	47	8	0	57	14	0	27	0	126	0	0	25	0	31	0	0
CTC_RS02025	393294..394553	11	0	32	18	18	41	22	37	0	23	0	19	11	12	0	11	0	10	37	38	38	10	0	32	31	9	0	87	39	30
CTC_RS02030	394803..396311	143	236	44	157	243	183	105	104	159	247	85	111	38	107	139	153	33	151	203	32	127	25	148	162	68	111	154	287	98	152
CTC_RS02035	396524..398716	25	43	73	52	10	31	9	29	37	124	22	87	49	33	96	96	35	104	95	22	44	0	34	37	51	20	42	50	22	61
CTC_RS02040	complement(398872..399390)	130	46	384	162	88	365	198	152	360	239	248	782	111	282	283	210	49	342	123	373	484	239	575	549	107	43	89	185	47	185
CTC_RS02045	complement(399487..399690)	199	581	652	636	225	1183	457	154	917	926	394	585	35	215	206	584	124	434	167	0	352	365	0	798	505	274	796	438	120	658
CTC_RS02050	399915..400664	0	32	71	0	0	23	0	63	36	0	0	32	0	98	28	0	0	135	0	0	0	0	0	0	11	75	155	0	131	0
CTC_RS02055	400654..401451	0	149	250	0	29	65	0	79	67	7	30	60	0	275	171	0	237	214	0	0	30	0	0	0	0	168	232	0	277	72
CTC_RS02060	401456..402478	7	510	572	7	90	101	9	431	731	12	189	93	7	715	349	0	949	675	4	0	23	0	109	40	0	732	453	13	660	319
CTC_RS02065	402595..403758	209	224	91	318	236	178	208	203	92	365	221	246	366	365	198	439	108	228	261	291	247	368	289	70	395	144	139	213	84	165
CTC_RS02070	403813..404451	85	111	21	119	215	0	161	25	84	157	101	37	136	46	66	107	39	59	60	76	0	117	117	0	99	35	73	65	38	60
hcp	404598..406310	47	14	39	13	27	0	82	0	0	100	47	42	34	0	49	20	59	37	5	28	0	22	22	0	60	13	14	32	14	34
CTC_RS02080	406476..407162	0	0	0	0	0	0	0	0	0	0	0	0	0	0	0	0	0	0	6	0	0	0	0	0	0	0	0	0	0	0
CTC_RS02085	407167..408621	0	0	0	0	0	0	0	0	0	0	0	0	0	0	0	0	0	0	9	0	33	0	0	0	0	0	0	9	34	0
CTC_RS02090	408683..409882	0	0	0	0	0	0	0	0	0	0	0	0	0	0	0	0	0	0	19	121	100	0	0	34	0	0	0	63	20	48
CTC_RS02095	409896..410273	36	0	35	121	0	46	99	42	71	187	43	63	76	39	0	54	67	67	11	0	127	33	0	0	63	30	123	109	130	152
CTC_RS02100	410415..411149	0	65	36	0	31	23	0	43	0	0	66	32	0	139	29	0	0	17	12	66	33	0	0	0	0	30	0	28	33	0
CTC_RS02105	411169..411546	0	63	0	0	61	46	25	83	71	16	298	0	19	232	0	0	67	33	11	0	0	0	0	0	0	177	61	18	130	51
CTC_RS02110	complement(411659..413212)	9	0	34	0	15	0	12	0	0	4	103	15	0	57	13	4	32	33	5	0	0	8	24	0	0	36	15	4	16	0
CTC_RS02115	complement(413308..413643)	0	0	0	0	0	0	0	0	0	0	0	0	0	0	0	20	0	0	0	0	0	0	0	0	0	0	0	0	0	0
CTC_RS02120	413931..415319	10	17	10	11	17	12	13	0	0	30	23	0	5	11	0	25	0	0	55	0	0	0	0	0	6	8	0	25	18	0
CTC_RS02125	415386..416528	0	42	47	53	20	38	41	0	70	72	56	42	38	38	37	77	88	22	89	0	42	0	33	0	35	59	41	102	0	84
CTC_RS02130	416718..418277	17	61	85	54	103	110	60	81	17	167	52	138	51	19	0	74	65	106	80	0	107	32	48	104	25	36	45	53	63	123
CTC_RS02135	complement(418324..418782)	0	103	87	0	0	38	0	137	0	0	490	156	0	159	0	0	220	0	19	105	0	0	81	177	0	73	0	0	214	84
CTC_RS02140	418970..420079	2045	1239	1354	1702	1301	1561	1681	936	1444	1351	1520	1462	2227	1306	1134	2721	852	1494	2122	2005	3276	2034	1479	1962	2129	1138	1546	1743	1415	2668
CTC_RS02145	420337..420771	0	0	0	0	0	79	0	0	0	0	0	55	0	34	0	0	0	29	0	0	0	0	0	0	0	0	0	0	0	0
CTC_RS02150	420773..422509	16	14	23	9	13	10	5	0	0	17	9	0	0	0	12	4	0	7	10	0	0	29	0	0	5	6	0	0	0	11

CTC_RS02155	422502..424277	0	27	0	26	0	19	21	0	15	33	27	13	8	25	24	8	14	36	19	0	0	7	42	23	4	13	13	23	14	0
CTC_RS02160	424421..426253	0	13	65	4	25	19	0	0	15	13	0	0	0	8	11	7	41	35	21	0	0	0	41	0	0	6	51	19	0	0
CTC_RS02165	426277..426549	0	87	49	0	0	0	0	115	0	108	0	0	0	0	0	50	92	93	62	0	0	0	0	0	29	41	85	76	90	0
CTC_RS02170	426570..427448	46	108	76	26	52	118	11	54	30	87	37	0	16	33	24	15	29	43	39	0	54	0	42	0	9	25	26	78	0	87
mscL	427480..427881	34	59	0	0	114	129	23	157	0	15	0	0	0	0	0	0	0	63	11	0	60	0	0	101	99	28	0	68	0	48
CTC_RS02180	428020..428448	3843	5972	6944	5176	6759	6127	4947	8568	7938	4915	5845	12741	3819	6159	10781	12177	6469	9887	7696	7780	7249	3427	6786	9964	4326	6201	11862	6722	6579	10947
CTC_RS02185	428496..429125	1512	1600	3610	2035	3457	8112	2043	1299	3625	5808	1454	2046	1375	1057	1565	2233	721	2050	1319	1612	4139	1536	3021	9532	938	1455	2594	1740	1247	5766
hisE	429283..429606	0	0	82	0	71	585	0	0	330	36	50	0	0	0	194	0	0	117	0	0	222	38	0	377	0	0	143	0	152	0
CTC_RS14085	complement(429780..430268)	0	49	27	16	0	141	19	32	55	12	0	0	0	60	0	0	0	26	0	0	0	0	76	0	0	23	47	0	0	274
CTC_RS02200	430438..431733	177	110	236	238	159	133	173	243	206	214	186	184	295	192	129	155	127	107	149	75	166	311	173	94	165	121	215	72	66	163
CTC_RS02205	complement(431770..432396)	0	0	0	0	0	0	0	0	0	0	0	0	0	0	0	0	0	0	0	0	0	0	0	0	0	0	0	0	0	0
CTC_RS14090	complement(432380..433162)	0	0	0	0	0	0	0	0	0	0	0	0	0	0	0	0	0	0	0	0	0	0	0	0	0	0	0	0	0	0
CTC_RS02215	complement(433445..433831)	0	0	0	0	0	0	0	0	0	0	0	0	0	0	0	0	196	0	0	0	0	0	96	0	0	0	0	0	0	0
CTC_RS02220	complement(433988..434299)	0	152	43	49	294	773	0	101	171	133	232	0	0	0	67	0	243	81	20	0	0	40	120	261	0	107	149	66	0	307
CTC_RS02225	434468..435526	0	0	38	7	0	0	26	0	0	39	30	45	14	21	20	6	24	24	16	0	0	0	0	0	0	11	22	13	0	0
CTC_RS02230	complement(435552..436496)	29	25	42	24	24	274	99	33	57	125	51	25	15	62	22	7	0	54	22	51	152	53	138	43	42	35	25	51	52	81
CTC_RS02235	436737..437081	39	309	77	22	66	0	135	183	155	103	233	0	84	255	122	79	73	73	12	140	0	0	0	118	0	65	0	60	142	56
CTC_RS02240	complement(437125..438300)	0	20	1713	0	0	308	0	0	1136	15	14	487	0	106	571	0	21	4489	0	0	305	0	0	138	0	29	897	0	21	750
CTC_RS02245	complement(438293..438817)	0	542	31232	0	87	2101	0	510	21190	11	61	3773	0	725	15741	13	336	34354	8	92	2050	0	213	3490	0	873	15986	0	0	5093
CTC_RS02250	439016..439225	0	678	20581	0	0	4186	0	1499	8776	0	383	909	0	1882	6592	0	1321	12540	0	0	1025	0	0	1163	0	2608	6734	0	234	1643
CTC_RS02255	439279..439731	0	3665	39572	0	557	4243	0	4118	23350	13	426	3794	0	5106	21137	0	3620	28522	0	107	2060	27	1153	2067	0	7624	21905	0	1951	6982
CTC_RS02260	439901..440086	0	0	0	0	0	0	0	0	0	0	0	128	0	0	0	0	136	0	46	0	0	0	0	0	0	0	249	0	0	0
CTC_RS02265	440378..441748	30	121	116	95	167	189	20	103	58	185	0	17	21	64	61	50	55	254	146	0	105	122	27	89	23	61	51	160	90	252
CTC_RS02270	442029..442382	0	0	0	0	0	49	0	0	0	0	0	0	0	0	0	0	0	18	0	0	0	0	0	0	0	0	0	0	0	0
CTC_RS02275	complement(442635..443396)	0	0	35	10	30	68	0	41	0	23	63	94	9	29	0	0	0	17	11	0	0	16	0	0	0	0	0	18	0	25
CTC_RS02280	443558..444445	0	0	15	0	0	0	0	18	30	0	18	27	0	16	0	0	0	0	0	0	0	0	0	0	0	13	0	0	55	0
CTC_RS02285	444472..445572	0	65	0	14	0	16	0	14	24	11	58	0	13	53	19	0	69	11	0	44	0	11	0	0	0	51	0	6	45	0
CTC_RS02290	445553..446518	0	123	0	16	24	0	0	16	0	0	200	0	0	91	22	0	52	13	13	0	0	0	0	0	8	93	24	7	102	40
CTC_RS02295	complement(446581..447917)	0	0	0	0	0	0	0	0	0	0	0	0	0	0	0	0	0	0	0	0	0	0	0	0	0	0	0	0	0	0
feoB_1	complement(448062..450125)	0	34	274	4	11	67	5	69	168	17	51	81	3	113	61	0	98	322	0	0	46	30	108	99	12	49	180	0	178	251
CTC_RS02310	complement(450183..450422)	0	0	332	0	96	0	0	66	334	0	134	0	0	488	175	0	0	158	18	0	0	0	0	0	0	70	0	0	614	160
CTC_RS02315	450576..451043	0	0	0	0	0	0	0	34	0	0	172	0	0	63	45	0	0	0	9	0	0	0	0	0	0	24	0	0	0	41
CTC_RS02320	complement(451239..452057)	116	8616	8305	149	1553	1242	34	12454	5658	101	2012	1078	141	13844	7645	283	10181	4499	117	768	351	136	3030	746	126	13791	6596	193	15419	3394
CTC_RS02325	452281..453636	0	210	137	0	51	51	0	302	138	4	24	18	0	356	170	0	279	121	5	0	0	18	55	0	0	396	239	0	434	57
CTC_RS02330	453944..454513	0	0	23	67	0	0	16	276	164	83	28	126	13	26	37	84	0	289	37	0	252	44	65	214	28	98	163	24	129	370
CTC_RS14405	454543..455826	21	129	72	42	125	94	58	98	42	32	50	130	28	86	82	69	0	227	46	38	56	19	174	32	19	122	90	54	38	179
CTC_RS02340	455907..457601	199	420	224	248	379	488	215	362	362	305	313	380	115	242	223	205	179	471	71	57	226	128	176	336	89	218	233	205	420	599
CTC_RS02345	complement(457791..458288)	81	95	214	169	322	381	112	95	107	1488	97	144	174	29	42	164	51	483	555	194	384	25	150	327	111	157	93	656	49	289
CTC_RS02350	458458..459504	26	23	102	47	131	214	45	45	51	90	138	137	55	28	20	52	72	351	18	231	411	24	71	78	23	32	0	46	94	238
CTC_RS02355	459770..463378	11870	14203	7771	23184	15850	8535	14469	12010	7479	5765	4287	4170	22172	11467	6027	15441	14218	13063	7147	8531	8730	27208	13873	7580	20139	11198	6777	7611	13649	16746
CTC_RS14635	463508..463672	164	431	242	1156	556	627	170	286	162	0	0	0	569	355	127	0	0	0	0	0	0	790	226	493	865	203	281	83	298	232
CTC_RS02360	463792..464136	9205	13579	5088	20991	13022	4397	12439	12319	3910	0	0	0	21151	10778	6232	0	0	0	0	0	0	23449	10818	3186	19354	11242	5544	5171	14300	10112
CTC_RS14640	464179..468183	9617	19616	9173	20488	18962	11308	10728	13424	8270	204	421	283	16964	13144	7924	0	0	0	0	0	0	22805	20744	8666	16291	12888	9685	7197	16188	14859
CTC_RS02370	469186..469884	1489	3258	2131	4340	4001	3193	2075	2646	1414	0	0	0	3402	2304	1650	3298	6118	4284	0	0	0	3497	4485	2388	3199	2878	1692	1982	4389	4127
CTC_RS14095	470110..470967	1236	2087	1441	1690	1763	1808	1147	1835	1774	0	0	0	1657	1723	1051	0	0	0	107	0	139	1981	1653	1518	1724	1544	1000	1715	1402	2457
CTC_RS02380	471902..473884	416	72	248	158	173	235	282	79	162	710	195	156	277	81	95	0	0	0	101	0	169	144	113	308	388	113	199	922	322	309
CTC_RS02385	474168..474449	1605	841	731	1218	691	917	1786	698	758	3278	912	592	2124	441	781	713	582	382	400	429	170	1056	794	1949	2418	436	1069	1390	1088	1122
CTC_RS02390	474881..476809	189	2109	700	1048	1693	773	46	2766	2423	591	292	235	103	2003	2120	590	889	1785	462	527	1234	556	1432	718	49	2158	1683	887	1947	4581
CTC_RS02395	477101..477976	123	460	509	183	628	1131	106	198	213	506	367	341	33	159	120	284	346	867	63	166	546	71	341	418	54	102	26	180	140	853
CTC_RS02400	complement(478094..478519)	16	223	1217	0	161	202	0	370	627	28	151	168	0	309	295	16	355	743	0	114	56	0	263	96	0	472	109	0	374	225
CTC_RS02405	478783..480489	32	0	23	18	40	0	11	18	31	69	28	42	25	0	25	36	30	96	65	28	42	0	22	72	5	7	27	77	14	34
CTC_RS02410	complement(480571..481875)	0	82	61	35	334	132	7	60	61	5	160	110	6	22	16	5	58	0	7	297	73	10	172	31	0	77	36	0	38	73
CTC_RS02415	complement(481986..483125)	196	2206	752	643	5550	665	454	1471	633	5	2566	921	272	2825	478	358	487	56	216	5219	630	490	4191	357	334	1608	447	6	1658	1160
CTC_RS02420	complement(483159..484706)	105	1647	816	345	4738	568	199	1103	587	29	2367	1187	214	2047	556	202	277	131	122	4859	556	353	3098	263	195	1469	494	9	1094	966
CTC_RS02425	complement(484749..485525)	87	1557	582	260	3643	421	276	1155	344	15	1696	829	121	2054	297	158	260	147	150	4856	400	144	2546	262	102	1446	328	9	1011	814
CTC_RS02430	485711..486139	63	111	93	196	53	121	130	183	311	69	581	1001	84	102	293	64	588	30	69	564	223	116	261	427	111	261	324	16	515	45
CTC_RS02435	486492..487301	33	0	49	66	141	128	138	136	0	168	0	29	9	36	0	17	0	47	47	0	30	31	0	151	29	41	29	195	0	24
CTC_RS02440	487490..488803	1172	812	800	4138	733	669	2013	527	589	166	1768	563	3323	323	686	3087	538	154	2037	2098	674	3914	682	589	2891	519	547	120	1046	1072
CTC_RS02445	488995..489450	119	208	233	100	201	76	20	104	176	330	70	105	32	64	138	119	55	83	172	0	0	54	327	357	35	123	305	271	54	378
CTC_RS02450	489447..490760	165	217	132	308	174	105	227	240	183	427	73	200	198	245	239	373	134	135	466	37	36	321	170	62	241	179	141	173	37	467
CTC_RS02455	490778..491491	170	365	130	438	417	266	248	198	299	835	203	234	303	267	235	410	71	195	512	271	268	417	157	228	255	235	227	308	241	752
CTC_RS02460	491488..492702	133	195	109	333	208	85	200	162	132	355	132	138	255	133	86	387	104	146	290	119	59	214	246	67	202	184	172	198	222	174
CTC_RS14645	492721..492858	0	0	0	55	0	0	0	0	0	0	0	0	0	0	0	49	0	0	31	0	0	0	0	0	57	0	0	0	0	0
CTC_RS02465	492846..493982	297	292	199	477	181	197	226	125	117	608	42	126	336	219	148	309	22	234	385	85	189	295	98	215	251	202	163	239	227	354
CTC_RS02470	493995..495191	147	99	167	440	191	302	164	342	112	844	255	249	187	147	245	307	169	201	465	202	120	384	125	204	272	224	58	359	205	432
CTC_RS02475	495298..496122	49	29	81	74	0	63	79	19	130	0	0	0	61	35	51	0	0	0	0	0	0	30	90	0	67	68	28	158	30	46
CTC_RS02480	496243..497769	168	93	148	70	135	169	220	175	52	101	126	219	173	134	110	103	149	124	366	285	251	24	73	160	93	81	167	131	48	138
CTC_RS02485	497989..500916	74	89	150	86	180	153	41	113	82	131	88	330	47	80	64	65	129	147	86	198	294	47	76	167	33	52	63	61	184	216
CTC_RS02490	501017..503086	7	46	26	15	33	17	14	30	13	37	62	81	10	7	30	10	12	31	14	0	58	18	54	39	23	16	11	3	0	65
CTC_RS02495	503151..505238	45	68	57	44	126	116	40	75	90	107	85	126	59	42	60	42	60	97	31	70	126	30	71	117	34	51	67	49	82	138
CTC_RS02500	complement(505633..506556)	29	0	0	0	0	0	10	0	0	6	0	26	0	0	0	0	0	0	5	0	0	0	0	44	0	0	0	0	0	41
CTC_RS02505	complement(506670..507542)	186	245	274	61	184	138	96	180	245	169	129	465	91	101	168	94	144	232	24	111	274	71	342	233	64	51	186	83	169	373
CTC_RS02510	complement(507557..508489)	14	25	86	8	74	185	20	34	115	51	52	128	8	16	67	22	81	68	14	52	103	40	40	218	25	0	25	7	0	123
CTC_RS02515	508728..509165	370	596	304	383	366	354	277	467	427	930	330	436	198	267	575	513	576	347	417	663	328	397	511	372	253	268	635	439	392	263
CTC_RS02520	509324..509797	0	0	0	16	0	0	0	0	0	12	0	0	0	0	0	0	0	0	0	0	0	0	157	0	0	0	0	0	0	0
CTC_RS14100	510000..511778	30	67	75	69	77	126	94	88	135	56	54	161	105	91	177	126	71	114	50	27	128	91	63	137	22	75	39	31	28	11
CTC_RS02530	complement(511884..512651)	35	0	35	30	0	0	12	20	0	215	21	0	9	0	0	0	0	99	39	0	47	16	0	0	0	0	0	0	96	100
CTC_RS02535	512896..514050	164	986	633	1810	1151	910	178	1036	46	169	153	41	137	482	109	366	2446	1184	456	1424	1533	1214	905	775	62	435	221	452	3071	2780
CTC_RS02540	514113..514958	144	1178	440	1660	1192	632	353	558	95	140	209	85	60	381	50	306	3400	1227	482	1715	1060	579	1632	626	75	495	110	309	3090	1949
iolC	514978..515997	146	419	352	1111	629	355	293	432	26	139	221	47	134	244	123	170	4415	881	233	2276	915	669	768	399	101	471	68	229	2719	1447

CTC_RS02550	516004..516792	308	301	253	1016	349	218	236	279	203	37	41	121	91	186	133	125	4924	265	38	1471	910	393	189	464	90	156	118	131	3033	911
iolD	516810..518744	140	196	172	325	107	169	116	138	97	6	42	86	104	45	98	74	3012	186	35	1187	464	189	19	231	139	69	72	43	2328	624
iolG	518789..519802	147	94	236	489	181	476	166	326	184	12	135	141	121	72	269	67	5275	250	50	2147	920	184	110	241	125	77	160	14	3607	718
iolE	519834..520730	60	53	208	213	153	307	125	193	149	13	72	53	129	114	140	38	3966	71	55	2642	587	97	83	182	124	75	26	23	2545	812
CTC_RS02570	520763..521788	145	208	194	260	223	302	136	123	130	23	110	140	70	128	20	53	3590	271	54	2216	746	85	327	397	100	109	23	0	2452	859
CTC_RS02575	521977..523569	17	0	25	43	29	119	12	30	17	0	20	15	9	9	0	4	697	32	5	334	60	23	23	102	10	7	15	9	1826	144
CTC_RS14105	523777..525531	146	135	106	117	91	88	229	152	152	50	73	109	185	92	203	58	86	36	56	110	41	141	43	139	145	102	66	43	406	164
CTC_RS02585	complement(525598..526934)	0	0	0	0	0	0	0	0	0	0	0	0	0	0	0	0	0	0	0	0	0	0	0	0	0	0	0	0	0	0
CTC_RS14110	527266..529023	46	229	182	35	339	118	16	269	198	10	366	176	21	300	203	58	129	173	29	193	395	49	149	185	9	251	185	16	126	262
CTC_RS02600	529141..530802	1049	2448	2372	1228	2386	1934	1574	1809	2644	345	4637	2829	1225	1885	2310	2723	2155	2278	1112	2649	2670	1034	1954	1298	1217	1621	2371	219	1093	1880
CTC_RS14115	531085..533700	108	36	112	76	88	145	114	42	31	280	86	109	166	6	96	158	159	160	124	18	146	123	14	124	97	38	106	181	28	132
CTC_RS02610	533741..535180	169	148	120	117	96	263	259	109	111	326	179	348	226	142	102	199	350	185	151	101	166	224	104	396	204	109	225	267	290	213
CTC_RS02615	535711..536772	445	1273	1052	525	1306	1185	624	1682	905	234	832	1393	727	1185	1205	988	1544	1228	597	2186	1059	596	1476	1418	568	968	971	298	878	1128
CTC_RS02620	536938..537777	467	1468	934	809	1228	1867	478	881	986	826	1598	2415	791	1098	949	811	1532	1387	785	1497	2364	812	1511	2157	699	945	718	540	935	1038
CTC_RS02625	538139..538690	24	0	0	83	125	31	17	0	0	11	87	130	0	0	0	12	0	69	15	0	43	0	0	0	29	40	0	25	0	174
CTC_RS02630	538767..539180	33	143	0	0	0	0	0	0	65	14	0	0	17	35	0	16	0	0	10	0	0	0	0	0	0	81	0	33	59	0
CTC_RS02635	complement(539295..539624)	0	288	403	23	208	470	28	215	81	1092	1364	1410	0	89	64	21	0	115	13	0	217	75	226	0	0	34	562	0	74	639
CTC_RS02640	539779..540177	0	0	33	0	0	0	23	0	0	0	0	0	0	0	53	17	0	0	0	0	0	31	94	102	0	0	0	34	0	0
CTC_RS02645	540426..541484	89	67	163	108	65	260	70	74	101	563	152	90	55	28	59	158	71	60	181	91	181	70	176	250	112	95	66	234	116	199
CTC_RS02650	541640..542677	39	0	0	15	22	100	9	15	26	80	31	0	0	14	0	7	0	37	14	93	46	0	36	78	0	11	0	26	0	37
CTC_RS02655	complement(542772..544502)	8	0	31	0	13	0	5	0	15	7	9	14	0	0	12	4	0	15	2	0	14	0	0	0	0	0	0	0	0	33
CTC_RS02660	544754..545746	14	0	0	23	23	17	19	0	0	36	0	24	18	0	0	21	0	51	17	49	0	0	0	0	0	11	0	7	25	39
CTC_RS14650	546126..546293	0	141	79	45	0	103	0	187	318	0	383	0	0	261	250	0	150	75	0	0	0	0	222	0	0	133	138	0	438	0
CTC_RS02665	546664..547968	72	164	173	94	211	423	164	96	225	296	234	402	88	79	241	318	406	272	220	37	275	48	172	624	128	128	249	200	169	206
CTC_RS02670	547972..549240	96	19	115	96	181	326	70	62	42	265	89	169	46	12	33	177	139	120	141	38	151	20	29	353	44	35	55	171	39	136
clpB	549604..552201	10	173	118	41	185	206	61	158	293	11	105	83	42	68	113	29	58	146	19	19	83	67	129	282	34	82	98	24	19	96
CTC_RS02680	552382..553179	0	0	0	0	0	22	0	20	0	0	0	0	0	46	0	0	126	32	0	0	0	0	0	0	0	28	0	0	31	0
CTC_RS14925	complement(553262..554478)	100	97	66	31	188	354	38	13	66	291	106	59	18	60	34	56	104	52	49	40	138	51	92	234	13	37	0	133	40	126
CTC_RS02690	complement(555169..555900)	120	162	73	73	188	165	76	32	73	153	22	98	39	20	0	84	69	52	55	198	98	17	0	222	11	31	32	113	34	52
CTC_RS02695	complement(555919..557289)	20	69	39	50	92	88	20	29	0	108	35	0	5	0	31	45	0	18	16	0	44	9	27	178	17	8	0	60	0	28
CTC_RS02700	complement(557324..558475)	23	62	23	13	60	30	49	27	0	144	0	21	6	13	18	41	0	66	22	0	125	0	32	18	34	19	0	60	0	67
CTC_RS02705	558694..560451	8	135	106	30	39	157	48	143	76	104	210	136	29	208	131	50	144	144	73	55	150	14	42	139	41	254	92	8	98	44
CTC_RS02710	560613..561758	12	0	35	20	0	90	24	27	23	31	0	21	6	0	18	6	0	44	33	84	42	0	33	0	35	0	20	36	0	0
CTC_RS02715	561864..562679	33	87	33	75	56	84	149	77	131	195	158	29	71	72	77	83	93	62	130	237	117	46	183	50	49	55	28	126	30	141
CTC_RS02720	562775..563215	0	161	302	0	52	117	0	732	303	0	292	0	0	1328	618	0	1316	230	0	0	54	0	254	0	0	710	421	0	668	304
CTC_RS02725	563306..563947	0	517	849	0	143	295	0	907	333	9	901	223	0	1152	441	0	1847	680	7	0	112	0	233	63	0	1167	614	0	1376	269
CTC_RS02730	564030..564518	0	3129	23292	0	797	4653	0	3283	13492	0	1693	3417	0	3503	8729	14	4385	22940	0	0	1614	0	992	3080	0	3783	8819	0	3965	6154
CTC_RS02765	complement(570458..571333)	0	0	30	0	0	0	0	0	0	0	0	0	0	0	0	0	0	0	0	0	0	0	0	0	0	0	0	0	0	0
CTC_RS02770	571590..572303	114	199	74	118	128	169	170	88	75	223	113	201	121	103	323	114	71	213	277	68	101	104	52	57	78	250	162	221	34	161
CTC_RS02775	complement(572399..572722)	0	0	41	0	0	0	0	0	0	0	0	0	0	0	0	0	78	0	0	0	0	0	0	0	0	0	0	0	0	0
CTC_RS02780	572773..573228	0	52	29	92	101	151	41	104	0	39	35	0	47	32	0	15	55	28	56	0	262	27	0	89	0	49	0	30	0	84
CTC_RS02785	573332..573847	79	460	515	133	1022	902	90	854	362	378	1293	1041	182	624	610	185	1125	1423	301	656	881	385	796	631	46	585	719	187	856	483
CTC_RS02790	573837..574796	282	1198	3241	676	2018	3385	544	1541	2323	812	2905	3394	414	1426	2469	433	2838	4003	421	1990	4012	607	1905	2756	281	1030	1860	716	2506	3235
CTC_RS14930	complement(575075..575767)	0	0	19	22	66	0	40	0	0	68	46	103	0	0	0	34	0	55	49	0	0	0	54	59	23	16	33	60	0	166
CTC_RS02800	576911..577729	0	0	0	9	0	0	0	0	0	325	10	0	0	0	0	0	31	15	0	0	0	45	0	50	0	0	0	50	0	0
CTC_RS02805	577754..578569	0	0	0	0	28	0	0	0	0	586	0	0	0	0	26	17	0	16	10	0	0	0	0	0	5	0	0	227	0	23
CTC_RS02810	578640..579290	21	36	41	0	0	26	0	0	0	327	0	73	22	0	0	0	39	19	0	0	0	0	0	0	0	17	0	32	0	0
CTC_RS02815	579332..580063	0	0	0	21	0	0	0	0	0	226	0	65	10	0	0	0	69	17	0	0	0	17	0	0	11	0	32	19	0	0
CTC_RS02820	580132..581328	11	40	0	6	0	14	0	0	0	222	13	40	18	12	0	6	0	0	11	0	40	0	0	0	7	0	19	60	0	0
argH	581425..582741	0	0	10	0	0	0	0	0	0	72	0	0	0	0	0	0	0	10	3	0	18	0	0	0	7	8	0	26	0	0
CTC_RS02830	583228..584580	2377	123	216	220	152	548	376	58	39	402	416	370	400	32	62	146	503	75	317	1216	884	404	110	752	322	50	86	544	272	99
glsA	584873..585790	88	78	101	33	150	244	20	17	58	39	193	104	24	16	160	7	220	28	28	474	339	54	0	89	69	85	152	127	0	63
CTC_RS14655	586068..586259	0	0	0	0	0	0	0	0	0	62	0	0	38	0	0	71	0	66	66	0	125	0	0	212	0	0	0	36	0	0
CTC_RS02840	586174..587934	77	94	151	87	156	147	21	54	91	389	192	230	33	100	119	217	86	144	377	302	231	28	127	23	27	83	145	252	84	131
CTC_RS02845	complement(588005..588478)	114	50	28	32	97	73	39	0	113	162	0	50	61	0	133	72	53	53	152	0	50	0	0	344	117	24	49	102	0	81
CTC_RS02850	588624..588965	0	0	0	112	67	50	0	46	0	52	47	0	21	43	61	159	0	74	87	141	0	36	0	0	70	0	0	40	0	0
thrC_1	589152..590360	140	137	22	126	114	114	89	117	22	64	106	39	245	97	139	242	292	209	118	120	139	185	216	101	164	157	115	114	183	111
CTC_RS02860	590475..591416	100	176	141	130	243	91	99	67	85	94	154	228	69	93	89	188	107	148	113	0	152	40	238	43	67	154	25	66	52	163
CTC_RS02865	591492..592298	84	147	157	38	28	171	104	137	66	29	279	177	80	145	182	101	156	78	119	60	119	23	46	151	39	83	57	34	0	119
CTC_RS02870	592355..592876	414	863	484	460	527	528	518	799	1177	102	1108	663	1120	953	924	744	1160	728	717	927	733	737	572	702	684	792	1377	369	658	386
CTC_RS02875	complement(592947..593177)	0	0	0	0	0	0	0	0	0	26	139	0	0	0	0	0	0	55	0	0	0	0	0	0	0	145	0	30	0	83
CTC_RS02880	complement(593351..596494)	64	113	38	137	131	315	30	75	85	1762	120	68	80	79	87	243	40	151	297	77	251	142	83	168	73	85	129	577	23	79
CTC_RS02885	596869..597276	66	0	0	0	112	84	160	0	0	72	39	0	35	36	51	17	0	93	94	0	59	91	183	0	117	110	0	67	0	47
CTC_RS02890	597458..598171	76	0	74	0	161	48	52	66	37	232	135	100	0	41	29	67	35	18	113	0	168	35	52	114	33	47	65	39	34	0
rihC	598200..599120	176	129	159	207	174	187	162	120	174	327	384	441	188	79	137	244	219	275	332	53	260	108	162	133	172	243	277	269	293	167
CTC_RS02900	599240..600415	11	20	0	0	0	29	48	13	0	25	14	101	12	12	18	17	0	32	11	0	0	0	63	0	7	19	20	12	0	33
CTC_RS02905	600416..603553	22	53	51	36	37	36	30	55	34	34	41	61	21	33	33	50	32	61	18	31	38	20	36	26	8	43	15	28	23	73
CTC_RS02910	603668..605254	94	224	117	168	260	163	91	179	84	368	142	196	111	120	93	193	143	180	212	91	166	149	94	103	65	155	73	206	124	326
CTC_RS02915	complement(605332..606510)	229	1247	2448	550	1594	3253	135	774	1835	35	3558	2085	12	807	676	462	1926	698	252	2318	3490	421	1646	1588	0	398	806	35	760	1683
CTC_RS02920	complement(606488..607696)	257	1560	2134	802	1517	2573	185	879	1237	44	3583	2527	30	908	885	772	2462	1047	355	3101	3186	862	1266	926	13	564	710	34	792	1411
CTC_RS02925	complement(607768..609333)	190	757	985	412	1156	1893	60	493	1058	38	2309	777	14	327	442	331	878	453	100	1514	1604	301	1120	1105	5	228	415	22	486	832
CTC_RS02930	609771..610280	26	47	0	0	45	34	18	93	0	93	32	47	28	0	0	13	0	99	42	0	47	24	0	0	31	22	0	0	0	56
CTC_RS02935	610381..611016	42	75	230	72	180	596	88	124	210	223	556	600	113	161	165	86	278	338	160	76	414	59	176	320	62	158	109	119	154	392
CTC_RS02940	611054..611602	49	130	97	56	251	283	85	143	0	22	117	304	79	107	38	74	460	184	151	88	218	45	136	222	87	163	42	88	313	175
CTC_RS02945	complement(611669..613225)	35	46	34	44	44	133	0	71	34	197	83	61	28	85	13	52	16	16	98	124	184	40	24	78	20	50	60	84	63	86
CTC_RS02950	613424..614062	0	0	0	12	0	0	15	74	0	28	0	75	11	23	0	21	0	40	27	0	0	19	0	0	12	35	0	0	0	30
CTC_RS02955	complement(614184..614645)	0	513	892	0	50	112	20	1158	983	26	452	155	0	1172	590	0	710	397	18	209	155	0	242	88	0	1331	753	15	1169	332
CTC_RS02960	complement(614765..615895)	24	0	82	13	41	76	8	14	47	52	43	21	6	13	0	18	89	22	4	0	63	0	99	108	0	10	20	24	43	17
CTC_RS02965	complement(615934..617346)	10	101	94	11	65	244	0	45	113	38	23	68	15	0	15	10	125	99	39	34	119	26	211	86	6	16	33	24	17	95
CTC_RS02970	complement(617375..617863)	0	0	82	0	0	247	0	32	55	24	0	0	0	0	0	0	0	26	0	0	0	0	0	250	0	0	0	14	0	0

CTC_RS02975	complement(617877..618686)	0	0	0	19	0	43	12	19	0	7	0	0	0	0	0	8	0	0	0	0	0	0	0	0	0	0	0	0	0	0
CTC_RS02980	619004..620275	0	298	450	0	108	27	0	792	231	0	329	56	0	817	330	5	1289	328	3	38	19	0	88	64	0	914	292	11	1351	211
CTC_RS02985	complement(620315..621415)	25	43	24	14	42	0	0	86	24	32	88	22	20	133	19	6	46	80	0	0	0	0	34	0	0	112	84	6	45	87
CTC_RS02990	621634..622338	0	0	0	0	65	0	0	45	0	8	0	0	0	0	0	10	0	0	0	0	0	0	0	0	11	0	0	0	35	0
CTC_RS02995	622335..623777	0	0	9	11	16	24	0	0	19	4	11	0	0	10	0	0	17	0	0	0	0	0	0	0	0	0	0	0	17	0
CTC_RS03000	623774..624880	12	43	24	21	21	31	4	128	24	5	44	54	39	145	19	37	91	80	23	44	151	22	34	0	7	71	42	12	89	17
CTC_RS03005	complement(625498..625818)	0	0	0	0	0	0	0	0	0	92	50	0	0	0	0	0	0	0	40	0	75	0	0	0	0	0	0	21	0	0
CTC_RS03010	626067..626483	0	0	0	0	0	0	0	0	0	14	0	0	0	0	0	0	0	0	10	0	0	0	0	0	0	0	0	0	0	92
CTC_RS03015	626699..628057	0	35	10	17	8	25	0	46	20	143	0	18	16	11	0	65	0	28	25	0	18	9	0	0	0	33	0	96	0	56
CTC_RS03020	complement(628116..630104)	34	60	94	88	127	147	84	150	107	178	65	48	33	81	11	82	63	102	36	0	108	44	113	41	32	34	35	47	62	135
CTC_RS03025	630397..630834	0	5605	972	0	1256	275	0	2480	2866	0	8734	1035	0	10027	2107	0	2765	434	0	442	492	0	511	372	0	7592	1958	0	1401	1422
CTC_RS03030	630891..631295	0	2665	328	0	792	85	0	1710	1055	0	4941	413	0	6380	880	0	1184	125	0	239	118	0	184	0	0	4071	1259	0	364	521
CTC_RS03035	complement(631339..632775)	0	0	0	0	0	24	0	0	0	4	34	17	0	0	7	0	0	0	0	0	8	9	0	0	0	0	0	5	34	27
CTC_RS03040	complement(632753..633292)	0	0	0	0	0	0	0	0	0	11	0	0	0	0	0	0	0	0	0	0	0	0	0	0	0	0	0	13	0	0
CTC_RS03045	633447..635615	0	22	18	4	21	16	9	7	12	0	7	0	10	13	0	16	12	35	14	0	33	6	17	0	7	5	0	3	11	0
CTC_RS03050	635981..636316	0	0	0	0	0	0	0	0	0	0	0	0	0	0	0	0	0	0	51	0	0	0	222	121	0	0	0	0	0	0
CTC_RS03055	636618..636824	0	0	0	37	0	0	0	0	0	0	0	0	0	0	0	0	0	0	21	0	0	0	0	0	0	0	0	0	0	0
CTC_RS03060	636817..637098	96	0	94	0	0	611	0	0	0	0	0	0	0	0	0	0	0	0	0	172	424	0	265	144	0	0	0	0	0	0
CTC_RS03065	complement(637244..638149)	1805	7750	15595	3323	7679	8181	3872	8131	28302	65	25804	2490	3509	16190	25513	5571	16652	5450	713	5099	2733	2753	7456	8717	3422	11548	27227	106	12109	10230
CTC_RS03070	638579..639160	0	0	0	26	79	237	0	54	46	0	28	82	12	25	0	0	43	22	0	0	0	0	0	70	0	19	40	12	42	66
CTC_RS03075	639228..639869	589	240	228	226	464	349	777	245	83	336	150	260	416	160	261	271	432	316	258	603	224	271	465	317	494	192	72	161	76	90
CTC_RS03080	complement(640106..640876)	245	154	78	178	0	0	423	122	173	414	313	124	393	152	245	168	164	82	127	0	62	113	0	0	267	261	150	339	127	75
CTC_RS03085	complement(640869..641351)	196	49	0	253	47	71	406	98	55	660	299	99	605	182	130	324	209	26	172	0	0	231	0	0	378	116	192	285	0	79
CTC_RS03090	641529..641876	0	0	0	0	0	50	0	0	0	0	0	0	0	0	0	0	0	0	0	0	0	0	0	0	0	0	0	40	0	0
CTC_RS03095	641866..642591	0	0	0	0	0	0	0	0	0	24	0	0	0	0	0	28	0	0	6	0	0	0	0	0	0	0	0	9	0	26
tnpA_2	642709..643107	102	0	33	0	57	497	0	0	67	0	0	0	0	0	53	0	0	0	0	0	0	31	47	0	0	0	0	0	0	0
tnpB_2	643117..644205	0	0	0	0	0	0	0	0	0	0	0	0	0	0	0	0	0	0	4	0	0	0	0	0	0	0	0	0	0	0
CTC_RS03110	644449..645153	0	34	19	0	65	73	0	0	76	8	0	34	10	21	30	19	0	0	0	0	0	0	0	0	0	95	33	0	104	381
CTC_RS03115	645156..645905	18	0	0	0	0	23	0	0	0	8	0	0	10	20	0	18	0	17	0	0	0	0	0	0	0	0	0	0	98	102
CTC_RS03120	645905..646681	0	0	0	10	0	22	0	20	0	0	0	0	9	19	0	0	0	16	0	0	0	0	0	52	0	0	0	0	32	74
CTC_RS03125	646738..647400	20	72	0	12	35	78	42	47	0	36	48	72	44	22	0	10	0	76	6	0	0	19	0	0	12	0	0	0	74	87
CTC_RS03130	647388..648752	0	0	29	11	0	13	7	23	0	26	24	35	5	11	15	0	0	9	6	0	53	9	0	0	6	8	17	10	36	14
CTC_RS03135	649712..650677	0	0	0	0	0	0	0	0	0	0	0	0	0	0	0	0	0	0	0	0	0	0	0	0	0	0	0	0	0	0
CTC_RS03140	650846..651709	0	0	0	0	0	0	0	0	0	0	0	0	0	0	0	0	0	0	0	0	0	0	0	0	0	0	0	0	0	0
CTC_RS14935	651660..651782	0	0	0	0	0	0	0	0	0	0	0	0	0	0	0	0	0	0	0	0	0	0	0	0	0	0	0	0	0	0
CTC_RS14940	651905..652560	0	72	0	47	35	0	0	48	0	0	0	0	11	45	0	21	38	19	10	0	0	0	0	0	12	0	0	0	0	0
CTC_RS03150	652710..653498	51	90	51	116	58	109	83	120	68	0	0	0	101	0	53	190	0	160	0	0	0	94	47	0	80	128	176	0	0	0
CTC_RS03155	653501..655198	64	84	16	81	81	41	33	0	16	0	0	0	43	0	37	84	15	82	0	0	0	29	0	24	33	33	0	0	0	0
CTC_RS03160	complement(655367..655930)	0	0	0	0	0	31	0	0	0	0	0	0	0	0	37	0	0	0	0	0	0	0	0	144	0	0	0	0	0	0
CTC_RS03165	656403..656660	0	0	103	59	89	134	72	0	104	0	0	0	0	0	0	0	0	0	0	0	0	0	0	158	0	43	0	0	0	0
CTC_RS03170	656753..657259	0	47	26	30	45	68	0	0	105	0	0	0	0	29	0	0	0	0	0	0	0	73	0	80	0	22	0	0	0	0
CTC_RS03175	657336..657650	0	0	0	0	0	55	0	0	0	0	0	0	0	0	0	0	0	0	0	0	0	0	0	0	0	71	0	0	0	0
CTC_RS03180	657839..658228	0	0	102	0	0	265	0	0	0	0	0	0	19	0	0	0	0	0	0	0	0	0	191	0	0	0	0	0	0	0
CTC_RS03185	658393..659250	16	28	0	53	0	80	43	18	31	0	0	0	0	17	24	0	0	0	942	1353	1422	72	0	95	37	13	81	0	0	0
CTC_RS03190	659923..660408	222	195	192	79	283	248	58	0	55	0	0	0	163	181	345	0	0	0	0	0	0	26	384	84	65	207	95	0	0	0
CTC_RS03195	660603..661634	26	115	206	96	289	301	18	92	26	63	265	278	28	128	142	7	24	25	49	0	174	48	108	118	31	108	270	13	24	93
CTC_RS03200	complement(662071..662277)	0	0	0	0	0	0	0	0	0	0	0	0	0	0	0	0	0	0	0	0	0	0	0	0	0	0	0	0	0	0
CTC_RS03205	662958..663476	208	46	512	132	265	731	180	182	206	171	434	184	153	169	364	79	243	342	82	373	622	120	431	471	61	129	357	46	47	74
CTC_RS03210	complement(663554..664051)	0	0	27	31	23	35	19	32	161	107	97	0	0	29	126	0	0	0	26	0	96	0	0	0	32	0	0	0	0	0
CTC_RS03215	complement(664600..666450)	0	0	14	12	37	84	10	26	43	13	9	26	8	24	0	7	14	27	11	26	116	13	40	0	2	6	38	7	13	78
CTC_RS03220	complement(666465..667169)	0	0	0	11	33	0	0	0	0	25	46	0	0	0	0	0	0	18	6	0	34	0	0	0	0	16	0	20	0	27
CTC_RS03225	complement(667209..668099)	15	27	75	17	51	116	31	0	60	80	144	134	16	0	0	0	28	28	19	0	134	0	42	137	0	38	52	0	55	65
CTC_RS03230	complement(668439..669101)	0	0	0	0	0	0	0	0	0	0	0	0	0	0	0	51	152	115	0	0	0	0	0	0	0	0	0	10	222	87
CTC_RS03235	complement(669287..669820)	25	44	50	0	0	97	70	0	50	33	60	0	14	110	79	0	0	0	24	91	90	23	0	76	30	0	0	0	0	0
CTC_RS03240	complement(669841..670236)	0	0	34	0	0	44	0	0	0	30	41	0	18	0	0	0	64	0	32	0	0	31	0	0	0	0	0	0	0	0
CTC_RS03245	670355..671200	48	56	110	108	163	122	22	112	63	56	114	85	51	35	74	93	0	75	111	172	141	59	221	0	56	66	110	41	15	23
CTC_RS03250	671322..671903	0	41	46	0	0	0	16	0	0	41	55	82	12	25	0	12	0	0	29	0	82	0	64	70	14	19	0	12	0	33
CTC_RS03255	672058..672957	15	26	59	8	25	38	10	17	30	0	18	27	40	33	47	0	56	0	9	54	27	14	0	45	0	75	0	0	27	43
CTC_RS03260	673015..673713	77	0	19	44	66	173	67	23	0	8	92	0	41	84	30	0	108	36	12	0	0	18	107	58	0	112	66	10	70	27
CTC_RS03265	673859..676351	16	152	112	70	166	270	37	51	75	31	39	29	25	59	42	11	10	30	5	19	58	25	90	196	19	94	37	8	0	15
CTC_RS03270	complement(676395..676709)	43	0	42	48	0	55	0	0	170	75	51	76	69	139	67	86	160	0	40	0	76	0	0	0	0	35	147	0	0	0
CTC_RS14945	676871..677131	0	0	0	0	0	0	0	0	0	0	0	0	0	0	0	0	0	49	0	0	0	0	0	0	0	0	0	0	94	0
CTC_RS03280	complement(677374..679029)	0	158	24	0	14	10	0	38	0	4	204	58	0	124	25	4	53	31	3	29	29	0	23	25	0	101	28	8	74	93
CTC_RS03285	679185..679718	0	0	0	43	43	97	17	29	0	44	60	0	0	0	39	26	47	24	16	0	0	0	0	0	0	21	0	0	0	0
CTC_RS03290	679724..680254	0	0	0	57	0	32	53	0	0	67	0	0	0	0	0	13	0	48	32	0	0	23	0	0	0	0	0	13	0	36
CTC_RS03295	680489..683152	30	62	65	14	52	13	4	165	0	47	54	27	8	115	55	15	95	105	14	18	27	14	28	15	3	113	35	13	92	58
CTC_RS03300	683258..683854	23	0	45	0	38	87	0	0	0	0	27	280	0	0	351	11	169	636	7	0	280	0	0	0	13	37	311	0	0	96
CTC_RS03305	683847..684146	45	0	89	51	0	172	31	52	89	20	0	0	0	0	210	0	84	633	0	0	319	0	0	0	0	0	0	23	0	0
CTC_RS03310	684158..684763	67	157	724	126	416	1508	46	78	837	58	637	689	24	145	1384	22	1499	4179	28	0	1342	41	62	1108	39	166	1416	11	81	95
CTC_RS03315	684841..685269	32	387	837	160	374	643	43	349	560	413	412	1057	135	307	1027	286	1059	2464	277	338	1283	0	348	854	129	287	1135	417	343	491
CTC_RS03320	complement(685315..685761)	0	53	178	17	103	39	0	35	0	370	36	107	0	0	0	76	0	28	95	0	0	0	0	91	18	0	207	169	0	214
CTC_RS03325	complement(685763..686593)	16	86	16	9	55	62	22	57	0	249	39	29	0	53	0	156	0	15	82	58	144	0	45	0	0	13	28	74	59	92
CTC_RS03330	complement(686590..687915)	20	54	60	23	86	52	106	12	40	187	36	108	0	22	79	175	133	124	189	36	126	9	56	123	30	25	35	99	74	101
ribD	688611..689678	25	22	25	0	0	48	35	44	0	28	45	112	7	14	0	38	83	59	36	91	134	0	35	0	30	10	22	6	115	27
ribE_1	689707..690357	0	55	20	0	0	0	14	0	0	0	49	37	11	22	0	31	194	19	26	0	37	0	57	0	0	52	36	21	75	59
CTC_RS03345	690719..691918	11	79	33	25	19	72	16	92	22	44	80	219	30	49	52	17	63	42	32	202	140	31	62	34	0	65	19	29	123	80
ribE_2	691944..692405	0	0	58	17	50	0	20	68	0	13	0	52	47	32	45	15	109	55	9	314	0	54	0	0	34	0	100	0	0	41
CTC_RS14135	692724..693743	132	256	156	135	135	169	174	170	288	87	347	398	290	172	329	377	371	223	317	522	164	158	73	160	241	175	205	364	337	470
CTC_RS03360	693837..694232	34	0	67	19	116	218	71	79	202	112	0	60	36	0	0	17	127	0	21	122	181	63	0	0	40	0	0	35	0	145
CTC_RS03365	694390..695022	64	75	21	96	0	109	103	124	42	37	254	113	0	93	66	75	120	100	141	153	189	0	59	64	38	177	0	337	504	454

CTC_RS03370	695073..695591	911	1051	615	794	530	664	611	1304	1544	558	526	598	640	818	930	1063	972	537	1114	652	415	789	647	314	657	969	804	887	804	794
CTC_RS03375	complement(695640..697559)	0	37	14	24	12	18	10	33	14	86	17	0	11	23	55	14	0	33	22	25	25	13	19	42	12	12	12	18	0	40
CTC_RS03380	697760..698200	521	1506	1643	415	1767	2189	793	1678	1030	1835	2296	812	360	1295	666	710	1430	1378	511	219	1871	338	1016	1846	414	1267	736	1263	2338	2521
CTC_RS03385	698197..699138	861	1637	2590	960	2117	3586	1070	1738	1928	1335	2841	2648	667	1476	1837	1298	2196	1680	803	1746	2603	922	1823	2680	657	1501	2461	1073	2058	2503
CTC_RS03390	complement(699185..699547)	0	588	476	0	316	47	0	1258	515	0	133	132	20	484	289	0	347	942	0	0	0	0	0	0	22	616	447	0	778	158
CTC_RS03395	699718..700005	0	0	46	0	80	120	0	0	0	41	0	0	0	51	218	24	88	0	15	0	0	0	0	0	0	39	0	0	0	0
CTC_RS03400	700107..701012	0	0	0	17	25	19	0	35	29	0	0	0	0	0	0	11	0	28	5	53	0	55	0	0	0	25	0	15	0	42
CTC_RS03405	701022..702467	0	16	46	47	48	143	0	22	0	29	44	50	10	51	29	26	17	96	44	0	33	0	26	28	0	23	32	95	102	93
CTC_RS03410	702591..703298	0	34	94	43	65	73	26	145	38	42	79	135	20	83	89	29	178	179	6	68	0	0	158	58	78	47	33	58	69	190
addB	703423..706875	43	62	73	53	146	100	58	73	77	38	65	121	48	64	43	63	66	99	62	70	125	68	119	59	48	74	34	44	36	105
CTC_RS03420	complement(706931..708031)	0	22	0	0	0	0	0	0	0	5	29	22	0	0	0	0	0	0	0	0	0	23	0	111	0	10	0	0	0	17
CTC_RS03425	complement(708021..708554)	0	0	0	0	0	32	0	0	0	0	30	0	0	0	0	0	0	0	0	0	0	0	0	0	0	0	0	0	0	72
CTC_RS03430	708816..709025	0	0	0	36	0	0	0	0	0	84	0	0	0	0	0	0	0	0	0	0	228	0	0	0	0	0	0	0	0	0
CTC_RS03435	complement(709060..709272)	0	891	62	0	0	324	0	1182	2006	0	453	1233	0	69	98	0	1303	297	0	0	225	0	0	0	0	105	218	0	1037	270
CTC_RS03440	709488..711554	0	11	19	30	0	67	9	0	0	67	31	35	0	0	0	0	12	6	21	0	12	6	36	98	8	11	0	20	0	19
CTC_RS14140	complement(711634..713133)	27	63	35	56	138	46	56	52	36	138	107	32	34	137	84	41	17	34	31	0	64	33	25	136	37	67	93	101	33	102
CTC_RS03455	complement(713469..715616)	0	0	0	0	0	0	0	0	0	7	0	0	0	7	20	3	0	6	0	0	0	0	17	38	0	0	0	3	0	9
CTC_RS03460	complement(715777..716577)	17	30	17	29	29	43	23	49	0	22	100	60	45	73	52	17	63	63	16	121	149	0	47	51	10	28	29	17	0	48
serA	complement(716641..718242)	17	89	100	33	100	65	52	88	17	15	130	119	50	64	26	38	63	103	53	393	105	62	70	51	59	70	14	69	15	72
CTC_RS03470	complement(718281..719351)	25	89	37	50	128	48	70	59	25	44	135	134	54	27	59	45	24	166	50	136	290	35	35	38	74	21	43	58	46	143
CTC_RS03475	complement(719684..721237)	78	15	77	34	59	189	78	71	52	114	31	92	46	19	27	39	49	41	36	31	92	40	144	183	26	29	15	58	47	12
CTC_RS03480	721335..721652	127	224	125	480	649	434	88	742	336	204	1567	1126	238	598	396	278	714	319	281	304	677	234	704	1024	150	598	146	173	1312	301
CTC_RS03485	721679..722023	196	275	154	243	299	350	81	319	310	0	1351	553	126	552	243	168	585	220	271	841	555	216	325	118	69	259	134	20	427	111
CTC_RS03490	722101..722661	337	465	320	612	572	492	200	645	286	168	1289	1021	277	365	262	340	899	474	296	1207	682	531	1064	363	269	319	165	294	613	171
CTC_RS03495	complement(722754..724013)	0	56	32	6	18	96	15	0	0	33	38	38	6	12	0	5	80	25	7	0	38	10	30	0	6	9	0	44	19	46
CTC_RS03500	complement(724019..725155)	36	42	0	13	60	15	33	28	23	47	28	63	32	39	18	36	0	100	11	43	21	0	0	36	14	49	0	42	43	67
CTC_RS14950	725494..725742	54	95	0	31	0	69	112	0	0	47	0	0	29	0	0	82	0	0	85	0	0	0	0	0	0	0	0	55	99	0
CTC_RS14955	725971..726267	0	0	45	0	0	58	126	53	0	477	162	161	73	0	0	46	0	0	200	489	81	0	126	0	0	38	0	93	165	0
CTC_RS03510	726485..727462	69	170	82	86	188	141	38	64	82	139	74	98	37	135	64	49	0	181	135	99	73	38	76	125	32	160	119	56	125	274
CTC_RS03515	727518..728144	43	265	127	110	329	137	119	126	213	104	282	133	69	163	234	98	282	192	136	77	76	158	60	65	25	125	222	110	157	183
CTC_RS03520	728293..728712	32	56	63	18	55	0	0	37	127	56	38	284	69	174	200	65	180	30	40	0	171	148	0	97	0	0	166	98	0	137
CTC_RS03525	728799..730481	0	0	0	0	14	0	0	9	0	0	19	57	0	9	0	0	15	8	0	0	0	0	0	0	0	0	0	0	0	11
CTC_RS03530	730766..732340	9	45	17	10	0	0	6	0	0	7	10	30	14	56	0	30	16	16	8	31	0	39	24	0	35	7	29	17	16	24
CTC_RS03535	732341..733738	39	51	0	11	16	0	0	11	0	8	11	0	10	52	0	15	0	0	3	35	17	0	0	0	6	0	0	15	0	0
CTC_RS03540	733820..734959	5465	1248	904	4853	1005	1134	5638	725	117	863	1565	4041	13676	848	1500	9786	2545	533	4979	4519	1490	7477	1179	589	15172	1088	1464	1791	2756	1572
CTC_RS03545	734977..735756	0	0	0	10	0	11	0	0	0	0	0	0	9	0	0	0	0	0	5	0	0	0	0	0	25	0	30	0	0	0
CTC_RS03550	735777..736787	815	633	552	8	0	0	6538	872	727	321	262	626	71	0	62	40	0	13	0	0	0	0	0	40	43	22	0	0	24	0
addA	736881..740645	14	19	35	26	61	23	22	17	7	31	34	38	19	35	19	33	13	37	29	0	44	26	10	22	13	27	18	15	7	25
CTC_RS03560	740793..741527	0	129	36	52	156	117	0	107	36	32	241	97	0	219	29	0	137	52	20	66	33	0	51	55	76	335	95	9	134	78
CTC_RS03565	complement(742344..743141)	0	0	33	57	72	43	58	20	33	37	121	0	36	37	0	51	32	32	16	30	60	31	94	102	50	70	0	0	0	96
cobC	743335..743928	0	0	0	0	0	29	31	0	0	10	0	40	12	25	0	0	0	64	57	81	0	0	0	0	0	0	0	12	0	0
cobU	743964..744518	24	0	72	69	0	124	67	28	0	64	29	258	52	0	38	86	45	114	153	87	86	67	0	220	14	60	125	12	133	35
cobS	744531..745289	36	63	35	10	0	0	25	0	0	70	0	126	10	19	28	9	33	33	67	0	95	0	0	54	0	15	0	54	32	25
CTC_RS03585	745755..747221	64	162	54	36	109	106	57	43	55	227	66	98	69	80	43	163	138	216	165	99	179	51	25	83	43	179	63	131	100	105
cbiB	747256..748209	14	124	125	40	168	108	20	0	56	266	101	0	38	92	0	36	0	27	196	152	50	26	0	85	33	82	49	86	51	40
CTC_RS03595	748215..749333	36	21	107	41	143	216	8	0	24	169	43	85	32	105	75	43	0	45	133	130	43	22	100	182	14	130	41	68	0	17
CTC_RS03600	749330..750055	205	457	55	294	568	356	13	130	37	537	255	197	239	202	231	122	104	122	492	133	165	308	308	785	109	431	192	312	135	211
CTC_RS03605	750052..750351	135	316	177	153	153	230	93	105	89	177	161	0	168	195	70	136	0	42	425	645	239	207	249	407	26	224	0	275	327	319
cbiQ	750329..751024	0	0	0	11	33	50	0	90	0	72	0	34	0	0	0	0	0	0	49	70	69	18	0	0	11	16	0	30	71	28
hemA	751072..752271	11	20	11	13	38	14	0	26	0	79	27	20	12	0	0	0	0	42	32	40	0	0	31	0	7	19	0	34	0	16
CTC_RS03620	752229..752960	0	0	0	10	0	24	13	22	0	56	0	0	10	20	0	9	34	17	23	66	65	0	0	56	0	0	32	19	0	0
hemC	752878..753750	93	109	30	52	53	39	21	72	0	115	55	82	58	50	24	129	0	116	170	0	55	28	43	0	18	166	27	150	56	88
cobA	753763..755220	56	81	46	63	189	130	51	119	37	267	33	65	64	80	58	84	35	96	149	66	82	128	282	56	60	84	32	165	51	79
hemB	755237..756217	83	375	81	109	257	141	86	112	82	337	66	195	151	179	64	215	206	90	204	197	195	89	114	207	137	182	260	294	125	274
hemL	756210..757478	106	206	94	162	253	122	37	217	168	326	152	150	154	288	83	209	239	150	293	267	170	166	162	96	137	273	238	287	155	121
CTC_RS03645	757491..758810	41	54	60	40	122	104	35	60	40	206	0	0	49	33	16	67	115	38	55	73	109	66	57	31	6	25	18	161	9	73
CTC_RS03650	758837..759469	149	150	84	145	253	82	29	224	42	224	25	151	114	185	166	194	159	240	141	0	189	137	59	0	125	141	147	195	271	30
cbiD	759483..760553	76	44	87	100	214	64	96	191	25	248	135	156	88	178	98	95	118	95	254	0	89	116	279	38	141	146	87	141	23	107
cbiE	760567..761193	65	38	21	37	146	55	30	25	43	94	51	38	35	47	0	65	40	40	102	0	0	40	0	0	25	71	0	77	39	31
cbiT	761212..761781	0	166	23	27	40	0	33	55	0	10	0	42	13	0	37	48	0	44	52	0	0	65	196	0	70	59	0	60	43	67
CTC_RS03670	761774..762439	41	71	120	80	69	0	28	71	0	133	24	0	33	33	0	72	38	38	109	73	0	37	168	122	48	101	0	114	37	58
cobM	762466..763227	266	93	52	140	90	23	122	145	105	318	42	31	95	19	83	98	132	116	335	190	188	49	147	0	31	29	61	271	32	101
cbiG	763231..764226	27	24	27	61	46	35	37	87	27	243	32	24	22	29	21	34	25	0	128	49	72	37	75	123	24	22	23	117	0	58
cobJ	764256..764972	38	66	19	64	160	24	26	0	0	181	22	33	40	102	0	85	35	71	125	0	67	87	0	57	22	31	65	134	34	0
CTC_RS03690	764990..765760	0	31	0	0	119	112	12	0	35	211	0	0	28	19	27	53	33	66	66	0	62	16	0	53	10	29	0	107	96	99
CTC_RS03695	765853..766359	53	140	52	90	45	0	18	0	0	280	32	0	50	0	41	40	100	0	59	95	0	73	74	0	16	132	0	81	48	38
CTC_RS03700	766346..767098	18	63	0	41	0	114	37	42	35	180	0	63	24	97	84	63	0	84	68	0	0	0	50	54	21	104	0	82	0	25
CTC_RS03705	767134..767937	34	89	17	38	0	64	12	20	0	154	40	0	27	36	0	25	31	63	79	60	0	0	46	0	20	14	58	51	0	72
CTC_RS03710	768079..768597	1042	868	589	941	1678	764	1141	789	412	603	1270	1012	1460	917	566	1024	729	683	844	1538	530	1207	1618	1020	1337	1066	625	623	804	776
CTC_RS14145	768888..769277	277	426	341	117	353	619	622	283	411	485	536	61	722	300	323	262	194	97	98	124	245	95	574	731	264	258	594	141	63	147
cobO	769299..769817	312	503	781	559	927	1627	539	607	721	552	1332	966	661	931	727	400	681	878	369	280	599	454	1223	1569	504	861	670	278	426	517
CTC_RS14150	770298..774542	6	50	25	4	22	37	7	19	6	22	34	17	2	14	15	3	12	9	7	0	0	0	26	19	2	18	16	31	6	34
CTC_RS03730	774778..776166	19	0	10	0	0	0	7	0	0	43	23	34	5	0	0	0	0	0	6	0	17	0	0	29	11	0	0	49	0	14
CTC_RS03735	776159..781825	0	17	19	1	24	33	8	14	2	38	34	23	1	8	19	0	13	25	5	9	0	4	13	22	6	6	0	25	17	24
CTC_RS03740	781883..786418	9	63	53	19	111	68	14	43	24	85	30	45	13	55	32	20	22	70	15	21	21	30	66	36	16	59	56	85	11	23
CTC_RS03745	786561..787463	0	0	15	0	0	38	10	0	0	26	0	0	16	0	0	0	0	28	0	54	26	0	0	0	9	12	0	0	54	0
CTC_RS03750	787445..787905	0	51	58	33	0	75	40	0	116	90	0	155	47	64	45	44	55	55	28	0	0	0	0	0	34	48	50	60	53	42
CTC_RS14960	788407..789069	61	72	181	12	35	78	56	142	0	45	24	0	65	44	158	31	76	76	26	73	0	37	113	246	24	17	245	73	37	29
CTC_RS03760	789070..789756	20	0	19	11	0	25	27	115	78	60	47	35	0	21	61	30	37	18	37	0	0	0	0	0	0	65	67	80	107	84
CTC_RS14155	789859..790839	441	1523	1234	327	1332	2029	504	1171	790	735	1131	827	287	716	706	562	772	871	340	690	975	329	2054	2158	331	900	898	554	625	997

CTC_RS03770	790974..791708	55	161	163	62	94	352	178	64	145	104	153	65	98	219	428	74	240	353	226	132	358	17	254	277	76	167	126	51	33	104
CTC_RS03775	791710..792630	132	52	188	124	149	243	46	154	116	154	349	415	78	151	228	185	411	412	355	368	260	27	81	88	121	158	227	153	133	271
CTC_RS03780	792649..792909	52	91	0	0	0	165	0	0	0	79	62	0	28	0	0	0	0	97	33	0	0	0	143	0	0	0	0	0	0	0
CTC_RS03785	793821..794966	0	62	46	0	60	60	33	55	93	67	140	83	25	51	110	6	44	44	15	84	21	0	33	36	28	68	142	6	0	33
CTC_RS03790	794986..797013	0	23	0	4	23	34	18	8	13	58	32	47	0	22	10	7	0	12	2	0	24	0	0	40	4	0	11	7	0	9
CTC_RS03795	797026..797754	0	33	18	0	0	0	0	0	0	0	0	0	0	0	0	0	0	0	0	0	0	0	51	56	0	0	0	0	0	0
CTC_RS03800	797735..799273	0	23	26	0	0	56	6	10	0	42	31	31	5	10	27	0	0	58	3	0	62	8	49	53	0	7	15	0	16	12
CTC_RS03805	799276..800250	0	24	27	8	71	71	10	65	0	67	0	49	15	0	22	14	0	65	17	50	25	0	0	42	0	34	48	14	0	39
CTC_RS03810	800251..801090	0	0	47	0	27	62	0	0	0	21	19	28	0	17	25	8	0	45	10	0	0	15	44	0	0	0	0	0	29	23
CTC_RS03815	801093..802607	0	31	0	0	30	46	6	21	18	66	53	63	0	48	0	0	33	58	11	0	32	0	0	27	5	15	0	9	0	63
CTC_RS03820	802609..805125	0	47	26	3	18	55	7	31	11	120	42	47	22	41	17	8	20	40	7	0	67	0	44	32	6	53	46	30	0	23
CTC_RS03825	805142..806728	9	157	109	19	188	293	53	40	151	227	142	211	27	120	172	13	16	56	19	30	90	8	165	128	57	155	102	95	15	24
CTC_RS14160	806878..808584	55	403	171	4	242	384	44	212	141	429	160	182	59	137	98	24	0	82	52	142	140	0	306	310	42	249	149	129	101	45
CTC_RS03835	808662..810473	37	367	95	8	202	171	31	165	147	308	257	310	48	153	301	30	28	77	70	107	132	14	206	157	83	228	154	186	68	201
CTC_RS14675	810525..810731	65	0	64	0	0	83	45	76	0	114	0	0	70	0	0	0	0	0	0	0	0	0	0	0	0	0	0	0	0	0
CTC_RS03840	810817..811866	64	316	127	7	306	181	80	225	51	298	184	386	82	195	280	13	24	72	49	92	114	47	142	0	68	245	177	111	47	37
CTC_RS03845	811902..812984	37	307	209	49	169	127	17	174	148	338	193	309	107	257	136	13	47	164	53	89	133	23	414	376	73	299	214	140	11	159
CTC_RS14165	813005..815008	34	213	93	23	229	172	33	157	107	348	144	119	97	183	52	48	63	47	59	72	90	15	186	61	123	184	93	134	49	77
CTC_RS03855	815058..816119	64	246	238	50	173	211	53	296	151	517	166	90	129	276	79	64	71	72	64	137	135	23	246	153	105	232	131	240	23	108
CTC_RS03860	816402..817469	7295	9539	4588	2197	4325	5060	8241	8799	4452	1319	768	927	9711	8580	3673	3275	2669	2584	3221	3805	3405	2236	5556	2554	7778	8069	4287	1725	287	682
CTC_RS14170	817556..823198	333	736	615	198	459	535	526	484	464	83	117	197	749	569	494	158	264	303	204	317	435	177	331	404	725	600	481	123	35	126
CTC_RS03875	823810..824295	28	390	876	47	189	709	115	421	879	36	33	98	59	346	1424	168	675	625	302	1593	1723	51	307	921	49	299	668	71	101	197
CTC_RS03880	824484..826205	196	200	100	386	186	210	282	402	171	137	485	319	348	281	244	411	220	279	302	253	486	490	433	284	320	331	242	104	371	267
CTC_RS03885	826235..827086	16	167	94	260	135	223	99	268	125	125	302	224	186	172	123	112	133	119	160	341	225	218	175	143	200	223	82	65	173	180
asnA	827494..828519	119	69	39	164	67	0	27	169	0	328	94	47	123	0	20	133	49	136	284	0	0	121	73	40	108	44	23	241	72	75
CTC_RS03895	828637..829236	0	0	0	0	0	0	0	0	0	0	0	0	0	0	0	0	0	0	0	0	0	0	0	0	0	0	0	0	0	0
CTC_RS03900	complement(829233..830090)	0	0	0	9	0	0	0	0	0	48	0	28	0	0	0	0	29	0	15	0	0	0	87	0	0	0	0	8	0	0
CTC_RS03905	complement(830092..831144)	0	0	0	7	0	0	0	15	0	45	15	0	0	7	0	6	0	60	12	0	23	0	0	0	8	0	0	13	0	18
CTC_RS03910	complement(831167..832111)	29	25	28	32	0	18	10	0	0	137	17	76	31	31	22	94	0	134	49	0	76	39	79	86	17	24	25	51	0	20
CTC_RS03915	832312..833145	0	0	16	14	27	103	0	0	0	71	19	0	17	0	0	16	0	15	41	0	86	0	90	98	0	27	0	62	0	0
brnQ_1	833605..834909	31	36	0	12	0	13	0	0	0	23	0	0	17	0	0	5	0	19	23	0	0	29	0	31	27	0	0	26	0	0
CTC_RS03925	complement(834976..836312)	0	0	0	0	0	0	0	0	0	0	0	0	0	0	0	0	0	0	0	0	0	0	0	0	0	0	0	0	0	0
CTC_RS03935	836525..837499	14	49	55	55	12	44	19	105	27	30	264	122	15	180	0	28	78	65	70	99	123	64	0	0	8	103	119	42	101	59
CTC_RS03940	837786..838793	94	188	66	106	45	120	139	125	159	264	144	142	86	131	166	264	225	364	299	192	285	62	370	202	118	122	115	273	317	152
CTC_RS03945	838806..839837	65	161	258	259	155	251	194	168	233	401	280	185	126	270	183	274	122	331	360	328	255	84	181	513	123	173	225	313	523	297
CTC_RS03950	839904..840542	0	37	21	24	0	27	44	49	0	55	0	0	11	0	0	75	0	0	53	0	37	0	58	0	37	0	0	65	0	30
trmB	840586..841221	0	0	0	12	0	27	0	0	0	46	0	0	11	0	0	21	0	20	27	0	0	0	0	0	12	0	36	43	0	30
CTC_RS03960	841362..842981	8	15	33	19	14	11	6	19	0	55	0	15	9	0	0	25	0	8	45	0	0	8	0	0	10	14	0	64	0	12
CTC_RS03965	complement(843031..844005)	0	97	68	8	47	35	0	65	55	194	0	24	7	45	0	35	26	65	39	0	25	25	0	0	0	29	48	35	76	39
tpx	complement(844256..844750)	2239	1773	2713	2197	3913	3865	980	2576	3885	11639	3685	2459	802	1286	2500	1926	764	1330	1709	3274	8602	2243	2941	5346	1234	1377	3419	5278	2479	8055
CTC_RS03975	844924..845673	54	32	0	0	0	92	50	21	0	24	129	32	10	0	0	18	67	0	113	64	0	17	0	0	0	15	0	82	65	26
CTC_RS03980	complement(845732..846319)	0	0	0	0	0	0	0	0	0	0	0	0	0	0	0	0	0	0	0	0	0	0	0	0	0	0	0	0	0	0
CTC_RS03990	complement(846963..847568)	22	0	0	0	0	28	15	0	0	0	0	0	0	0	0	0	0	0	0	0	0	0	0	0	0	0	0	0	0	0
CTC_RS03995	complement(847585..848184)	0	0	0	0	0	0	0	0	0	0	0	0	0	0	0	0	0	0	7	0	0	0	0	0	0	37	0	0	0	0
CTC_RS04000	complement(848197..849078)	31	0	15	17	26	59	0	18	0	20	18	0	0	0	0	0	0	14	14	0	0	0	0	46	27	0	26	16	0	0
CTC_RS04005	complement(849091..849456)	37	0	73	31	0	0	0	0	0	32	0	65	39	0	0	37	0	0	0	0	65	68	306	111	0	92	0	19	0	105
CTC_RS04010	849627..850328	58	34	133	54	65	74	0	179	38	84	114	170	0	125	60	68	36	144	67	69	34	18	213	174	11	127	66	39	35	82
CTC_RS04015	850345..851910	17	61	76	73	117	66	30	50	68	87	31	46	14	103	27	115	113	154	58	62	115	40	119	52	20	71	30	75	78	147
CTC_RS04020	851930..852808	15	135	76	104	78	176	11	125	91	108	55	54	33	100	48	39	86	72	73	0	95	28	127	232	27	38	53	16	0	22
CTC_RS04025	complement(853101..853544)	396	1068	1887	498	1084	2698	714	1383	2587	439	652	1666	797	857	1323	690	1648	4563	493	1961	4957	279	1261	2567	1054	1133	1932	364	1769	4727
CTC_RS04030	complement(853754..854002)	488	191	1255	368	276	1108	487	0	644	2253	646	1438	58	118	674	109	1013	610	376	777	1441	149	300	3515	159	45	279	1905	690	693
CTC_RS04035	854223..857144	28	114	77	97	110	47	93	86	46	136	99	61	83	90	65	114	104	108	71	33	90	106	64	28	62	142	48	71	76	131
CTC_RS04040	857352..859070	483	2815	2081	1214	2814	1354	1308	2660	2276	141	2819	2624	1100	2248	2825	1377	3331	1422	1604	4334	1642	1458	1759	1137	1010	1967	2542	212	3362	1784
CTC_RS04045	859140..860060	0	26	14	66	25	37	66	85	0	0	105	104	55	0	23	22	55	27	9	158	78	40	0	0	0	12	0	0	27	42
CTC_RS04050	complement(860094..860978)	99	80	90	52	52	58	63	53	91	254	18	0	24	50	47	100	0	86	34	0	27	14	211	184	18	114	26	124	28	108
CTC_RS04055	861354..862046	0	0	77	0	0	0	13	23	0	0	23	34	0	63	0	0	0	18	0	0	35	0	0	0	0	16	0	0	142	28
CTC_RS04060	862143..863414	0	131	31	6	36	0	0	62	21	14	625	19	0	253	33	5	20	50	3	0	0	10	0	0	0	211	55	0	116	60
preA	863414..864649	0	192	54	6	185	14	8	153	22	10	663	77	6	355	34	22	194	31	7	157	39	0	91	33	0	217	38	11	139	78
CTC_RS04070	complement(864827..865564)	37	0	108	103	93	374	51	43	0	88	196	323	68	20	57	46	34	86	52	262	357	151	152	496	75	30	31	0	67	156
CTC_RS04075	865890..867272	3826	6097	14524	6489	12008	24746	3120	3341	18367	179	9774	17629	4470	4869	18306	7732	6047	10624	2359	11088	24199	6688	11618	26567	4243	4934	18398	303	2600	11996
CTC_RS04080	867419..868759	2217	1707	3560	4407	3179	7731	1023	857	3018	185	3056	2510	1421	737	2354	2697	1260	2346	1322	2237	6618	3877	2728	7013	1443	754	2706	103	540	1880
CTC_RS04085	868891..869565	60	35	79	79	0	153	69	47	158	477	48	71	32	43	31	20	56	131	113	0	142	37	55	0	0	66	103	194	0	170
CTC_RS04090	869578..870492	118	0	247	125	125	377	122	69	29	387	53	183	71	32	46	104	28	249	200	53	366	170	41	222	78	24	152	240	27	356
CTC_RS04095	870766..871995	2098	2276	2124	1653	1892	2284	1305	1523	2052	735	3195	2367	1567	1821	1518	2209	2502	731	850	1848	1400	1664	1760	1953	1560	1536	1753	556	1856	1231
CTC_RS04100	872010..872705	1320	1636	1414	1250	1153	1139	831	792	1343	280	2379	1680	1333	1956	1627	1869	1758	682	614	1460	1066	1658	1394	819	1117	1188	1232	504	1058	826
CTC_RS04105	872757..874169	1559	1301	1308	1472	1225	2013	924	791	775	397	1866	2432	1272	901	638	1687	1107	394	626	1575	1168	1620	898	1585	1353	791	853	341	660	1017
CTC_RS04110	874260..875438	321	121	135	168	194	453	222	40	45	185	409	283	86	74	36	139	107	64	119	123	386	189	127	311	121	123	39	128	62	130
CTC_RS04115	875536..876081	13514	21678	14869	18582	16016	15769	19222	25759	27346	15274	13041	17726	15242	21489	44274	38403	25136	22261	27480	33755	21775	21748	23207	39555	18845	21434	39001	45579	43604	30017
CTC_RS04120	876206..876997	34	30	67	48	29	174	24	79	101	127	142	90	27	37	53	26	0	128	59	0	60	0	47	308	40	0	146	87	62	24
thiC	877273..878589	21	108	121	58	157	236	7	72	41	27	244	326	33	33	80	26	287	48	26	220	145	28	85	124	42	110	106	26	37	87
CTC_RS04135	878962..880298	0	0	0	0	0	0	0	0	0	0	0	0	0	0	0	0	0	0	0	0	0	0	0	0	0	0	0	0	0	0
CTC_RS04140	complement(880363..880692)	410	431	685	509	486	1045	509	382	1376	1083	292	217	503	710	1144	599	764	652	528	147	1305	113	452	1357	433	1118	773	708	372	1278
CTC_RS04145	880851..881933	0	0	25	21	32	16	0	29	0	65	30	88	0	27	19	6	23	0	16	45	155	23	0	75	0	26	21	13	68	53
CTC_RS04150	882354..882863	26	0	0	0	0	0	18	0	0	12	32	0	0	0	0	0	0	0	8	0	0	0	0	0	0	0	0	0	0	0
CTC_RS04155	complement(882919..883518)	23	0	0	25	0	29	47	0	45	128	0	80	24	0	35	45	0	21	50	0	80	62	0	0	0	0	0	11	0	32
CTC_RS04160	883736..884341	0	0	22	25	0	0	0	52	0	49	27	0	0	24	0	11	42	21	7	0	0	0	0	0	0	0	0	23	0	95
CTC_RS14680	884401..884553	0	0	0	0	0	0	0	0	0	0	0	0	0	96	0	0	0	0	0	0	0	0	0	0	0	0	0	0	0	0
CTC_RS04165	884772..885617	0	0	31	9	27	41	0	56	0	21	19	85	9	0	25	32	30	60	5	0	0	0	44	48	9	40	55	0	29	23
CTC_RS04170	885610..887325	0	28	8	22	40	0	22	37	0	31	9	0	0	34	12	40	0	30	0	0	14	7	22	24	18	33	14	24	29	22
CTC_RS04175	887328..888587	11	75	11	30	18	41	7	37	0	61	13	19	6	46	0	22	20	60	10	0	0	20	118	0	25	0	0	27	39	15

CTC_RS04180	888637..890709	13	23	13	48	44	33	41	30	26	85	31	0	24	35	0	16	12	31	14	47	12	24	36	0	8	22	28	33	0	28
CTC_RS04185	890763..891866	24	107	72	159	145	62	46	43	73	166	73	43	46	119	133	123	46	92	77	88	22	0	0	37	29	121	63	93	89	104
CTC_RS04190	complement(891896..892183)	0	82	0	26	80	299	0	55	93	41	279	0	25	0	0	0	0	88	15	168	415	43	0	141	55	116	0	24	0	67
CTC_RS04195	complement(892279..893637)	10	17	20	0	17	0	7	12	0	174	24	9	0	0	62	5	19	9	31	0	0	0	0	30	0	8	17	71	0	56
trxB	893891..894838	135	325	477	56	339	418	153	374	282	1193	492	151	15	371	431	172	213	401	715	357	858	321	472	902	46	401	440	243	466	910
CTC_RS04205	894831..895061	176	616	173	165	595	448	444	886	463	358	835	310	125	190	454	752	764	658	626	838	1139	537	323	1939	103	1016	1104	268	319	332
CTC_RS04210	895220..895957	0	0	0	0	0	0	0	43	36	32	44	32	29	20	0	28	0	17	29	66	32	50	0	110	0	15	0	37	0	26
CTC_RS04215	896090..897043	184	423	572	360	1057	470	284	759	728	180	758	988	310	721	792	471	344	624	165	406	602	351	743	427	320	697	510	166	708	764
CTC_RS04220	897166..898548	0	0	0	6	0	25	0	0	39	13	12	17	0	11	0	10	0	0	9	35	69	0	0	0	0	8	0	0	0	14
CTC_RS04225	898564..899466	0	0	29	0	25	38	10	17	30	13	53	0	16	16	0	0	56	14	28	0	53	14	0	0	0	37	0	0	14	0
CTC_RS04230	899488..900448	0	25	21	0	0	54	0	0	0	18	17	0	0	0	11	14	26	7	13	50	25	26	0	42	0	0	0	14	26	10
CTC_RS04235	900528..901220	0	103	58	0	0	0	13	0	0	26	0	34	21	0	30	39	109	73	37	0	242	0	54	0	23	0	67	40	35	83
CTC_RS04240	901222..902664	28	115	37	32	16	60	26	87	111	41	150	83	20	112	29	33	79	35	74	235	99	0	0	0	22	139	64	71	153	53
CTC_RS04245	complement(902694..903824)	0	21	47	7	0	30	0	0	0	52	0	21	19	0	0	18	0	11	19	0	0	11	0	0	0	0	0	12	0	0
CTC_RS04250	complement(903838..905022)	11	40	11	32	0	29	16	0	23	35	14	40	6	0	0	17	0	107	7	0	61	0	0	69	0	19	20	29	0	49
CTC_RS04255	complement(905037..906020)	0	24	0	8	23	18	0	16	0	12	33	24	15	0	21	7	26	39	22	0	24	13	0	41	0	0	0	0	150	136
CTC_RS14965	complement(906064..906384)	0	0	41	0	0	0	0	0	0	0	0	0	0	0	0	0	0	0	0	0	0	0	0	0	0	0	0	21	0	0
CTC_RS04265	906970..907248	48	255	1001	178	822	2224	67	169	766	402	807	1540	26	157	376	134	814	363	61	867	1115	44	268	2189	28	361	582	148	572	824
CTC_RS04270	907272..908141	47	818	1590	232	909	2179	295	1086	1458	662	905	3896	83	496	1495	360	1450	1550	276	1223	3135	143	643	2527	118	488	1466	482	1044	1586
CTC_RS04275	908491..908829	0	0	0	45	0	0	110	46	0	0	95	70	85	0	62	80	74	75	63	0	141	0	0	120	47	33	0	0	0	0
CTC_RS04280	908892..909572	754	662	332	1871	471	557	1137	231	471	147	401	333	1908	215	185	1845	704	465	499	497	105	2250	548	897	1537	378	170	61	432	535
CTC_RS04285	complement(909774..911297)	9	16	79	0	0	90	0	31	70	109	42	0	9	43	41	4	166	141	17	0	47	0	0	27	0	37	15	90	64	38
CTC_RS04290	complement(911324..912334)	40	0	0	8	0	0	9	0	26	134	0	24	7	0	21	20	25	0	34	0	0	0	0	0	8	0	0	82	24	0
mglB	912809..913855	13	340	102	0	131	230	0	226	0	0	507	296	7	391	180	13	723	97	4	370	0	0	143	194	8	267	155	13	2250	73
CTC_RS04300	913939..915453	0	0	44	5	0	34	0	0	0	4	42	32	0	0	0	0	117	33	6	64	24	0	25	0	0	0	15	5	227	0
mglC	915473..916483	13	23	0	8	45	68	0	16	53	6	32	47	7	29	0	7	175	63	4	0	24	0	0	0	0	11	0	7	218	19
CTC_RS04310	916580..917584	13	24	66	53	160	137	28	63	0	53	120	166	14	44	83	136	226	214	63	48	95	49	186	122	47	0	46	75	98	210
CTC_RS04315	917668..919221	9	15	26	10	37	89	0	20	0	8	93	92	5	19	13	0	24	33	8	31	46	0	72	79	5	29	15	0	95	49
CTC_RS04320	919242..920399	0	41	0	13	0	104	0	54	23	0	28	124	6	13	18	18	0	22	15	0	41	16	64	141	7	39	40	12	106	17
CTC_RS14685	920389..920529	0	0	0	0	0	0	0	0	0	0	0	0	0	0	0	0	0	0	0	0	0	0	0	0	0	0	0	0	0	0
galE	920555..921538	0	145	68	23	140	193	0	80	27	24	180	194	7	74	192	28	128	51	26	0	73	0	114	124	0	182	47	14	224	39
rpoD_1	921774..922598	49	115	161	92	222	481	102	134	97	444	292	550	61	71	153	99	92	215	149	293	580	0	181	493	19	95	169	175	238	174
CTC_RS04335	complement(922635..923366)	0	32	18	0	31	118	13	0	36	65	22	33	10	40	57	9	34	17	12	0	131	0	0	56	0	0	32	28	0	26
CTC_RS14690	923605..923748	0	0	0	0	0	60	0	0	0	0	0	0	0	0	0	0	0	0	15	0	0	0	0	0	0	78	0	0	0	0
CTC_RS04340	923821..924381	36	42	95	54	41	92	67	112	95	53	86	43	116	300	112	61	0	90	91	172	0	0	67	145	28	279	83	110	175	103
CTC_RS04345	complement(924419..925012)	0	1278	963	0	328	116	0	2676	764	0	406	161	0	2046	777	0	2081	703	14	81	40	0	377	206	0	2729	781	0	3058	742
CTC_RS04350	complement(925035..925304)	0	1318	1428	0	255	192	0	2798	1088	0	952	177	0	1789	1010	0	1588	891	0	358	0	0	553	0	0	2422	687	0	3909	1065
CTC_RS04355	complement(925328..925507)	0	132	74	0	0	0	0	262	0	0	0	0	0	163	0	0	0	0	0	0	0	0	0	0	0	248	0	0	273	106
CTC_RS04360	complement(925635..925898)	0	0	0	0	0	131	0	0	0	0	61	0	0	0	0	0	0	0	0	0	0	0	0	154	0	0	0	0	186	73
CTC_RS04365	926067..926753	20	0	0	0	0	25	0	0	0	17	47	35	0	43	31	0	0	18	0	0	0	0	0	0	0	0	0	0	0	0
CTC_RS04370	926753..928126	10	0	10	11	0	0	0	0	0	9	23	0	0	0	0	0	18	18	5	0	0	0	0	0	0	8	0	3	0	28
CTC_RS04375	928285..928551	101	1866	1096	457	2146	1097	489	1120	1100	1217	3612	894	243	1864	236	332	378	522	1011	725	448	418	2935	1525	594	1821	260	592	827	933
CTC_RS04380	928573..931017	171	601	310	314	769	634	132	396	317	587	539	459	100	533	184	266	103	207	550	119	205	152	779	666	114	430	175	523	286	521
CTC_RS04385	931050..931256	65	0	0	147	111	0	0	76	129	57	0	115	0	71	0	33	0	0	0	0	116	0	180	0	38	0	112	66	0	0
CTC_RS04390	931494..933143	164	115	105	220	167	146	226	134	81	344	78	101	87	89	76	194	61	165	299	88	145	83	113	148	53	78	169	385	149	186
CTC_RS04395	complement(933159..933425)	202	355	747	486	859	1097	280	472	1901	730	843	1341	216	493	628	153	472	1280	350	362	896	720	839	3050	238	335	1129	180	368	790
CTC_RS04400	933871..934452	998	4035	2742	2209	3663	2962	1763	3300	4130	330	7070	7813	1184	2968	3658	3189	2557	2339	1439	2327	2261	2089	3431	2938	1097	3025	2948	331	2952	4660
CTC_RS14970	complement(934564..934875)	43	0	0	0	0	110	0	0	86	0	0	0	0	0	67	0	0	0	27	0	0	0	120	0	0	0	0	0	0	0
CTC_RS04410	complement(934960..935547)	92	484	520	201	156	1055	32	187	273	306	191	406	86	100	428	197	172	366	72	123	854	84	698	2008	135	57	453	234	668	1043
CTC_RS04415	complement(935728..936285)	0	0	24	14	0	0	0	28	0	32	0	0	13	26	0	0	0	0	8	0	0	44	0	0	0	0	0	25	0	0
CTC_RS04420	936420..938111	16	84	63	18	41	61	55	37	111	87	180	310	26	61	136	68	104	120	53	57	113	22	44	120	33	89	69	102	29	79
CTC_RS04425	938231..938458	0	104	58	67	101	151	0	35	117	181	282	523	95	64	0	90	111	0	37	0	105	0	0	0	35	49	102	30	215	168
CTC_RS04430	938486..938812	0	0	203	140	0	685	114	144	408	235	295	511	22	90	257	21	77	77	52	0	658	0	0	871	24	68	354	42	75	176
CTC_RS04435	938958..939809	16	223	94	45	215	81	11	222	31	42	189	112	42	137	74	64	118	163	40	57	28	29	307	96	19	157	27	32	115	90
CTC_RS04440	939815..940249	0	55	61	0	105	40	64	90	0	54	148	110	50	135	145	0	174	87	20	222	165	29	86	0	36	128	160	16	0	132
CTC_RS04445	940357..941379	0	23	13	15	67	152	46	77	78	29	31	233	49	129	21	47	74	25	71	47	0	36	182	40	23	44	113	47	72	56
CTC_RS04450	941506..943563	125	161	207	189	301	327	122	306	182	60	648	371	186	228	173	108	221	172	136	494	349	193	236	257	129	196	158	104	143	102
ablA	complement(943582..944856)	435	167	344	434	126	514	391	49	230	908	170	225	266	103	181	347	79	457	187	76	244	438	88	287	330	132	473	380	154	586
CTC_RS04460	complement(944872..945651)	69	0	153	73	29	199	102	0	68	613	41	31	19	38	54	52	32	146	38	0	61	32	0	157	31	29	59	106	0	455
CTC_RS04465	945865..946962	455	454	472	334	418	581	399	172	487	659	322	478	237	227	325	292	345	311	209	132	370	396	238	538	267	285	253	288	201	899
CTC_RS04470	947049..947684	297	112	376	300	288	732	440	124	420	613	404	338	148	230	363	284	238	159	214	0	339	351	235	640	200	246	255	162	116	332
CTC_RS04475	947821..948492	20	141	336	23	34	51	0	211	159	62	120	71	54	174	250	0	75	132	19	0	0	18	0	61	12	249	242	0	110	171
CTC_RS14175	948820..949518	0	0	19	22	0	49	7	23	0	84	23	0	21	21	30	10	0	36	24	0	34	0	0	0	23	0	33	39	35	27
CTC_RS04485	complement(950007..950678)	0	0	0	0	0	0	0	0	0	9	0	0	0	0	31	0	0	19	0	0	0	0	0	0	0	0	0	0	0	0
CTC_RS04490	950794..951276	0	0	28	0	0	36	0	0	0	12	0	0	0	0	0	0	52	52	0	0	0	0	77	0	0	0	0	0	0	0
CTC_RS04495	951273..951992	0	0	18	0	0	0	0	0	0	0	0	0	10	0	0	0	35	18	12	0	0	0	0	0	0	0	0	10	0	53
CTC_RS04500	952044..952232	0	0	0	40	0	0	0	0	0	0	0	0	0	0	0	72	133	67	45	0	0	0	0	0	0	0	0	0	0	0
CTC_RS04505	952258..952722	0	0	0	82	99	74	20	0	0	89	104	0	0	0	45	44	0	245	9	0	51	0	80	0	0	0	0	44	26	165
splB	952968..953990	0	0	13	7	0	0	0	0	0	6	63	0	0	0	0	0	0	0	0	0	0	0	36	40	0	33	0	0	0	0
nhaC	954324..955751	0	100	102	5	64	109	0	77	94	149	34	33	10	113	73	0	71	106	9	34	101	9	183	86	3	133	146	19	86	121
CTC_RS04520	955793..956671	15	189	204	17	209	196	11	179	304	67	110	190	8	100	143	15	29	173	10	165	218	0	255	139	0	312	396	86	251	414
CTC_RS14975	complement(956704..956949)	0	0	0	0	0	0	0	0	0	0	0	0	0	0	0	0	0	0	0	0	0	0	0	0	0	0	0	0	0	0
CTC_RS04530	957425..957838	0	0	32	18	55	83	90	38	65	200	39	288	35	35	101	165	61	138	51	117	58	30	180	98	38	108	112	158	59	46
CTC_RS04535	957878..958888	67	47	13	38	45	51	37	31	26	53	40	71	29	43	145	101	150	100	29	48	166	49	0	0	8	11	69	48	24	19
CTC_RS04540	958989..960494	18	63	97	25	46	114	31	0	18	82	0	111	29	19	84	86	50	76	54	96	191	33	74	0	5	45	62	64	16	38
CTC_RS04545	960509..961477	42	122	41	63	47	205	19	0	83	98	50	25	15	0	130	127	52	183	75	50	198	13	154	210	25	46	144	156	0	119
CTC_RS04550	961532..962506	679	316	109	282	329	495	239	307	247	351	165	171	333	300	151	545	362	377	467	447	638	318	230	209	244	390	238	219	151	118
CTC_RS04555	962558..963166	388	351	306	338	489	991	230	181	44	504	158	78	261	144	620	559	829	437	527	635	668	448	429	334	417	275	533	293	121	220
rbsK_1	963183..964103	484	309	159	307	324	730	233	137	203	500	175	518	235	159	228	333	192	289	291	578	805	202	324	486	138	61	352	261	107	219
CTC_RS14180	964188..965216	0	0	26	15	56	117	18	0	26	52	16	23	7	14	41	26	0	25	37	0	46	0	109	79	15	33	0	7	24	37

CTC_RS04570	965238..965723	14	0	0	16	47	35	19	0	0	0	66	49	0	0	0	0	0	0	9	0	98	0	0	0	0	0	0	0	37	0
CTC_RS04575	965997..966662	20	36	40	103	69	26	98	95	40	80	97	108	22	66	31	72	114	76	32	73	144	56	56	122	60	84	70	52	37	29
CTC_RS04580	966707..967690	0	0	14	31	23	53	0	48	0	6	65	24	0	15	43	28	103	0	9	0	49	0	38	0	8	34	0	7	25	39
CTC_RS04585	967874..969628	39	203	76	143	235	147	21	81	46	111	73	54	16	125	48	39	0	115	132	138	286	57	223	220	14	127	66	4	56	131
CTC_RS04590	969630..971354	8	96	39	111	252	110	5	64	0	84	37	83	4	119	12	32	29	125	76	0	28	50	216	212	9	104	27	8	57	56
CTC_RS04595	complement(971529..972089)	0	0	24	0	0	0	0	28	48	0	0	0	0	0	0	0	45	45	8	0	0	0	0	0	0	0	0	0	0	0
CTC_RS04600	complement(972536..973894)	0	0	0	6	17	13	0	0	0	7	47	184	0	0	0	0	19	37	0	0	0	0	0	0	0	0	0	0	18	42
CTC_RS04605	974050..974418	0	0	0	83	124	0	0	0	0	0	0	0	0	0	0	0	0	0	0	0	0	0	0	0	0	0	0	0	0	0
CTC_RS04610	974498..975592	0	22	0	7	0	0	0	0	0	0	15	0	0	0	0	0	0	0	0	0	0	0	0	0	0	0	0	0	0	0
CTC_RS04615	975595..976845	0	0	0	6	0	0	0	0	0	0	0	0	0	0	0	0	0	0	0	0	0	0	0	0	0	0	0	0	0	0
CTC_RS04620	976848..977513	0	0	0	23	0	0	0	0	0	0	0	0	0	0	0	0	0	0	0	0	0	19	0	0	0	0	0	0	0	0
CTC_RS14455	complement(977695..978077)	0	31	52	0	0	45	0	41	70	0	0	0	0	0	0	0	33	99	0	0	0	32	0	0	0	0	30	0	0	50
CTC_RS04630	978387..979013	0	0	21	0	0	0	15	0	0	0	26	0	0	70	0	0	0	0	7	0	0	0	0	65	0	36	37	0	0	0
CTC_RS04635	979108..979488	0	0	35	0	0	0	0	0	0	31	0	0	0	0	0	0	0	0	22	0	0	0	98	107	0	88	61	0	0	0
CTC_RS04640	979678..980580	187	79	44	85	51	95	83	35	30	654	89	185	24	32	0	83	56	140	106	161	132	96	0	135	105	99	77	495	0	42
CTC_RS04645	981122..983131	34	24	66	11	23	51	65	23	27	138	56	71	43	36	21	10	100	76	36	0	24	12	56	61	75	22	12	62	37	0
CTC_RS04650	983277..984467	0	0	22	6	0	0	0	26	0	15	13	0	6	0	0	0	0	0	0	0	20	0	0	0	0	0	0	6	0	16
CTC_RS04655	984561..985781	0	19	22	0	19	14	31	0	22	19	26	39	0	0	34	6	21	10	24	40	39	10	0	0	6	37	19	6	40	78
CTC_RS04660	985778..987043	21	0	11	0	0	14	15	0	0	33	25	38	11	0	17	16	20	30	24	0	0	0	0	0	0	0	0	11	0	30
CTC_RS04665	987116..987337	0	0	0	0	0	0	0	0	0	0	0	0	0	0	0	0	0	0	0	0	0	0	0	0	0	0	0	0	0	0
CTC_RS04670	987337..988797	0	16	18	10	63	24	6	32	18	129	22	49	30	0	14	23	35	43	35	33	65	17	0	28	16	0	0	42	17	13
CTC_RS04675	988834..989532	0	0	0	44	0	49	13	0	0	110	0	0	0	0	0	19	36	0	43	0	0	0	0	0	57	32	66	10	0	27
CTC_RS04680	989533..990438	0	0	0	0	0	19	21	17	0	20	71	0	8	16	69	8	56	0	14	53	0	0	0	0	26	12	0	23	108	42
CTC_RS04685	990525..990803	0	0	0	0	0	0	0	0	0	0	0	0	0	0	0	0	0	0	0	0	0	0	0	0	0	0	0	0	0	0
CTC_RS04690	990985..991356	0	0	0	0	0	0	0	0	0	16	0	32	19	0	0	0	0	0	11	0	0	0	0	0	0	0	0	37	66	52
CTC_RS14695	991363..991509	0	0	0	0	0	0	0	0	0	0	0	0	0	0	0	0	0	0	0	0	0	0	127	0	0	0	0	0	0	0
CTC_RS04695	991598..993019	86	83	28	38	64	12	46	133	0	96	102	17	20	206	74	38	71	71	33	34	0	9	105	29	84	157	16	5	86	27
CTC_RS04700	993066..994313	43	171	43	37	73	83	90	76	64	66	142	77	81	70	34	22	40	30	24	39	0	60	90	0	64	125	56	11	138	15
CTC_RS04705	994497..995717	77	58	152	175	94	268	107	206	153	203	197	117	124	96	17	89	62	187	73	40	137	91	92	333	58	110	114	101	40	157
CTC_RS04710	995774..996511	110	129	72	72	31	163	152	107	0	88	87	65	88	119	114	55	154	172	58	66	130	50	101	110	43	91	63	102	67	104
CTC_RS04715	996725..999136	11	305	221	92	223	172	12	170	177	12	20	30	42	255	104	20	52	210	11	20	30	31	124	51	23	236	125	6	71	381
CTC_RS04720	999210..1000142	0	51	57	16	49	18	0	17	29	13	17	0	8	63	67	0	0	41	9	52	0	0	0	0	0	72	50	0	26	0
CTC_RS04725	complement(1000265..1000999)	0	0	0	0	0	23	0	0	0	16	0	0	0	0	0	0	0	0	0	0	49	0	0	0	0	0	0	0	0	0
CTC_RS04730	complement(1001000..1002040)	0	0	0	0	22	17	0	0	0	11	0	0	0	0	0	0	0	0	0	0	0	0	0	0	0	0	0	0	0	37
CTC_RS04735	complement(1002063..1002698)	0	0	0	0	0	0	15	0	0	0	0	0	0	0	0	0	0	0	0	0	0	0	0	0	0	0	0	0	0	0
CTC_RS04740	complement(1002719..1003678)	0	0	0	0	0	18	0	0	0	0	0	0	0	15	22	0	0	13	0	0	75	0	0	42	0	0	0	0	0	0
CTC_RS04745	1004043..1006124	0	0	19	0	0	0	0	0	0	6	0	34	3	0	0	3	0	6	10	0	0	6	0	0	0	5	11	7	0	0
CTC_RS04750	1006356..1007291	332	76	114	41	49	129	309	34	86	259	927	153	77	94	45	189	27	54	91	258	77	53	0	87	178	96	25	257	210	20
CTC_RS04755	1007319..1007969	332	0	61	59	35	106	358	24	41	299	420	183	144	45	32	178	78	175	65	223	147	248	115	63	292	189	178	380	151	147
CTC_RS04760	1008007..1009059	488	113	114	127	87	65	337	30	76	236	595	159	212	56	60	272	216	132	101	184	45	200	142	135	324	117	110	222	140	127
mtnK	1009091..1010302	491	78	159	246	274	156	458	195	220	258	464	118	262	205	121	458	354	198	209	579	217	184	92	269	344	166	268	306	263	190
CTC_RS04770	1010564..1011931	0	121	39	33	168	50	7	46	78	246	587	87	0	171	61	119	148	56	25	0	17	18	55	30	6	90	102	5	36	14
CTC_RS04775	1012080..1012898	182	405	195	224	364	379	387	519	457	79	118	58	220	179	192	191	92	62	405	118	117	273	273	50	145	184	198	243	270	304
CTC_RS04780	complement(1012943..1014613)	0	0	0	9	14	10	0	0	0	4	10	0	0	0	0	0	0	0	5	0	14	0	0	0	0	0	0	0	15	0
CTC_RS04785	complement(1014662..1015366)	0	0	0	0	0	49	0	0	0	17	0	0	0	0	0	0	0	0	0	0	68	0	0	0	0	16	0	0	0	0
CTC_RS04790	1015561..1017318	23	0	23	0	26	29	5	0	0	27	0	41	4	0	0	0	0	0	29	0	0	7	0	46	5	0	40	188	0	11
CTC_RS04795	1017334..1018062	19	0	18	0	0	24	13	22	0	36	0	33	20	20	29	9	0	17	58	66	66	17	51	56	0	15	64	500	51	26
CTC_RS04800	complement(1018111..1018881)	53	31	17	10	59	22	0	20	35	100	63	0	9	76	27	18	0	0	44	0	0	0	0	0	0	14	60	196	32	50
CTC_RS04805	complement(1018882..1019949)	13	0	0	0	21	16	9	0	0	77	15	45	7	0	20	26	0	12	12	0	22	12	0	0	0	21	0	45	0	18
CTC_RS04810	complement(1019961..1020983)	26	23	104	7	22	17	18	46	0	113	31	0	14	0	0	13	0	0	33	0	0	24	0	40	23	22	23	104	48	75
CTC_RS04815	1021323..1022063	18	320	520	0	124	256	13	425	288	80	22	113	10	198	170	9	681	701	17	65	0	0	50	55	32	422	250	306	994	207
CTC_RS04820	complement(1022207..1024330)	0	0	0	7	0	8	4	0	0	19	8	0	0	0	0	0	0	6	0	0	0	0	0	58	0	5	22	6	0	9
CTC_RS04825	1024881..1025945	38	67	12	14	280	227	9	44	50	227	30	45	14	41	39	32	47	59	88	0	22	23	35	172	15	31	0	71	46	18
CTC_RS04830	1025947..1027044	37	86	24	63	188	330	8	29	0	323	59	0	20	13	38	12	0	127	35	0	44	23	170	204	0	20	63	106	22	35
CTC_RS04835	1027060..1027818	18	31	35	10	181	159	12	41	0	377	0	94	19	19	0	18	0	50	34	0	95	16	0	161	21	0	0	154	0	51
CTC_RS04840	1027836..1028159	0	73	0	0	71	0	29	146	0	365	0	74	22	0	0	21	0	78	13	149	222	0	115	0	0	0	0	64	0	0
CTC_RS04845	complement(1028210..1028563)	0	0	0	0	65	0	0	0	75	0	0	135	0	0	59	19	0	0	0	0	0	0	0	0	0	0	0	0	0	0
CTC_RS04850	complement(1028608..1028874)	0	0	50	29	0	0	35	59	100	199	0	0	27	55	0	77	0	47	96	0	0	93	280	0	59	0	174	232	0	0
CTC_RS04855	1029104..1031137	0	87	85	8	0	25	18	77	53	15	198	35	28	72	15	10	12	25	4	0	0	6	18	10	19	85	46	5	48	94
argS	1031452..1033143	56	56	47	59	122	41	44	74	47	241	114	113	77	61	62	56	67	90	122	29	71	59	66	168	66	59	55	138	73	91
CTC_RS04865	1033253..1034047	34	179	117	67	144	228	35	20	134	74	61	180	36	55	26	51	95	0	43	0	60	47	282	51	40	70	146	43	31	96
CTC_RS14460	complement(1034081..1034239)	85	298	335	192	288	325	176	0	168	520	101	150	91	92	132	343	0	80	134	0	150	78	0	0	50	0	146	216	154	1025
feoB_2	complement(1034253..1036016)	138	269	475	407	500	1031	249	299	575	2464	164	595	104	116	297	554	114	876	298	137	563	179	423	1096	148	165	237	850	230	1478
CTC_RS04875	complement(1035991..1036269)	969	2040	2526	916	2711	2904	1337	1410	2681	2847	1786	2738	1138	2256	2255	1880	995	3131	1189	867	1458	845	2140	3940	540	1643	3075	2045	2639	5084
CTC_RS04880	1036524..1037258	55	129	145	166	281	258	165	236	291	386	350	357	157	319	342	250	481	327	243	132	33	101	152	222	151	335	315	281	167	365
CTC_RS04885	1037282..1037929	63	73	82	47	141	186	86	24	41	173	74	147	45	113	129	121	39	313	151	224	222	57	0	440	12	121	72	106	76	266
CTC_RS04890	1037992..1038687	49	307	153	208	132	99	114	136	154	178	185	343	83	252	301	245	145	273	287	104	69	71	0	58	80	225	167	257	141	220
CTC_RS04895	1038823..1040343	178	405	472	181	678	918	166	331	650	8	586	942	62	674	758	228	1476	275	42	2163	3452	196	994	749	68	323	564	9	274	466
CTC_RS04900	1040392..1041180	163	842	1070	164	1351	1376	160	559	643	0	1324	2481	46	1169	1409	380	2766	682	22	3740	4791	236	1845	1084	75	524	1792	35	280	437
CTC_RS04905	1041206..1042405	68	613	920	121	1184	1178	23	426	646	20	844	1472	24	671	1023	199	2228	570	18	2459	3050	134	1166	916	40	326	792	23	184	423
CTC_RS04910	1042424..1043833	62	555	1198	76	1479	1528	33	558	1023	25	1129	1837	41	851	1041	164	2764	557	21	4117	4131	53	1403	1559	34	285	1135	39	574	856
gltX	complement(1043876..1045342)	175	65	63	177	109	106	153	0	91	678	99	358	148	60	43	163	17	155	152	66	82	173	0	139	84	38	63	216	50	78
CTC_RS04920	complement(1045533..1047053)	302	437	525	587	949	691	371	497	509	210	867	753	356	462	276	345	597	524	307	763	802	498	1104	589	302	503	351	199	710	693
CTC_RS04925	1047217..1048122	0	0	15	0	0	0	0	35	0	20	0	0	0	0	0	23	0	0	5	0	26	14	0	0	0	12	0	0	27	0
CTC_RS04930	complement(1048156..1049415)	21	113	106	42	55	246	207	125	170	567	13	114	52	70	117	59	100	201	152	0	38	10	59	81	13	106	55	226	78	145
CTC_RS04935	1049554..1050177	43	38	21	12	147	83	0	151	0	66	52	0	12	0	0	11	0	0	14	0	192	0	0	65	13	90	37	44	0	0
CTC_RS04940	1050297..1050518	0	0	180	34	103	0	84	71	0	160	145	0	33	66	0	31	0	171	19	0	108	112	0	0	0	0	104	0	0	173
CTC_RS04945	complement(1050562..1052043)	119	80	215	154	263	395	44	117	162	397	119	274	93	148	142	115	119	312	164	131	323	100	327	494	64	68	141	158	99	116
CTC_RS04950	1052170..1054176	0	0	0	4	0	0	0	16	0	6	40	12	0	0	0	7	0	6	8	0	0	6	0	0	4	6	0	0	37	29
CTC_RS04955	1054282..1056333	13	23	26	52	45	25	50	8	0	43	31	12	25	43	10	53	12	62	70	24	23	36	0	0	21	22	17	47	48	9

CTC_RS04960	1056371..1057333	0	25	41	40	0	0	48	0	28	67	50	25	52	15	0	71	26	39	66	25	50	26	0	0	8	35	0	43	51	20
CTC_RS04965	1057352..1058959	50	30	33	95	71	75	29	29	66	173	50	52	27	36	26	42	47	47	66	0	89	31	0	51	39	28	43	73	15	48
spoVB	complement(1058991..1060481)	0	0	0	0	0	0	0	11	0	0	11	0	0	10	0	0	0	8	0	0	0	0	0	0	0	0	0	0	0	0
CTC_RS04975	1060627..1061187	0	0	71	88	82	123	50	28	48	105	57	128	13	52	0	49	90	181	53	86	213	44	67	0	14	60	41	61	0	68
CTC_RS04980	1061281..1062009	111	260	201	209	314	284	128	259	110	340	397	65	109	201	86	121	104	174	163	398	558	136	154	223	82	245	159	207	168	316
CTC_RS04985	1062115..1062627	0	46	26	0	45	0	55	0	0	0	31	47	28	29	0	27	0	25	25	0	47	24	0	0	15	0	0	13	0	37
CTC_RS04990	1062642..1064372	23	14	38	31	26	20	43	0	0	51	9	0	25	34	24	39	29	59	37	84	69	14	22	71	11	19	27	12	0	33
CTC_RS04995	1064504..1064830	413	653	468	1050	631	316	742	481	163	885	295	0	662	448	641	1510	154	697	1073	0	805	607	685	249	825	581	213	862	676	1114
CTC_RS05000	1064817..1066787	672	481	175	1009	558	402	833	459	312	1699	188	170	597	490	282	1310	218	1134	972	368	789	929	454	289	614	476	223	966	274	705
CTC_RS05005	1066802..1067275	1568	951	421	1586	1040	982	1338	996	676	2112	746	453	982	803	620	2507	639	2150	1709	816	1741	1780	1614	1546	1196	743	538	1407	570	465
CTC_RS05010	1067312..1067908	294	278	111	550	288	289	469	211	224	1064	242	200	478	466	246	782	296	763	637	162	701	488	313	273	505	356	272	518	41	417
CTC_RS05015	1067922..1068923	283	118	119	453	297	516	279	298	80	1082	144	191	151	234	105	364	126	531	265	193	477	235	112	609	198	145	93	377	147	268
CTC_RS05020	1068910..1069224	644	678	169	933	800	109	800	450	254	1087	459	227	642	674	666	1341	240	1306	1060	154	759	867	711	1034	856	390	736	655	701	243
CTC_RS05025	1069243..1071015	930	743	251	1166	672	544	818	644	422	1880	290	377	666	900	473	1406	647	1875	1110	1037	1336	756	831	482	939	867	379	1311	623	703
CTC_RS05030	1071008..1072393	775	770	278	1104	728	585	895	596	337	1743	383	551	628	929	409	1278	728	1644	982	1274	1320	877	754	470	807	863	401	1089	868	415
CTC_RS05035	1072408..1073046	634	520	312	1075	538	513	569	739	125	1044	755	560	587	344	525	767	948	1486	532	606	674	757	526	319	459	1015	544	1173	615	540
CTC_RS05040	1073192..1073596	4154	1230	2463	5483	1670	3681	12128	1244	3561	190	3750	2888	7458	542	5852	4625	1059	94	4414	10391	3662	7000	2396	3518	7490	552	4522	272	1545	1420
CTC_RS05045	1073733..1074233	0	0	133	15	46	34	0	31	0	0	64	0	14	0	42	0	0	101	8	193	95	0	0	81	0	22	46	0	245	153
CTC_RS05050	1074521..1075696	287	595	277	513	448	748	222	415	250	477	164	183	252	162	143	371	279	301	544	370	488	390	508	519	236	390	217	275	334	668
CTC_RS05055	1075753..1076103	0	135	76	22	457	98	80	45	304	151	275	68	82	334	120	78	144	252	230	0	136	71	0	116	113	287	0	196	350	55
CTC_RS05060	1076136..1076693	48	255	71	0	246	371	33	113	96	64	115	86	13	79	75	98	136	159	69	173	300	0	334	219	28	20	83	74	220	69
glpX	1076938..1077909	42	195	192	39	165	106	19	194	55	55	248	147	22	136	259	63	234	143	70	100	74	64	77	42	24	69	95	71	177	217
CTC_RS05070	1078073..1079860	113	358	186	239	410	265	110	299	134	320	198	334	190	205	141	236	212	326	295	271	361	201	292	148	133	250	169	158	151	150
hprK	1079898..1080815	59	90	72	83	75	75	61	103	29	39	123	104	55	32	114	59	55	69	72	211	0	34	122	44	69	61	76	37	27	42
CTC_RS05080	1080834..1081385	0	86	48	41	83	31	0	57	145	43	0	0	78	27	38	74	0	69	31	88	152	0	0	0	14	81	84	0	44	0
CTC_RS05085	1081442..1082842	96	339	180	231	262	271	60	270	114	194	161	136	227	251	105	292	162	334	161	207	290	168	107	203	209	279	116	128	333	328
CTC_RS05090	complement(1083040..1083909)	16	0	15	26	26	0	0	0	0	48	0	0	17	0	0	23	116	44	0	0	55	29	0	47	9	39	0	32	28	0
CTC_RS05095	1084283..1084630	0	0	191	88	66	198	0	271	77	17	46	206	0	379	60	0	290	255	0	0	69	0	107	117	0	225	67	0	776	220
CTC_RS05100	1084725..1085048	0	2050	6690	24	283	1224	0	2866	4781	0	1141	1694	0	5106	6506	21	16975	6096	0	448	295	38	922	503	0	6796	6404	0	4091	2781
CTC_RS05105	complement(1085140..1085616)	0	0	28	0	0	0	0	33	0	0	67	0	0	31	0	0	0	0	0	0	0	0	0	0	0	0	0	0	0	0
CTC_RS05110	1086018..1086287	0	88	0	0	0	64	69	58	0	0	179	354	0	0	155	76	0	188	94	179	0	0	0	0	0	0	86	51	91	213
CTC_RS05115	complement(1086342..1086788)	60	0	30	102	154	39	63	35	60	66	36	53	16	0	47	61	56	28	10	0	107	56	83	91	0	0	0	15	55	129
CTC_RS05120	complement(1086785..1087555)	18	0	17	59	149	112	24	0	35	107	63	62	37	57	109	18	0	66	22	0	124	16	24	0	10	29	0	9	96	75
rsxC	1087927..1089234	362	290	173	228	298	329	221	193	123	549	197	201	232	201	128	479	193	319	491	296	567	294	228	280	182	171	89	392	188	234
CTC_RS05130	1089255..1090187	550	559	299	450	393	406	230	363	372	709	241	614	487	126	79	526	433	556	768	311	641	346	740	633	319	228	248	578	500	390
CTC_RS05135	1090189..1090758	593	416	257	576	282	242	213	442	234	466	282	419	380	437	221	586	443	222	1059	1018	630	392	589	429	362	373	81	880	388	404
CTC_RS05140	1090761..1091363	695	315	176	582	456	672	186	261	221	1244	293	277	299	146	104	373	251	493	649	562	754	473	371	675	342	278	192	838	529	556
rsxA	1091410..1091985	610	618	358	437	756	853	162	683	487	1328	279	539	451	330	218	508	526	637	528	840	1412	474	778	919	234	310	382	1134	533	333
CTC_RS05150	1092010..1092882	1718	1005	647	953	1313	1007	684	929	780	2094	1215	1531	914	838	961	1123	1445	1798	1602	2216	2357	860	1667	1259	777	1011	1049	2815	1476	1197
CTC_RS05155	complement(1092919..1093449)	0	89	0	0	43	32	18	0	50	78	30	0	0	0	0	38	0	48	24	0	45	0	70	0	0	0	0	13	46	36
CTC_RS05160	complement(1093465..1093863)	34	0	0	19	0	130	0	0	0	89	0	0	18	0	0	17	0	32	21	0	0	0	94	102	0	56	58	86	0	0
CTC_RS05165	complement(1093850..1094878)	53	46	26	119	22	0	45	76	26	212	31	46	35	28	61	66	0	86	107	141	46	24	36	158	15	22	68	107	119	56
CTC_RS05170	complement(1095152..1097023)	0	0	0	8	0	0	0	0	14	22	0	0	0	0	0	7	0	7	7	0	0	0	0	0	0	0	12	7	0	31
CTC_RS05175	1097207..1100983	4	50	18	22	12	23	67	46	14	34	43	63	19	31	39	18	53	74	27	26	13	13	10	11	17	18	12	9	26	76
CTC_RS05180	1101063..1101674	0	78	43	12	37	28	15	77	0	10	105	78	0	120	69	22	165	83	14	79	39	0	0	67	0	18	114	34	80	94
CTC_RS05185	1102034..1102675	21	0	104	12	36	81	0	74	250	101	25	37	39	23	98	0	0	99	13	0	37	19	0	63	12	52	36	21	115	90
CTC_RS05190	1102665..1103372	57	67	19	43	97	97	53	89	151	58	45	101	71	103	119	115	143	161	108	137	169	35	105	0	11	32	33	58	520	27
CTC_RS05195	1103511..1103780	50	0	0	0	170	0	0	117	99	44	0	88	0	0	0	50	93	188	205	179	89	0	0	0	59	124	172	102	182	213
CTC_RS05200	1103797..1105155	149	227	98	168	135	89	103	151	138	174	83	193	74	237	93	261	204	410	357	107	88	55	55	90	123	197	85	298	325	197
CTC_RS05205	1105168..1105674	160	0	184	151	226	68	18	31	53	186	63	24	114	217	41	27	199	75	109	382	94	24	221	80	109	176	0	68	145	76
CTC_RS05210	complement(1105724..1106788)	679	668	606	964	796	1133	631	517	1028	998	1094	695	583	522	315	742	521	773	421	727	1190	944	1156	765	428	425	457	788	737	1260
ltaE	complement(1106909..1107934)	553	358	454	863	715	554	555	614	677	127	1097	651	749	799	675	624	369	555	557	707	548	937	691	397	827	686	497	174	215	467
CTC_RS05220	1108103..1109992	0	126	7	0	73	9	5	121	14	0	285	0	0	302	44	4	240	27	0	51	13	0	79	22	0	251	18	4	65	0
yhbH	1109996..1111156	0	123	0	7	79	74	8	54	46	15	263	62	6	139	36	0	65	22	4	83	0	0	32	35	0	77	40	6	42	33
CTC_RS05230	1111153..1112355	0	39	33	6	38	72	23	52	0	25	214	0	0	134	0	11	42	32	0	0	40	0	31	0	0	139	0	6	41	64
CTC_RS05235	complement(1112395..1113681)	399	479	537	477	695	710	449	587	560	312	637	519	252	455	326	529	755	925	401	1015	1069	540	522	554	339	426	198	278	610	804
proB	complement(1113702..1114505)	303	502	744	389	584	472	354	313	432	345	540	445	287	200	535	474	816	803	286	421	922	201	557	911	325	264	173	137	92	405
CTC_RS05245	1114883..1115914	13	23	0	0	0	0	9	15	0	0	0	12	7	0	20	13	24	49	33	0	23	0	0	0	0	0	22	13	0	0
CTC_RS05250	1115970..1116746	35	31	0	20	0	0	48	0	0	53	0	0	0	0	0	44	65	0	11	0	0	0	144	52	0	14	30	18	0	49
CTC_RS05255	1116761..1117255	0	0	27	31	0	70	38	64	54	24	32	48	80	59	0	21	102	77	43	0	0	0	0	0	0	45	0	42	0	39
CTC_RS05260	1117281..1118564	0	37	21	12	18	13	7	0	21	32	50	19	0	34	16	32	39	39	36	38	37	19	0	63	12	9	0	48	19	90
CTC_RS05265	1118697..1119131	1491	1091	1162	1631	1686	1109	1952	1230	2211	5125	1275	713	664	1043	1591	3045	783	2707	4208	2780	2640	941	1459	3463	711	1054	1439	4259	2144	1807
nifS	1119134..1120336	2157	1597	1194	2604	1734	1418	2040	1747	1810	6454	1243	1012	1005	1241	1508	5271	1111	3105	5929	2252	3629	1815	2637	2877	1503	1254	1696	5235	1387	2446
nifU	1120342..1120770	1071	442	465	1032	641	442	413	550	436	1452	225	501	336	546	929	1810	588	856	1571	564	1004	521	696	759	573	261	378	2548	515	1564
mnmA_1	1120836..1121921	174	175	147	232	274	143	77	203	172	789	104	88	53	121	290	640	232	350	769	134	463	206	241	300	113	154	192	1288	316	512
CTC_RS05285	1121993..1122490	0	0	0	0	0	0	0	32	0	12	0	0	14	0	0	0	0	0	17	0	0	0	0	0	0	22	47	14	0	0
CTC_RS05290	1122483..1123496	0	0	26	0	0	0	0	16	0	6	0	0	0	0	0	0	0	6	0	0	0	0	37	0	0	0	0	0	24	0
alaS	1123799..1126438	72	54	15	127	61	39	71	60	30	208	85	54	137	100	64	168	67	101	151	73	27	103	71	62	129	74	61	129	93	44
CTC_RS05300	1126523..1126774	912	1788	3483	1454	2729	2941	925	2811	4876	1336	7431	6156	974	4124	6159	1595	5806	2261	1164	4031	2658	1477	2073	3069	818	2528	5198	1091	3701	2700
ruvX	1126830..1127246	681	1081	989	1025	1869	1591	1007	1057	1025	1742	1311	1202	433	843	1157	931	847	1214	882	464	1377	684	940	1562	799	1206	945	1236	912	1655
CTC_RS05310	1127266..1127535	425	2284	2807	1371	3099	2809	1002	2449	3858	1192	4524	3182	521	2657	3574	757	3831	1594	693	2150	2126	1011	2765	2563	558	1904	2404	1044	1818	2982
CTC_RS05315	1127653..1128117	378	408	143	427	246	482	221	203	172	495	242	411	217	189	226	403	380	463	590	520	566	347	161	525	153	409	349	503	106	206
CTC_RS05320	1128164..1129831	506	569	375	814	618	620	607	472	432	952	337	901	517	491	390	768	318	592	1011	725	617	692	671	366	452	684	459	1092	361	632
CTC_RS05325	1129916..1130266	347	473	493	348	784	295	346	404	609	707	550	408	370	292	299	602	288	289	388	0	613	424	425	232	226	255	429	333	629	819
typA	1130388..1132214	89	208	109	286	151	127	107	224	117	357	132	183	154	176	80	149	110	152	398	79	131	115	102	145	65	202	165	453	81	199
mltG	1132232..1133251	0	0	0	67	67	34	9	62	26	151	0	47	35	14	0	40	0	37	71	0	23	49	0	80	8	11	0	108	48	19
CTC_RS05340	1133269..1133916	21	0	21	177	0	27	0	49	41	283	74	37	45	90	65	74	78	59	269	0	37	0	0	251	12	86	72	180	0	0
CTC_RS05345	1133919..1135136	33	78	66	169	38	71	31	52	44	204	66	98	6	36	86	45	21	62	187	0	59	20	61	167	20	55	0	198	0	31
udk	1135167..1135796	21	38	42	85	36	109	30	0	127	141	153	76	57	116	33	54	120	60	196	154	0	20	0	194	13	71	74	207	78	152
CTC_RS05355	1135881..1137494	0	15	16	19	0	11	0	0	0	7	10	30	0	27	0	0	16	0	3	0	15	0	23	25	0	7	0	9	15	24

CTC_RS05360	1137612..1138034	0	0	157	0	0	41	0	223	284	0	190	56	0	121	298	16	298	314	0	114	0	0	176	0	0	211	164	0	319	272
CTC_RS05365	complement(1138054..1139562)	90	0	26	215	30	23	12	0	18	0	0	0	5	0	0	14	17	42	20	32	16	206	74	0	5	0	15	9	16	25
CTC_RS05370	complement(1139585..1139869)	0	0	0	214	0	60	98	0	0	0	0	0	0	0	0	0	0	0	15	0	252	348	262	143	0	0	0	48	86	67
CTC_RS05375	complement(1139887..1140231)	118	69	0	265	0	375	81	0	155	0	0	0	0	42	0	79	0	37	12	140	381	216	0	118	0	0	0	0	0	111
CTC_RS05380	complement(1140258..1140716)	88	0	29	449	100	113	20	34	0	0	0	0	31	0	46	0	55	83	46	105	156	433	163	0	17	0	51	15	53	0
CTC_RS14190	complement(1140732..1141175)	15	0	90	172	52	39	11	0	120	0	0	0	33	33	142	31	0	43	10	0	54	168	42	138	45	0	104	8	0	22
CTC_RS05390	1141336..1141551	0	0	0	0	0	0	0	0	0	0	0	0	0	0	0	0	0	0	0	0	0	0	0	0	0	0	0	0	0	0
CTC_RS05395	1141567..1142370	622	148	0	3350	485	161	12	98	0	0	0	0	108	237	26	8	31	0	16	0	0	3758	580	76	54	264	43	98	92	60
CTC_RS05400	complement(1142403..1142804)	101	0	99	0	114	214	46	78	199	0	0	0	54	510	835	68	126	409	32	120	0	62	279	405	20	306	346	34	0	48
CTC_RS14980	1143191..1143622	657	110	0	2712	345	0	32	18	0	0	0	0	58	152	24	24	0	0	0	0	0	2456	518	94	55	181	54	8	114	44
CTC_RS05410	1143708..1145051	226	35	0	996	324	32	0	0	0	0	0	0	0	0	0	5	0	9	0	0	0	1112	236	30	0	0	0	0	0	0
CTC_RS05415	1145053..1145859	0	15	0	5	0	0	0	0	0	0	0	0	0	0	0	0	31	16	0	0	0	8	0	0	0	0	0	0	0	0
CTC_RS05420	1145852..1146031	0	0	0	0	0	0	0	0	0	0	0	0	0	0	0	0	0	0	0	0	0	0	0	0	0	0	0	0	0	0
CTC_RS05425	1146049..1146927	200	108	0	1294	209	39	0	0	0	0	0	0	16	25	0	0	29	14	0	0	0	1722	467	93	0	13	0	0	28	0
CTC_RS05430	1146940..1147314	108	0	0	315	153	0	25	0	0	0	0	0	77	273	0	0	0	34	0	0	0	347	149	0	42	134	31	55	262	0
CTC_RS05435	1147396..1147824	32	0	31	0	0	0	22	73	0	0	0	0	168	444	147	0	0	59	0	0	0	0	0	0	74	261	0	16	172	45
CTC_RS05440	1147821..1148450	21	0	0	0	0	0	30	200	42	0	0	0	92	186	0	11	0	0	0	0	0	0	0	0	120	266	0	11	273	0
CTC_RS05445	complement(1148625..1149065)	0	161	151	0	0	0	0	143	303	0	0	0	0	299	0	0	57	0	0	0	0	0	0	0	0	228	105	0	56	87
CTC_RS05450	1149550..1149711	0	0	0	47	0	213	0	0	0	0	0	0	0	0	0	0	0	0	0	0	0	0	0	0	0	0	0	0	0	0
CTC_RS05455	1149692..1149889	68	0	0	1330	232	261	0	318	0	0	0	0	73	0	424	0	0	0	0	0	0	1441	188	0	40	169	117	0	0	0
CTC_RS14700	1149892..1150032	0	0	0	0	0	0	0	0	0	0	0	0	0	0	0	0	0	0	0	0	0	0	0	0	0	0	0	0	0	0
CTC_RS05460	1150032..1150232	269	118	66	759	114	343	0	0	0	0	0	0	108	583	730	34	0	0	0	0	0	741	557	0	0	556	461	0	122	0
CTC_RS05465	1150270..1150581	173	684	597	1773	1212	773	30	757	342	0	0	0	116	469	740	44	81	41	0	0	0	1710	718	979	114	609	594	44	354	123
CTC_RS05470	1150593..1151060	29	51	57	0	0	0	40	471	228	0	0	0	0	0	0	29	0	0	0	0	0	0	0	0	0	0	0	0	0	0
CTC_RS14850	1151297..1151473	0	0	0	0	0	0	0	0	0	0	0	0	0	0	0	0	0	0	0	0	0	0	0	0	45	0	0	0	0	0
CTC_RS05475	1151705..1151947	83	293	27	298	283	638	0	0	0	0	0	0	0	0	0	0	0	0	0	0	0	332	154	419	0	0	0	0	0	0
CTC_RS14465	1152347..1152439	0	0	0	0	0	0	0	0	0	0	0	0	0	0	0	0	0	0	0	0	0	0	0	0	0	0	0	0	0	0
CTC_RS14195	1152545..1153633	137	22	49	231	42	63	51	87	74	0	0	0	0	0	0	231	46	81	0	0	0	80	69	112	0	0	0	13	68	53
CTC_RS05495	1153679..1154602	0	26	65	0	0	0	0	0	0	0	0	0	55	32	114	0	0	0	0	0	0	0	0	0	34	48	75	0	27	31
CTC_RS05500	1154592..1155935	10	35	20	0	0	0	0	0	0	0	0	0	21	22	78	0	0	0	0	0	0	0	0	0	41	8	43	10	18	57
CTC_RS05505	1155935..1157392	0	49	182	10	110	260	6	11	0	0	0	0	20	10	43	23	17	9	0	0	0	17	115	251	38	54	80	5	0	92
CTC_RS14200	1157449..1157922	0	150	168	16	145	182	20	0	0	0	0	0	15	31	133	7	53	80	0	0	0	79	79	86	33	71	293	0	155	81
CTC_RS05515	1157959..1159659	0	126	188	114	229	466	16	46	79	0	0	0	76	34	148	16	0	7	0	0	0	58	241	239	79	112	123	67	43	293
CTC_RS05520	1159656..1159856	0	472	265	76	342	172	0	157	0	0	0	0	180	0	313	0	0	0	0	0	0	62	186	405	39	167	231	171	122	1001
CTC_RS05525	1159920..1160120	0	0	0	0	0	86	0	0	0	0	0	0	0	0	104	0	0	0	0	0	0	0	371	203	0	0	0	0	0	0
CTC_RS05530	1160120..1160737	0	230	409	37	352	502	0	25	0	0	0	0	93	118	170	6	0	0	0	0	0	20	423	329	45	181	300	0	79	217
CTC_RS05535	1160752..1161084	0	285	40	0	551	311	28	47	0	0	0	0	43	44	315	20	0	0	0	0	0	0	112	245	48	269	209	0	0	0
CTC_RS05540	1161104..1162138	13	252	450	29	509	816	9	0	52	0	0	0	77	57	203	26	73	24	0	0	0	24	541	669	46	43	269	20	24	148
CTC_RS05545	1162194..1162382	0	0	0	0	0	0	0	0	0	0	0	0	38	155	0	0	0	0	0	0	0	66	0	215	42	0	123	0	0	0
CTC_RS05550	1162385..1162687	0	235	483	25	605	910	31	0	0	0	0	0	191	97	623	22	0	0	0	0	0	82	616	537	79	111	77	23	81	127
CTC_RS05555	1162684..1163049	0	259	182	42	438	565	0	43	0	0	0	0	79	80	115	19	0	0	0	0	0	34	510	890	43	153	0	0	0	0
CTC_RS05560	1163054..1163398	0	206	463	155	465	799	54	91	0	0	0	0	105	170	243	20	0	0	0	0	0	0	325	1298	69	97	202	40	71	111
CTC_RS05565	1163403..1163822	0	0	63	18	164	246	0	0	0	0	0	0	17	35	150	97	0	30	0	0	0	30	44	388	0	0	55	0	58	91
CTC_RS05570	1163827..1164699	15	82	366	17	210	434	0	18	61	0	0	0	74	67	108	39	58	58	0	0	0	0	299	513	50	51	66	0	112	11
CTC_RS05575	1164715..1165176	0	205	230	17	50	224	0	34	0	0	0	0	16	0	0	29	0	0	0	0	0	0	404	264	9	0	25	15	0	0
CTC_RS05580	1165343..1165669	83	218	122	23	210	474	0	0	0	0	0	0	22	0	0	0	0	0	0	0	0	0	0	0	0	0	142	0	75	0
CTC_RS05585	1165727..1167772	0	64	159	19	101	329	5	8	26	0	0	0	56	14	123	0	12	9	0	0	0	24	328	159	16	38	45	0	60	84
CTC_RS05590	1168161..1169258	0	22	85	7	125	173	0	14	0	0	0	0	33	13	19	0	0	0	0	0	0	0	34	74	14	0	42	6	0	70
CTC_RS05595	1169258..1169590	0	0	40	11	138	181	0	0	0	0	0	0	0	0	0	0	0	0	0	0	0	0	112	122	0	34	0	0	0	345
CTC_RS05600	1169603..1170163	0	42	71	27	0	92	17	28	0	0	0	0	26	0	0	12	0	23	0	0	0	44	266	218	14	40	0	0	88	68
CTC_RS05605	1170160..1171191	0	23	26	0	111	117	0	15	0	0	0	0	21	14	20	0	0	0	0	0	0	0	36	39	23	0	22	0	0	37
CTC_RS05610	1171194..1172429	0	77	129	12	148	181	0	0	0	0	0	0	58	12	119	17	0	0	0	0	0	10	91	231	32	36	19	0	119	31
CTC_RS05615	1172442..1174334	0	38	42	4	36	155	0	8	0	0	0	0	19	15	11	14	0	0	0	0	0	7	0	43	4	18	12	7	0	0
CTC_RS05620	1174339..1174593	106	0	156	0	90	203	0	185	0	0	0	0	0	0	165	0	0	0	0	0	0	0	0	160	187	88	182	0	0	75
CTC_RS05625	1174609..1175607	0	71	40	8	46	86	0	0	0	0	0	0	51	0	42	7	0	0	0	0	0	0	37	41	16	0	0	0	0	19
CTC_RS05630	1175618..1176700	0	22	104	7	254	318	9	0	0	0	0	0	20	27	0	13	0	35	0	0	0	11	69	150	7	10	21	0	0	18
CTC_RS14205	1176776..1177561	0	0	59	10	73	121	12	0	0	0	0	0	9	37	27	17	0	16	0	0	0	32	0	0	20	14	29	0	31	73
CTC_RS05640	1177611..1178489	0	0	0	9	0	0	0	0	0	0	0	0	0	0	0	0	0	0	0	0	0	0	0	0	0	0	0	0	0	0
CTC_RS05645	1179054..1179269	0	0	0	35	0	80	0	0	0	0	0	0	0	0	0	0	0	0	0	0	0	57	0	0	0	0	0	32	0	0
CTC_RS05650	1179688..1179921	0	101	114	33	392	258	40	0	0	0	0	0	46	63	359	0	0	0	0	0	0	0	319	0	34	48	495	0	105	0
CTC_RS05655	1179939..1180202	0	0	50	0	0	0	0	0	0	0	0	0	0	0	0	0	0	0	0	0	0	0	0	0	0	0	0	0	0	0
CTC_RS14480	1180203..1181003	0	30	83	0	86	398	0	0	17	0	0	0	0	9	0	26	0	0	0	0	0	0	140	76	0	7	0	0	46	12
CTC_RS05670	1181236..1182288	0	0	0	0	0	0	0	0	0	0	0	0	0	0	0	0	0	0	0	0	0	0	0	0	0	0	0	0	0	0
CTC_RS05675	1182272..1182604	0	0	0	0	0	0	0	0	0	0	0	0	0	0	0	0	0	0	0	0	0	0	0	0	0	0	0	0	0	0
CTC_RS14210	1182597..1183403	0	0	0	0	0	0	0	0	0	0	0	0	0	0	0	0	0	0	0	0	0	0	0	0	5	0	0	0	0	0
CTC_RS05685	1183756..1184898	0	0	0	0	0	0	0	0	0	0	0	0	0	0	0	0	0	0	0	0	0	0	0	0	0	0	0	0	0	0
CTC_RS05690	1185027..1185305	0	0	357	0	41	62	0	367	335	0	634	128	0	813	639	0	452	182	61	173	86	0	0	146	0	581	166	25	352	172
CTC_RS05695	1185379..1186149	0	46	69	40	0	45	0	20	104	54	104	46	0	76	54	18	98	230	11	0	0	0	0	0	0	43	120	9	223	199
CTC_RS05700	1186168..1187223	128	180	428	152	239	277	88	104	177	257	365	237	75	263	318	161	597	420	181	504	249	94	318	270	128	328	351	280	558	345
CTC_RS05705	1187387..1188667	84	74	114	143	36	188	102	172	167	166	201	279	90	131	196	117	39	178	129	76	243	19	102	318	118	166	127	80	345	150
ftsA	1188787..1190049	942	413	316	900	563	314	1403	442	444	437	229	510	1143	597	465	1418	280	792	1072	326	568	727	384	242	1130	619	385	329	408	592
ftsZ	1190065..1191180	2095	1467	834	2445	1130	680	2299	1100	1221	958	850	1925	2083	1095	1043	3119	1062	1668	1728	1474	1758	2146	1505	730	2381	1222	1433	736	1188	1383
spoIIGA	1191335..1192138	0	207	198	19	342	150	0	157	233	0	140	237	0	237	209	8	31	362	0	60	417	31	93	51	0	292	288	0	122	1311
sigE	1192285..1192992	0	452	376	32	842	341	26	356	604	8	341	472	20	393	593	24	143	492	6	205	608	0	264	230	0	268	589	0	277	1733
sigG	1193066..1193839	0	521	704	59	622	245	0	569	897	8	872	617	28	1324	1274	79	522	671	22	375	340	0	241	53	0	917	1588	0	634	1511
CTC_RS05735	1193973..1194236	0	90	50	0	0	0	0	60	0	0	61	0	0	222	159	0	96	48	0	0	91	0	0	0	0	127	176	0	0	145
nrdR	1194304..1194762	0	362	232	17	400	38	20	206	407	64	175	156	0	478	503	30	0	166	37	0	313	27	407	532	0	414	0	30	160	125
CTC_RS05745	1195145..1195843	0	68	0	22	0	0	13	0	0	59	23	68	0	63	30	0	0	36	18	0	68	0	0	58	0	48	33	69	0	27
CTC_RS05750	1195854..1197569	8	138	108	33	53	70	27	64	125	95	169	42	8	119	55	8	162	59	26	56	70	7	152	47	0	72	41	52	129	212
CTC_RS05755	1197706..1198578	0	0	30	0	26	20	21	0	31	20	18	55	8	50	120	8	29	44	10	0	0	0	0	93	18	77	80	8	0	44

pstC	1198597..1199517	0	0	72	17	0	19	0	0	29	13	17	0	0	0	0	0	0	0	0	0	26	0	0	0	0	12	101	0	0	21
pstA	1199522..1200409	0	0	30	17	0	19	32	0	0	33	0	81	0	16	24	15	57	0	14	0	0	0	0	46	0	25	26	0	0	0
pstB	1200420..1201169	0	0	9	10	0	0	0	0	71	8	21	0	0	0	0	0	0	0	6	64	0	0	50	0	0	15	0	0	0	0
CTC_RS05775	1201292..1202608	0	54	101	116	52	65	50	48	61	193	110	109	33	67	175	72	38	106	97	37	73	57	85	93	54	25	88	89	56	102
der	1202609..1203925	51	108	212	185	191	262	113	108	81	130	134	399	126	200	223	171	230	288	149	147	182	160	170	93	78	216	158	193	261	218
CTC_RS05785	1203933..1204922	14	288	201	285	278	331	104	175	243	310	114	555	102	192	64	158	178	281	240	391	242	88	113	206	88	282	211	271	347	155
spoIVA	1205056..1206534	0	1933	751	5	775	52	0	1474	605	0	5646	81	5	4320	1234	0	2994	377	0	1014	243	0	580	28	0	3597	1066	9	2614	797
CTC_RS05800	complement(1206919..1207515)	0	0	0	0	0	0	0	0	0	0	0	0	0	0	0	0	0	0	0	0	0	0	0	0	0	0	0	0	0	0
CTC_RS05805	complement(1207827..1208132)	0	0	0	0	0	0	0	0	0	0	0	0	0	0	0	0	0	0	0	0	0	0	0	0	0	0	0	0	0	0
cas6_1	1208850..1209593	0	96	54	31	0	46	38	254	108	0	0	0	0	118	28	0	0	0	0	0	0	0	150	55	32	150	0	0	0	0
cas8a1	1209608..1210951	10	106	59	45	51	90	69	211	229	0	0	0	38	174	117	0	0	0	0	0	0	28	83	61	24	200	95	0	0	0
cas7i	1210967..1211866	30	237	251	178	280	249	145	560	683	0	0	0	120	325	303	0	0	0	0	0	0	152	373	181	167	484	361	0	0	0
cas5	1211894..1212976	75	307	270	155	296	302	121	574	395	0	0	0	77	365	155	0	0	0	0	0	0	92	207	150	95	382	289	0	0	0
CTC_RS05830	1213027..1215306	12	83	58	20	20	45	4	83	47	0	0	0	16	58	46	0	0	0	0	0	0	11	16	0	17	74	31	0	0	0
cas4_1	1215335..1215829	0	48	27	0	46	35	0	127	162	0	0	0	0	0	42	0	0	0	0	0	0	0	0	0	16	23	187	0	0	0
cas1b_1	1215847..1216839	14	131	67	54	46	17	56	143	108	0	0	0	0	74	63	0	0	0	0	0	0	0	38	0	16	45	23	0	0	0
cas2_1	1216851..1217129	48	85	0	0	246	0	0	169	0	0	0	0	0	52	0	0	0	0	0	0	0	44	0	0	28	80	0	0	0	0
CTC_RS05850	1219629..1221578	0	0	0	0	0	0	0	0	0	0	0	0	0	0	0	0	13	19	0	0	0	0	19	0	0	0	0	11	0	20
CTC_RS05855	1221805..1222617	0	175	33	0	28	21	11	155	66	7	178	88	0	216	103	0	341	16	0	0	0	15	46	50	5	192	86	8	362	165
CTC_RS05860	1222806..1223213	0	465	4808	0	0	634	0	347	2226	14	276	1433	0	466	3444	0	618	7401	0	237	352	0	366	798	0	822	4432	0	361	1456
CTC_RS05865	1223395..1223634	0	1483	997	0	382	144	0	2164	779	0	1406	1094	0	4209	2622	0	3994	923	0	403	399	0	467	509	0	4890	2415	0	5011	1038
metA	complement(1223807..1224727)	0	0	0	0	0	0	0	0	0	19	0	26	0	0	23	7	27	0	5	0	0	0	0	44	0	24	0	0	0	0
CTC_RS05875	complement(1224746..1226023)	0	0	10	36	0	13	0	0	0	23	13	93	0	11	0	5	39	0	10	0	37	10	0	32	0	44	36	11	0	15
CTC_RS14215	complement(1226392..1226925)	860	89	523	157	215	710	926	354	100	210	692	447	311	82	393	472	709	427	255	362	582	93	559	991	238	293	434	695	506	323
CTC_RS05885	1227261..1228748	54	96	116	205	216	284	172	116	108	433	76	337	119	59	99	270	119	204	317	163	209	183	176	109	165	105	93	268	231	206
cooS	complement(1228884..1231022)	0	22	6	4	0	8	0	52	12	0	113	33	0	96	20	0	35	0	0	0	11	0	17	0	0	26	22	3	115	0
CTC_RS14855	1231665..1231820	0	0	85	0	0	0	0	0	0	0	103	0	0	0	0	44	0	162	0	0	0	0	0	0	0	0	149	0	0	0
CTC_RS05895	1231968..1233002	26	69	64	44	133	67	0	76	77	29	47	23	14	85	20	66	0	12	29	93	116	0	72	79	31	43	0	13	47	0
CTC_RS05900	1233152..1233442	46	0	0	0	0	0	0	0	92	0	0	0	0	0	0	0	0	0	15	0	0	0	0	0	0	0	0	0	0	0
CTC_RS05905	1233525..1234469	0	25	28	8	0	0	10	17	28	25	0	51	0	0	0	40	0	7	18	0	76	13	0	43	0	12	0	0	0	0
nifJ_1	1234853..1238380	4	235	75	17	71	10	3	234	38	12	194	54	2	469	48	37	186	140	8	178	41	7	63	12	4	456	46	12	285	49
CTC_RS14485	complement(1238439..1238606)	0	0	0	0	0	0	0	0	159	0	0	0	0	0	125	0	0	0	0	0	0	0	0	0	0	0	0	0	0	0
CTC_RS05915	complement(1238697..1240334)	17	0	0	0	0	11	0	10	0	22	20	44	4	9	0	0	15	23	5	30	15	0	0	99	0	7	0	8	0	23
CTC_RS05920	complement(1240543..1241454)	30	78	87	50	101	189	51	121	59	104	194	79	16	64	23	45	55	28	51	53	131	14	205	357	9	61	76	60	108	95
CTC_RS05925	complement(1241631..1243571)	21	12	41	24	0	36	10	8	41	67	17	25	19	15	32	21	13	33	22	0	62	13	38	63	16	12	36	18	0	99
CTC_RS14220	1243800..1245053	129	416	429	250	402	289	104	377	288	471	346	190	178	210	301	228	523	651	248	386	1011	287	298	292	133	432	314	219	470	948
CTC_RS05935	1245064..1245330	0	0	0	29	0	0	70	0	0	0	60	89	0	0	0	51	0	0	16	0	90	0	0	0	0	42	0	0	0	0
CTC_RS05940	1245391..1245921	25	0	0	14	0	32	0	30	50	22	0	0	0	55	0	77	0	0	32	0	0	0	70	0	0	21	0	13	46	72
CTC_RS05945	complement(1246008..1246901)	15	0	30	0	26	0	0	0	0	59	0	27	0	0	0	0	0	14	0	0	80	0	0	0	0	0	0	23	0	0
CTC_RS05950	1247092..1247406	0	0	0	0	0	55	0	0	0	19	51	0	0	0	67	0	0	40	67	0	0	0	0	0	0	35	0	65	0	183
CTC_RS14705	1247448..1247597	0	0	0	0	0	0	0	0	0	0	0	0	0	0	0	0	0	0	0	0	0	0	0	0	0	0	0	0	0	0
CTC_RS05955	1247616..1248062	0	0	30	17	0	39	0	0	0	13	36	0	0	33	0	0	0	0	0	0	0	0	167	0	0	25	0	0	0	0
CTC_RS05960	complement(1248109..1248873)	53	31	0	20	30	0	49	0	105	39	21	0	28	0	0	45	33	17	50	0	63	0	0	0	10	0	0	144	32	13
CTC_RS05965	1249155..1249838	20	35	39	0	0	25	14	46	39	52	0	70	0	21	0	30	0	0	31	0	0	36	55	119	0	33	0	30	0	112
CTC_RS05970	1250121..1250888	18	31	35	10	0	45	0	41	0	38	0	31	9	0	0	53	0	16	55	0	31	0	49	0	52	15	30	36	96	0
CTC_RS05975	1250908..1252284	10	69	29	17	33	63	30	23	19	47	47	69	10	21	15	69	18	37	12	70	35	32	54	30	0	41	34	25	0	0
CTC_RS05980	1252512..1253789	0	0	21	6	0	27	15	0	0	5	25	37	0	0	0	32	20	50	7	0	75	0	0	96	6	0	0	22	0	15
CTC_RS05985	1253834..1254787	0	25	42	32	0	18	10	0	0	43	51	25	26	77	22	21	53	13	27	0	0	0	0	43	17	23	24	14	26	20
CTC_RS05990	1254972..1255655	40	69	136	22	268	353	14	46	195	69	211	70	21	21	61	60	148	148	62	141	245	0	109	298	23	33	68	30	36	84
CTC_RS05995	1255677..1255904	0	104	117	0	0	0	41	0	0	104	70	0	95	0	92	119	0	56	112	0	420	0	0	179	139	147	203	60	0	168
CTC_RS06000	1255919..1256446	51	270	101	116	43	163	53	30	101	112	30	0	14	83	40	77	0	120	72	0	91	47	71	0	30	0	0	91	46	218
CTC_RS06005	complement(1256470..1257360)	30	133	45	34	26	77	42	53	30	172	18	54	8	0	0	46	0	71	33	0	27	14	84	228	0	13	78	31	55	65
CTC_RS06010	1258012..1259712	365	760	86	211	512	132	285	685	236	2444	321	168	295	267	111	448	89	104	350	256	183	365	658	144	371	565	177	1437	361	146
CTC_RS06015	1259759..1261075	92	90	40	75	35	39	28	48	20	381	0	36	71	56	32	83	57	0	168	0	36	9	142	62	42	51	35	438	0	29
CTC_RS06020	complement(1261207..1262487)	0	241	659	0	143	94	0	455	188	0	201	37	0	560	278	0	689	415	0	38	56	0	87	32	0	733	145	0	690	135
CTC_RS06025	1262903..1263259	3445	4153	4135	3783	6068	6470	2848	4101	4714	1357	405	334	1779	2768	3408	1068	141	745	357	0	436	2746	6273	7983	2033	3272	3052	732	550	886
CTC_RS06030	1263382..1263948	2955	5438	2556	6043	5539	3891	3702	5525	4051	818	595	589	3380	4776	4828	1045	400	514	637	512	591	4497	6253	5600	3811	5579	4436	806	649	1251
CTC_RS06035	1264288..1265496	0	20	11	32	0	57	15	13	22	34	0	0	12	24	17	138	21	73	11	0	40	41	0	34	13	18	0	11	20	32
CTC_RS14985	complement(1266153..1266335)	295	259	0	250	0	188	255	344	292	258	527	0	39	400	0	335	138	69	256	1057	0	68	0	445	173	183	253	38	134	314
CTC_RS14990	complement(1266555..1267175)	218	115	300	295	369	416	240	380	172	1412	440	500	139	306	68	197	244	428	205	156	693	120	180	131	77	144	224	703	375	741
CTC_RS06045	1267554..1268306	0	0	18	10	0	23	12	0	35	16	21	32	19	39	0	27	101	0	45	0	64	0	0	0	0	0	0	0	0	25
CTC_RS06050	complement(1268355..1269821)	415	1617	1881	733	1938	2802	264	1331	1857	302	3774	1904	389	1322	1423	434	843	777	170	1945	2022	778	2366	2442	357	831	1541	122	937	1169
CTC_RS14910	complement(1270208..1270561)	0	0	0	0	0	49	0	0	0	33	0	0	0	0	0	0	0	0	0	137	0	0	0	0	0	0	65	0	0	0
CTC_RS06060	complement(1270573..1271070)	0	48	0	0	0	69	0	32	54	0	0	0	43	0	0	0	0	25	9	0	0	0	0	0	0	22	0	0	0	0
CTC_RS06065	1271359..1272675	10	108	91	72	35	79	7	48	81	105	12	72	49	11	48	21	77	24	65	37	163	19	0	124	24	51	53	50	19	73
CTC_RS14995	complement(1272788..1273717)	0	255	729	0	99	93	0	220	172	0	190	26	0	205	282	0	570	551	0	0	51	0	80	0	0	216	274	0	343	206
CTC_RS06075	1273925..1277524	15	198	164	130	156	237	73	103	178	244	40	242	38	138	253	121	81	283	125	40	213	76	249	226	41	149	184	63	85	631
CTC_RS14225	1277552..1278436	46	214	150	155	130	127	26	107	181	227	18	108	8	66	0	135	57	143	55	0	54	84	0	92	54	126	131	39	28	152
isdE	1278532..1279422	0	80	75	9	0	58	0	35	0	66	0	27	0	33	0	76	0	21	67	0	0	28	0	0	0	38	26	39	55	172
CTC_RS06090	1279432..1280439	13	59	53	30	91	34	19	0	0	100	0	47	0	29	0	47	25	25	42	0	24	12	37	0	8	0	46	27	24	95
CTC_RS06095	1280432..1281229	0	0	0	0	0	0	0	20	0	30	0	0	0	18	0	26	32	16	5	61	60	0	47	51	0	28	0	0	0	96
CTC_RS06100	1281257..1281856	0	0	0	13	38	0	0	0	0	30	54	0	0	0	0	34	0	0	14	0	0	0	0	0	0	19	0	11	0	32
CTC_RS06105	1281846..1283588	8	14	15	0	13	40	0	18	15	24	0	0	0	8	0	4	14	7	5	0	27	7	21	0	0	19	0	12	14	0
CTC_RS06110	1283608..1285269	0	14	8	0	0	10	0	9	0	32	10	0	0	18	0	8	0	15	5	0	0	0	22	0	0	13	14	21	0	46
CTC_RS06115	complement(1285373..1285690)	0	0	0	0	72	0	29	0	0	56	0	225	0	0	0	0	0	119	13	0	150	39	117	128	0	0	73	0	0	0
CTC_RS06120	1285902..1287380	128	0	9	62	46	87	82	0	36	188	11	65	44	10	14	87	17	43	170	0	65	34	101	110	21	45	16	279	83	26
CTC_RS06125	1287420..1287980	120	42	71	27	0	61	33	0	0	168	0	128	64	0	75	49	45	90	114	0	43	44	0	218	14	60	83	221	0	0
CTC_RS06130	1288228..1289043	149	29	98	19	84	190	263	77	0	174	177	512	44	90	180	0	0	0	115	356	220	15	366	349	87	151	114	135	90	282
CTC_RS06135	1289376..1290731	0	0	39	0	0	38	21	0	20	22	24	0	0	22	15	35	0	56	16	0	18	0	0	0	12	16	0	10	0	57
CTC_RS06140	complement(1290735..1291007)	0	0	49	56	0	0	0	0	0	0	0	0	0	0	77	0	0	0	16	0	0	0	0	0	0	0	85	0	0	0

CTC_RS06145	1291140..1291889	0	0	0	0	0	0	0	0	0	0	43	0	0	0	0	0	0	17	0	0	0	0	0	0	0	0	0	9	0	0
feoB_3	complement(1292407..1294413)	121	295	351	468	343	309	172	251	253	1489	128	202	94	171	225	616	75	681	447	145	644	244	242	609	119	100	231	538	269	721
CTC_RS06155	complement(1294426..1294653)	296	936	525	1038	1709	605	1187	414	351	2357	141	628	412	128	460	1359	553	972	1380	1273	1679	109	1473	893	452	294	203	1266	861	1345
CTC_RS06160	complement(1294673..1294891)	309	542	243	383	942	315	639	431	488	1106	367	654	395	267	287	964	115	694	1068	1325	1092	170	170	0	398	179	106	848	224	219
CTC_RS06165	1295211..1295669	29	0	290	299	300	601	244	274	524	553	385	156	63	144	137	371	220	634	185	0	261	108	163	355	104	195	152	374	267	543
CTC_RS06170	1296082..1296501	0	56	0	0	0	41	0	75	0	0	77	114	17	35	0	16	120	30	40	230	0	0	0	0	0	0	0	33	0	0
dut	1296538..1296975	0	54	0	35	0	157	21	0	61	40	37	109	33	33	0	62	0	58	78	0	218	0	0	0	0	51	159	141	56	88
CTC_RS06180	1297047..1298759	103	111	113	143	134	262	133	230	125	147	122	209	122	154	159	183	236	251	246	226	349	65	218	190	83	144	81	165	172	224
CTC_RS06185	1298882..1299763	31	27	15	9	0	20	11	18	0	60	55	0	8	17	0	0	0	29	19	0	0	0	0	0	0	13	0	16	0	22
CTC_RS06190	1299884..1300756	387	82	30	376	79	0	609	90	0	61	92	191	852	34	72	417	87	44	351	55	27	689	86	0	858	90	27	220	84	66
CTC_RS06195	complement(1300817..1301287)	0	0	0	0	0	110	0	0	0	75	91	0	0	0	0	0	107	134	0	0	0	0	0	0	0	0	0	15	0	81
CTC_RS06200	1301584..1302462	215	162	151	230	156	333	361	116	61	323	91	407	213	33	143	349	57	302	409	165	354	85	85	232	167	102	26	196	307	174
CTC_RS06205	1302476..1302745	501	88	0	283	255	192	276	291	99	394	179	265	254	325	155	353	280	141	693	358	266	92	415	151	88	373	86	280	91	213
gmk	1302745..1303371	862	492	456	1205	548	357	841	452	213	815	103	990	656	374	201	978	241	707	1248	386	534	416	833	779	487	481	296	647	509	764
rpoZ	1303352..1303570	370	433	182	348	105	79	256	144	122	485	294	109	379	201	0	311	230	231	369	442	765	680	341	372	145	204	0	439	448	525
coaBC	1303573..1304766	498	417	646	981	538	967	473	422	291	913	363	900	290	294	211	602	401	594	538	405	882	582	688	341	226	290	291	746	349	602
priA	1304848..1307055	12	54	18	45	31	39	17	29	36	104	44	119	29	33	0	65	34	120	65	22	43	17	68	55	9	30	0	37	33	43
def	1307078..1307530	0	52	205	152	253	76	144	243	59	130	71	53	32	194	185	135	111	224	99	0	158	82	82	90	88	74	256	61	54	254
fmt	1307535..1308464	15	77	129	115	173	74	30	102	0	146	86	257	140	55	68	139	163	184	165	156	232	80	0	44	68	168	25	74	53	82
CTC_RS06240	1308480..1309160	20	0	137	78	135	228	0	23	39	217	24	35	11	86	31	50	37	177	19	71	246	0	55	149	58	33	68	61	90	338
rsmB	1309238..1310566	10	18	40	17	34	130	14	24	40	67	12	36	16	33	0	15	0	57	35	73	72	9	0	92	24	17	17	26	0	43
rlmN	1310576..1311616	0	46	64	59	44	116	45	30	26	96	15	115	21	56	0	72	73	73	78	93	161	24	72	39	15	43	45	86	24	92
CTC_RS06255	1311621..1312367	18	64	18	20	31	92	0	42	0	47	22	32	48	59	0	27	0	68	23	0	64	17	100	0	21	60	31	37	99	128
pknB	1312342..1314153	22	105	161	118	240	257	57	113	206	183	177	395	64	129	69	64	153	133	127	133	363	34	227	202	53	68	64	129	176	116
rsgA	1314172..1315044	0	27	30	61	26	109	43	90	61	20	37	41	25	34	0	16	29	0	29	0	0	0	86	47	0	51	27	31	84	44
rpe	1315038..1315691	0	73	20	58	140	105	71	72	41	108	25	219	22	90	0	0	0	194	72	0	110	19	0	62	0	137	71	74	38	176
CTC_RS06275	1315688..1316329	21	148	124	59	107	215	0	98	42	120	25	0	22	0	33	21	118	20	40	0	0	0	0	127	12	17	36	75	0	30
CTC_RS14710	1316392..1316553	0	0	328	47	141	0	0	291	495	0	99	0	0	542	518	0	156	78	26	0	0	0	0	0	0	138	716	0	152	118
rpmB	complement(1316675..1316866)	985	371	69	238	239	90	194	0	139	661	0	249	188	229	109	177	0	198	177	0	249	323	389	212	124	175	0	716	511	100
CTC_RS06285	1317056..1317406	154	68	114	43	196	49	133	224	304	84	137	68	288	292	478	252	72	325	200	138	68	106	213	348	90	255	462	118	0	218
CTC_RS06290	1317421..1319061	560	737	373	530	503	525	421	460	521	551	333	756	576	446	422	550	246	455	488	236	802	484	671	459	498	409	494	348	254	438
recG	1319119..1321170	7	0	6	19	11	25	18	0	26	32	20	0	11	21	20	13	0	80	35	24	64	30	18	40	12	5	23	37	12	19
rsmD	1321261..1321818	121	128	95	137	41	309	134	71	0	318	86	128	78	52	188	195	136	136	84	87	214	111	134	73	43	60	83	172	44	240
coaD	1321820..1322302	308	147	165	395	237	71	270	33	0	379	33	198	463	182	43	338	104	262	423	501	495	231	386	506	197	139	48	584	152	437
CTC_RS06310	1322314..1322841	102	314	227	145	65	131	88	60	152	157	30	271	137	55	159	129	239	96	129	366	136	70	283	77	150	64	44	260	93	290
ylbJ	complement(1322842..1323990)	12	0	12	53	20	0	0	0	0	0	28	0	0	13	18	24	44	11	4	0	0	0	0	0	0	10	20	12	21	0
CTC_RS06320	complement(1324141..1324746)	0	0	0	0	0	0	0	0	0	58	0	0	0	0	0	0	0	0	7	0	79	0	0	0	0	0	0	0	0	0
CTC_RS06325	complement(1324854..1326065)	0	20	11	6	19	0	8	0	0	39	13	20	0	12	17	8	0	21	14	40	59	10	31	0	7	0	0	11	0	16
pta	1326361..1327359	122	558	293	153	849	311	65	646	508	349	322	526	58	762	273	232	455	1267	458	387	670	75	523	367	56	515	534	396	479	480
CTC_RS06335	1327414..1328616	45	335	243	216	267	387	31	432	200	550	214	228	36	329	209	159	357	705	307	322	338	93	558	372	66	400	125	291	143	366
CTC_RS06340	1328693..1329196	1180	1459	475	1560	1455	787	666	1343	768	4412	638	1042	959	1249	499	2101	851	1344	3159	672	759	886	889	969	708	1397	759	5245	730	2054
rpmF	1329210..1329392	923	1685	872	876	1503	1036	306	1118	511	1985	703	652	828	960	688	1340	965	553	2021	0	1177	1220	1020	445	910	672	507	3494	805	733
plsX	1329690..1330694	40	142	119	91	91	103	121	219	186	138	64	166	93	189	188	108	301	227	190	48	143	12	74	243	103	178	92	120	220	191
acpP	1330795..1331028	3725	2433	4035	1989	2939	6777	2173	4944	10958	2574	5495	5355	2406	3378	11384	2183	4366	5654	3289	3721	5929	2810	4147	7307	3372	4060	13128	3349	2570	5284
rnc	1331154..1331861	19	0	19	0	0	24	0	0	38	17	91	0	0	0	0	19	0	18	12	0	0	18	0	0	0	0	0	10	35	27
CTC_RS06365	1331854..1332909	26	45	0	36	43	82	44	30	0	22	46	113	27	28	60	39	24	48	32	46	136	12	0	77	0	32	88	52	23	36
CTC_RS06370	1333029..1333289	984	1454	764	1593	439	330	536	1206	1740	181	1293	823	1535	1066	2210	2662	677	873	1955	1668	458	951	1001	156	1033	1242	2087	158	752	588
CTC_RS14715	1333452..1333550	0	0	0	0	0	0	0	0	0	0	0	0	0	0	0	0	0	0	0	0	0	0	0	0	0	0	0	0	0	0
smc	1333563..1337123	15	40	49	41	58	92	31	35	38	106	41	54	20	53	53	44	57	121	70	14	67	21	42	34	16	22	65	58	28	97
ftsY	1337149..1338060	74	78	58	142	101	94	41	121	88	117	141	157	47	80	23	127	138	167	182	0	52	68	82	0	43	86	153	128	108	105
CTC_RS06385	1338306..1338659	38	67	113	65	65	97	53	89	0	367	45	67	20	124	59	38	0	340	192	137	68	0	0	0	0	189	65	194	0	325
ffh	1338662..1340017	10	87	186	84	118	165	7	116	158	684	59	211	11	86	201	35	167	289	241	250	318	64	83	30	20	78	171	243	36	452
rpsP	1340057..1340314	5971	1287	747	1923	711	468	3435	1159	1139	2484	934	740	1958	1532	976	3379	978	981	8421	937	185	1828	1736	158	2567	1690	1078	6715	666	743
CTC_RS06400	1340331..1340558	119	0	58	0	0	0	41	0	0	207	141	419	0	0	184	60	0	56	149	0	105	109	0	0	0	0	0	151	0	0
rimM	1340616..1341110	0	48	27	31	0	104	19	0	0	143	0	0	0	30	42	0	51	0	116	0	0	50	151	0	0	90	0	97	0	77
trmD	1341095..1341814	38	66	18	21	64	48	0	0	0	168	156	133	20	142	0	0	0	70	127	0	33	17	0	0	0	78	0	134	34	53
rplS	1341939..1342286	1204	2181	1605	3245	1317	941	844	2329	2072	1561	1062	960	1690	2860	2290	2867	1414	2674	3360	834	481	3120	1501	936	1367	2907	2798	4791	1693	2038
lepB	1342341..1342865	0	45	76	15	87	33	53	30	102	135	0	45	14	56	80	143	48	169	89	0	91	24	213	78	15	43	44	92	0	146
ylqF	1342886..1343731	0	84	31	81	27	20	22	37	0	98	38	56	13	52	50	72	60	120	121	0	141	59	0	96	28	53	110	65	87	23
CTC_RS06430	1343782..1344561	0	0	0	29	29	44	12	20	34	45	41	61	9	0	0	9	32	16	27	0	0	0	96	52	20	43	0	18	31	25
CTC_RS06435	complement(1344580..1344948)	0	0	0	0	124	0	0	0	145	32	44	0	0	0	114	18	0	34	0	0	65	0	0	221	0	30	0	0	0	0
CTC_RS06440	1345199..1346704	0	16	9	0	0	0	0	10	0	4	21	32	0	10	14	0	17	0	0	0	0	0	0	0	0	15	15	0	0	13
dprA	1346718..1347806	0	0	0	0	0	0	0	0	25	5	0	0	0	0	0	0	0	0	8	0	0	0	0	0	0	10	0	0	0	0
topA	1347862..1349946	52	125	19	55	88	58	54	30	0	184	116	23	104	63	40	98	36	79	106	23	34	48	90	20	30	43	44	137	24	92
codY	1350208..1350984	2235	1160	394	2907	1003	510	2515	669	653	1448	1427	307	2977	1413	567	2415	1136	554	2137	1183	708	2954	961	786	2858	1381	567	2645	916	493
rpsB	1351198..1351899	1992	3007	1231	3973	1992	1547	2100	2108	1408	4695	2290	2210	2848	2648	1972	3905	1797	2236	6381	3101	2794	2863	2685	1160	2186	2548	1767	8138	2273	2103
tsf	1351985..1352899	1499	2100	1686	2018	1854	2072	1234	1600	1547	3779	2020	2609	1175	1408	1857	1660	1792	2117	2277	2115	2536	1227	2039	1935	1170	1301	1761	3073	1878	1760
pyrH	1353050..1353763	303	565	372	588	1059	676	340	860	411	422	743	1003	460	697	734	620	777	851	724	678	972	452	941	627	378	720	455	751	825	1315
frr	1353778..1354329	734	1203	1180	1127	1308	1358	439	1027	968	1150	684	1730	628	849	684	747	846	1422	578	438	1170	1068	1623	1143	603	911	735	822	1045	2066
CTC_RS06480	1354391..1355158	0	62	17	89	0	157	36	41	0	100	0	93	94	76	82	124	0	148	138	0	125	48	0	159	62	73	60	72	32	50
CTC_RS06485	1355395..1356192	51	59	50	105	86	281	35	39	67	126	81	90	27	18	79	17	32	127	107	182	120	16	47	153	20	56	29	52	0	120
CTC_RS06490	1356347..1357321	14	0	0	8	47	53	0	0	0	24	0	0	0	15	43	14	0	39	17	50	0	0	0	0	0	11	0	7	25	20
CTC_RS06495	1357347..1358501	0	0	23	20	20	60	16	41	46	133	42	41	44	63	73	65	22	110	66	0	21	0	97	106	14	19	40	54	64	116
rseP	1358545..1359558	40	47	79	68	158	85	18	93	26	99	63	71	28	130	41	81	25	125	63	95	165	0	147	120	31	33	91	27	48	132
ispG	1359571..1360620	26	181	127	87	131	33	71	105	76	177	61	68	27	70	20	58	48	326	105	46	68	95	36	0	15	149	66	124	94	146
CTC_RS06510	1360643..1364926	50	94	53	77	177	105	33	156	94	255	101	111	27	103	54	76	141	167	99	124	114	46	122	67	29	120	49	95	149	166
rimP	1365136..1365597	0	103	0	83	50	149	61	68	116	345	139	207	31	222	182	44	109	301	64	0	362	27	485	176	17	73	50	60	0	83
nusA	1365613..1366698	87	284	110	123	169	175	60	290	246	386	281	374	93	216	155	194	209	303	215	89	242	103	395	262	51	329	213	228	181	441
CTC_RS06525	1366716..1366988	0	0	49	112	0	126	103	404	98	335	118	525	106	107	0	175	370	232	140	177	0	136	410	149	116	41	255	126	180	140
CTC_RS06530	1366978..1367283	0	388	174	150	300	169	30	309	0	444	263	156	94	191	69	156	247	124	208	0	235	162	0	133	26	183	0	157	80	188

infB	1367306..1369354	92	486	286	302	554	278	246	430	313	517	451	839	282	500	287	399	394	473	396	472	490	230	528	199	273	464	362	302	311	458
rbfA	1369370..1369735	37	1167	218	209	564	377	204	344	584	597	527	1761	237	360	401	223	276	346	412	529	588	237	714	111	173	611	380	601	268	576
CTC_RS06545	1369722..1370687	84	123	179	205	403	205	232	171	194	514	100	272	179	152	282	127	104	197	251	150	371	141	425	337	82	185	192	285	102	179
truB	1370684..1371553	31	136	76	70	132	99	21	72	184	258	129	219	41	135	96	23	87	116	108	167	165	43	43	47	36	154	27	142	0	176
CTC_RS06555	1371565..1372515	57	75	70	80	121	217	10	83	56	438	118	201	30	31	88	50	106	200	94	102	126	39	78	107	0	47	24	173	52	60
rpsO	1372619..1372882	2201	5301	4685	4278	4168	4636	2297	4830	4755	6085	9285	7956	2297	5850	6912	4360	4491	4172	6144	4214	4712	3243	5231	7094	2343	7155	8167	6406	4463	4974
CTC_RS06565	1372995..1375112	810	1019	848	1850	1212	1143	824	1107	1236	2047	615	1375	1053	743	1134	1740	476	1366	1588	662	1519	1617	1313	1211	1069	1114	1029	1678	556	1525
CTC_RS06570	1375177..1376475	31	18	41	23	35	80	36	24	82	50	37	92	6	11	48	26	0	83	36	0	18	19	29	31	12	9	45	37	0	44
CTC_RS06575	1376552..1376827	0	0	145	0	0	0	0	57	0	0	175	86	0	106	76	0	91	46	15	0	87	45	0	0	0	0	0	25	89	139
dapG	1376844..1378049	11	177	132	44	114	86	46	131	22	73	293	158	24	255	139	45	251	178	35	80	198	31	62	135	20	334	135	29	265	95
CTC_RS06585	1378111..1378788	0	0	0	11	34	0	0	0	0	0	95	70	11	43	31	0	74	0	0	143	0	0	0	0	0	49	34	0	0	28
CTC_RS06590	1379043..1381274	18	53	12	62	72	23	21	42	0	52	43	32	23	26	9	76	34	57	24	22	21	22	17	18	21	25	21	43	55	52
rimO	1381295..1382629	30	89	90	114	120	39	35	41	40	84	96	18	103	143	63	163	57	218	121	0	0	93	112	0	83	100	0	72	92	72
pgsA_2	1382613..1383176	24	210	71	95	41	275	83	140	189	147	285	0	64	78	0	133	134	269	113	0	403	0	132	180	0	40	103	24	131	306
recA	1383544..1384596	154	248	366	413	305	196	301	359	558	595	137	589	473	278	697	285	120	715	561	184	454	318	461	348	369	382	484	418	373	583
rny	1384768..1386308	1956	3002	2697	2865	3369	2875	1740	2487	1777	5331	2326	5189	1740	2014	1797	3912	2423	4297	3661	3673	5224	2569	2579	2140	1770	2227	2076	5311	2668	5423
CTC_RS06615	1386462..1386722	2641	909	611	2997	790	726	3253	1025	1330	634	369	366	3332	2412	1527	3784	193	631	2655	1112	367	3661	572	0	4178	1328	1066	764	752	808
CTC_RS06620	1386822..1387679	0	28	15	0	0	0	0	0	31	34	0	0	8	0	0	16	0	15	20	0	0	0	0	0	9	0	0	8	0	0
CTC_RS06625	1387699..1388958	0	0	11	0	0	0	0	0	0	37	0	19	0	23	0	5	0	20	13	0	0	0	30	0	6	0	0	16	19	0
CTC_RS06630	1388975..1389331	38	0	0	43	64	0	0	0	0	33	0	0	20	0	0	0	0	0	83	0	0	35	0	0	0	0	0	19	0	0
CTC_RS06635	1389517..1390710	34	159	262	64	288	419	23	119	313	188	283	100	36	307	404	86	232	636	212	122	531	42	250	307	7	169	272	92	123	787
CTC_RS06640	1390790..1391047	157	368	567	118	355	267	217	671	932	206	187	463	224	908	2927	475	1271	1472	494	1500	1113	289	1302	631	307	563	1977	107	190	223
CTC_RS06645	complement(1391083..1391301)	0	0	0	0	105	79	0	72	0	0	367	0	0	201	0	0	230	0	0	0	0	0	0	0	0	153	0	0	0	88
CTC_RS06650	1391585..1392850	1612	319	678	319	579	1062	715	99	274	865	1060	1075	311	93	298	291	1196	495	734	688	756	147	442	2187	294	177	385	923	620	379
CTC_RS06655	1393171..1394157	0	0	27	12	23	35	0	0	27	6	0	24	0	15	21	7	77	38	0	0	24	0	0	0	0	0	23	21	25	19
CTC_RS06660	complement(1394175..1394546)	0	128	71	21	62	185	125	254	0	79	0	0	0	39	0	18	0	170	34	0	64	67	100	0	85	210	125	74	0	52
lexA	1394722..1395324	134	275	221	89	228	515	62	235	177	362	267	396	132	97	70	147	167	147	162	0	159	123	309	473	79	37	308	171	122	191
CTC_RS06670	complement(1395361..1396644)	37	129	52	12	54	54	7	25	0	120	25	37	34	11	49	127	59	148	113	0	75	19	0	32	31	9	36	145	57	45
hfq	complement(1396751..1396990)	0	494	55	191	0	215	0	0	0	49	670	597	481	915	830	454	315	528	35	202	598	155	156	0	562	605	386	86	614	639
miaA	complement(1397038..1397982)	57	75	70	73	146	274	10	100	113	100	85	202	69	46	133	79	107	241	83	154	203	79	158	86	42	118	49	95	104	81
mutL	complement(1398000..1399862)	0	13	46	41	25	79	20	42	115	76	95	51	8	31	39	37	108	82	32	78	90	7	40	87	15	72	68	41	92	93
mutS	complement(1399874..1402519)	26	72	85	49	104	143	25	36	101	102	85	144	19	55	55	35	57	191	35	146	199	33	56	138	21	46	44	62	111	159
miaB	complement(1402637..1403995)	70	140	132	174	118	260	76	232	79	282	47	88	69	118	123	175	149	242	100	249	370	73	137	150	99	164	119	167	0	254
CTC_RS06700	complement(1404107..1404604)	0	48	134	0	46	0	0	32	349	0	97	0	0	118	84	41	51	89	0	0	192	50	225	0	0	112	140	0	0	173
CTC_RS06705	complement(1404625..1405395)	105	492	535	59	268	89	230	715	1022	31	354	294	75	475	979	203	229	452	88	314	496	16	97	211	21	159	571	18	64	398
CTC_RS06710	1405654..1405839	0	128	71	0	0	185	0	85	0	0	0	0	0	630	113	0	0	0	0	0	0	0	0	0	0	421	125	0	132	412
CTC_RS06715	1405851..1406099	0	476	587	0	276	208	0	759	1287	0	1033	192	0	705	253	0	861	153	0	583	0	0	150	327	0	718	745	0	690	693
CTC_RS14495	complement(1406158..1406325)	0	0	0	0	0	0	0	0	0	0	0	0	0	0	0	0	0	0	0	0	0	0	0	0	0	0	0	0	0	0
CTC_RS06720	complement(1406454..1406861)	33	233	7236	37	169	1394	91	231	3601	333	158	1843	177	574	2493	117	495	10023	21	0	1583	30	91	1397	97	164	3552	84	241	1738
CTC_RS06725	complement(1407047..1409047)	182	95	80	126	92	319	219	31	13	332	145	131	144	11	31	121	88	120	121	48	179	143	112	203	135	28	46	234	86	96
CTC_RS06730	1409405..1410604	45	0	89	32	38	101	31	66	45	59	80	80	30	12	70	74	0	106	78	81	140	10	124	102	13	75	58	132	82	80
CTC_RS06735	complement(1410626..1411504)	0	27	76	26	26	118	11	0	61	94	37	81	0	83	48	15	57	115	24	0	27	0	85	46	0	76	132	102	84	174
CTC_RS06740	complement(1411596..1412651)	0	45	13	0	11	33	0	15	0	0	76	0	0	69	20	6	119	36	16	0	0	0	0	0	0	85	22	0	23	36
speD	complement(1412752..1413132)	106	249	279	180	120	181	73	207	245	171	84	188	95	115	110	179	66	100	597	0	251	98	196	107	21	205	183	180	193	201
CTC_RS06750	complement(1413361..1415268)	460	398	251	728	505	289	428	363	462	320	278	125	513	284	209	660	198	478	374	355	351	624	352	256	484	296	194	256	283	482
cmk	complement(1415265..1415945)	159	279	137	202	303	494	82	208	314	208	708	210	138	290	339	290	667	920	194	213	492	128	164	598	105	164	340	192	432	760
aroH	complement(1416013..1416369)	0	0	37	0	0	0	0	0	0	17	45	0	0	0	0	0	0	35	0	0	0	0	105	114	0	63	0	39	0	215
CTC_RS06765	complement(1416369..1417595)	11	77	0	12	19	42	8	0	0	87	0	19	12	12	17	17	21	41	42	0	39	0	30	17	6	0	38	67	40	47
CTC_RS06770	complement(1417647..1418525)	154	135	408	95	522	912	85	340	456	480	731	597	57	383	143	194	316	447	189	660	572	113	297	1135	117	146	317	317	112	611
CTC_RS14500	complement(1418887..1419057)	0	0	0	0	0	0	0	0	78	0	0	0	0	0	0	0	0	0	0	0	0	0	0	0	0	0	0	0	0	0
CTC_RS06775	complement(1419076..1419759)	40	104	58	89	168	126	27	23	78	86	70	105	16	21	0	40	74	56	56	71	0	0	0	119	12	49	34	70	0	56
CTC_RS06780	complement(1419806..1420573)	106	124	156	209	507	314	36	61	104	285	188	373	28	114	164	124	0	396	119	378	716	97	632	424	31	146	91	134	160	250
CTC_RS06785	complement(1420652..1422052)	178	423	285	283	712	578	133	472	400	769	562	460	103	261	329	258	315	660	267	501	973	186	706	1162	57	347	381	466	578	1026
CTC_RS06790	complement(1422199..1422768)	107	458	257	228	925	696	295	580	375	596	705	796	171	745	442	191	398	578	276	764	462	392	851	429	181	412	366	314	624	773
CTC_RS14505	complement(1422791..1422889)	0	0	0	0	0	0	0	0	0	0	0	0	0	0	0	0	0	0	0	0	0	0	0	0	0	0	0	0	0	0
CTC_RS06795	complement(1423319..1424887)	5064	1867	1187	4312	2988	2340	2747	968	766	2140	2243	1506	2436	728	548	4606	1093	1485	1646	1973	1860	4203	2938	1738	2497	755	369	1720	1416	2089
CTC_RS06800	complement(1425126..1425656)	0	89	0	0	43	0	0	0	0	44	0	0	14	110	40	0	0	0	8	91	90	23	0	0	0	21	44	0	0	0
hydF	complement(1425681..1426871)	0	100	33	0	19	43	8	13	0	55	27	0	0	37	0	17	42	32	16	41	181	0	63	0	0	0	19	12	21	0
hydG	complement(1426885..1428309)	38	117	0	21	64	24	46	144	37	46	79	33	25	103	44	33	18	80	52	170	235	52	52	57	17	63	16	34	0	40
hydE	complement(1428311..1429360)	13	23	51	0	0	49	0	75	0	39	31	23	0	0	20	26	96	84	40	369	137	12	71	78	0	43	22	20	23	18
CTC_RS06820	complement(1429377..1429622)	0	96	54	0	0	0	38	0	0	48	0	0	29	0	0	0	0	0	52	393	194	101	152	0	0	0	0	0	0	0
CTC_RS14230	complement(1430219..1431136)	29	26	14	0	75	56	0	0	0	77	35	104	24	48	0	104	82	172	0	0	0	7	41	0	26	0	76	0	0	0
CTC_RS06830	complement(1431270..1431686)	0	0	32	37	165	0	11	38	128	57	39	0	87	70	101	0	0	61	51	0	57	60	0	98	57	0	111	16	59	92
CTC_RS06835	1431865..1433076	0	39	44	0	0	57	0	52	22	5	66	39	0	36	17	17	42	10	0	0	0	0	31	34	0	92	19	0	142	32
tkt	complement(1433121..1435109)	581	787	361	575	784	620	833	1227	658	906	501	828	566	346	390	657	850	923	765	1386	1094	387	863	839	602	683	513	1016	851	569
CTC_RS06845	complement(1435389..1436258)	78	136	122	211	158	297	102	72	184	414	92	110	17	50	0	102	116	131	132	56	316	128	86	94	36	103	27	213	197	176
CTC_RS06850	complement(1436400..1437806)	67	169	76	195	269	196	73	246	133	302	57	144	131	281	119	336	197	396	169	69	391	185	186	203	147	250	239	232	497	375
CTC_RS06855	complement(1437814..1438932)	97	233	107	177	266	223	133	113	72	253	302	256	135	334	56	256	135	305	141	216	385	122	267	255	92	150	124	147	329	257
CTC_RS06860	complement(1438945..1439724)	243	471	170	205	441	354	227	373	68	310	515	306	102	338	134	314	97	390	223	372	644	143	526	522	51	186	119	185	378	344
CTC_RS06865	complement(1439858..1441822)	55	12	20	50	23	18	0	8	0	50	16	0	33	15	0	28	39	39	35	25	49	13	19	0	12	6	0	59	25	68
CTC_RS06870	complement(1441842..1443302)	28	97	46	52	78	94	32	48	0	53	11	16	35	10	0	33	0	78	41	66	98	34	51	0	11	8	0	52	17	39
CTC_RS06875	complement(1443314..1444786)	18	48	27	47	62	117	6	0	36	68	44	49	20	40	14	46	17	103	49	99	162	17	101	111	32	8	0	37	17	39
CTC_RS06880	complement(1444783..1445148)	0	65	36	0	0	47	25	43	73	0	0	65	20	40	57	74	69	0	122	0	196	0	102	0	22	61	0	19	201	52
CTC_RS14235	complement(1445150..1445575)	32	0	62	18	54	162	44	0	0	21	38	0	34	0	0	16	0	89	80	0	168	29	263	0	0	0	0	65	115	135
CTC_RS14240	complement(1445576..1445854)	48	85	0	27	164	0	33	113	0	21	58	86	26	0	0	98	90	182	46	173	86	0	134	0	28	120	0	49	0	69
CTC_RS06890	complement(1445851..1446090)	0	0	0	0	191	72	0	66	0	0	0	0	0	0	0	85	0	211	142	0	0	0	311	339	0	0	0	43	102	0
mnhG	complement(1446077..1446400)	42	0	41	24	71	106	173	97	0	36	50	0	22	0	0	21	0	195	66	0	74	0	230	0	73	34	0	11	0	59
CTC_RS06900	complement(1446397..1446669)	0	0	49	0	168	0	0	0	98	22	0	87	26	54	0	50	0	46	93	0	88	0	0	0	0	41	0	0	0	0
CTC_RS06905	complement(1446659..1447147)	0	0	0	16	0	35	19	32	0	12	66	0	15	0	43	28	0	129	17	0	98	0	76	83	32	23	0	28	50	78
cysK	complement(1447306..1448223)	147	439	406	133	175	394	71	240	786	154	368	988	86	112	423	208	962	869	144	738	573	41	163	399	104	85	455	90	160	271

CTC_RS06915	complement(1448369..1448806)	0	54	152	0	105	79	0	36	61	54	73	327	16	33	144	78	288	318	19	0	218	0	85	93	18	26	53	31	0	175
ytaF	1448955..1449554	0	0	44	0	0	29	0	0	0	0	0	0	0	73	0	0	42	42	0	0	0	0	0	0	0	56	0	11	41	64
CTC_RS14245	complement(1449765..1450637)	0	2934	3801	0	827	612	0	4436	2922	0	2173	684	8	5794	3988	0	6098	4068	10	332	356	0	1112	373	0	6159	3638	8	5834	1361
CTC_RS06930	1450922..1451620	0	0	133	11	66	173	13	68	153	169	92	102	0	84	90	29	72	72	0	138	240	18	53	175	0	16	33	0	35	219
CTC_RS06935	1451634..1451930	0	240	134	128	309	174	0	159	90	60	271	482	146	148	424	11	0	256	72	814	886	167	0	137	0	226	390	23	165	323
mgtE	complement(1451971..1453314)	10	106	148	74	119	180	76	105	99	316	191	266	38	152	125	66	188	188	70	108	214	37	222	363	47	116	104	169	55	71
CTC_RS06945	complement(1453535..1454074)	125	747	616	353	552	223	17	947	594	153	699	619	241	867	388	265	1402	844	307	627	576	299	622	226	147	1283	644	51	727	319
CTC_RS06950	complement(1454164..1454988)	0	72	81	18	28	63	23	19	0	57	78	29	26	71	51	25	31	46	15	0	29	45	45	49	10	257	84	0	30	46
CTC_RS06955	complement(1454996..1455637)	0	0	0	0	0	0	0	0	0	28	0	0	0	0	0	0	0	0	7	0	37	0	116	0	0	17	0	11	0	30
CTC_RS06960	complement(1455638..1456594)	0	25	28	0	0	0	10	16	28	19	0	50	0	0	0	0	26	13	18	25	0	0	0	0	0	0	0	7	0	0
pssA_2	complement(1456837..1457355)	52	91	0	110	177	66	0	121	154	262	62	0	14	56	0	26	97	122	49	0	0	24	144	78	46	22	0	79	47	259
CTC_RS06970	1457684..1458595	0	52	0	33	0	0	0	52	0	39	18	26	0	16	0	0	55	0	5	0	26	0	0	0	0	12	0	15	0	21
CTC_RS06975	1458595..1459791	11	20	67	26	77	29	16	39	22	69	40	20	12	12	0	17	21	32	4	0	0	0	0	68	7	19	0	29	0	80
CTC_RS06980	complement(1459918..1461210)	0	110	494	0	35	107	0	268	186	14	62	74	0	170	114	5	205	392	0	0	19	0	29	63	0	311	126	0	323	119
CTC_RS06985	complement(1461283..1461945)	0	0	20	0	69	0	0	0	0	0	73	0	0	0	0	0	76	0	0	0	36	0	0	0	0	0	0	0	0	29
CTC_RS06990	complement(1462098..1462298)	0	2242	1191	0	228	515	0	4386	3588	0	1279	1900	0	16532	11375	0	16505	2142	0	1203	119	0	743	203	0	18685	4383	0	7815	858
CTC_RS06995	1462765..1464843	0	6	13	11	22	8	0	8	39	43	0	11	10	0	0	16	0	49	16	0	12	3	18	20	4	0	11	10	12	0
CTC_RS07000	1465006..1466385	59	206	993	420	656	1674	81	354	658	235	664	726	73	180	342	328	420	1257	142	351	1508	153	595	1121	80	162	454	45	569	1646
CTC_RS07005	complement(1466464..1468410)	0	18	27	35	0	18	10	0	14	15	17	61	61	11	54	0	0	13	9	99	0	6	19	10	53	23	48	60	38	79
CTC_RS07010	1468596..1468949	0	0	0	0	0	0	0	44	0	0	0	0	0	0	0	0	0	0	0	0	0	0	0	0	0	0	0	0	139	0
adhE	complement(1469020..1471650)	3465	1330	1395	8861	714	753	6480	1059	1503	128	1191	1365	8373	220	789	6587	968	188	2589	4845	2159	8027	880	1037	8052	234	996	120	2995	1435
CTC_RS07020	complement(1472103..1474580)	11	67	129	18	28	38	0	70	54	12	208	48	3	254	195	14	326	220	5	20	48	20	0	66	0	221	187	0	158	166
CTC_RS07025	complement(1474665..1476422)	0	0	15	0	26	20	5	18	30	13	9	0	4	8	0	0	0	22	2	0	0	7	21	69	5	0	66	4	42	22
CTC_RS07030	1476572..1477453	0	269	106	0	78	0	0	518	575	0	228	27	0	548	95	0	601	172	0	110	0	0	85	0	0	368	53	0	390	130
CTC_RS07035	complement(1477502..1478695)	0	0	11	6	0	43	0	0	0	25	67	80	0	12	0	11	63	0	18	81	40	0	0	34	0	19	0	29	21	32
CTC_RS07040	complement(1478692..1479720)	0	0	39	0	22	67	0	0	0	52	31	23	0	0	0	0	0	12	4	0	23	0	0	0	0	0	23	47	0	37
CTC_RS07045	complement(1479733..1480881)	0	62	23	7	20	30	0	14	0	26	42	62	0	13	0	33	44	72	26	63	94	0	32	71	0	19	20	75	117	83
CTC_RS07050	complement(1481268..1481711)	0	107	60	34	52	78	0	35	60	93	72	108	65	66	47	92	114	143	96	0	0	28	0	0	18	0	0	77	55	130
CTC_RS07055	complement(1481814..1482647)	0	0	32	0	0	0	0	19	0	42	0	0	0	0	0	0	0	0	10	0	0	0	45	0	0	0	0	8	0	23
CTC_RS07060	complement(1482750..1483556)	0	59	66	9	28	64	0	117	33	190	0	30	9	73	26	0	31	16	26	0	0	0	0	0	20	42	0	319	152	48
CTC_RS07065	complement(1483582..1484382)	0	30	17	19	29	22	0	39	0	177	20	89	0	0	0	17	31	0	21	0	0	15	0	0	0	0	0	146	31	24
CTC_RS07070	complement(1484420..1485217)	0	30	83	10	57	65	0	39	33	259	20	60	0	73	79	0	32	32	21	0	30	0	47	0	10	0	0	258	31	24
CTC_RS07075	complement(1485207..1486085)	0	54	15	17	26	39	0	0	0	128	0	54	0	33	0	15	29	29	15	55	0	0	42	0	0	25	79	106	28	65
CTC_RS07080	complement(1486158..1487717)	17	30	26	0	0	22	6	0	0	125	0	77	0	9	40	17	16	8	19	31	0	0	0	0	5	7	30	84	0	74
CTC_RS07085	complement(1488225..1489361)	0	0	0	0	20	0	0	0	0	78	0	0	6	0	0	0	0	22	22	0	42	11	0	36	0	0	20	30	0	17
CTC_RS07090	1489571..1490557	0	168	222	0	46	17	19	351	284	36	98	97	0	378	213	0	383	269	4	49	0	0	227	0	8	476	141	0	982	136
CTC_RS07095	1490554..1491831	0	724	375	0	81	67	0	825	167	37	270	205	0	945	213	0	1046	367	3	114	56	19	175	0	12	1238	290	5	1018	150
CTC_RS07100	complement(1492110..1492964)	0	0	31	36	161	121	0	37	31	104	169	84	0	17	37	72	118	133	30	0	224	0	44	238	9	39	54	48	0	269
CTC_RS07105	complement(1493239..1493844)	22	137	22	38	38	284	31	52	0	409	0	79	0	24	35	67	42	251	77	0	237	20	0	336	0	37	38	125	162	253
CTC_RS14720	complement(1494052..1494366)	43	0	84	121	73	164	30	0	0	581	0	0	23	46	0	43	0	121	162	0	76	79	0	388	0	177	74	175	0	913
CTC_RS07115	complement(1494815..1495078)	0	0	756	14	0	0	0	60	506	0	0	0	0	0	795	0	0	1175	0	0	0	0	0	0	0	42	176	0	0	73
CTC_RS07120	complement(1495389..1495556)	0	0	0	0	0	0	0	0	0	0	0	0	0	0	0	0	0	0	0	0	0	0	0	0	0	0	0	0	0	0
CTC_RS07125	complement(1495878..1496090)	63	111	250	36	538	243	88	370	502	55	981	560	0	344	591	32	355	238	120	1136	449	58	0	0	74	210	544	32	346	180
CTC_RS07130	complement(1496493..1497692)	901	1305	465	1739	1041	898	622	866	824	2397	509	418	415	653	577	1981	841	1445	795	927	1126	1773	1415	1476	529	741	753	2028	1125	1597
CTC_RS14510	complement(1497708..1499615)	2645	3139	1603	4144	3178	2231	2188	3288	2324	3533	1853	1658	1750	3088	2331	4953	1732	2837	3574	2320	3047	3902	3932	3137	2147	3125	2673	3909	3332	3963
deoD	complement(1499839..1500546)	2004	704	1484	2016	1360	1753	1041	1056	2000	834	1612	2124	2202	744	1363	1530	641	680	2144	1708	1250	2322	764	1955	1378	631	1768	757	520	1083
CTC_RS07145	complement(1500580..1501776)	745	317	755	822	766	886	362	408	881	587	866	768	790	324	456	1033	485	613	1027	970	939	1000	670	986	676	224	562	686	267	897
CTC_RS07150	complement(1501819..1503021)	112	138	254	209	210	487	109	124	89	407	281	258	102	73	122	159	63	274	106	402	527	196	217	626	46	116	58	160	82	414
CTC_RS07155	1503295..1504029	0	0	27	21	0	70	0	0	73	24	44	65	0	0	0	0	0	0	0	0	130	0	0	0	11	0	0	9	0	0
CTC_RS07160	1504353..1505129	0	0	0	0	0	0	0	0	34	91	21	0	0	19	54	18	0	0	27	0	0	0	48	0	0	29	30	27	0	0
CTC_RS07165	1505162..1506196	0	92	0	7	66	83	18	15	52	180	47	92	21	28	20	53	219	159	49	47	92	0	144	98	15	54	22	173	47	157
CTC_RS07170	1506254..1507051	17	30	83	29	57	86	35	79	0	174	20	90	18	0	0	77	32	95	21	61	30	16	47	0	10	14	29	198	31	24
CTC_RS07175	complement(1507135..1507764)	21	38	0	12	36	109	0	75	0	9	77	0	34	0	0	0	160	121	13	0	76	39	0	0	0	35	37	0	58	411
CTC_RS07180	complement(1507796..1508539)	0	32	18	10	31	93	0	32	72	24	43	64	10	0	0	18	0	170	6	0	129	33	0	55	11	0	62	0	99	567
CTC_RS07185	complement(1508640..1508939)	0	0	0	0	76	57	0	105	0	59	54	0	0	0	0	0	0	0	0	161	159	41	0	0	26	37	0	0	0	0
CTC_RS07190	complement(1509112..1509855)	0	0	0	10	0	0	0	0	0	0	0	0	10	20	0	0	0	0	6	0	0	0	0	0	0	0	0	0	0	0
CTC_RS07195	complement(1510107..1510838)	0	0	0	0	31	0	13	65	36	0	22	0	10	20	29	19	0	17	0	66	33	0	0	0	0	0	0	0	34	26
CTC_RS07200	complement(1511004..1511588)	0	0	23	0	0	29	0	0	0	10	0	0	0	0	36	12	0	43	7	83	82	0	64	139	0	0	40	0	42	0
CTC_RS07205	1512061..1513647	17	15	25	10	0	22	0	0	0	128	10	15	0	0	13	0	0	0	5	0	30	0	24	51	0	7	0	26	0	0
CTC_RS07210	1513648..1514598	0	0	14	0	0	0	0	0	0	31	0	25	0	0	0	7	0	13	13	0	0	0	0	0	0	0	0	0	0	0
CTC_RS07215	1514600..1515415	0	0	16	9	28	42	0	0	0	65	20	29	0	0	77	0	31	0	0	0	0	0	0	0	0	27	0	17	30	0
CTC_RS07220	1515429..1516370	0	0	0	0	0	18	0	0	0	38	34	0	0	16	45	0	0	40	9	0	0	0	0	86	0	12	0	0	0	20
CTC_RS07225	1516354..1517124	0	0	17	0	30	0	0	0	0	8	0	31	0	0	0	0	0	0	0	0	0	0	0	0	0	0	0	9	0	0
CTC_RS07230	1517168..1518019	0	28	16	9	0	0	0	0	0	62	0	0	0	0	49	0	0	0	10	0	0	0	44	48	0	13	27	16	0	22
CTC_RS07235	complement(1518084..1519420)	0	0	0	0	0	0	0	0	0	0	0	0	0	0	0	0	0	0	0	0	0	0	0	0	0	0	0	0	0	0
CTC_RS07245	complement(1519574..1521298)	0	28	15	9	13	10	5	9	0	24	9	69	0	42	36	4	15	7	7	0	14	0	43	24	0	13	13	4	14	44
CTC_RS07250	complement(1521295..1523103)	0	26	29	0	0	29	5	9	0	10	9	66	0	16	0	4	0	28	5	0	0	0	0	0	0	12	13	0	0	21
CTC_RS07255	complement(1523253..1524242)	0	96	67	62	116	139	19	64	27	95	32	72	15	118	42	14	0	179	43	0	121	50	151	82	0	45	70	83	0	290
CTC_RS07260	complement(1524369..1524887)	0	0	26	15	0	33	0	0	0	46	0	0	0	0	0	0	0	0	0	0	0	0	0	0	0	0	0	0	0	37
CTC_RS07265	1525054..1525524	0	151	0	0	243	73	0	33	170	63	68	101	31	31	0	29	0	54	27	0	51	0	79	346	0	47	0	15	104	122
CTC_RS07270	1525542..1526228	20	173	77	22	133	100	0	115	39	103	70	35	21	64	61	59	37	0	19	70	70	18	163	0	23	33	34	20	36	167
tlp	complement(1526295..1526522)	0	0	175	0	0	0	0	276	586	0	141	209	0	578	552	0	332	167	0	0	0	0	164	0	0	270	610	0	538	0
CTC_RS07280	1526776..1526877	0	0	0	0	0	0	0	0	0	0	0	0	0	0	0	0	0	0	0	0	0	0	0	0	0	0	0	0	0	0
CTC_RS07285	1527185..1527490	0	0	87	0	0	0	0	51	0	174	263	156	47	96	206	22	82	62	28	0	78	41	0	0	0	73	0	0	80	251
CTC_RS07290	1527471..1527860	35	0	68	20	29	22	60	121	308	288	412	61	46	75	108	70	0	32	87	0	184	0	0	0	0	100	59	71	0	98
CTC_RS14250	complement(1527941..1528858)	0	52	14	0	0	0	10	34	29	19	18	26	8	32	0	0	110	28	5	0	26	0	41	0	9	24	0	22	53	63
CTC_RS07300	complement(1528858..1531485)	5	36	5	3	35	26	7	0	10	2	18	18	0	6	8	3	10	10	3	0	0	2	14	0	0	28	9	3	9	15
CTC_RS07305	complement(1531482..1532165)	0	0	19	0	67	101	41	23	0	9	47	0	0	0	0	20	0	56	19	0	0	0	0	0	0	0	34	10	72	84
CTC_RS07310	complement(1532289..1533542)	0	19	21	0	0	0	15	0	0	14	26	19	0	0	33	0	0	0	0	0	0	0	0	32	0	0	0	11	20	31
CTC_RS07315	complement(1533553..1534245)	0	34	19	0	0	50	0	0	0	9	70	0	0	21	30	10	0	0	0	70	0	0	0	0	11	32	0	10	0	55

CTC_RS07320	complement(1534420..1534764)	39	550	116	133	465	300	54	183	310	51	745	415	42	255	304	118	146	110	86	280	347	72	108	354	92	259	134	0	0	56
CTC_RS07325	complement(1534786..1535958)	0	0	45	52	156	242	16	0	0	43	69	102	6	12	72	0	0	11	18	41	41	11	64	278	0	29	40	0	0	49
CTC_RS07330	complement(1536280..1537938)	41	365	184	101	387	405	34	199	177	85	252	273	26	115	215	62	198	229	38	87	216	75	517	319	48	141	154	70	178	243
CTC_RS07335	1538200..1538826	0	0	21	0	0	55	0	25	0	38	51	0	0	0	0	33	0	20	0	0	0	0	0	0	0	0	0	11	0	31
CTC_RS07340	complement(1538872..1539324)	30	0	117	67	0	266	41	174	118	39	248	105	32	97	46	30	167	28	0	107	158	27	82	719	0	49	51	23	0	42
CTC_RS07345	complement(1539348..1539818)	0	50	71	32	0	73	20	0	57	50	34	51	0	0	134	0	0	54	36	0	51	26	0	0	17	0	49	15	0	0
CTC_RS07350	complement(1539846..1540844)	0	24	53	53	92	86	0	0	0	89	64	60	0	0	0	0	0	0	0	0	0	0	0	41	4	0	0	0	0	0
CTC_RS07355	complement(1540913..1541209)	0	0	0	77	77	0	0	0	0	20	0	80	0	0	0	0	0	0	0	0	0	0	126	137	0	0	0	0	0	0
CTC_RS14515	complement(1541190..1541744)	24	0	0	0	0	93	0	0	0	53	0	0	0	0	38	0	0	0	0	0	0	22	0	0	0	0	0	0	0	0
CTC_RS07365	1542058..1542324	0	355	498	86	0	710	70	59	900	88	722	268	135	603	511	230	189	379	0	0	0	70	0	305	119	293	608	26	0	718
CTC_RS07370	1542746..1543096	0	0	38	0	0	0	53	0	0	0	0	0	21	0	60	0	0	0	0	0	0	0	0	0	0	32	66	0	70	0
CTC_RS07375	1543098..1543298	0	0	0	0	0	0	0	0	0	0	0	0	36	0	0	0	0	0	0	0	0	0	0	0	0	0	0	0	0	0
CTC_RS07380	1543431..1544165	0	0	54	0	0	0	38	0	0	0	0	0	29	20	114	0	0	34	0	0	0	0	0	0	22	0	0	9	33	104
CTC_RS15000	1544287..1544379	0	0	0	0	0	0	0	0	0	0	0	0	0	0	0	0	0	0	0	0	0	0	0	0	0	0	0	0	0	0
CTC_RS14860	complement(1544489..1544659)	0	0	311	0	0	0	0	0	312	0	0	0	0	0	0	0	0	185	0	0	0	0	0	0	0	0	136	0	0	0
CTC_RS07385	1545061..1545255	0	243	136	0	0	0	0	0	0	0	0	0	74	0	215	0	129	0	65	0	0	0	0	0	41	57	0	35	0	0
CTC_RS07390	1545261..1545680	0	0	32	18	55	82	44	0	0	14	38	0	0	0	0	16	0	30	0	0	0	0	0	97	38	27	0	0	58	0
CTC_RS07395	1545786..1546073	0	82	300	132	398	299	65	55	927	123	0	83	50	102	510	24	263	0	15	168	166	0	130	777	83	155	403	119	256	333
CTC_RS07400	complement(1546172..1546390)	0	4495	61	52	9368	0	0	1078	61	0	0	0	0	4846	383	187	9677	405	19	0	0	0	5283	0	36	4134	318	0	1233	0
CTC_RS07405	complement(1546407..1546628)	0	3312	60	34	2891	78	0	709	0	0	72	0	0	1714	94	0	1364	0	0	0	0	0	2858	0	0	1510	0	0	221	0
CTC_RS07410	1546799..1547686	0	187	90	17	52	39	0	35	90	20	72	27	49	132	118	23	0	14	38	109	108	42	126	0	27	101	183	31	28	130
CTC_RS07415	1547947..1548507	0	0	12	0	41	123	0	28	95	21	29	0	0	0	0	0	0	23	0	86	43	0	67	0	0	0	0	12	0	0
CTC_RS07420	1548735..1549283	0	0	0	0	0	0	0	0	0	0	0	0	0	0	0	0	0	0	0	0	0	90	2277	2262	0	0	0	0	0	0
CTC_RS07425	1549313..1549768	0	0	0	17	0	38	0	69	0	0	35	0	0	32	0	15	0	28	9	0	0	27	0	446	0	0	0	0	0	0
CTC_RS07430	1550040..1550384	0	0	0	0	66	350	0	0	0	17	140	69	0	0	0	0	73	147	49	280	139	0	0	118	0	0	0	80	0	167
CTC_RS07435	complement(1550478..1552106)	17	102	114	37	127	159	57	48	33	129	143	132	51	18	32	92	201	89	108	148	279	53	92	112	39	34	43	80	128	206
CTC_RS07440	complement(1553027..1553614)	0	0	0	0	0	0	0	0	0	50	164	690	0	0	0	220	429	549	72	0	61	0	0	69	0	0	0	0	0	0
CTC_RS07445	1553850..1554401	0	0	0	0	0	0	0	0	0	11	0	130	0	0	0	12	0	23	0	0	0	0	0	0	0	0	0	0	0	0
CTC_RS14525	complement(1554454..1554775)	0	0	0	0	0	0	0	0	41	18	50	74	0	0	0	0	0	39	0	150	74	0	0	0	0	0	36	0	38	0
eutM_1	complement(1555300..1555590)	813	408	91	26	158	118	256	0	92	81	221	0	74	0	216	246	0	44	248	332	0	1023	0	280	27	0	159	756	84	66
CTC_RS07460	complement(1555658..1556308)	2076	0	61	35	70	132	29	73	41	200	25	37	166	56	64	251	0	39	52	149	0	133	315	0	73	52	0	1236	75	0
CTC_RS07465	complement(1556308..1557906)	2345	104	83	14	129	32	29	49	33	181	25	15	81	37	0	132	32	24	80	30	120	279	140	76	84	56	29	1341	15	84
CTC_RS07470	complement(1557941..1558630)	1273	103	58	33	166	50	14	46	0	111	47	35	31	0	0	99	0	55	80	0	69	108	0	59	46	32	0	578	71	0
CTC_RS07475	complement(1558661..1559311)	1204	36	0	35	0	0	29	24	0	73	0	0	33	67	32	42	39	0	39	149	0	76	0	0	24	17	0	644	38	88
CTC_RS07480	complement(1559346..1559609)	2867	359	101	29	0	65	35	60	202	425	0	0	55	111	0	206	0	96	129	0	0	94	0	154	30	127	176	2422	0	0
CTC_RS07485	complement(1559606..1559908)	1450	78	44	0	0	0	0	0	88	97	0	0	24	48	0	90	83	0	168	0	79	123	0	0	79	37	0	953	0	0
eutJ_1	complement(1559924..1560772)	2610	224	63	27	54	81	22	93	0	167	0	0	34	52	25	176	30	15	178	114	0	161	88	0	84	39	0	1356	29	23
CTC_RS07495	complement(1560754..1561422)	909	71	60	23	34	26	56	0	0	199	48	36	32	22	0	92	38	19	70	0	0	0	0	61	36	0	139	575	18	57
CTC_RS07500	complement(1561429..1561872)	1552	53	0	52	52	0	0	0	0	206	36	0	33	0	0	92	0	29	48	109	0	28	84	0	54	25	0	712	55	0
CTC_RS07505	complement(1561869..1562222)	3588	134	75	86	130	0	53	178	75	384	0	0	82	0	178	250	0	36	168	0	0	70	105	115	179	63	0	1456	69	217
cutD	complement(1562262..1563215)	2521	0	42	24	48	54	39	49	28	325	34	0	91	15	0	82	26	13	165	51	25	52	0	0	58	12	0	976	0	20
cutC	complement(1563237..1565777)	4186	103	58	54	54	41	51	93	32	350	32	56	134	35	41	180	10	25	259	114	38	281	73	16	122	53	100	1836	68	75
CTC_RS07520	complement(1565916..1566224)	1137	77	0	0	0	0	0	0	0	134	0	0	23	47	68	132	0	82	206	157	0	0	121	0	26	0	0	423	0	0
CTC_RS07525	complement(1566246..1566542)	2139	0	45	0	0	0	31	53	0	318	54	241	73	0	71	161	0	0	229	0	0	0	0	0	27	0	0	694	0	0
CTC_RS07530	complement(1566649..1566915)	1114	0	0	0	0	0	0	0	0	66	0	0	0	0	0	77	0	47	64	181	0	0	0	0	0	0	0	257	0	0
CTC_RS07535	complement(1566902..1568017)	2688	0	12	34	41	0	17	0	24	217	43	0	97	0	38	159	23	23	244	0	21	78	67	0	64	0	62	924	0	17
CTC_RS07540	complement(1568461..1569291)	81	29	96	18	0	124	45	57	32	36	97	172	17	0	0	33	0	76	15	58	58	0	45	0	0	0	28	33	59	92
CTC_RS07545	complement(1569374..1570516)	491	270	209	220	261	467	41	124	140	550	281	271	63	218	128	161	177	354	260	42	1005	87	359	160	56	186	183	701	279	352
CTC_RS07550	complement(1571588..1572091)	27	47	26	45	0	0	19	31	53	12	32	0	14	0	0	0	0	0	0	192	47	25	148	0	0	0	46	0	49	38
CTC_RS14260	1572396..1572800	0	0	0	19	0	0	0	0	0	15	0	59	0	36	0	0	0	0	0	0	0	0	0	0	0	0	0	0	0	0
mnmA_2	complement(1573868..1574935)	25	22	37	14	0	48	0	0	25	39	75	67	7	14	20	13	24	71	6	0	22	0	0	76	7	21	22	39	46	108
CTC_RS07565	complement(1574978..1576114)	0	0	12	7	0	30	8	0	0	21	71	147	0	13	55	12	22	28	4	0	21	11	0	36	0	10	61	0	43	84
CTC_RS14265	1576490..1577302	0	233	196	66	56	106	34	194	66	145	59	59	27	126	181	75	124	132	47	0	59	46	92	0	39	206	171	34	151	141
CTC_RS07575	complement(1577340..1577690)	0	68	38	0	0	98	0	45	0	168	183	0	0	0	0	19	0	72	24	0	0	0	0	0	0	0	0	39	0	109
CTC_RS07580	complement(1578402..1579985)	17	60	130	183	217	370	24	70	143	117	218	136	36	55	66	56	151	308	50	244	302	20	118	154	25	99	124	85	170	206
cas2_2	complement(1580563..1580853)	46	0	0	79	79	296	0	162	92	20	221	246	124	151	0	70	173	0	7	166	164	0	128	140	27	0	0	24	0	0
cas1b_2	complement(1580854..1581852)	81	71	146	99	161	328	47	71	107	35	274	621	108	59	84	177	253	51	89	0	192	161	187	122	71	56	23	103	74	163
cas4_2	complement(1581862..1582353)	55	0	81	124	233	315	38	32	0	36	98	146	103	60	85	138	359	180	43	98	49	25	228	290	64	68	47	98	299	78
CTC_RS07600	complement(1582362..1584962)	29	36	41	47	44	235	18	12	41	53	90	206	19	17	24	46	87	63	26	65	147	43	65	110	32	26	18	26	28	41
cas5b	complement(1585019..1585789)	140	154	181	158	253	660	12	61	104	96	803	495	122	38	109	159	589	246	41	125	357	80	97	739	26	58	60	103	255	99
CTC_RS07610	complement(1585793..1586746)	439	597	516	416	457	1671	381	247	308	285	1668	1714	427	199	330	421	1190	557	227	608	802	429	958	1494	308	258	194	411	746	563
cas8b	complement(1586758..1588497)	186	170	229	149	382	758	169	172	31	188	882	521	120	109	60	180	623	229	67	514	577	185	279	585	87	128	80	138	536	132
cas6_2	complement(1588510..1589202)	59	171	134	121	496	796	108	114	424	205	789	586	52	296	151	138	582	238	80	279	483	107	269	1057	69	97	234	174	638	166
CTC_RS14725	complement(1589322..1589468)	0	0	0	0	0	0	0	0	0	0	0	0	0	0	0	0	0	0	0	0	0	0	0	0	0	0	0	0	0	0
CTC_RS07625	complement(1590320..1591906)	17	15	59	48	72	239	24	109	135	35	182	241	23	74	93	17	72	88	19	30	151	0	165	154	5	70	124	35	23	109
CTC_RS15005	complement(1592613..1593000)	0	61	34	0	0	0	0	0	0	0	0	0	0	0	0	0	0	131	0	0	0	0	0	0	0	0	0	0	0	0
CTC_RS07635	complement(1593444..1594592)	0	103	116	0	40	0	8	151	46	5	70	62	3	217	128	6	110	88	15	0	0	11	97	0	0	306	101	0	406	100
CTC_RS14540	complement(1594969..1595304)	0	0	0	45	68	103	0	47	79	18	0	0	0	0	62	0	0	0	0	0	0	0	0	0	0	0	0	0	0	0
CTC_RS07645	complement(1595395..1595637)	0	0	0	0	0	0	38	0	0	24	0	0	0	0	0	0	0	0	0	0	0	0	0	0	0	0	0	0	0	0
CTC_RS07650	complement(1595606..1595929)	0	73	41	24	71	213	29	0	82	46	50	0	22	45	0	21	0	0	0	0	74	0	0	0	0	17	72	0	0	0
CTC_RS07655	complement(1596262..1596732)	0	0	14	0	0	37	0	33	0	0	0	0	0	16	0	0	0	0	0	0	0	0	0	0	0	24	0	0	0	0
CTC_RS14280	complement(1596867..1597955)	0	0	24	7	0	0	0	0	25	11	15	22	7	13	0	0	46	23	0	0	0	0	0	0	0	31	0	0	23	0
CTC_RS07665	1598120..1598677	48	468	929	308	1458	1545	0	282	1149	85	1498	1668	0	315	677	208	633	885	99	954	1072	267	1070	1751	0	120	748	49	264	1013
CTC_RS07670	1598678..1599271	23	319	515	283	1081	1190	16	212	809	75	1204	1406	0	148	600	229	531	789	104	570	846	272	691	1165	0	56	546	23	331	904
CTC_RS07675	complement(1599282..1600583)	0	55	26	26	114	60	25	30	62	18	31	46	6	45	89	0	0	5	11	0	18	33	72	31	12	47	27	0	19	44
CTC_RS07680	1600701..1600883	0	130	0	42	125	94	0	0	0	16	0	0	0	0	0	0	0	0	0	0	0	0	0	0	0	0	0	0	0	0
CTC_RS07685	1601044..1602771	55	494	777	144	743	708	43	528	603	84	1051	622	71	631	437	142	613	381	59	728	775	122	583	401	92	505	510	76	383	910
CTC_RS07690	complement(1602895..1603476)	93	82	160	52	118	118	16	257	367	183	166	123	124	226	36	304	173	326	190	166	288	64	128	140	136	154	120	142	0	198
CTC_RS07695	complement(1603541..1604416)	123	162	121	174	105	216	64	162	30	297	165	136	66	167	96	272	86	506	150	166	464	57	213	186	72	115	185	165	112	175
CTC_RS15010	complement(1604409..1604558)	90	0	0	0	0	0	0	0	0	0	0	159	0	0	0	0	0	0	0	0	0	0	249	0	53	0	0	0	0	0

CTC_RS07700	complement(1604649..1605866)	11	39	66	19	38	85	54	13	44	44	13	137	30	24	0	22	21	114	45	79	98	20	0	33	13	18	76	23	20	31
CTC_RS07705	complement(1605838..1606506)	40	35	40	11	0	77	42	47	40	26	48	107	32	22	31	102	75	114	64	72	143	0	56	61	12	67	35	51	37	86
CTC_RS07710	complement(1606861..1607079)	62	542	182	209	105	157	170	359	0	27	73	109	99	134	192	156	806	58	155	221	0	113	170	186	36	102	318	0	224	175
CTC_RS14865	complement(1607291..1607458)	0	424	1544	0	0	0	0	187	477	0	96	0	0	697	624	0	451	603	0	0	0	0	0	0	0	1065	690	0	292	228
CTC_RS15015	complement(1607642..1607781)	0	0	0	0	0	0	0	0	0	0	0	0	0	0	0	0	0	0	0	0	0	0	0	0	0	0	0	0	0	0
CTC_RS07715	complement(1607756..1608070)	0	75	42	48	146	164	0	0	0	94	306	76	23	0	0	0	0	161	0	0	0	0	0	0	0	35	74	0	0	0
CTC_RS07720	complement(1608151..1608726)	23	0	115	26	119	180	16	164	0	226	251	269	13	0	36	65	197	308	0	0	228	0	65	0	0	39	40	6	43	300
CTC_RS07725	complement(1608982..1610160)	0	0	23	26	0	0	0	0	0	60	27	20	6	0	18	0	21	21	11	0	20	0	32	35	0	0	0	29	21	0
CTC_RS07730	complement(1610174..1610893)	0	0	28	21	32	0	0	0	0	33	0	0	10	0	0	9	70	18	35	0	33	0	52	0	0	31	32	19	0	27
CTC_RS07735	1611186..1611941	89	94	88	172	182	205	86	146	212	164	43	126	105	174	222	248	234	184	231	448	158	98	99	431	178	118	184	191	357	279
CTC_RS07740	complement(1612018..1613811)	331	304	104	334	115	154	161	114	60	46	260	366	402	73	129	38	84	21	40	54	0	304	250	91	380	125	52	184	363	64
htpG	complement(1614004..1615878)	115	101	57	57	147	92	149	84	85	364	154	140	77	55	34	51	40	41	93	52	51	79	189	109	59	36	62	103	39	72
CTC_RS07750	1616114..1616332	0	108	0	0	0	0	0	0	0	0	0	0	0	0	0	0	0	0	0	0	0	0	0	0	0	0	0	0	0	0
CTC_RS14870	complement(1616366..1616536)	0	0	661	0	0	0	0	0	469	0	0	279	0	0	245	0	0	481	0	0	0	0	0	0	0	131	0	0	0	0
CTC_RS14550	complement(1616560..1616754)	69	122	4706	0	0	1238	0	161	1781	0	0	122	0	150	2366	0	388	3474	0	0	613	0	383	0	0	287	1308	0	126	295
CTC_RS07755	complement(1617147..1617842)	175	239	191	110	181	272	40	113	115	144	162	343	21	84	90	49	72	273	55	209	241	196	268	409	34	80	133	40	176	220
CTC_RS07760	complement(1617863..1619299)	42	33	37	58	128	84	0	22	74	58	45	133	5	0	15	14	35	35	38	34	50	26	0	57	22	0	16	5	17	0
CTC_RS07765	complement(1619309..1620061)	0	0	0	0	0	69	0	0	106	8	0	0	0	0	84	9	0	34	6	0	0	0	0	54	0	0	62	0	0	0
CTC_RS07770	complement(1620048..1620773)	0	0	37	0	63	0	0	0	110	24	44	148	10	0	58	19	0	35	29	0	0	0	51	0	11	31	0	9	135	53
CTC_RS07775	complement(1620869..1622110)	33	172	418	61	138	402	38	82	409	266	155	307	23	283	220	22	142	102	51	117	154	20	90	164	51	171	243	11	20	370
CTC_RS07780	complement(1622284..1623747)	65	49	0	0	16	0	13	43	18	20	11	0	5	50	0	0	69	0	6	0	16	0	0	0	0	23	16	23	34	26
CTC_RS07785	complement(1623829..1624878)	1178	158	456	131	22	681	3625	75	229	478	245	1114	2244	139	240	418	1177	386	146	184	137	154	213	543	2689	181	442	579	164	256
CTC_RS07790	complement(1624939..1625622)	474	0	39	33	34	202	1316	23	78	302	117	244	496	21	61	40	240	37	44	212	140	73	0	119	638	33	203	90	0	56
CTC_RS07795	complement(1625990..1627435)	0	16	46	0	16	12	26	22	0	16	44	17	15	35	15	5	52	35	15	67	0	9	26	0	5	39	0	14	17	13
CTC_RS07800	complement(1627576..1627896)	168	222	166	95	214	54	0	245	166	110	100	149	90	274	196	127	236	158	212	301	0	0	116	0	74	209	506	128	76	0
CTC_RS07805	1628071..1628256	0	0	0	0	0	93	50	0	0	32	173	0	0	0	0	0	0	0	0	0	0	0	0	0	0	0	0	0	0	0
CTC_RS07810	complement(1628308..1629249)	0	101	198	162	292	220	40	117	198	376	17	291	61	233	245	94	80	269	117	257	330	66	198	475	67	166	320	175	104	448
CTC_RS07815	1629413..1630360	0	0	0	8	24	0	10	0	0	12	0	0	0	0	0	0	0	0	0	0	25	0	0	86	0	0	0	0	0	0
CTC_RS07820	complement(1630409..1631362)	14	50	125	24	120	90	68	16	0	68	17	150	15	0	66	21	53	60	27	51	50	26	0	0	0	23	24	36	51	241
CTC_RS07825	complement(1631534..1632244)	0	0	19	11	32	48	0	22	150	33	0	67	30	0	59	19	142	53	15	0	34	0	0	115	0	0	33	0	35	81
CTC_RS07830	1632367..1633356	14	48	67	15	23	35	9	16	81	72	49	72	15	30	21	7	25	64	21	0	72	13	151	41	24	0	23	28	0	136
CTC_RS07835	complement(1633654..1635354)	8	28	55	9	13	162	11	19	63	99	14	0	4	9	25	12	15	37	19	57	28	7	88	550	5	10	27	75	58	23
CTC_RS07840	complement(1635668..1637026)	0	209	10	6	34	0	0	116	20	9	95	123	5	399	31	10	0	37	3	36	35	18	27	0	0	230	119	18	36	42
CTC_RS07845	complement(1637452..1638834)	0	669	87	6	149	62	20	364	97	4	604	35	5	1593	174	10	347	73	15	70	17	9	81	0	11	1273	168	5	177	139
CTC_RS07850	complement(1639001..1640692)	144	0	55	50	68	102	149	28	32	117	24	56	36	26	0	40	60	52	62	57	42	15	22	217	28	17	14	122	87	102
CTC_RS07855	complement(1641128..1641574)	30	106	89	51	51	116	0	70	179	26	108	0	65	98	0	15	113	0	0	0	0	28	83	182	18	50	0	15	55	0
CTC_RS07860	1642117..1643286	1282	223	364	548	225	398	937	202	183	601	247	184	608	138	108	690	108	249	214	207	286	604	319	418	793	96	178	176	126	360
CTC_RS07865	1643432..1644829	0	68	105	0	0	25	0	79	76	13	34	34	5	42	30	0	54	145	3	35	34	0	0	0	6	80	66	5	70	82
CTC_RS07870	complement(1644873..1645526)	289	10137	9150	630	3435	1160	242	15175	8046	686	4719	1423	265	13990	5212	489	18323	8150	182	1331	805	417	5964	1432	230	15980	6346	463	20566	7269
rpsA	complement(1645594..1646748)	222	329	161	311	179	134	170	177	162	251	153	41	225	355	182	442	87	274	278	84	228	247	420	211	240	368	161	280	319	149
CTC_RS07880	1646937..1647281	0	69	0	0	0	0	0	0	0	0	47	0	0	0	0	0	0	0	0	0	0	0	0	0	0	0	0	0	0	0
CTC_RS07885	complement(1647336..1647680)	470	344	193	354	66	50	216	228	310	51	140	208	293	170	0	276	73	183	259	0	208	252	216	0	552	130	269	40	213	167
CTC_RS07890	complement(1647708..1649165)	232	195	100	314	63	47	147	130	18	89	77	213	238	60	72	93	346	156	233	33	49	298	128	56	288	100	111	66	101	184
CTC_RS07895	complement(1649283..1649984)	0	0	0	0	0	25	27	45	0	17	23	34	0	0	30	0	72	18	12	0	34	0	0	0	0	16	0	0	52	27
CTC_RS07900	complement(1649962..1651578)	0	44	8	14	57	21	0	19	33	22	80	44	13	18	0	21	62	23	24	60	44	8	0	25	10	48	0	9	15	0
CTC_RS07905	complement(1651759..1652883)	0	0	12	7	82	0	0	42	0	0	57	42	0	52	56	0	45	34	0	0	0	0	33	0	7	99	82	6	65	51
CTC_RS07910	1653015..1653521	0	0	0	0	0	0	0	0	0	0	0	0	0	58	0	13	0	0	0	0	0	12	0	80	0	0	0	0	0	0
ytfJ	1653539..1653955	32	114	159	0	0	41	0	75	0	0	424	114	0	246	151	0	60	0	0	0	57	0	0	0	0	161	0	0	59	46
scpB	complement(1654115..1654684)	0	0	47	13	80	121	0	0	0	93	28	0	0	0	0	0	89	89	15	85	42	22	65	0	0	0	0	72	86	168
CTC_RS07925	complement(1654659..1655423)	0	0	35	0	0	45	0	0	0	85	42	31	0	0	27	0	33	66	6	63	63	0	0	0	0	15	0	36	0	75
CTC_RS07930	complement(1655502..1656617)	0	191	179	14	21	77	0	353	144	26	475	107	13	577	169	49	113	68	23	87	0	11	67	0	0	491	125	12	220	52
CTC_RS07935	complement(1656937..1657752)	729	465	277	776	309	296	995	473	131	253	1103	527	867	951	488	1565	835	465	1279	593	352	973	549	349	1011	589	313	455	963	317
xerD	complement(1657784..1658626)	273	169	47	290	136	245	144	93	63	224	343	113	163	52	199	461	30	225	270	0	255	353	89	48	216	159	110	245	204	91
spoIIM	complement(1659010..1659636)	0	0	42	0	0	0	0	38	0	0	0	38	0	0	0	0	0	0	0	0	0	0	0	0	0	0	37	0	0	0
CTC_RS07955	1659775..1660320	0	739	219	0	210	0	0	807	342	0	1236	131	0	1354	346	0	739	46	0	177	88	0	342	0	0	1126	297	0	1866	105
CTC_RS07960	complement(1660410..1660886)	0	0	28	0	0	72	0	0	0	124	0	0	15	0	88	0	0	0	18	0	0	0	0	0	0	0	0	0	0	40
CTC_RS07965	complement(1661019..1663010)	7	60	120	73	104	121	89	24	67	44	77	228	40	37	116	44	63	83	17	24	108	6	37	41	32	34	81	28	49	58
CTC_RS14555	complement(1663518..1663610)	0	0	0	0	0	0	0	0	0	0	0	0	0	0	0	0	0	0	0	0	0	0	0	0	0	0	0	0	0	0
CTC_RS14285	complement(1663821..1664825)	0	0	0	4	650	463	0	0	0	0	0	0	0	0	0	0	0	0	0	0	0	0	0	0	0	0	0	0	0	0
CTC_RS07975	complement(1664842..1665102)	26	136	204	29	307	462	0	0	0	0	0	0	0	0	0	0	0	0	0	0	0	0	357	156	0	0	0	0	94	73
CTC_RS07980	complement(1665120..1665353)	0	0	0	0	0	0	0	0	0	0	0	0	0	0	0	0	0	0	0	0	0	0	0	0	0	0	0	0	0	0
CTC_RS07990	complement(1665626..1666054)	0	0	0	0	0	0	0	0	0	0	0	0	0	0	0	0	0	0	0	0	0	0	0	0	0	0	0	0	0	0
CTC_RS14290	complement(1666338..1668320)	0	0	0	0	0	0	0	0	0	0	0	6	0	0	0	0	0	0	0	12	0	0	0	0	0	0	0	0	12	29
CTC_RS08005	complement(1668304..1668492)	0	0	0	0	0	0	0	0	0	0	0	0	0	0	0	0	0	0	0	0	0	0	0	0	0	0	0	0	0	0
CTC_RS08010	complement(1668482..1670374)	0	0	0	0	0	0	0	0	0	0	0	0	0	0	0	0	0	0	0	0	0	0	0	0	0	0	0	0	26	20
CTC_RS08015	complement(1670386..1671108)	0	0	0	0	0	0	0	0	0	0	0	0	0	0	0	0	0	0	0	0	0	0	0	0	0	0	0	0	0	0
CTC_RS08020	complement(1671112..1673850)	0	0	0	0	0	0	0	0	0	0	0	0	0	0	0	0	0	0	10	0	4	0	0	0	0	0	0	0	0	0
CTC_RS08025	complement(1673831..1674166)	0	0	0	0	0	0	0	0	0	0	0	0	0	0	0	0	0	0	0	0	0	0	0	0	0	0	0	0	0	0
CTC_RS08030	complement(1674181..1674483)	0	0	0	0	0	0	0	0	0	0	0	0	0	0	0	0	0	0	0	0	0	0	0	0	0	0	0	0	0	0
CTC_RS08035	complement(1674530..1675156)	11	0	0	0	0	0	0	0	0	0	0	0	0	0	0	0	0	0	0	0	0	0	0	0	0	0	0	0	0	0
CTC_RS08040	complement(1675175..1675558)	0	0	0	0	0	0	0	0	0	0	0	0	0	0	0	0	0	0	0	0	0	0	0	0	0	0	0	0	0	0
CTC_RS08045	complement(1675561..1675887)	0	0	0	0	0	0	0	0	0	0	0	0	0	0	0	0	0	0	0	0	0	0	0	0	0	0	0	0	0	0
CTC_RS08050	complement(1675874..1676203)	0	36	0	0	0	0	0	0	0	0	0	0	0	0	0	21	0	0	0	0	0	0	0	0	0	0	0	0	0	0
CTC_RS08055	complement(1676218..1676535)	0	0	0	0	0	0	0	0	0	0	0	0	0	0	0	0	0	0	0	0	0	0	0	0	0	0	0	0	0	0
CTC_RS08060	complement(1676589..1677404)	0	0	0	0	0	0	0	0	0	0	0	0	0	0	0	0	0	0	0	0	0	0	0	0	0	0	0	0	0	0
CTC_RS08065	complement(1677494..1678117)	0	0	0	0	0	0	0	0	0	0	0	0	0	0	0	0	0	0	0	0	0	0	0	0	0	0	0	0	0	0
CTC_RS08070	complement(1678199..1679071)	0	0	0	0	0	0	0	0	0	0	0	0	0	0	0	0	0	0	0	0	0	0	0	0	0	0	0	0	0	0
CTC_RS08075	complement(1679064..1680335)	0	0	0	0	0	0	0	0	0	0	0	0	0	0	0	0	0	0	0	0	0	0	0	0	0	0	0	8	0	8
CTC_RS08080	complement(1680351..1682081)	0	0	0	0	0	0	0	0	0	0	0	0	0	0	0	0	0	0	0	0	0	0	0	0	0	0	0	0	0	0
CTC_RS08085	complement(1682210..1682779)	0	0	0	0	0	0	0	0	0	0	0	0	0	0	0	0	0	0	0	0	0	0	0	0	0	0	0	0	0	0

CTC_RS08090	complement(1682965..1683393)	0	0	0	0	0	0	0	0	0	0	0	0	0	0	0	0	0	0	0	0	0	0	0	0	0	0	0	0	0	0
CTC_RS08095	complement(1683520..1683708)	0	0	0	0	0	0	0	0	0	0	0	0	0	0	0	0	0	0	0	0	0	0	0	0	0	0	0	0	0	0
CTC_RS08100	complement(1683954..1684307)	0	0	0	0	0	0	0	0	0	0	0	0	0	0	0	0	0	0	0	0	0	0	0	0	0	0	0	0	0	0
CTC_RS08105	complement(1684323..1684835)	0	0	0	0	0	0	0	0	0	0	0	47	0	0	0	0	0	0	0	0	0	0	0	0	0	0	0	0	0	0
CTC_RS08110	complement(1684984..1685232)	0	0	0	0	0	0	0	0	0	0	0	0	0	0	0	0	0	0	0	0	0	0	0	0	0	0	0	0	0	0
CTC_RS14295	complement(1685300..1686064)	0	0	0	0	0	0	0	0	0	0	0	0	0	0	0	0	0	0	0	0	0	0	0	0	0	0	0	0	0	0
CTC_RS08120	complement(1686429..1686695)	0	0	0	0	0	0	0	0	0	0	0	89	0	0	0	0	0	0	16	91	269	0	0	0	0	0	0	0	0	0
CTC_RS08125	complement(1687004..1689745)	0	0	0	0	0	0	0	0	0	11	41	9	0	0	0	0	0	0	0	0	0	0	0	0	0	0	0	0	0	0
CTC_RS08130	complement(1690009..1691229)	0	0	0	0	0	0	0	0	0	0	0	0	0	0	0	0	0	0	0	0	20	0	0	0	0	0	0	0	0	0
CTC_RS08135	complement(1691931..1692686)	0	0	0	0	0	0	0	0	0	0	0	0	0	0	0	0	0	0	0	0	0	0	0	0	0	0	0	0	0	0
CTC_RS08140	complement(1692999..1693367)	0	0	0	0	0	0	0	0	0	0	0	0	0	0	0	0	0	0	0	0	0	0	0	0	0	0	0	0	0	0
CTC_RS08145	1693628..1693849	0	0	0	0	0	0	0	0	0	0	0	0	0	0	0	0	0	0	0	0	0	0	0	0	0	0	0	0	0	0
CTC_RS08150	complement(1694789..1695271)	0	0	0	0	0	0	0	0	0	6	33	49	0	0	0	0	0	0	0	0	0	0	0	0	0	0	0	0	0	0
CTC_RS08155	complement(1695271..1696272)	0	0	0	0	0	0	0	0	0	0	0	0	0	0	0	0	0	0	0	0	24	0	0	0	0	0	0	0	0	0
spo0A	complement(1696400..1697218)	1675	1839	1380	2851	2015	1494	2455	2172	3196	995	3758	2419	3322	2663	2971	4021	1432	3347	2518	2215	2862	3295	2301	1442	2827	2129	3298	781	1049	2153
spoIVB	complement(1697400..1698608)	0	39	88	19	57	14	0	117	66	34	40	39	0	121	191	0	83	21	11	40	0	10	0	101	0	268	96	0	142	16
recN	complement(1698805..1700502)	32	42	125	85	189	152	16	65	63	111	95	155	30	86	86	72	149	149	78	114	99	44	0	96	9	112	96	53	145	214
CTC_RS08175	complement(1700507..1700968)	59	103	144	83	447	261	0	204	116	51	70	52	78	32	91	59	164	274	138	0	129	134	0	176	34	48	151	134	0	290
CTC_RS08180	complement(1700987..1701811)	16	144	113	102	167	209	34	172	32	43	0	203	26	124	76	41	31	169	57	0	145	90	45	148	58	68	28	42	60	302
CTC_RS08185	complement(1701820..1702638)	17	58	81	84	112	42	80	77	163	65	49	58	53	89	51	83	185	247	114	177	88	106	46	50	58	82	57	76	150	140
dxs	complement(1702660..1704516)	33	64	43	66	148	111	65	136	72	64	65	154	47	71	23	55	109	191	133	104	193	27	121	44	21	84	87	63	26	103
CTC_RS08195	complement(1704519..1705085)	0	42	47	40	202	30	0	83	141	115	57	84	38	77	74	36	44	156	52	85	169	0	132	144	14	39	0	24	87	203
CTC_RS08200	complement(1705839..1706705)	16	246	46	123	53	99	43	109	154	48	93	193	92	34	97	204	145	329	101	167	138	43	43	94	27	64	214	167	113	133
CTC_RS08205	complement(1706731..1706955)	0	0	0	203	204	77	124	0	0	52	0	0	96	65	93	61	0	56	94	0	106	0	0	0	35	99	0	92	0	85
xseA	complement(1707049..1708239)	23	80	123	32	115	87	39	53	45	109	54	160	18	111	123	109	64	159	46	0	161	21	31	0	13	75	117	110	144	97
CTC_RS08215	complement(1708239..1709075)	0	28	111	68	82	103	145	150	96	134	96	342	56	122	25	81	151	136	173	0	171	74	178	146	19	67	83	140	88	137
nusB	complement(1709091..1709552)	0	0	29	17	50	37	61	136	58	38	0	258	47	63	91	147	218	82	37	314	52	0	162	0	34	97	100	60	53	124
CTC_RS08225	complement(1709702..1710097)	1877	4792	3291	2100	3010	3983	2591	3915	4114	1074	6657	5123	1567	6654	10011	2632	5415	3549	1900	4520	3806	1817	3346	5037	1983	4544	9075	1328	3657	2807
CTC_RS08230	complement(1710200..1710709)	0	1116	287	0	270	68	0	494	367	0	3325	0	0	1952	411	0	1533	199	0	285	94	0	220	0	16	1688	273	0	1251	338
spoIIIAG	complement(1710768..1711385)	0	154	151	0	111	56	0	127	130	0	312	77	0	569	204	0	82	61	0	0	39	0	0	33	0	235	188	11	199	62
spoIIIAF	complement(1711397..1711978)	0	0	23	0	0	30	0	81	0	0	110	41	0	75	0	0	43	0	0	166	0	0	0	0	0	19	0	0	0	0
spoIIIAE	complement(1711983..1713143)	0	123	57	0	20	0	0	68	115	0	208	0	0	177	18	0	152	44	0	42	0	0	64	0	0	116	40	0	85	66
spoIIIAD	complement(1713154..1713540)	0	184	69	0	0	0	0	122	69	0	332	0	0	265	54	0	130	33	11	0	0	0	96	0	0	173	60	0	0	0
spoIIIAC	complement(1713550..1713747)	0	0	0	0	0	0	0	0	0	0	0	0	0	0	0	0	0	0	0	0	0	0	0	0	0	0	0	0	0	0
spoIIIAB	complement(1713760..1714281)	0	0	0	0	0	33	0	30	0	0	31	0	0	42	0	0	0	0	0	0	0	0	0	0	0	43	0	0	0	0
spoIIIAA	complement(1714298..1715206)	0	209	59	0	76	0	0	17	29	0	141	0	0	193	46	0	56	28	0	213	0	0	0	0	0	283	0	0	108	21
CTC_RS08270	complement(1715369..1715776)	232	233	424	243	449	422	69	270	720	434	433	761	159	287	411	267	309	403	333	1008	235	395	1006	748	136	356	568	236	361	329
efp	complement(1715864..1716421)	775	765	810	944	1068	1205	752	903	1292	1302	835	941	854	1181	1240	848	769	1271	1345	1387	1329	756	1806	1496	824	1042	1246	1146	1056	2233
CTC_RS08280	complement(1716499..1717566)	228	89	174	200	279	178	253	118	225	426	196	156	176	247	157	261	260	468	297	362	179	58	210	76	111	241	22	386	184	377
aroQ	complement(1717579..1718019)	92	323	121	121	364	313	42	143	121	455	73	0	98	199	48	108	0	488	280	110	380	0	85	0	72	177	105	374	167	130
CTC_RS08290	complement(1718119..1718622)	0	0	0	15	0	68	37	0	0	35	64	0	0	87	0	0	50	25	17	0	0	0	74	0	0	22	46	0	49	0
CTC_RS08295	complement(1718687..1719085)	0	0	0	0	0	0	0	0	0	0	0	0	0	37	0	0	0	32	0	0	0	0	0	0	0	28	0	0	0	48
CTC_RS08300	complement(1719075..1719530)	0	0	0	0	0	0	0	0	0	0	0	0	0	32	46	0	0	0	0	0	105	0	0	0	0	0	51	0	54	42
CTC_RS08305	complement(1719542..1720303)	0	31	0	0	0	0	0	0	105	0	0	0	0	0	28	0	0	0	0	0	0	0	0	0	0	0	0	0	0	0
CTC_RS08310	complement(1720456..1720908)	0	52	0	0	0	0	0	0	0	0	0	0	0	0	46	0	56	0	0	0	0	0	0	0	0	0	0	0	0	0
CTC_RS08315	complement(1720905..1721336)	0	0	0	0	0	0	22	0	0	0	0	0	0	0	0	0	0	29	5	0	0	0	0	0	0	0	0	0	0	0
CTC_RS08320	complement(1721320..1721769)	0	0	0	0	0	0	0	0	0	0	0	0	0	0	0	0	0	0	0	0	0	0	0	0	0	0	0	0	0	0
CTC_RS08325	complement(1721777..1722166)	0	0	34	0	0	44	0	0	0	0	0	0	0	0	54	17	0	32	0	0	61	0	0	0	0	0	0	0	63	0
CTC_RS08330	complement(1722198..1723253)	0	45	0	0	0	16	0	0	0	6	0	0	0	0	0	0	0	12	0	0	0	0	0	0	0	11	0	0	0	18
CTC_RS08335	complement(1723411..1724859)	0	0	0	0	0	12	0	11	0	4	11	16	0	10	0	0	0	9	0	0	0	0	0	0	0	0	16	5	0	0
CTC_RS08340	complement(1724971..1726026)	38	67	50	7	109	131	35	15	25	45	61	68	7	55	0	19	0	36	28	46	113	12	35	0	8	21	44	33	0	73
CTC_RS08345	1726190..1727914	0	41	46	13	13	120	16	64	31	123	19	28	4	8	0	28	0	37	23	0	111	22	43	47	18	39	54	56	28	100
CTC_RS08350	complement(1727958..1728506)	0	43	0	28	0	94	0	0	0	43	0	0	13	0	38	0	46	0	15	88	0	23	0	74	0	20	84	0	0	0
CTC_RS08355	complement(1728507..1729154)	42	0	21	24	0	0	29	49	0	46	0	37	11	23	0	0	0	0	23	149	0	19	0	0	12	0	0	11	0	0
CTC_RS08360	1729341..1730264	0	0	0	0	0	19	0	0	0	0	17	26	0	16	0	0	0	0	0	0	0	0	0	0	0	0	0	0	27	0
CTC_RS08365	complement(1730290..1731063)	70	123	155	217	178	290	145	163	242	206	187	278	112	189	163	255	196	262	214	437	216	48	241	368	143	274	180	195	285	149
CTC_RS14730	complement(1731065..1731202)	0	172	0	0	0	0	0	0	0	0	0	0	0	0	0	0	0	0	31	0	0	0	0	0	0	0	0	0	0	0
uraA	complement(1731350..1732678)	112	393	655	212	483	1284	84	249	502	429	1415	808	65	220	552	400	3075	1496	346	1147	1674	131	871	1440	69	168	593	352	1330	1053
pyrR	complement(1732758..1733291)	0	133	112	57	86	161	0	59	50	66	90	179	14	110	236	64	378	332	68	91	314	23	0	0	15	21	174	39	230	108
CTC_RS08380	complement(1733457..1734374)	15	155	58	83	25	38	0	34	58	51	105	78	16	96	23	45	82	110	74	105	182	27	41	0	26	49	51	37	27	63
lspA	complement(1734349..1734819)	29	50	0	0	0	0	20	0	57	25	0	0	0	31	0	58	0	54	18	0	102	0	79	0	0	47	0	15	0	0
CTC_RS08390	complement(1734873..1735505)	0	150	189	72	72	109	15	174	0	47	355	113	23	324	133	43	40	200	94	0	340	20	59	64	13	300	110	54	620	91
aroA	complement(1735558..1736877)	0	18	30	52	0	39	21	0	0	54	24	90	5	11	0	72	19	48	39	0	36	9	0	0	6	8	35	89	0	73
aroF	complement(1736877..1737674)	135	89	183	258	172	130	234	79	100	178	101	120	163	174	158	282	253	254	341	364	300	311	94	51	129	252	174	181	185	180
CTC_RS08405	complement(1737686..1738378)	332	719	384	308	546	547	337	500	424	503	417	706	448	433	303	462	619	603	423	489	759	385	539	646	275	726	636	337	496	553
aroB	complement(1738495..1739568)	101	99	99	107	64	177	104	117	124	99	105	44	60	55	137	101	23	94	40	360	178	0	0	152	48	83	65	38	23	18
CTC_RS08415	complement(1739632..1740252)	783	974	1092	1733	1550	1596	766	735	903	942	1553	730	924	825	507	812	772	1254	897	1324	1599	1439	1382	2163	715	522	747	376	711	957
CTC_RS08420	complement(1740269..1741045)	696	611	308	1041	679	532	432	324	103	559	693	307	483	433	243	920	162	473	971	747	739	623	336	419	418	518	328	221	474	691
CTC_RS08425	complement(1741042..1741317)	392	344	241	525	374	187	406	456	194	471	233	692	418	265	152	629	183	229	401	526	433	495	0	443	517	202	252	174	267	417
sepF	complement(1741325..1741786)	995	667	432	1181	1141	597	1171	988	405	422	870	362	1219	792	272	2351	655	932	1795	1047	1243	859	404	352	961	847	502	565	1116	1120
CTC_RS08435	complement(1741801..1742469)	354	496	298	502	617	283	739	447	399	415	973	250	755	503	376	1400	528	625	826	362	787	593	725	304	605	418	104	349	404	401
CTC_RS08440	complement(1742542..1743273)	92	32	18	21	0	47	0	43	0	56	110	130	30	60	29	9	0	69	52	0	196	0	102	0	22	15	32	28	101	105
CTC_RS08445	complement(1743288..1743626)	40	70	0	45	270	153	83	93	79	17	47	282	21	173	62	40	74	187	88	285	494	37	0	120	0	33	0	20	0	226
CTC_RS08450	complement(1743646..1744362)	75	397	278	106	288	216	130	154	149	157	45	100	50	20	293	104	106	283	148	135	400	52	208	114	11	171	97	58	171	160
CTC_RS08455	complement(1744418..1745194)	70	0	51	39	30	44	0	61	34	23	21	0	0	38	0	26	97	196	66	0	92	16	48	52	0	43	0	0	0	49
spoVE	complement(1745386..1746492)	49	86	84	145	145	31	84	57	48	80	44	22	98	106	38	160	23	91	108	87	151	90	67	37	64	101	168	50	22	260
mraY	complement(1746512..1747471)	84	0	69	40	48	108	39	131	83	123	84	50	45	91	87	156	53	106	104	50	50	78	78	0	83	82	24	129	77	100
CTC_RS08470	complement(1747502..1748881)	29	17	39	83	50	12	20	0	19	30	82	35	73	42	30	99	37	64	52	0	35	99	54	0	52	57	17	35	0	97
CTC_RS08475	complement(1748894..1750351)	0	16	36	94	47	47	45	43	55	20	22	16	40	90	43	79	87	52	61	66	98	43	51	28	33	23	48	38	42	26
CTC_RS08480	complement(1750427..1752613)	306	721	426	480	660	473	318	522	427	174	853	502	355	639	484	517	623	709	340	752	1116	272	427	214	232	606	610	200	281	855

CTC_RS08485	complement(1752628..1753113)	0	146	246	141	377	319	221	162	330	109	595	295	163	211	388	196	208	261	131	100	640	128	538	754	131	299	334	14	152	592
rsmH	complement(1753148..1754080)	130	305	242	188	491	388	150	202	272	158	224	281	170	204	270	212	122	502	146	259	641	120	440	87	106	156	323	37	53	514
mraZ	complement(1754115..1754543)	63	111	124	142	214	362	130	220	125	55	281	445	151	205	293	333	235	177	159	226	279	145	348	95	92	313	216	160	114	313
CTC_RS08500	1754783..1756219	0	0	19	42	16	72	6	11	19	49	28	50	5	10	29	14	18	18	30	34	33	43	0	0	11	8	0	5	0	0
CTC_RS08505	complement(1756290..1757411)	361	296	107	422	449	215	649	407	262	529	287	617	839	183	168	668	315	293	496	216	362	398	233	218	686	329	103	423	339	308
CTC_RS08510	complement(1757440..1758615)	241	262	45	227	97	117	444	201	114	472	123	223	417	187	71	313	43	172	260	206	203	317	159	138	432	95	20	266	83	196
CTC_RS08515	complement(1758620..1759828)	224	78	33	240	133	71	675	143	22	464	93	395	513	36	69	400	104	178	348	80	139	267	154	101	407	139	38	262	61	95
ychF	complement(1760176..1761273)	135	151	339	222	334	267	178	373	462	533	351	1011	171	507	420	434	551	865	492	352	468	158	374	482	173	433	465	545	626	681
CTC_RS08525	complement(1761426..1763714)	0	41	134	7	20	23	0	131	47	5	70	21	0	192	37	0	110	66	2	63	21	0	33	0	0	127	51	0	161	42
CTC_RS08530	1763976..1764383	232	407	261	299	506	676	297	367	458	709	591	468	177	395	360	492	557	372	287	593	1114	335	915	399	136	411	455	522	602	1175
CTC_RS08535	complement(1764611..1767172)	0	19	16	0	18	13	0	25	31	12	69	9	0	51	8	5	20	5	3	0	19	10	29	0	0	44	18	0	10	30
CTC_RS08540	1767333..1768973	25	14	16	19	0	42	11	0	0	110	29	44	4	27	0	12	15	62	13	0	0	0	45	0	10	7	14	29	30	82
CTC_RS08545	1769066..1769260	0	0	0	0	0	0	0	0	0	30	0	0	0	0	0	0	0	0	0	0	0	0	0	0	0	57	0	0	0	0
flgG_1	complement(1769380..1770237)	811	387	387	249	187	542	424	202	218	685	225	376	437	222	196	111	559	280	550	226	335	188	609	664	508	326	135	961	529	156
flgG_2	complement(1770258..1771040)	380	121	238	156	293	462	357	121	171	656	62	396	313	93	54	122	193	259	391	124	306	127	524	780	284	128	30	711	345	367
CTC_RS08560	complement(1771037..1771303)	51	0	0	57	0	65	0	0	0	111	0	0	0	0	0	26	94	47	16	0	0	46	0	305	30	0	0	26	0	0
CTC_RS08565	complement(1771240..1771701)	88	103	201	66	149	224	61	68	0	205	0	103	16	63	45	44	55	27	64	0	155	0	81	0	0	24	0	268	53	83
CTC_RS08570	complement(1771715..1772443)	120	98	73	63	126	142	90	130	0	385	22	65	40	80	0	47	104	122	117	66	263	51	0	112	87	107	95	217	236	79
CTC_RS08575	complement(1772447..1773103)	21	36	81	81	0	26	43	168	0	216	73	0	99	89	64	83	307	58	129	0	109	19	0	62	115	34	0	215	187	88
CTC_RS08580	complement(1773130..1774020)	197	80	142	120	13	348	199	53	0	613	108	134	105	16	94	38	283	85	162	109	188	28	0	69	62	75	0	440	220	215
flhF	complement(1774014..1775147)	131	84	152	104	202	198	123	69	82	388	71	126	95	39	55	54	323	156	112	85	200	44	0	323	119	30	41	321	65	118
flhA	complement(1775149..1777215)	242	46	97	52	44	133	86	69	45	491	101	150	103	32	71	59	146	92	129	23	208	18	108	138	69	32	34	366	190	121
CTC_RS08595	complement(1777246..1779076)	214	52	138	38	50	245	76	34	44	369	149	117	51	48	23	45	138	111	130	159	248	41	143	334	65	12	38	280	94	105
fliQ	complement(1779097..1779366)	50	0	0	113	0	128	104	0	99	142	60	88	160	0	78	50	93	94	79	179	266	0	276	302	59	0	0	153	182	71
fliP	complement(1779379..1780164)	335	60	135	78	117	351	142	20	34	503	102	121	83	0	27	61	385	113	157	0	183	16	47	311	91	43	0	280	94	73
fliO	complement(1780168..1780530)	112	0	73	42	0	475	308	0	0	504	244	132	159	0	58	131	347	140	176	0	132	103	103	336	197	31	128	360	0	53
CTC_RS08615	complement(1780556..1781068)	632	46	181	193	134	454	418	0	104	662	157	279	450	29	123	146	443	247	419	189	280	121	364	317	510	65	0	536	0	187
CTC_RS08620	complement(1781065..1781802)	485	96	378	114	31	561	556	0	109	404	218	420	264	20	85	148	513	292	369	328	357	151	152	165	236	0	0	531	333	130
CTC_RS08625	complement(1781795..1782625)	813	257	416	239	83	705	696	95	32	778	445	287	521	18	50	164	729	305	524	291	547	239	180	588	429	40	84	869	443	161
CTC_RS08630	complement(1782629..1782841)	317	223	375	143	0	243	307	74	0	444	377	336	474	0	0	160	948	357	439	0	112	0	0	382	261	105	0	581	461	0
CTC_RS08635	complement(1783145..1784173)	2180	369	969	393	267	1315	1836	245	363	769	601	951	975	228	489	417	1814	517	971	1034	907	422	363	1385	1345	174	406	1396	1085	373
CTC_RS08640	complement(1784258..1784653)	1092	60	269	116	232	174	966	0	0	209	244	241	328	0	159	189	191	224	118	122	121	0	377	0	290	28	59	260	124	145
CTC_RS08645	complement(1784657..1785181)	541	90	51	44	218	131	711	90	0	191	122	546	254	0	40	78	144	145	251	0	182	47	142	78	211	43	44	92	187	73
CTC_RS08650	complement(1785193..1786458)	416	56	126	102	91	231	486	12	21	187	133	170	234	46	17	81	159	120	212	76	151	39	29	64	157	9	37	198	136	30
fliJ	complement(1786464..1786901)	339	54	243	87	366	433	341	108	0	337	330	1199	107	0	48	171	576	318	204	110	273	57	0	372	91	51	212	275	56	219
fliI	complement(1786924..1788246)	358	90	422	110	35	808	374	36	121	339	219	613	136	44	206	108	572	268	249	274	561	28	85	338	138	59	140	387	278	130
CTC_RS08665	complement(1788279..1789061)	397	61	476	136	88	1101	429	101	307	788	369	1311	129	75	188	139	1643	307	288	309	886	71	95	962	122	128	30	711	345	392
fliG	complement(1789045..1790067)	608	162	793	93	314	1281	547	92	339	889	503	1423	219	43	123	120	777	408	364	520	783	121	255	1035	287	120	113	759	312	422
fliF	complement(1790074..1791639)	298	212	675	39	220	897	399	90	205	355	380	930	101	56	228	70	797	323	262	556	649	24	238	910	96	64	222	250	313	392
fliE	complement(1791656..1791958)	89	0	263	25	76	484	216	0	0	136	106	236	24	0	138	45	333	251	98	160	79	41	123	806	26	111	153	204	81	63
flgC	complement(1791970..1792401)	219	384	739	88	212	1476	821	73	309	96	335	663	167	136	243	126	876	264	320	448	443	86	173	1602	239	129	537	191	170	399
flgB	complement(1792431..1792835)	300	59	427	0	226	979	276	39	660	372	476	88	107	108	311	84	810	188	147	0	118	61	369	1407	59	83	458	153	303	95
CTC_RS08695	complement(1793199..1794029)	1081	257	448	450	124	373	1151	95	289	917	387	460	1133	141	555	250	911	267	460	175	446	463	90	539	1055	202	502	1150	295	669
CTC_RS08700	1794287..1795093	50	59	49	132	57	320	58	156	165	307	60	118	45	73	104	127	0	282	153	120	148	46	46	101	20	0	57	166	46	95
CTC_RS08705	complement(1795134..1795499)	0	65	36	0	63	0	0	86	73	0	439	0	0	320	0	0	207	69	0	0	0	0	102	0	0	153	0	19	0	0
CTC_RS08710	1795631..1796128	0	48	0	31	46	35	0	32	0	12	65	0	29	0	0	14	0	25	26	97	0	0	0	0	32	0	47	14	0	38
CTC_RS08715	complement(1796218..1796907)	0	34	0	44	0	25	14	0	0	51	0	69	0	0	0	99	73	55	18	0	0	0	0	59	23	0	0	30	36	0
CTC_RS08720	complement(1796912..1797793)	31	27	0	52	0	0	11	0	0	67	0	0	25	0	0	23	57	29	43	0	0	56	42	46	9	0	0	78	0	0
CTC_RS08725	complement(1797807..1798430)	0	0	0	0	0	0	0	0	0	28	0	0	0	0	0	11	0	0	14	0	38	0	60	0	0	0	0	0	0	0
CTC_RS08730	complement(1798435..1798800)	0	0	0	21	0	0	0	0	0	48	0	0	0	0	0	0	0	0	12	0	0	0	0	0	0	0	0	19	0	0
CTC_RS08735	complement(1798814..1799518)	48	0	0	0	0	0	0	0	0	0	0	0	0	21	0	10	0	0	12	0	34	18	0	0	0	16	0	0	35	0
CTC_RS08740	complement(1799640..1800155)	3562	873	928	2181	1644	1854	3272	1052	2278	6610	2648	2637	3231	1021	935	2891	636	1644	2554	2062	2341	2813	2242	3432	3212	910	719	1779	1142	1040
CTC_RS08745	complement(1800565..1800900)	80	212	119	136	0	0	0	47	159	202	191	71	0	87	312	111	75	0	190	72	71	37	222	121	47	33	276	82	73	57
CTC_RS08750	complement(1800980..1801168)	0	0	70	81	243	0	0	83	565	31	170	0	153	0	0	108	0	201	45	0	127	0	0	215	0	118	123	36	0	0
CTC_RS08755	complement(1801310..1802116)	1474	382	742	747	568	1378	2069	429	629	1464	697	1420	1288	399	650	882	797	879	727	539	815	861	740	858	1494	291	718	1235	502	784
CTC_RS08760	complement(1802226..1802606)	106	125	209	80	241	679	171	41	210	124	0	63	95	154	0	36	132	100	78	0	188	0	196	214	42	0	0	54	0	0
CTC_RS08765	1802765..1803625	157	0	15	18	27	170	98	0	31	254	19	28	84	17	24	16	0	44	89	0	222	58	43	47	111	39	54	56	0	67
CTC_RS08770	complement(1803881..1804807)	0	0	0	0	0	0	0	0	0	13	52	0	0	16	23	11	0	20	0	0	0	0	0	0	0	0	0	0	26	0
CTC_RS08775	complement(1804930..1805727)	17	30	17	77	57	238	47	39	0	259	141	45	0	55	53	34	158	48	75	121	90	31	0	0	30	56	29	69	62	96
CTC_RS08780	complement(1805941..1806423)	0	0	0	0	0	36	19	0	55	73	0	247	0	30	43	0	0	52	44	0	50	0	77	0	16	0	48	114	51	79
CTC_RS08785	complement(1806498..1807736)	22	38	43	6	93	56	60	0	22	186	52	116	17	59	34	11	41	20	48	39	135	30	0	0	0	0	37	17	20	0
CTC_RS08790	complement(1807851..1808078)	59	0	0	0	0	76	0	0	0	0	0	0	0	0	0	0	0	0	0	0	0	0	0	0	0	0	0	0	0	0
CTC_RS08795	complement(1808156..1808395)	0	0	0	0	0	0	0	0	0	0	0	0	0	0	0	0	0	0	0	0	0	0	0	0	0	0	0	0	0	0
aepX	complement(1808817..1810121)	953	109	122	123	88	317	861	145	72	0	0	0	523	56	80	115	271	233	0	0	0	190	172	218	352	111	71	392	150	147
aepY	complement(1810192..1811322)	585	31	82	101	122	358	495	97	118	0	0	0	239	78	56	148	257	313	0	0	0	66	66	144	242	44	61	313	228	186
CTC_RS08810	complement(1811406..1812533)	0	0	0	0	0	0	0	0	0	0	0	0	0	0	0	0	0	0	0	0	0	0	0	0	0	0	0	0	0	0
CTC_RS08815	complement(1812559..1814220)	0	0	0	0	0	0	0	0	0	0	0	0	0	0	0	0	0	0	0	0	0	0	0	0	0	0	0	0	0	0
CTC_RS08820	complement(1814248..1815228)	0	0	0	0	0	0	0	0	0	0	0	0	0	0	0	0	0	0	0	0	0	0	0	0	0	0	0	0	0	0
CTC_RS08825	complement(1815250..1816272)	0	0	0	0	0	0	0	0	0	0	0	0	0	0	0	0	0	0	0	0	0	0	0	0	0	0	0	0	0	0
CTC_RS08830	complement(1816285..1817226)	0	0	0	0	0	0	0	0	0	0	0	0	0	0	0	0	0	0	0	0	0	0	0	0	0	0	0	0	0	0
CTC_RS08835	complement(1817379..1819424)	59	12	19	30	56	34	75	8	0	0	0	0	37	7	21	20	25	56	0	0	0	15	0	99	19	22	11	141	12	28
CTC_RS08840	complement(1819448..1820596)	0	0	0	0	0	0	0	0	0	0	0	0	0	0	0	3	0	0	0	0	0	0	0	0	0	0	0	0	0	0
CTC_RS08845	complement(1820660..1821709)	0	0	0	0	0	0	0	0	0	0	0	0	0	0	0	0	0	0	0	0	0	6	0	0	0	0	0	0	0	0
CTC_RS08850	complement(1821699..1822679)	0	0	0	0	0	0	0	0	0	0	0	0	0	0	0	0	0	0	0	0	0	0	0	0	0	0	0	0	0	0
CTC_RS08855	complement(1822712..1823695)	0	0	0	0	0	0	0	0	0	0	0	0	0	0	0	0	0	0	0	0	0	0	0	0	0	0	0	0	0	0
CTC_RS08860	complement(1823748..1824491)	0	0	0	0	0	0	0	0	0	0	0	0	0	0	0	0	0	0	0	0	0	0	0	0	0	0	0	0	0	0
CTC_RS08865	complement(1824551..1825606)	0	0	0	0	0	0	0	0	0	0	0	0	0	0	0	0	0	0	0	0	0	0	0	0	0	0	0	0	0	0
pseB	complement(1825628..1826632)	155	24	86	76	68	180	246	31	0	288	80	95	83	0	42	54	163	145	231	48	71	25	37	223	83	0	23	284	134	162
CTC_RS08875	complement(1826648..1826977)	41	0	81	0	69	52	28	0	0	161	0	72	0	44	0	62	76	38	64	0	0	0	226	0	48	0	0	208	149	0
CTC_RS08880	complement(1826977..1828779)	75	0	37	13	0	100	52	0	0	161	9	73	24	0	0	25	42	53	25	0	40	7	21	113	11	0	13	112	14	16

CTC_RS08885	complement(1828967..1829794)	83058	10199	12367	22133	13551	20620	109317	12015	15081	35048	13414	24834	70987	7063	14376	27532	18769	14832	17654	15716	20484	35433	18571	40932	83977	9186	16562	35092	21891	11054
CTC_RS08890	complement(1830297..1830629)	365	214	200	69	138	362	448	95	80	417	603	717	217	88	441	61	455	418	140	726	431	37	0	367	214	67	278	206	74	58
fliD	complement(1830649..1832319)	1387	554	481	384	412	1093	1851	358	416	486	539	878	1073	228	446	497	1585	909	244	275	465	427	581	658	1025	167	361	699	639	740
fliS	complement(1832342..1832728)	978	184	326	99	178	534	1085	122	414	351	581	493	634	227	379	299	2673	720	104	625	371	321	193	736	963	202	240	586	698	693
CTC_RS08905	complement(1832743..1833027)	285	83	140	161	0	242	458	166	0	124	282	167	380	51	221	96	531	267	90	170	0	87	0	143	362	0	0	145	86	269
CTC_RS14565	complement(1833046..1833426)	337	62	297	150	180	204	343	124	280	140	0	219	303	115	193	179	596	299	61	0	94	65	0	748	364	44	61	198	258	352
csrA	complement(1833454..1833666)	1015	223	562	322	538	566	1051	222	188	638	755	224	644	275	492	320	474	654	240	454	786	175	175	191	670	367	109	387	807	180
fliW	complement(1833660..1834097)	309	0	91	87	52	354	362	18	61	229	239	272	264	67	96	171	518	289	83	110	109	113	0	0	253	0	106	298	56	88
flgL	complement(1834419..1835369)	1208	549	531	353	494	852	1256	463	393	938	532	1569	1021	246	397	423	1592	706	282	331	667	418	314	1156	1293	212	463	437	671	625
flgK	complement(1835382..1837121)	458	150	176	180	211	376	515	145	261	540	240	768	483	126	145	243	1131	386	182	348	488	250	322	398	506	96	180	379	430	330
CTC_RS08935	complement(1837154..1837561)	232	233	196	84	225	507	663	116	262	1006	827	878	345	144	103	200	618	714	83	356	967	91	0	798	496	0	114	489	180	376
flgM	complement(1837580..1837864)	806	333	233	107	161	181	556	166	94	269	846	837	431	51	368	287	1151	444	194	0	168	174	65	429	459	118	325	531	431	605
fliY	complement(1838003..1839241)	2498	747	1406	1420	1462	2775	1781	839	733	2553	1284	2312	1357	756	660	1091	1792	1303	1172	1230	1487	1132	1566	2497	1491	474	636	2355	1307	1253
fliM	complement(1839234..1840232)	1204	380	725	581	895	1208	995	378	294	1927	627	860	925	293	336	600	1136	970	700	581	910	559	785	1039	794	291	209	1387	590	595
CTC_RS08955	complement(1840249..1840653)	367	351	328	188	57	426	484	194	66	510	119	236	392	72	52	370	436	406	304	239	236	123	92	402	313	359	57	373	424	284
CTC_RS08960	complement(1840723..1841082)	1351	593	1219	1261	1273	1532	1166	962	668	1427	848	1923	1063	366	466	672	1542	914	856	1881	864	724	1866	1866	947	404	515	1824	1329	852
CTC_RS08965	complement(1841098..1841697)	1284	633	776	610	764	1939	739	315	312	1767	723	1392	644	195	454	494	757	897	415	1129	877	517	1057	1425	674	373	309	1169	245	990
CTC_RS08970	complement(1841710..1844106)	851	643	591	403	851	1323	932	427	345	1202	590	1399	611	333	411	389	963	671	600	727	1163	404	685	1325	582	303	343	1070	707	448
CTC_RS08975	complement(1844119..1844886)	70	247	147	30	209	337	109	61	104	361	105	249	56	0	55	53	99	82	78	126	436	65	146	636	10	15	121	125	96	50
CTC_RS08980	complement(1844867..1845939)	447	354	428	217	513	851	339	176	174	451	434	667	188	177	254	298	1129	566	337	316	357	150	591	759	251	167	130	423	526	625
CTC_RS08985	complement(1845954..1846442)	442	437	381	328	328	388	382	258	164	423	690	732	369	359	257	418	980	570	452	989	587	330	496	999	373	251	284	492	753	235
CTC_RS08990	complement(1846472..1846930)	501	258	319	216	400	300	264	171	349	476	350	364	299	255	91	334	934	662	273	422	261	378	407	266	354	244	202	562	401	543
CTC_RS08995	complement(1846942..1848522)	252	255	286	188	377	534	148	179	135	370	681	770	201	176	371	215	846	320	210	275	454	126	354	502	150	233	396	291	295	218
CTC_RS09000	complement(1848647..1848937)	46	245	0	52	79	0	32	0	0	71	110	164	99	0	72	47	260	0	29	166	0	0	0	140	27	77	159	71	253	66
CTC_RS09005	complement(1848967..1849788)	82	29	32	46	195	105	68	38	65	144	78	174	61	107	51	33	184	62	67	177	58	60	0	149	48	54	113	109	60	70
CTC_RS09010	complement(1850050..1850703)	41	73	20	93	35	26	29	0	0	9	61	0	0	22	192	73	39	39	52	0	0	76	57	62	0	17	35	11	0	29
CTC_RS09015	complement(1850778..1851119)	1225	1110	894	1573	1273	1058	1718	2209	2421	449	2867	1605	1667	3039	3189	1334	1770	1370	1523	1697	1189	1361	1310	1667	1785	2843	2000	935	861	1401
nifJ_2	complement(1851264..1854785)	7845	2845	1474	3075	3872	2310	4001	2418	1297	6014	1814	4063	2785	1694	959	3379	2084	2311	3390	3118	2965	3620	4138	1809	3160	1739	1014	5045	2000	2125
CTC_RS09025	complement(1855196..1855621)	793	2394	1811	1612	2744	1457	1336	3289	3354	749	2867	4818	1576	3436	3397	1599	4264	3180	1188	2498	2976	1485	3548	3823	1284	4014	3211	758	3169	1935
CTC_RS09030	complement(1855693..1856769)	38	66	49	57	0	48	26	44	25	55	45	66	34	177	136	47	23	41	47	0	44	17	35	76	22	93	65	32	46	18
CTC_RS09035	1856917..1857117	0	0	198	0	0	429	0	235	930	0	320	0	0	218	1044	0	126	504	0	0	119	0	0	405	0	111	923	0	244	191
recJ	complement(1857165..1858931)	0	13	23	26	13	39	11	62	30	43	18	27	25	33	36	58	14	21	26	0	14	7	63	0	13	25	26	12	0	43
CTC_RS09045	1859302..1860120	182	203	244	214	420	863	103	96	228	710	285	321	106	214	205	137	154	533	197	354	876	136	365	1193	107	150	142	239	165	351
CTC_RS09050	complement(1860244..1860858)	0	39	76	0	37	0	0	51	0	0	78	39	0	24	34	0	0	0	7	0	0	20	0	132	19	0	0	0	0	0
thiH	complement(1860837..1861923)	25	153	49	21	169	63	34	14	25	0	118	110	7	13	19	6	116	23	4	0	22	11	103	112	15	21	43	0	45	71
CTC_RS09060	complement(1861943..1862710)	0	93	69	0	60	0	0	61	35	0	84	62	19	38	27	18	66	82	0	252	93	0	49	53	31	73	30	0	96	150
thiF	complement(1862722..1863525)	17	59	17	19	200	43	0	39	33	0	80	0	0	0	78	0	31	31	0	60	30	0	46	51	0	42	0	0	31	95
thiS	complement(1863527..1863721)	0	0	0	0	0	0	0	0	0	0	0	0	0	0	0	0	0	0	0	0	0	0	0	0	0	57	0	0	0	0
thiE	complement(1863853..1864482)	0	38	21	12	36	27	0	0	0	0	128	76	0	46	0	0	80	20	0	77	76	20	59	129	13	18	0	0	0	61
thiM	complement(1864475..1865299)	0	0	0	18	0	63	0	57	0	21	97	29	35	35	25	33	122	92	21	0	0	15	0	0	0	41	0	0	89	46
thiD	complement(1865320..1866192)	0	82	61	26	26	0	0	36	31	7	92	55	17	34	48	31	0	44	10	166	27	14	86	47	18	51	27	24	84	22
thiW	complement(1866195..1866695)	0	47	27	0	0	138	0	31	53	24	64	95	14	29	84	14	151	25	0	0	0	0	74	0	0	22	46	14	49	77
CTC_RS09095	complement(1866948..1868171)	22	19	11	25	94	28	23	26	22	63	0	98	12	24	69	33	186	155	38	0	20	10	30	33	0	18	19	17	20	78
CTC_RS09100	complement(1868187..1868735)	25	130	0	14	84	31	17	86	49	54	0	0	53	0	38	87	46	46	70	88	0	23	0	0	14	41	84	50	134	140
CTC_RS09105	1869141..1869575	62	164	428	158	316	396	193	434	553	326	517	274	83	303	96	376	986	349	313	111	770	114	515	936	109	283	373	190	395	573
glpK_1	complement(1869621..1871117)	4920	1870	728	11337	3016	484	3403	1193	607	809	601	638	7391	724	546	6772	455	203	3811	2682	1055	9507	2967	952	6807	818	650	1511	7386	1357
CTC_RS09115	complement(1871307..1871879)	637	911	1392	706	780	2196	488	659	1212	216	1907	3082	605	1099	1318	226	1233	784	267	1351	1294	866	716	710	609	936	971	108	685	769
CTC_RS09120	complement(1871890..1872279)	450	730	1381	714	647	972	1017	807	959	288	3379	2815	805	713	1775	349	2070	601	425	1861	1625	716	574	313	1220	1060	1605	123	566	836
dhaL	complement(1872308..1872943)	319	597	721	522	721	298	609	718	630	121	2009	2008	579	978	1022	439	1547	458	435	1369	1016	449	411	320	835	703	365	108	810	527
dhaK	complement(1872960..1873958)	406	1140	852	588	837	449	1111	1221	1364	89	3017	2079	1116	1480	1722	610	1856	735	536	1598	1030	708	785	448	1199	862	1694	124	958	633
rocF	1874307..1875203	121	26	59	34	51	19	31	53	30	178	36	0	8	33	0	38	84	56	95	0	53	0	83	91	0	62	0	130	27	43
CTC_RS09140	complement(1875291..1876328)	26	23	26	0	44	17	0	15	0	46	0	92	28	0	20	26	0	49	37	140	69	12	0	0	8	22	22	53	71	55
CTC_RS09145	complement(1876451..1879330)	9	8	9	19	0	12	13	11	9	27	22	25	3	0	0	17	26	22	22	50	17	22	0	0	3	8	8	14	26	0
CTC_RS09150	complement(1879343..1881298)	0	0	14	23	0	0	12	8	0	30	8	73	4	0	0	28	52	45	24	25	12	19	19	21	0	0	12	39	0	49
CTC_RS09155	complement(1881470..1882117)	42	73	21	82	0	80	29	0	0	182	74	111	45	45	32	11	39	78	20	0	37	38	58	126	12	17	0	0	0	30
CTC_RS09160	complement(1882238..1883134)	0	53	30	26	0	38	21	53	30	0	18	27	16	33	47	23	56	56	33	54	0	41	62	0	0	37	52	0	0	0
ptsP	complement(1883395..1885110)	32	152	105	93	160	161	38	128	125	98	450	195	46	145	159	79	118	155	50	141	153	29	43	261	51	130	95	40	86	156
CTC_RS09170	complement(1885269..1885988)	188	395	573	238	987	1149	130	525	334	525	826	779	100	346	408	128	140	264	177	605	465	103	674	1018	66	388	676	124	273	439
CTC_RS09175	1886105..1887451	0	18	20	0	17	26	14	12	0	4	12	35	0	22	0	0	19	0	0	0	0	0	0	30	0	17	0	0	0	0
CTC_RS09180	complement(1887630..1887854)	60	211	355	68	306	0	0	140	475	184	357	530	96	65	186	15	0	0	19	215	532	0	166	181	176	248	103	31	327	85
CTC_RS09185	complement(1887860..1888996)	48	63	70	20	121	106	12	42	23	140	184	84	0	39	0	12	55	134	22	43	84	11	98	143	7	20	20	48	86	169
CTC_RS09190	1889232..1890719	0	32	40	0	15	0	0	53	18	4	86	0	0	10	14	5	0	26	0	0	0	0	25	0	0	23	47	5	16	0
CTC_RS09195	1890737..1891918	0	0	0	0	0	15	0	0	0	0	27	0	0	12	0	6	0	0	0	0	0	0	0	0	0	9	0	0	21	0
CTC_RS09200	1891939..1892115	0	0	0	0	0	0	0	0	0	0	0	0	0	0	0	0	0	0	0	0	0	0	0	0	0	0	0	0	0	0
CTC_RS09205	1892127..1893212	12	0	0	0	0	0	0	0	0	0	0	0	0	0	0	0	0	0	0	0	22	0	0	0	4	0	0	0	0	0
CTC_RS09210	complement(1893258..1893710)	0	628	205	17	253	152	21	243	0	13	497	158	16	1196	324	30	2005	363	9	0	53	0	494	180	105	1012	51	15	2059	719
hydA	complement(1893713..1895098)	0	667	163	0	265	50	0	397	96	0	707	52	0	988	212	5	1929	311	6	70	17	0	364	0	0	1077	234	10	1541	498
CTC_RS14570	complement(1895195..1895926)	37	65	36	31	31	118	51	22	36	81	154	130	20	0	29	65	69	156	99	66	196	17	153	0	43	15	0	75	168	26
CTC_RS09225	complement(1895977..1897221)	0	57	43	18	0	83	30	13	0	47	39	19	0	0	17	11	0	41	31	0	38	10	0	33	0	0	74	33	39	46
CTC_RS09230	complement(1897354..1898739)	49	17	10	77	17	37	114	79	39	85	81	17	146	42	0	162	146	201	3	35	17	63	81	0	126	32	0	15	53	28
CTC_RS09235	complement(1898736..1899299)	24	0	24	41	0	31	149	0	47	52	114	0	115	0	37	85	179	157	0	0	0	66	0	0	169	40	0	30	0	170
CTC_RS09240	complement(1899442..1900875)	19	0	28	16	48	36	52	22	19	12	34	67	20	0	73	0	0	0	3	0	0	17	0	28	6	39	65	0	0	0
CTC_RS09245	complement(1900875..1901441)	0	0	0	0	0	0	0	0	0	0	28	0	0	0	0	0	0	22	0	0	0	0	0	0	42	20	0	0	0	0
CTC_RS09250	complement(1901595..1902968)	0	0	19	0	17	0	0	11	0	4	12	17	0	11	0	10	0	83	0	0	0	0	0	0	0	8	34	0	36	42
CTC_RS09255	complement(1902955..1903482)	26	0	0	0	0	0	0	0	0	0	0	0	0	0	0	0	96	72	0	0	0	0	0	77	0	0	0	0	0	0
CTC_RS09260	complement(1903616..1904086)	115	50	28	65	49	37	59	33	0	88	137	101	0	31	89	202	295	188	208	205	102	0	0	0	67	47	98	131	52	41
CTC_RS09265	complement(1904218..1905048)	16	86	48	9	55	0	0	38	0	192	19	0	0	106	50	49	0	15	5	116	0	15	0	98	10	81	56	25	59	46
CTC_RS09270	complement(1905064..1906278)	0	117	55	6	57	14	0	26	22	287	93	98	24	96	35	123	42	10	7	80	39	31	123	0	26	83	38	62	101	63
CTC_RS09275	complement(1906478..1908265)	0	0	0	17	0	19	0	26	15	3	18	13	8	25	12	11	14	14	5	54	0	0	0	0	9	53	0	0	21	0
CTC_RS09280	1908382..1909317	0	25	14	0	0	0	0	0	0	0	17	0	0	0	0	0	54	0	0	0	26	0	0	0	0	12	0	0	0	41

CTC_RS09285	1909491..1910801	10	0	0	26	17	39	0	0	0	50	25	36	11	11	0	0	0	29	6	0	0	9	0	0	18	43	18	47	37	29
CTC_RS09290	complement(1910839..1912086)	0	19	21	12	0	41	22	38	0	47	84	38	12	12	17	11	40	51	37	0	19	0	0	0	6	0	0	28	20	46
CTC_RS09295	complement(1912129..1912860)	0	0	0	0	31	0	0	0	0	8	0	0	0	0	0	0	0	0	0	0	0	0	0	0	0	0	0	0	34	0
CTC_RS09300	complement(1912853..1913767)	0	0	0	8	0	0	0	0	0	13	0	0	0	0	0	7	28	0	0	0	0	0	0	0	0	0	0	15	0	42
CTC_RS09305	complement(1913879..1914790)	44	156	0	42	226	94	20	86	176	45	229	157	24	64	46	67	55	69	37	0	26	14	143	0	35	80	25	60	54	63
CTC_RS09310	complement(1914787..1915494)	38	67	75	32	130	97	13	78	0	8	91	101	61	83	0	19	36	107	36	0	34	0	53	0	0	111	65	29	35	27
CTC_RS09315	complement(1915540..1917906)	51	100	124	42	174	73	35	160	56	37	143	182	98	158	106	43	117	193	69	327	131	63	126	69	64	142	88	52	114	97
CTC_RS09320	complement(1917896..1918576)	20	104	0	22	34	76	0	162	0	43	118	35	106	150	62	20	74	112	94	142	70	73	0	60	23	98	170	50	0	113
CTC_RS09325	complement(1918598..1920040)	9	197	157	95	238	131	58	251	111	37	412	240	85	228	160	109	210	224	121	101	133	69	78	28	88	213	145	57	136	173
CTC_RS09330	complement(1920215..1921351)	24	188	58	81	81	167	25	173	47	65	85	126	38	64	37	0	0	0	71	64	105	33	33	36	35	93	143	0	0	0
CTC_RS09335	1921775..1922005	0	308	230	0	99	75	0	273	694	0	765	207	0	824	272	0	546	110	0	209	0	0	162	0	0	484	100	0	106	83
CTC_RS09340	1922021..1922380	0	1120	1847	21	573	670	0	2143	4563	0	2188	995	0	2094	1340	0	1822	1442	12	672	332	0	622	565	0	1646	1546	0	1909	1118
CTC_RS09345	complement(1922425..1924164)	39	14	46	31	40	50	32	9	15	75	9	27	29	8	12	43	29	29	22	0	14	14	43	23	14	45	27	71	14	22
hutG	1924296..1925312	120	327	458	150	113	237	64	310	184	209	221	211	121	216	124	107	198	448	121	95	141	98	147	40	78	154	228	95	265	170
CTC_RS09355	1925665..1926663	108	214	512	298	367	768	140	95	374	71	306	334	275	249	168	157	278	507	70	48	766	211	486	1263	175	168	325	89	86	787
CTC_RS09360	complement(1926724..1927374)	0	802	1277	23	35	79	0	919	780	27	716	623	0	1878	1224	21	1434	350	7	0	55	0	573	63	0	1975	1496	0	1206	589
yfcE	1927512..1928057	0	0	0	0	42	63	17	0	0	32	0	0	13	0	0	12	0	0	31	89	44	45	0	0	0	20	42	25	0	105
CTC_RS09370	complement(1928142..1929476)	0	0	0	0	0	39	0	0	0	0	0	0	5	0	0	0	0	0	3	0	0	0	0	0	6	0	0	0	0	0
CTC_RS09375	complement(1929460..1930041)	0	0	0	0	0	0	0	0	0	0	0	82	0	0	36	0	0	0	0	0	0	0	0	0	0	0	0	0	42	0
CTC_RS14735	complement(1930191..1930439)	0	0	0	61	0	0	37	0	0	24	0	0	0	59	0	96	355	254	0	0	96	0	0	163	32	45	0	41	148	308
CTC_RS09380	complement(1930457..1931701)	22	0	43	18	0	28	11	0	0	24	0	0	6	0	17	5	20	41	14	0	0	0	0	98	13	27	0	22	39	15
CTC_RS09385	complement(1931708..1932403)	0	0	38	0	0	0	13	23	38	0	0	0	0	42	0	20	0	18	0	0	0	18	54	0	0	0	33	0	0	0
CTC_RS09390	complement(1932489..1935026)	0	9	16	6	0	20	4	25	0	9	25	9	6	23	17	3	20	5	0	0	9	10	0	0	9	18	18	8	0	30
CTC_RS09395	complement(1935040..1935720)	0	35	39	11	0	51	0	0	39	0	0	70	0	21	0	0	37	0	0	71	70	0	0	0	0	0	34	10	0	0
CTC_RS09400	complement(1935906..1936835)	0	0	0	8	25	0	0	17	57	25	17	0	0	16	0	0	0	0	0	0	0	0	0	0	0	0	0	0	0	0
CTC_RS09405	complement(1936908..1937501)	0	0	0	26	77	0	31	26	0	60	0	40	24	0	0	34	0	21	29	0	0	0	0	0	0	38	0	35	0	32
CTC_RS09410	1937667..1938368	0	0	0	0	0	0	0	0	0	0	23	0	0	21	0	0	0	0	0	0	0	0	0	0	0	32	0	0	0	27
CTC_RS09415	1938591..1938944	0	0	38	0	0	0	0	0	0	33	0	0	0	0	0	0	0	0	24	0	0	0	0	0	0	0	0	19	0	54
CTC_RS09420	1938941..1939630	0	0	19	0	0	0	0	0	0	43	0	35	0	0	0	0	0	0	6	0	0	0	0	0	0	0	0	30	36	0
CTC_RS09425	complement(1939714..1941441)	352	288	231	199	318	219	146	228	185	92	84	332	249	51	121	327	58	139	182	84	222	280	108	118	216	136	181	123	170	122
CTC_RS09430	complement(1941603..1942697)	0	22	0	7	21	16	9	29	0	5	0	0	7	13	38	12	69	12	4	0	0	11	0	0	0	31	0	0	45	35
CTC_RS09435	complement(1942768..1943877)	1071	2180	1683	1763	1755	1157	1038	1914	1901	830	2071	2795	1778	2308	2589	1503	1296	1072	1526	2658	1530	2163	1614	1981	1361	2447	2298	709	1747	1658
CTC_RS09440	complement(1944088..1944723)	0	37	42	0	0	27	0	0	0	0	51	75	23	23	0	21	0	100	20	0	75	0	0	0	0	0	0	11	39	0
CTC_RS09445	complement(1944883..1946643)	0	27	8	0	13	10	0	36	0	7	73	14	0	75	36	0	143	22	2	0	14	0	42	23	9	13	53	39	98	109
CTC_RS09450	complement(1946790..1948949)	19	22	31	35	21	32	17	7	12	55	37	66	3	20	29	11	12	59	20	22	55	0	17	38	0	10	43	48	0	18
CTC_RS14310	complement(1949020..1950354)	0	0	0	0	0	0	0	0	0	9	0	0	0	0	0	0	0	0	3	0	0	0	0	30	0	0	0	10	0	0
CTC_RS14315	complement(1950420..1952483)	13	0	13	4	0	17	9	8	0	46	39	12	10	7	20	3	24	25	12	0	0	6	0	0	0	5	11	40	0	9
CTC_RS09465	complement(1952703..1953686)	0	24	0	0	23	18	0	0	0	6	33	24	0	15	0	28	26	0	13	0	24	0	0	41	0	0	0	49	0	19
CTC_RS09470	complement(1953705..1954739)	0	46	39	0	0	0	0	0	0	6	16	69	0	0	20	0	0	0	4	0	0	0	0	39	0	0	22	33	0	37
CTC_RS09475	complement(1954847..1955674)	0	0	0	9	28	0	0	38	0	0	19	29	0	18	0	0	0	0	0	0	0	0	0	0	10	13	0	0	30	35
CTC_RS09480	complement(1955756..1956943)	0	20	56	0	19	29	8	26	0	0	27	80	0	62	0	0	0	11	0	0	0	0	0	0	0	38	59	17	41	16
CTC_RS09485	1957114..1958151	13	0	38	22	0	0	27	45	26	125	15	46	28	14	0	33	0	0	29	0	69	24	36	78	53	0	22	20	24	0
CTC_RS09490	complement(1958204..1958938)	0	0	0	0	62	70	13	43	36	48	0	0	0	0	29	19	34	34	6	0	0	0	0	0	0	15	0	19	33	26
CTC_RS09495	1959136..1959399	102	90	101	29	0	0	0	0	101	0	0	90	0	0	79	77	96	0	0	0	0	47	0	0	30	0	0	52	93	290
CTC_RS09500	complement(1959441..1961207)	76	13	15	17	26	0	26	0	0	100	0	14	29	0	0	42	29	14	17	27	14	28	0	0	45	19	13	270	56	22
CTC_RS14320	1961528..1963138	0	0	8	5	14	32	17	10	0	26	10	0	0	5	13	0	0	0	11	0	15	15	0	0	5	14	14	4	0	0
CTC_RS09510	1963126..1963905	0	0	17	20	88	0	0	0	0	30	21	0	9	0	0	9	32	49	11	0	0	16	0	0	0	14	30	9	0	25
CTC_RS09515	complement(1963926..1967174)	4	7	16	12	56	32	11	10	8	25	30	37	4	32	0	10	16	43	14	15	0	0	0	0	2	28	7	2	15	35
truA_1	1967301..1968038	0	0	0	0	62	47	0	64	72	80	0	0	0	20	0	28	17	34	17	0	32	0	51	221	21	0	0	93	67	182
CTC_RS09525	complement(1968108..1968521)	33	0	128	147	111	125	0	38	194	128	78	115	52	71	152	99	61	107	185	0	58	60	0	0	57	108	56	399	0	93
CTC_RS09530	complement(1968611..1970050)	28	33	28	40	80	60	84	22	74	66	67	17	35	30	29	71	70	70	94	101	166	26	143	141	39	39	16	468	605	200
CTC_RS14875	complement(1970282..1970434)	0	0	0	0	0	0	0	0	0	0	0	0	0	0	0	0	0	0	0	0	0	0	0	0	0	0	0	0	0	0
CTC_RS09535	1970578..1971135	0	85	0	0	0	0	0	56	0	32	0	0	0	0	38	0	0	0	0	0	0	0	0	0	0	0	0	37	0	34
CTC_RS09540	1971377..1972963	9	75	50	19	116	33	12	89	84	22	101	30	0	46	53	4	16	8	3	0	0	0	141	77	5	63	205	9	46	48
CTC_RS09545	1973130..1974290	0	20	34	13	20	0	8	27	0	10	14	62	6	0	0	23	11	33	29	0	41	11	32	105	14	10	20	24	0	66
CTC_RS09550	complement(1974357..1976366)	0	12	26	19	0	17	5	0	13	47	24	47	4	22	0	10	25	13	6	24	24	12	0	20	6	17	35	44	0	10
CTC_RS09555	1976575..1977009	62	382	1437	105	422	1902	43	326	553	149	480	1262	66	202	338	125	812	1281	98	667	2145	57	1115	2246	18	283	320	95	677	925
CTC_RS09560	1977056..1977469	0	0	289	18	55	333	23	0	65	86	78	173	0	0	51	16	0	122	31	117	404	0	90	197	0	0	0	33	59	93
bioD	complement(1977603..1978286)	0	0	0	0	0	25	0	0	0	9	23	0	0	21	0	0	0	0	31	71	0	0	0	0	0	0	34	0	0	0
CTC_RS09570	complement(1978283..1978849)	0	0	0	0	121	0	0	28	0	10	0	0	13	0	0	12	0	0	15	0	0	0	0	0	0	0	0	0	0	0
CTC_RS09575	complement(1979029..1979649)	87	229	86	258	332	250	150	355	215	380	336	307	70	236	338	439	223	510	322	467	539	360	120	328	89	324	560	343	257	247
zwf	complement(1979649..1981088)	122	132	74	228	287	168	155	339	148	461	391	133	65	183	248	336	333	501	353	403	781	121	156	141	72	179	177	275	153	319
gndA	complement(1981078..1982499)	181	200	94	298	242	145	276	260	301	359	226	84	310	278	339	730	310	663	508	408	673	244	315	344	184	314	342	353	250	445
CTC_RS09590	1982704..1984149	336	1050	1573	792	1728	1389	497	1165	2013	605	1378	1403	404	942	747	551	419	762	244	1522	3061	622	1342	760	307	796	1106	219	441	954
brnQ_2	complement(1984250..1985587)	40	18	10	46	17	0	14	12	40	124	24	0	43	22	31	76	0	38	54	0	0	65	28	0	12	0	0	62	18	14
CTC_RS09600	complement(1986060..1989029)	137	519	508	848	644	763	144	771	477	1456	127	249	78	453	438	1054	123	671	626	130	209	340	515	672	124	681	476	691	566	1481
CTC_RS09605	complement(1989054..1989851)	389	1323	983	1109	1307	1112	433	1085	736	1798	463	538	217	807	815	1131	285	1864	709	242	929	762	1076	1709	278	665	784	646	677	3567
CTC_RS09610	complement(1989867..1991189)	260	1533	1025	955	1057	1713	578	1321	1232	1275	541	830	336	1046	1015	1349	362	1885	704	512	1609	656	1326	1631	342	988	1086	668	983	3905
CTC_RS09615	complement(1991410..1992165)	0	0	26	20	61	46	0	83	71	31	64	32	10	97	83	0	33	33	8	0	0	0	49	0	10	103	92	9	32	25
ric	complement(1992192..1992890)	0	0	0	0	33	0	0	68	0	8	0	0	0	21	30	10	36	0	0	0	0	0	0	0	11	0	33	10	0	0
CTC_RS09625	complement(1992909..1994141)	0	0	0	6	37	0	0	0	0	0	26	39	0	12	0	0	20	31	3	0	0	0	0	0	0	0	0	0	0	16
CTC_RS09630	complement(1994384..1995094)	0	67	37	32	161	48	39	44	38	224	68	67	61	41	30	105	71	249	78	0	34	17	52	115	100	16	33	68	104	297
CTC_RS09635	complement(1995155..1996177)	13	116	65	142	134	202	18	31	26	237	79	93	113	72	103	113	271	111	83	47	23	158	73	40	62	131	23	101	144	225
CTC_RS09645	complement(1996450..1997553)	0	0	0	0	0	0	0	0	0	0	0	0	0	0	0	31	69	80	0	0	0	0	0	0	0	0	0	34	322	182
CTC_RS09650	1997963..1999030	0	244	461	14	386	210	0	177	950	11	196	190	14	233	314	6	0	77	12	226	358	0	105	114	0	147	174	0	46	179
CTC_RS09655	complement(1999106..2000815)	24	21	62	22	40	40	82	28	31	59	122	84	21	68	25	16	30	67	50	85	14	44	44	262	14	39	81	80	43	67
CTC_RS09660	complement(2000949..2001317)	0	0	72	0	0	140	0	0	0	64	44	0	0	0	0	0	0	0	0	0	0	0	0	0	0	0	0	19	0	0
codA	complement(2001581..2002843)	32	0	21	12	0	0	44	75	85	9	153	0	23	93	0	38	80	10	10	0	66	20	0	0	50	186	92	5	466	46
CTC_RS09670	2003107..2003862	36	126	18	30	0	0	111	62	35	39	149	63	38	39	28	27	100	0	22	64	47	0	197	0	42	118	31	18	487	76
CTC_RS09675	2003891..2004790	15	79	0	8	51	96	104	52	0	13	54	106	8	57	0	15	84	113	19	107	66	14	83	45	35	50	26	0	436	64

CTC_RS09680	complement(2004829..2006034)	0	0	33	32	0	14	0	0	0	83	0	0	18	12	0	0	0	10	25	0	60	0	31	34	7	19	19	11	0	8
CTC_RS09685	complement(2006098..2007078)	0	0	14	8	0	18	10	0	0	18	0	0	7	30	0	7	0	26	4	0	24	13	0	0	0	0	0	7	0	0
tly	complement(2007254..2008837)	3349	1228	2926	1566	2373	5381	3566	566	1265	3913	8371	4279	1249	379	914	959	4276	1135	1533	2657	3542	2150	3075	9342	1232	321	966	3513	3238	1246
CTC_RS09695	2009114..2010292	0	20	0	6	0	0	0	0	0	0	14	0	6	50	0	0	0	21	0	0	0	0	0	0	7	0	0	0	0	0
CTC_RS09700	complement(2010371..2010826)	0	0	0	0	0	0	0	0	0	13	0	0	0	32	0	0	0	0	9	0	0	0	0	0	0	0	0	0	0	0
CTC_RS09705	complement(2010853..2011491)	0	0	0	0	0	0	0	0	0	28	0	0	0	0	0	0	0	0	0	0	0	19	0	0	0	17	0	0	0	0
CTC_RS09710	complement(2011488..2012348)	0	0	15	0	0	20	0	0	0	0	0	0	0	0	0	0	0	15	0	0	0	0	0	0	0	0	0	0	0	0
CTC_RS09715	complement(2012351..2012728)	0	0	35	20	0	0	0	0	0	0	0	0	19	0	0	0	0	0	0	0	0	0	0	0	0	0	0	18	0	0
CTC_RS14575	complement(2012875..2013033)	0	0	0	0	0	0	0	0	0	0	0	0	0	0	0	43	0	0	0	0	0	0	0	0	0	0	0	0	154	0
CTC_RS09720	2013140..2013451	0	76	0	24	0	0	60	0	86	19	52	77	116	0	0	22	0	0	0	0	0	0	0	0	51	0	149	0	0	0
arsD	2013479..2013841	0	65	37	21	63	95	0	43	0	0	0	197	20	40	58	19	69	35	0	0	0	0	103	112	22	0	64	0	0	0
arsA	2013866..2015623	15	13	53	22	26	39	16	0	46	74	91	183	25	8	36	19	14	36	5	0	0	21	21	46	27	32	66	8	14	22
CTC_RS09735	complement(2015986..2016717)	0	0	18	0	0	0	0	0	36	8	0	33	10	0	0	0	0	52	0	0	0	0	0	0	0	15	32	9	0	52
CTC_RS09740	complement(2016894..2017166)	0	0	0	0	84	0	0	0	0	0	0	0	0	0	0	0	0	0	16	0	0	0	0	0	0	0	0	0	0	0
CTC_RS09745	complement(2017344..2017907)	48	168	94	81	122	92	0	84	47	304	28	0	0	104	37	12	0	0	30	0	42	66	0	0	14	40	206	122	0	0
CTC_RS09750	complement(2017897..2018361)	0	51	0	33	49	74	0	0	115	114	0	26	0	31	45	29	0	0	91	0	51	0	80	0	0	0	0	59	53	0
CTC_RS09755	2018610..2019830	0	670	338	6	319	85	0	322	175	0	494	59	0	1283	241	0	227	124	0	119	176	0	214	0	0	604	342	0	121	220
CTC_RS09760	2019983..2020540	0	850	1954	14	205	247	0	832	670	0	1699	428	0	1417	789	12	1537	1021	15	433	214	0	535	292	0	1232	1143	0	1254	515
CTC_RS14745	complement(2020937..2021113)	0	0	0	0	0	0	0	0	0	0	0	135	0	0	0	0	0	72	0	0	0	0	0	0	0	0	0	0	0	0
CTC_RS09765	2021141..2022811	16	0	48	0	14	10	11	28	16	14	38	14	0	0	38	0	106	30	3	0	0	7	0	24	0	0	42	12	15	34
CTC_RS09770	2022901..2023878	0	0	41	0	0	18	0	16	0	12	0	0	7	0	43	0	26	13	4	0	73	0	0	0	0	0	0	0	0	0
CTC_RS09775	2023871..2024707	16	0	16	9	0	0	0	19	0	21	0	0	0	17	50	0	0	15	0	0	0	0	0	0	0	13	0	8	29	0
CTC_RS09780	2024720..2025541	0	29	49	19	84	21	11	57	32	14	0	58	18	27	0	25	31	15	5	0	58	60	91	0	10	27	85	33	30	23
CTC_RS09785	2025541..2026143	0	79	0	0	0	0	0	0	0	29	27	0	12	0	35	11	126	21	0	0	0	0	0	0	0	19	0	11	0	32
CTC_RS09790	complement(2026226..2027530)	41	36	10	41	0	0	14	24	0	104	172	110	11	11	16	47	19	29	59	0	37	67	0	0	12	9	0	68	19	29
CTC_RS09795	complement(2027523..2028221)	0	34	0	22	0	25	0	45	0	76	69	0	21	0	0	88	0	72	73	138	0	0	0	0	23	0	0	59	35	82
CTC_RS09800	complement(2028647..2029771)	12	0	24	41	41	15	41	14	24	52	57	0	32	39	19	109	112	56	147	64	128	22	33	0	28	20	41	244	44	34
CTC_RS15020	complement(2030221..2030313)	0	0	0	0	0	0	0	0	0	0	0	0	0	0	0	0	0	0	0	0	0	0	0	0	0	0	0	0	0	0
CTC_RS09805	complement(2030365..2030649)	0	250	420	134	0	121	33	331	281	21	338	84	76	257	294	96	0	0	67	0	168	0	131	143	56	431	81	0	0	67
CTC_RS09810	complement(2030650..2030952)	0	0	0	0	76	57	62	0	264	78	53	0	0	0	138	0	0	0	21	0	0	0	0	134	26	74	0	23	0	0
greA_2	complement(2031229..2031693)	58	51	143	49	99	148	40	135	115	127	52	0	62	157	135	59	54	245	0	0	51	27	161	175	0	96	100	89	53	103
CTC_RS09820	complement(2031919..2032242)	0	0	0	71	0	0	0	0	0	18	0	74	0	0	0	0	0	0	0	0	0	0	0	126	0	0	0	0	0	0
CTC_RS09825	2032348..2032746	0	0	0	0	0	0	0	0	0	15	0	0	0	0	0	68	63	95	11	0	0	0	0	0	0	0	0	52	0	0
CTC_RS09830	complement(2032848..2035820)	0	0	0	4	15	6	0	0	9	34	0	48	0	0	0	5	8	17	19	0	0	0	0	7	0	0	0	30	50	39
CTC_RS09835	complement(2036218..2037690)	28	48	36	73	47	70	25	43	0	124	22	32	29	45	14	32	51	120	89	66	73	17	0	0	16	76	31	103	83	260
CTC_RS09840	complement(2037687..2039495)	30	66	59	122	139	105	26	61	30	150	18	40	32	0	70	45	0	70	143	80	119	21	62	45	13	31	26	68	41	101
CTC_RS09845	complement(2039485..2042472)	23	8	9	41	38	69	9	47	18	99	38	16	27	73	21	41	17	68	71	32	64	21	12	14	11	37	0	25	25	64
CTC_RS09850	complement(2043201..2045789)	78	82	82	0	0	0	65	61	52	36	43	212	50	74	24	68	88	117	0	0	0	48	58	142	21	39	143	42	9	133
CTC_RS09855	complement(2045786..2046469)	20	0	78	0	0	0	27	46	0	26	0	174	32	21	123	80	111	111	0	0	0	54	55	60	12	16	68	10	0	84
CTC_RS09860	complement(2046552..2047541)	55	72	40	0	0	0	66	24	54	36	32	145	44	59	64	131	127	115	0	0	0	100	0	82	72	79	70	49	25	155
CTC_RS09865	complement(2047528..2048214)	39	138	39	0	0	0	68	92	0	52	152	313	63	64	31	149	184	129	0	0	0	54	54	59	81	98	169	50	36	167
CTC_RS09870	complement(2048211..2048405)	0	0	0	0	0	0	0	0	0	0	0	0	0	0	0	35	129	0	0	0	0	0	0	0	0	57	0	0	0	0
CTC_RS14755	complement(2048484..2048678)	0	0	0	0	0	0	0	0	0	0	0	0	0	0	0	0	0	0	0	0	0	0	0	0	0	0	0	0	0	0
CTC_RS15025	complement(2048819..2049106)	0	0	0	0	0	0	0	0	0	21	0	166	0	0	0	0	0	0	0	0	0	0	0	0	0	0	0	0	0	33
CTC_RS15030	complement(2049399..2049575)	0	0	0	0	0	0	0	0	0	0	0	0	0	0	0	0	0	0	0	137	0	0	0	0	0	0	0	0	0	217
CTC_RS14880	complement(2049557..2049730)	0	0	0	0	0	50	0	0	0	0	0	0	0	0	121	0	0	0	0	0	0	0	0	0	91	0	0	0	0	0
CTC_RS09880	complement(2049793..2050185)	0	60	0	0	0	0	0	0	0	15	0	0	18	0	0	17	0	32	0	0	0	0	0	0	0	0	0	35	62	0
CTC_RS09885	complement(2050517..2051134)	0	0	129	0	0	0	0	25	86	76	52	193	0	0	68	11	122	20	0	0	0	0	60	264	13	18	0	0	0	0
CTC_RS09895	2051876..2052550	0	0	39	0	0	0	0	0	79	17	0	106	0	0	31	0	112	0	0	0	0	0	55	60	0	0	34	0	0	0
CTC_RS09900	complement(2052894..2053541)	0	0	0	0	0	0	0	0	0	32	12	37	0	0	0	74	0	29	7	0	0	0	58	0	12	0	0	0	0	0
CTC_RS09905	complement(2053689..2053901)	0	111	0	36	0	0	0	0	0	0	0	0	0	0	0	0	0	0	20	0	0	0	0	0	37	52	0	0	0	0
CTC_RS09910	complement(2053903..2054295)	0	0	0	58	0	132	24	0	204	0	0	0	0	37	53	0	0	0	11	0	0	0	0	104	20	28	0	0	0	0
CTC_RS09915	complement(2054549..2054860)	0	76	0	0	0	0	15	0	0	0	0	0	0	0	0	0	0	0	0	0	0	0	0	0	13	36	0	0	0	0
CTC_RS09920	complement(2054930..2056153)	0	19	11	0	0	0	0	0	0	0	0	39	0	36	34	0	0	0	0	0	0	0	0	0	0	0	19	0	0	0
CTC_RS09925	complement(2056143..2056601)	0	0	0	0	0	0	0	0	0	0	0	0	0	0	0	0	0	0	0	0	0	0	0	0	17	0	0	0	0	0
CTC_RS09930	complement(2056796..2057179)	563	1483	2078	318	1254	1975	607	943	2365	0	0	0	451	953	2103	0	0	0	376	2393	2554	485	1555	2863	372	640	2264	251	5209	2745
CTC_RS09935	complement(2057176..2057418)	167	98	930	377	377	2412	230	324	550	0	0	0	208	120	691	0	0	0	140	1593	1083	306	768	1340	294	322	334	170	2626	1341
CTC_RS09940	complement(2057478..2057687)	0	0	63	0	109	0	0	0	0	0	0	0	0	0	0	0	0	0	0	230	0	0	0	0	0	0	0	0	0	91
CTC_RS14595	2057695..2058159	0	0	0	0	0	0	0	0	0	0	0	0	0	0	0	0	0	0	0	0	0	0	0	0	0	0	0	0	0	0
CTC_RS09950	complement(2058191..2058736)	0	65	0	0	0	0	0	0	0	0	0	0	0	0	0	0	0	0	0	0	0	0	0	0	0	0	0	38	0	0
CTC_RS09955	complement(2058779..2059435)	0	0	0	0	0	0	0	0	0	0	0	0	0	0	0	0	0	0	0	0	0	0	0	0	0	0	0	0	0	0
CTC_RS09960	complement(2059432..2060217)	0	0	0	0	0	0	0	0	0	0	0	0	0	0	13	0	0	0	0	0	0	0	0	0	0	0	0	0	0	0
CTC_RS09965	2060399..2060614	0	0	0	0	0	0	0	0	0	0	0	0	0	0	0	0	0	0	0	0	0	0	0	0	0	0	0	0	0	0
CTC_RS09970	complement(2060653..2062320)	0	0	0	0	0	0	0	0	0	0	0	0	0	0	0	0	0	0	0	0	0	0	0	0	0	0	0	0	0	0
CTC_RS09975	complement(2062295..2062561)	0	0	0	0	0	0	0	0	0	0	0	0	0	0	0	0	0	0	0	0	0	0	0	0	0	0	0	0	0	0
CTC_RS09980	complement(2062572..2063540)	0	0	0	0	0	0	0	0	0	0	0	0	0	0	0	0	0	0	0	0	0	0	0	0	0	0	0	0	0	0
rlmD_1	complement(2063929..2065293)	99	139	175	212	34	88	89	69	39	452	130	105	143	139	123	217	37	186	246	35	53	109	82	239	139	180	136	327	144	169
CTC_RS09990	complement(2065312..2065680)	37	0	108	62	62	187	25	0	72	96	87	0	59	119	0	55	0	103	35	0	130	34	0	0	43	0	0	75	0	104
CTC_RS09995	2065939..2067807	0	51	43	8	49	83	0	17	14	101	34	0	4	47	0	0	0	27	14	26	115	13	0	0	4	30	12	70	53	82
CTC_RS10000	complement(2067862..2068638)	104	366	1095	403	797	843	240	425	584	661	1386	707	149	462	891	307	584	750	364	1121	1539	256	841	1179	184	331	865	323	995	950
pepV	complement(2068819..2070219)	328	525	626	520	687	775	576	595	782	586	964	1158	366	512	898	418	864	660	334	794	777	540	706	814	433	423	803	280	517	561
CTC_RS10010	2070500..2071393	45	27	0	9	0	96	10	35	0	92	18	0	24	16	23	15	56	71	10	0	54	0	0	46	18	13	26	15	0	43
CTC_RS10015	complement(2071440..2072752)	51	199	122	41	227	118	43	252	163	207	184	36	11	67	96	52	38	48	26	74	91	47	142	93	36	170	35	79	75	15
CTC_RS10020	2073188..2074369	11	60	23	13	39	0	8	40	0	10	122	40	6	50	35	0	85	11	7	41	0	0	0	34	0	19	0	0	62	16
CTC_RS10025	complement(2074515..2076044)	0	31	17	30	45	23	24	21	0	27	0	109	19	10	14	31	49	50	28	32	86	8	24	0	10	95	15	49	48	13
CTC_RS10030	complement(2076037..2077452)	10	84	47	27	81	61	26	89	75	21	34	51	20	31	15	48	18	63	30	34	51	9	53	0	6	32	16	78	35	54
CTC_RS10035	complement(2077470..2078159)	39	138	116	33	100	50	14	160	39	86	70	138	63	85	30	30	0	128	92	140	35	90	270	59	11	178	134	60	107	194
cadA	complement(2078343..2080547)	37	75	368	239	333	813	17	29	194	612	532	1515	56	53	95	312	195	540	440	219	640	174	152	480	86	51	116	1726	111	752
pepF	2080705..2082507	0	0	15	21	51	19	0	0	0	23	0	0	0	16	0	19	0	14	5	0	13	7	0	0	0	6	0	11	0	21
hisS	complement(2082554..2083873)	215	593	494	515	434	431	399	596	688	338	457	416	383	654	667	694	344	403	606	183	308	423	594	524	336	940	755	453	205	857

CTC_RS10055	complement(2084059..2085000)	14	0	14	16	0	0	10	0	0	50	0	0	8	0	0	7	0	13	5	0	0	0	0	43	8	12	25	95	0	20
CTC_RS10060	complement(2085018..2086013)	14	0	0	15	0	17	0	16	0	30	0	0	22	0	0	34	0	13	26	0	24	50	37	0	16	0	23	90	49	19
purD	complement(2086236..2087483)	32	95	32	37	55	41	45	126	21	28	52	19	46	70	34	93	40	51	63	116	115	60	60	33	19	72	111	39	108	61
purH	complement(2087506..2089005)	36	47	44	36	31	69	44	52	36	47	32	64	19	49	168	27	0	76	28	0	48	33	0	54	37	22	46	23	33	51
purN	complement(2089019..2089639)	0	0	21	37	37	56	30	25	0	10	129	0	12	24	68	0	0	61	14	0	0	20	60	0	13	0	0	11	0	31
purM	complement(2089667..2090662)	14	24	40	31	23	17	66	32	0	24	0	96	22	0	42	21	0	38	47	49	0	12	0	0	24	34	23	0	0	58
purF	complement(2090681..2092078)	39	34	19	19	33	37	67	45	57	46	11	68	15	31	15	58	0	45	33	35	34	0	0	29	28	56	17	10	18	14
purE	complement(2092156..2092635)	0	0	0	0	0	108	0	0	0	0	0	99	0	61	0	0	0	0	0	0	50	0	78	0	17	0	0	0	0	0
CTC_RS10095	2092849..2093514	61	0	60	34	34	39	14	47	0	137	0	0	0	44	63	31	0	19	13	73	36	9	56	0	0	34	0	21	0	0
CTC_RS10100	complement(2093580..2093825)	220	675	865	1706	1491	631	493	832	1303	0	2156	97	910	893	853	858	1026	412	346	393	875	1412	1821	331	774	818	754	28	698	468
CTC_RS10105	complement(2093943..2094521)	0	0	0	26	0	0	0	0	0	10	56	41	12	25	0	59	174	22	7	84	124	0	0	0	27	19	0	12	212	33
CTC_RS10110	complement(2094541..2095230)	0	0	0	22	66	0	0	0	0	0	23	0	0	0	0	0	0	18	6	0	0	0	0	0	0	0	17	0	0	28
CTC_RS14765	2095629..2095898	0	0	0	0	0	0	0	0	0	0	0	0	0	0	0	0	0	47	0	0	0	0	0	0	0	83	0	0	0	0
CTC_RS10120	2095915..2096358	0	267	120	0	52	0	0	638	842	0	72	215	16	725	472	15	511	200	10	0	54	0	168	642	0	629	104	31	884	259
CTC_RS10125	2096499..2097230	0	32	0	0	0	0	0	0	0	73	0	0	10	0	0	0	0	0	0	0	33	0	0	0	0	0	32	0	0	79
CTC_RS10130	complement(2097271..2098731)	0	0	0	5	31	12	26	11	9	18	0	16	0	0	14	61	86	113	3	0	16	8	26	0	0	23	0	19	0	92
CTC_RS10135	complement(2098988..2100382)	19	17	67	16	49	105	7	34	38	214	35	137	10	10	15	10	18	100	21	139	120	9	27	117	0	32	83	49	18	110
CTC_RS10140	complement(2100952..2101509)	218	383	500	41	493	772	267	197	287	53	1008	171	52	236	413	55	1854	227	46	1127	257	111	535	584	99	200	249	111	44	412
CTC_RS10145	complement(2101651..2103117)	976	2692	3621	609	4820	8201	324	2264	3104	1119	6903	4303	89	783	1315	448	3414	2058	430	4583	5218	161	4223	7507	108	724	1359	1005	6224	2842
CTC_RS10150	2103331..2105019	8	0	16	0	14	41	6	28	47	28	14	14	0	9	12	12	0	37	8	0	14	0	0	24	0	7	0	20	15	45
CTC_RS10155	2105016..2105783	0	31	0	0	60	0	0	0	0	15	0	0	9	0	0	27	0	33	17	0	93	16	0	0	0	0	0	0	0	25
CTC_RS14325	2106159..2108327	1318	1859	1812	1889	2948	2905	1178	1727	1903	2278	2683	1997	958	1161	517	2212	1024	1424	1284	1740	2206	1716	2874	2393	932	747	722	596	747	2634
sdaAB	2108449..2109135	334	207	252	156	200	452	299	263	311	318	257	313	247	309	92	367	294	479	291	599	766	135	652	652	300	309	101	190	286	558
sdaAA	2109136..2110008	495	217	640	354	670	691	299	415	811	386	377	301	446	352	312	328	405	573	273	831	904	405	599	1073	518	423	478	291	365	922
CTC_RS10175	complement(2110061..2110951)	15	53	75	17	77	135	10	18	150	113	72	27	8	16	24	0	0	0	48	54	54	14	0	183	0	13	26	62	83	108
CTC_RS10180	2111145..2111525	0	0	0	20	60	0	0	0	70	31	42	0	19	0	0	0	0	0	0	0	0	33	196	107	21	0	61	18	0	50
CTC_RS10185	2111530..2112426	0	0	0	9	26	0	10	0	0	20	0	0	8	0	0	0	0	0	5	0	0	14	0	0	0	0	0	0	0	0
CTC_RS10190	2112407..2113471	0	0	0	0	0	8	0	0	0	6	0	0	0	14	0	0	0	0	0	0	11	0	0	0	0	0	0	0	23	0
CTC_RS10195	2113489..2114181	0	0	19	11	0	0	0	0	0	0	0	0	0	0	0	0	0	0	0	0	0	18	0	0	11	0	0	10	0	0
CTC_RS10200	2114182..2114964	0	0	0	39	29	0	0	0	0	0	0	30	9	0	0	0	0	0	0	0	0	48	0	0	0	0	0	0	31	0
CTC_RS10210	2115094..2116430	0	0	0	0	0	0	0	0	0	4	0	0	0	0	0	0	0	0	0	0	0	0	0	0	0	0	0	0	0	0
CTC_RS10215	complement(2116507..2118579)	72	435	510	134	475	840	50	342	399	71	2171	2274	211	406	602	223	1114	305	82	1843	1194	138	558	550	184	464	526	179	699	388
CTC_RS14770	2118829..2118966	0	0	0	0	0	0	0	0	0	0	0	0	0	0	0	0	0	0	0	0	0	0	0	0	0	0	0	0	0	0
CTC_RS10220	2118985..2119548	0	0	0	0	0	0	0	0	0	0	0	0	0	0	0	0	0	0	0	0	0	0	0	0	0	0	0	0	0	0
CTC_RS10225	complement(2119615..2120919)	1232	1590	1396	1479	1915	1988	1541	2129	1934	466	4188	3969	1449	1621	2122	1143	2677	1373	1574	5838	2841	1350	1301	1872	1616	1769	1768	522	1918	1829
deoC	complement(2120950..2121600)	1578	3425	2788	2004	3204	2926	1913	2430	3200	798	4802	5500	1818	2058	3222	1308	3216	2353	1757	6093	4281	2373	2408	2564	1766	2232	3668	475	2488	2400
CTC_RS10235	complement(2121746..2123527)	387	1211	1937	467	1608	3540	291	1475	2166	139	9764	6496	474	1306	2419	596	4382	931	382	4818	3866	494	1340	3107	416	1048	2036	357	1866	801
CTC_RS10240	complement(2123661..2123933)	545	0	195	335	336	316	308	288	391	260	942	0	185	107	77	299	92	186	171	532	175	364	410	597	407	82	0	126	90	351
CTC_RS10245	2124330..2125034	1476	908	321	2506	1236	465	582	603	114	142	673	508	1208	218	238	1483	429	54	862	1510	611	1707	1403	577	1114	222	263	224	4300	218
CTC_RS10250	2125192..2126427	33	19	11	49	74	14	15	0	22	48	52	58	47	71	34	50	41	41	28	0	19	60	91	49	38	54	38	28	119	109
CTC_RS10255	complement(2126483..2127433)	1009	985	1035	1970	2266	3208	1133	1324	1601	3239	1403	1907	638	739	1390	2252	902	1757	1732	1323	2390	1253	2119	2954	796	834	1365	2469	877	3104
CTC_RS10260	complement(2128052..2128216)	737	647	1128	2151	556	627	339	906	1619	4152	487	579	962	976	890	1589	612	1304	4612	0	2610	1053	1357	247	384	779	1265	7375	1339	2323
CTC_RS10265	2128461..2130035	43	15	118	141	131	142	24	90	204	442	51	76	55	74	146	125	112	161	132	0	91	24	47	258	43	64	59	229	16	353
CTC_RS10270	complement(2130075..2131655)	120	165	135	191	203	142	130	149	84	303	163	468	75	120	159	162	255	280	245	337	151	133	142	52	75	163	103	170	186	242
CTC_RS10275	complement(2131785..2132663)	46	27	106	61	104	196	85	0	61	571	73	244	33	67	143	31	144	194	145	0	218	28	0	232	9	51	106	305	56	218
CTC_RS10280	2132838..2133563	19	0	0	0	0	0	0	0	0	24	0	0	0	0	0	0	0	0	6	0	0	0	0	56	0	0	0	9	0	0
CTC_RS10285	complement(2133591..2134550)	0	74	42	8	72	72	0	33	0	37	33	25	8	0	0	21	53	79	27	50	0	13	117	42	0	47	24	43	0	40
CTC_RS14330	complement(2135079..2135807)	19	0	36	42	31	166	154	0	0	49	110	131	79	0	58	19	104	174	50	66	98	34	0	195	76	15	32	28	67	26
CTC_RS10295	complement(2135868..2136683)	83	407	106	150	197	275	23	154	164	362	138	234	97	108	77	150	155	171	211	237	469	76	137	100	58	55	57	177	90	258
CTC_RS10300	complement(2136680..2137363)	59	0	58	0	134	151	14	23	0	69	70	105	0	0	0	0	184	93	62	71	35	0	109	0	12	65	68	20	0	0
CTC_RS10305	complement(2137383..2138267)	15	27	45	9	0	117	11	18	60	7	18	81	0	0	0	8	29	14	19	55	27	0	0	0	9	13	79	0	0	65
rpoD_2	complement(2138329..2139396)	810	289	311	750	386	420	708	398	300	376	436	346	862	254	452	848	661	498	1035	317	538	546	594	381	854	262	412	463	437	431
dnaG	complement(2139416..2141185)	206	40	53	177	142	97	174	27	45	127	109	94	120	33	107	239	43	186	231	0	189	91	84	138	134	51	39	82	55	130
CTC_RS10320	complement(2141435..2142469)	39	46	90	0	0	17	9	0	0	17	62	23	0	42	41	33	49	61	66	0	46	48	0	0	0	11	0	46	24	37
CTC_RS10325	2142648..2143715	0	22	12	0	21	16	0	0	0	0	15	67	0	14	0	0	47	12	0	0	22	0	0	0	0	31	22	0	23	18
ppdK	complement(2143810..2146443)	190	513	992	240	496	681	156	323	497	410	809	1350	129	350	669	284	1657	920	258	808	599	141	411	294	126	214	546	290	727	779
CTC_RS10335	complement(2146492..2147136)	42	110	412	130	71	588	14	171	166	156	424	370	22	23	130	74	313	432	119	300	260	19	58	505	12	69	180	160	266	475
CTC_RS10340	complement(2147379..2147933)	170	342	216	124	289	497	84	199	193	293	695	516	130	106	113	258	455	251	77	697	388	201	336	440	143	121	251	173	265	345
recO	complement(2147935..2148678)	254	128	340	256	339	278	226	254	144	302	324	257	194	374	254	174	305	340	229	325	514	83	201	274	107	255	93	102	132	451
era	complement(2148695..2149582)	350	454	374	421	568	563	326	558	541	349	552	551	252	511	661	345	227	599	555	599	485	391	378	642	205	667	274	217	318	604
CTC_RS10355	complement(2149592..2149990)	169	297	267	249	402	302	304	395	268	178	161	120	289	404	368	290	253	159	341	727	779	93	748	510	99	224	0	155	123	144
CTC_RS10360	complement(2150074..2150775)	0	135	152	54	65	172	93	112	76	126	206	102	21	188	30	49	467	162	73	0	375	88	159	464	56	96	165	29	122	27
ybeY	complement(2150802..2151305)	54	282	290	121	364	239	56	281	424	158	574	426	72	407	83	162	350	327	110	864	427	49	74	808	16	444	276	95	317	266
CTC_RS10370	complement(2151324..2153375)	33	266	337	74	268	475	36	222	260	147	352	378	18	136	92	50	246	265	59	165	501	27	164	397	35	131	147	60	108	411
yqfD	complement(2153449..2154633)	11	40	22	0	19	58	0	13	23	25	122	0	6	74	35	6	85	96	36	0	40	0	63	69	0	38	0	12	145	97
yqfC	complement(2154617..2154901)	0	166	0	27	0	0	0	166	187	21	226	0	0	257	74	0	266	0	0	0	0	44	0	0	0	196	81	0	172	135
CTC_RS10385	complement(2155014..2155469)	5735	3485	2975	6025	2865	1588	5391	4125	3602	4844	3278	4371	5635	4302	3657	6326	2932	4165	6205	3713	3672	5115	2374	1696	5374	4130	3254	6257	3391	3195
rpsU	complement(2155496..2155672)	2100	1273	1503	2026	1036	974	2214	1779	2867	2853	1635	2292	2814	2647	3437	3694	2851	2396	4156	273	2027	3084	633	1380	2845	2652	3537	4603	1387	2707
CTC_RS10395	complement(2155822..2156166)	1058	206	655	929	930	1049	1487	593	1626	976	978	623	1109	764	1338	1678	439	1211	665	421	1248	1223	1082	1298	1356	389	806	478	356	1556
mtaB	complement(2156269..2157570)	31	73	20	59	18	66	50	60	41	197	111	92	58	22	97	86	19	107	104	74	18	10	0	31	43	60	0	158	57	59
CTC_RS10405	complement(2157570..2158322)	36	32	0	20	0	23	0	21	0	94	43	95	10	0	56	63	0	67	73	64	0	33	149	108	0	45	31	82	33	51
prmA	complement(2158335..2159279)	29	25	70	57	49	73	30	17	0	131	77	51	46	31	67	36	0	121	49	0	0	53	79	0	8	59	25	58	0	61
dnaJ	complement(2159632..2160759)	467	946	595	687	1077	749	645	1116	592	665	948	1333	864	753	669	815	693	1201	861	1372	1633	792	827	650	1055	723	884	451	413	816
dnaK	complement(2160832..2162688)	979	1705	1375	886	1901	1467	1085	1441	1295	585	1190	1163	1112	840	1570	1137	1440	1667	750	1355	1630	1173	1688	1809	1358	1159	1111	698	806	888
grpE	complement(2162754..2163335)	70	1141	366	393	709	681	417	609	734	325	787	923	248	478	1135	433	1344	1196	384	332	904	341	1090	979	259	615	876	248	380	461
hrcA	complement(2163380..2164405)	132	439	298	119	804	470	164	353	182	294	595	442	113	385	470	193	418	494	120	283	921	206	364	754	77	261	181	94	311	523
hemW	complement(2164602..2165750)	12	0	12	0	0	0	0	0	0	10	0	21	6	0	18	18	22	22	0	0	0	0	0	0	0	10	0	0	21	17
lepA	complement(2165822..2167630)	22	105	85	105	127	114	57	135	96	251	98	106	64	129	70	143	42	112	189	134	93	62	41	135	57	154	103	217	109	127
CTC_RS10445	complement(2167707..2168063)	0	0	0	0	0	0	0	0	0	0	0	0	0	0	59	0	0	0	0	0	0	0	0	0	0	0	0	0	0	0
CTC_RS10450	complement(2168123..2169178)	0	0	0	0	22	16	0	0	0	0	0	23	0	0	20	0	24	24	0	0	0	0	35	0	0	11	0	0	0	36

CTC_RS10455	complement(2169242..2170291)	0	0	13	0	22	0	0	45	0	11	61	0	0	28	0	0	0	12	0	46	0	0	0	0	0	21	22	0	23	0
gpr	complement(2170342..2171313)	0	24	55	0	0	0	0	32	55	0	50	0	7	75	0	7	26	26	9	0	25	0	38	0	0	92	48	14	25	0
rpsT	2171512..2171769	1257	1747	1340	1538	1244	2405	832	854	1967	3983	997	2220	783	1135	976	1188	587	1669	2266	187	1762	818	1881	1894	876	780	1438	3890	761	2823
holA	complement(2171813..2172847)	0	0	0	29	0	33	9	0	26	29	16	69	0	0	20	0	0	49	21	0	46	0	36	39	8	22	0	27	24	19
CTC_RS10475	complement(2172868..2174544)	0	14	0	0	0	21	0	0	0	0	10	14	4	0	0	4	0	4	5	29	0	0	0	0	0	0	14	0	0	0
CTC_RS10480	complement(2174705..2177182)	0	0	5	6	28	0	4	32	0	10	0	29	3	0	0	3	41	15	2	10	19	5	0	16	3	0	28	6	10	23
CTC_RS10485	complement(2177251..2177532)	0	252	47	0	81	0	0	112	189	84	57	85	0	104	223	48	89	135	45	0	170	0	0	0	28	0	82	98	0	68
CTC_RS10490	2177733..2178359	0	0	64	37	37	27	0	0	0	66	0	0	0	0	0	0	40	0	0	0	0	0	0	0	0	0	37	0	0	92
CTC_RS10495	complement(2178426..2179424)	0	237	200	53	298	673	103	189	214	118	274	215	29	73	273	34	303	570	55	242	694	87	448	571	56	257	302	34	344	710
lpdA	complement(2179414..2181102)	40	379	134	136	394	378	130	466	237	28	181	254	175	407	348	117	388	555	211	802	892	121	486	410	131	390	371	31	334	420
CTC_RS10505	complement(2181209..2181643)	31	273	168	184	474	634	129	398	184	122	185	110	50	370	338	78	174	320	151	334	330	57	686	468	36	231	160	174	226	264
CTC_RS10510	complement(2181800..2182189)	104	243	989	274	823	1679	144	908	1096	560	989	857	130	488	484	210	906	1331	120	1116	1717	127	574	1200	102	172	594	247	755	2359
CTC_RS10515	complement(2182591..2183157)	0	0	23	0	0	0	0	0	0	0	0	0	0	0	0	0	22	22	0	0	0	0	0	0	0	0	41	0	0	0
CTC_RS10520	2183278..2184516	0	0	32	0	19	28	15	13	65	76	13	58	12	0	34	11	0	31	10	0	0	20	0	0	6	0	19	28	20	46
CTC_RS10525	complement(2184566..2185471)	45	79	59	84	127	76	10	139	118	137	53	184	48	81	93	98	111	182	174	214	343	41	124	45	35	62	51	61	81	169
CTC_RS10530	complement(2185493..2186716)	22	58	65	44	75	197	15	64	44	135	13	117	41	96	103	95	62	228	137	40	235	61	30	0	39	18	19	56	160	141
yqeK	complement(2186723..2187292)	0	83	23	13	80	60	0	28	0	31	56	42	0	0	0	0	133	44	15	0	84	0	131	0	0	20	81	36	0	0
nadD	complement(2187310..2187912)	0	39	22	0	38	129	62	78	0	98	160	0	24	97	70	34	126	63	78	0	79	0	62	135	13	167	38	0	0	127
yhbY	complement(2187932..2188225)	230	403	90	363	390	235	254	321	454	723	492	325	295	199	428	174	257	301	456	329	244	169	381	692	202	342	315	398	83	587
obgE	complement(2188249..2189526)	264	334	260	430	161	351	314	431	251	578	453	374	401	298	263	586	217	451	627	189	730	437	263	350	267	551	381	533	173	450
CTC_RS10555	complement(2189545..2190087)	25	44	24	112	0	63	103	0	0	141	0	88	13	108	0	151	93	93	172	89	0	91	69	0	102	103	43	165	45	106
rpmA	complement(2190149..2190454)	1435	1938	2129	1995	1124	2253	854	1800	2575	3754	945	3354	2123	2248	4935	2972	2391	2441	5773	1897	2267	1764	2317	1197	1685	2703	3902	8301	1925	1785
CTC_RS10565	complement(2190456..2190785)	983	1222	1310	2035	1632	3604	565	1240	1295	7964	877	1193	1640	1286	1081	1218	841	1611	3092	1319	1377	1429	679	1727	1105	1253	1475	5375	669	1830
rplU	complement(2190788..2191102)	2188	2786	2111	3598	2583	2161	1747	2199	2968	5784	1939	1402	2532	2812	2264	3114	561	1568	8571	2611	1747	3072	1007	1292	3210	2431	3386	8391	1403	3560
CTC_RS10575	complement(2191259..2192692)	0	50	46	11	64	60	0	44	0	91	0	50	25	10	44	36	123	88	42	0	50	9	104	0	11	16	0	29	17	27
CTC_RS10580	complement(2192719..2193429)	19	0	37	32	97	73	0	44	38	66	0	101	41	82	30	57	35	36	60	68	0	0	52	0	22	47	0	58	69	27
CTC_RS10585	complement(2193398..2195260)	36	166	71	98	160	167	60	177	158	143	104	51	105	228	34	117	95	129	147	91	51	47	140	109	85	84	112	151	211	103
CTC_RS10590	complement(2195475..2196323)	0	28	0	0	0	20	0	37	0	7	19	0	0	17	0	0	0	0	0	0	0	0	44	0	0	13	0	0	0	0
CTC_RS10595	complement(2196338..2197111)	0	31	17	10	30	0	0	122	0	0	166	31	0	38	27	0	163	0	11	0	31	0	0	0	0	43	0	0	63	25
rodA	complement(2197247..2198368)	48	106	130	129	143	77	108	196	262	200	100	149	64	130	112	79	45	124	129	129	171	55	133	145	71	90	72	181	131	282
minE	complement(2198715..2198981)	683	267	224	857	687	129	629	472	400	177	301	358	676	713	79	1071	283	285	510	181	1165	511	699	0	891	167	347	283	276	144
minD	complement(2198993..2199790)	339	357	308	712	661	281	818	602	402	422	463	389	873	404	289	1135	269	555	535	576	360	591	374	459	835	420	378	250	338	625
minC	complement(2199803..2200435)	342	0	105	428	181	54	501	174	127	149	229	226	462	312	133	646	199	260	289	382	227	216	118	129	282	141	110	109	194	121
CTC_RS10620	complement(2200555..2203413)	61	91	107	173	152	96	88	99	84	263	62	75	81	77	29	141	238	244	196	85	218	91	65	100	47	125	81	123	52	107
mreD	complement(2203417..2203908)	0	0	27	0	93	35	0	0	0	168	33	0	0	0	0	7	51	103	26	0	194	0	76	0	0	23	0	42	100	39
mreC	complement(2203919..2204767)	80	28	16	81	135	142	66	56	94	174	76	28	60	17	0	132	178	89	135	114	28	29	44	48	9	26	55	69	231	113
CTC_RS10635	complement(2204771..2205787)	133	443	209	296	406	186	284	248	79	467	126	235	227	173	186	335	372	542	575	143	235	183	257	160	187	242	137	470	265	207
radC	complement(2205807..2206493)	20	0	39	44	33	100	14	46	156	172	94	35	21	21	31	50	0	111	161	141	104	54	163	119	35	98	34	170	36	28
CTC_RS10645	complement(2206509..2207087)	70	82	23	53	79	208	64	136	92	393	111	206	100	240	0	129	0	306	294	418	124	43	193	281	68	58	80	249	42	66
CTC_RS10650	complement(2207586..2208269)	0	0	19	0	0	0	0	23	0	0	0	70	0	64	0	0	0	37	0	0	0	0	0	0	0	33	0	0	0	0
CTC_RS10655	complement(2208411..2209010)	473	1008	2017	598	1987	1321	466	1364	2270	1034	1661	2188	517	1659	1363	499	547	675	655	1451	2153	517	498	814	568	1174	1739	298	450	1645
CTC_RS10660	complement(2209220..2209861)	168	259	207	83	464	107	291	172	541	37	200	186	90	456	425	265	79	118	126	301	224	116	291	63	124	331	397	43	115	418
CTC_RS14335	complement(2209872..2210369)	27	0	27	15	46	173	0	32	54	24	0	48	0	88	253	41	51	102	43	0	48	0	75	82	32	67	0	28	0	77
CTC_RS10670	2210507..2211646	0	874	222	0	342	91	0	635	492	5	1156	272	0	1194	294	0	620	122	0	170	63	0	164	36	0	814	458	0	538	639
CTC_RS10675	complement(2211762..2212760)	0	166	53	15	0	17	9	126	53	30	161	72	40	249	42	0	0	0	26	0	24	0	37	0	16	207	162	14	98	0
CTC_RS10680	complement(2212772..2213560)	0	241	0	0	29	0	0	140	68	0	122	121	0	334	80	0	64	32	0	61	30	0	95	0	0	269	88	4	0	0
CTC_RS10685	complement(2213567..2214709)	0	270	23	0	80	0	0	41	23	21	197	42	6	179	165	18	66	22	4	0	0	0	65	0	0	264	20	0	43	17
CTC_RS10690	complement(2214856..2215251)	0	240	101	39	58	0	24	278	0	0	446	0	0	370	0	0	64	32	21	122	0	0	0	0	0	226	117	17	124	145
CTC_RS10695	complement(2215269..2215715)	0	796	178	0	308	39	0	458	0	0	683	0	0	1179	94	15	621	28	0	108	54	0	250	0	0	1025	207	0	439	257
CTC_RS10700	complement(2215737..2216927)	0	538	45	0	173	14	0	225	22	0	202	20	0	479	35	0	127	85	0	203	20	0	63	0	0	460	0	6	185	32
CTC_RS10705	complement(2217022..2218365)	0	53	0	6	51	38	0	70	0	0	120	0	5	120	16	0	56	28	0	0	36	0	0	0	0	50	17	10	55	14
CTC_RS10710	2218530..2219819	31	202	201	41	346	207	25	262	176	39	131	83	59	397	309	16	39	39	30	37	352	43	87	95	43	373	306	0	76	45
recQ	complement(2219892..2222012)	13	34	38	14	38	49	18	37	13	17	15	45	17	48	20	43	48	78	24	23	23	29	53	0	26	37	22	42	58	99
CTC_RS10720	2222188..2222742	0	0	24	14	41	0	0	0	0	43	58	0	0	26	0	0	0	0	0	0	0	0	0	0	0	0	0	0	0	69
CTC_RS10725	complement(2222964..2224490)	0	0	26	5	30	45	0	0	0	19	42	63	0	10	0	0	17	17	8	0	0	8	0	27	0	7	0	14	32	31
CTC_RS14340	complement(2224639..2226735)	52	215	120	257	328	353	82	218	204	132	429	433	89	157	180	193	96	254	262	369	456	237	561	447	91	133	166	179	375	640
CTC_RS10740	complement(2226837..2227934)	0	0	12	7	21	0	0	0	0	5	0	0	0	0	0	6	23	23	12	0	22	0	0	0	0	0	0	0	0	0
CTC_RS10745	complement(2227955..2229106)	0	10	12	0	20	0	0	41	0	0	153	21	0	25	18	0	44	11	11	0	0	0	0	0	7	29	0	0	85	0
CTC_RS10750	complement(2229103..2230659)	0	30	17	10	29	0	6	30	0	8	52	31	0	28	13	0	0	8	0	0	0	0	0	26	0	36	0	0	47	0
CTC_RS10755	complement(2230886..2233012)	13	67	6	4	0	8	9	4	25	7	0	0	17	45	20	58	36	48	0	0	0	0	0	0	0	60	33	13	12	9
CTC_RS10760	complement(2233073..2233711)	0	0	0	0	0	0	0	0	21	0	0	0	0	0	0	0	0	0	47	76	75	0	0	0	0	0	0	59	0	30
CTC_RS10765	complement(2233713..2235842)	13	11	3	14	97	97	0	4	0	54	19	11	0	3	0	6	0	6	24	0	67	9	88	38	0	0	0	37	12	108
CTC_RS10775	complement(2236490..2237035)	0	0	49	70	0	189	77	58	0	173	29	44	106	54	38	87	139	93	101	89	44	136	68	0	29	41	42	63	135	0
coaE	complement(2237052..2237594)	124	44	24	63	0	32	103	29	25	98	0	0	40	0	77	88	0	47	59	89	88	69	0	0	0	62	85	51	90	35
polA	complement(2237704..2240334)	46	63	45	87	87	92	67	18	20	131	49	54	60	56	24	91	10	77	84	0	109	47	50	77	44	30	44	76	47	58
aroC	complement(2240356..2241516)	47	41	23	13	20	0	72	14	92	61	55	21	19	25	0	29	22	98	59	0	103	11	32	70	7	39	40	12	21	33
CTC_RS10795	2241760..2242416	0	0	20	0	35	26	14	0	41	207	0	0	22	0	0	41	77	0	0	0	0	28	0	62	0	17	0	10	0	0
CTC_RS10800	complement(2242455..2242931)	0	0	28	0	0	0	0	0	0	37	34	0	0	0	0	7	0	27	9	0	0	26	0	0	0	0	0	14	0	40
CTC_RS10810	complement(2243230..2243517)	0	0	0	0	0	0	0	0	0	0	0	0	0	0	0	0	0	0	0	0	0	0	0	0	0	0	0	0	0	0
CTC_RS10815	complement(2243717..2243869)	0	0	0	0	0	0	0	0	0	0	0	0	0	0	0	0	0	0	0	0	0	0	0	0	0	0	0	0	0	0
CTC_RS10820	2244030..2244266	0	0	0	0	0	0	0	0	0	0	0	0	0	0	0	0	0	0	0	0	0	0	0	0	0	0	0	29	104	0
CTC_RS10825	complement(2244315..2244782)	0	0	0	0	0	0	0	0	0	0	0	0	0	0	0	0	0	0	0	103	0	0	0	0	0	0	0	0	0	0
CTC_RS10830	complement(2244783..2245454)	0	0	0	0	0	0	0	0	0	0	0	0	0	0	0	0	0	0	0	0	0	0	0	0	0	0	0	0	0	0
CTC_RS14605	complement(2245647..2246447)	0	0	33	10	100	291	0	0	50	0	0	0	0	0	26	0	0	0	0	0	0	0	93	25	0	0	0	9	0	36
CTC_RS10845	complement(2246448..2246711)	0	0	101	14	0	33	0	0	0	0	0	0	0	0	0	0	0	0	0	0	0	0	0	0	0	0	0	78	139	182
CTC_RS10850	complement(2246729..2246962)	0	0	0	0	0	0	0	0	0	0	0	0	0	0	0	0	0	0	0	0	0	0	0	0	0	0	0	0	0	0
CTC_RS10855	complement(2247035..2247310)	0	0	0	0	0	0	0	0	0	0	0	0	0	0	0	0	0	0	0	0	0	0	0	0	0	0	0	0	0	0
CTC_RS10860	complement(2247424..2248482)	0	0	0	7	584	545	0	0	0	0	0	0	0	0	0	0	0	0	0	0	0	0	0	0	0	0	0	0	0	0
CTC_RS10865	complement(2248485..2248862)	0	0	0	0	303	593	0	0	0	0	0	0	0	0	0	0	0	0	0	0	0	0	0	0	0	0	0	0	0	0
CTC_RS10870	complement(2248862..2249521)	0	0	0	12	451	340	0	0	0	0	0	0	0	0	0	0	0	0	0	0	0	0	0	0	0	0	0	0	0	0

CTC_RS10875	complement(2249525..2250172)	0	0	0	0	0	0	0	0	0	0	0	0	0	0	0	0	0	0	0	0	0	0	0	0	0	0	0	0	0	0
CTC_RS14900	complement(2250183..2251433)	0	0	0	9	257	434	0	0	0	0	0	0	0	0	0	0	0	0	0	0	0	0	0	0	0	0	0	0	0	0
CTC_RS10885	complement(2251430..2252491)	0	0	0	0	561	682	0	0	0	0	0	0	0	0	0	0	0	0	0	0	0	0	0	0	0	0	0	0	0	0
CTC_RS10890	complement(2252484..2252924)	0	0	0	0	442	469	0	0	0	0	0	0	0	0	0	0	0	0	0	0	0	0	0	0	0	0	0	0	0	0
CTC_RS10895	complement(2252927..2253142)	0	0	0	0	106	239	0	0	0	0	0	0	0	0	0	0	0	0	0	0	0	0	0	0	0	0	0	0	0	0
CTC_RS10900	complement(2253168..2254262)	0	0	0	0	502	590	0	0	0	0	0	0	0	0	0	0	0	0	0	0	0	0	0	0	0	0	0	0	0	0
CTC_RS10905	complement(2254259..2254678)	0	0	0	36	1310	1765	0	0	0	0	0	0	0	0	0	0	0	0	0	0	0	0	0	0	0	0	0	0	0	0
CTC_RS10910	complement(2254724..2257306)	0	0	0	37	648	971	0	0	0	0	0	0	0	0	0	0	0	0	0	0	0	0	0	0	0	0	0	0	0	0
CTC_RS10915	complement(2257360..2257737)	0	0	0	0	0	0	0	0	0	0	0	0	0	0	0	0	0	0	0	0	0	0	0	0	0	0	0	0	0	0
CTC_RS10920	complement(2257924..2258334)	0	0	0	0	920	965	11	0	0	0	0	0	0	0	0	0	0	0	10	0	0	0	0	0	0	0	0	0	0	0
CTC_RS14780	complement(2258336..2258488)	0	0	0	0	300	113	0	0	0	0	0	0	0	0	0	0	0	0	0	0	0	0	0	0	0	0	0	0	0	0
CTC_RS10925	complement(2258513..2258944)	0	0	0	18	1433	2254	0	0	0	0	0	0	0	0	0	0	0	0	0	0	0	0	0	0	0	0	0	0	0	0
CTC_RS10930	complement(2258964..2260025)	0	0	0	7	971	860	0	0	0	0	0	0	0	0	0	0	0	0	0	0	0	0	0	0	0	0	0	0	0	0
CTC_RS10935	complement(2260030..2260440)	0	0	0	37	613	1510	0	0	0	0	0	0	0	0	0	0	0	0	0	0	0	0	0	0	0	0	0	0	0	0
CTC_RS10940	complement(2260509..2260739)	0	0	0	0	99	448	0	0	0	0	0	0	0	0	0	0	0	0	0	0	0	0	0	0	0	0	0	0	0	0
CTC_RS10945	complement(2260748..2261179)	0	0	0	18	584	838	0	0	0	0	0	0	0	0	0	0	0	0	0	0	0	0	0	0	0	0	0	0	0	0
CTC_RS10950	complement(2261154..2261513)	0	0	0	0	414	1436	0	0	0	0	0	0	0	0	0	0	0	0	0	0	0	0	0	0	0	0	0	0	0	0
CTC_RS10955	complement(2261522..2261800)	0	0	0	0	863	556	0	0	0	0	0	0	0	0	0	0	0	0	0	0	0	0	0	0	0	0	0	0	0	0
CTC_RS14890	complement(2261809..2261946)	0	0	0	0	0	0	0	0	0	0	0	0	0	0	0	0	0	0	0	0	0	0	0	0	0	0	0	0	0	0
CTC_RS10960	complement(2261989..2263095)	0	0	0	0	0	0	0	0	0	0	0	0	0	0	0	0	0	0	0	0	0	0	0	0	0	0	0	0	0	0
CTC_RS10965	complement(2263127..2263807)	0	0	0	0	135	152	0	0	0	0	0	0	0	0	0	0	0	0	0	0	0	9	0	0	0	0	0	0	0	0
CTC_RS10970	complement(2263804..2265036)	0	0	0	0	0	0	0	0	0	0	0	0	0	0	0	0	0	0	0	0	0	0	0	0	0	0	0	0	0	0
CTC_RS10975	complement(2265048..2266739)	0	0	0	0	0	0	0	0	0	0	0	0	0	0	0	0	0	0	0	0	0	7	0	0	0	0	0	0	0	0
CTC_RS10980	complement(2266736..2267197)	0	0	0	0	0	0	0	0	0	0	0	0	0	0	0	0	0	0	0	0	0	0	0	88	0	0	0	0	0	0
CTC_RS10985	complement(2267273..2267701)	0	0	0	0	0	0	0	0	0	0	0	0	0	0	0	0	0	0	0	0	0	0	0	0	0	0	0	0	0	0
CTC_RS10990	2267823..2268005	0	0	0	0	0	0	0	0	0	0	0	0	0	0	0	0	0	0	0	0	0	0	0	0	0	0	0	0	0	0
CTC_RS10995	2268063..2268470	0	0	0	0	0	0	0	0	0	0	0	0	0	0	26	0	0	0	0	0	0	0	0	0	0	0	0	0	0	0
CTC_RS11000	complement(2268846..2269088)	0	0	0	0	0	0	0	0	0	0	0	0	59	0	0	0	0	0	0	0	0	0	0	0	0	46	191	0	0	0
CTC_RS14785	complement(2269452..2269604)	0	0	0	0	0	0	0	0	0	0	0	0	0	96	0	0	0	0	0	0	0	0	0	0	0	73	0	0	0	0
CTC_RS11005	complement(2269616..2270131)	0	0	0	0	0	0	0	0	0	0	0	0	98	397	1037	0	0	0	0	0	0	0	0	0	46	455	674	0	0	0
CTC_RS11010	complement(2270156..2270380)	0	0	0	0	0	0	0	0	0	0	0	0	0	0	0	0	0	0	0	0	0	0	0	0	0	0	0	0	0	0
CTC_RS14790	complement(2270377..2270517)	0	0	0	0	0	0	0	0	0	0	0	0	0	0	0	0	0	0	0	0	0	0	0	0	0	0	0	0	0	0
CTC_RS11015	complement(2270520..2270909)	0	0	0	0	0	0	0	0	0	0	0	0	0	0	0	0	0	0	0	0	0	0	0	0	0	0	0	0	0	0
CTC_RS11020	complement(2271016..2271378)	0	0	0	0	0	0	0	0	0	0	0	0	0	0	0	0	0	0	0	0	0	0	0	0	0	0	0	0	0	0
CTC_RS11025	complement(2271462..2272178)	0	0	0	0	0	0	0	0	0	0	0	0	0	0	0	0	0	0	0	0	0	0	0	0	0	0	0	0	0	0
CTC_RS11030	complement(2272202..2272585)	0	0	0	0	0	0	0	0	0	0	0	0	0	0	0	0	0	0	0	0	0	0	0	0	0	0	0	0	0	0
CTC_RS11035	complement(2272722..2273129)	0	0	0	0	0	0	0	116	0	0	0	0	124	287	51	0	0	0	0	0	0	0	0	0	19	301	0	0	0	0
CTC_RS11040	complement(2273142..2273396)	0	0	0	0	0	0	0	0	0	0	0	0	28	86	82	0	0	0	0	0	0	0	0	0	31	88	45	0	0	0
CTC_RS11045	complement(2273410..2274306)	8	40	0	259	64	19	0	0	0	0	0	0	0	0	0	0	0	0	0	0	0	311	62	0	0	0	0	0	55	0
CTC_RS11050	complement(2274324..2274503)	75	0	0	1102	127	0	0	0	0	0	0	0	0	0	0	0	0	70	0	0	0	620	207	0	0	0	0	0	0	0
CTC_RS11055	complement(2274496..2275302)	519	162	33	3381	852	214	0	0	0	0	0	0	0	0	0	30	31	16	0	0	0	4036	1064	126	0	0	0	0	0	0
CTC_RS11060	complement(2275304..2276224)	110	0	0	389	124	66	0	0	0	0	0	0	0	0	0	0	55	14	2	0	0	357	41	0	0	0	0	0	0	0
CTC_RS11065	complement(2276217..2276648)	266	0	0	1881	212	40	0	0	0	0	0	0	0	0	0	0	0	0	0	0	0	2212	691	0	0	0	0	0	0	0
CTC_RS11070	complement(2276733..2276996)	0	0	0	0	0	0	0	119	0	0	0	0	0	0	0	0	0	0	0	0	0	0	0	0	0	0	0	0	279	0
CTC_RS14795	complement(2276990..2277145)	130	76	0	440	73	0	0	0	0	0	0	0	0	0	134	0	0	0	0	0	0	557	0	0	0	0	0	0	0	0
CTC_RS11075	complement(2277253..2277624)	0	0	0	0	0	0	0	0	0	0	0	0	0	0	0	0	0	0	0	0	0	0	0	0	0	0	0	0	0	0
CTC_RS11080	complement(2277629..2277841)	0	0	0	0	0	0	0	0	0	0	0	0	0	0	0	0	0	0	0	0	0	0	0	0	0	0	0	0	0	0
CTC_RS11085	complement(2277927..2278676)	0	0	0	0	0	0	0	0	0	0	0	0	0	0	0	0	0	0	0	0	0	0	0	0	0	0	0	0	0	0
CTC_RS14800	complement(2278843..2279016)	0	0	0	0	0	0	0	0	0	0	0	0	0	0	0	0	0	0	0	0	0	0	0	0	0	0	0	0	0	0
CTC_RS11090	complement(2279027..2279242)	0	0	0	0	0	0	0	0	0	0	0	0	0	0	0	0	0	0	0	0	0	0	0	0	0	0	0	0	0	0
CTC_RS11095	2279479..2279880	0	0	0	0	0	0	0	0	0	0	0	0	0	0	0	0	0	0	0	0	0	0	0	0	0	0	0	0	0	0
CTC_RS11100	2279903..2281387	0	0	0	0	0	0	0	0	0	0	0	0	0	0	0	0	0	0	0	0	0	0	0	0	0	0	0	0	0	0
CTC_RS11105	complement(2281472..2282092)	109	76	161	172	74	56	45	76	86	57	181	77	76	0	0	99	203	82	82	0	154	80	120	66	0	0	0	66	158	185
CTC_RS11110	complement(2282112..2283677)	69	91	85	107	88	121	66	101	85	221	144	137	46	47	54	139	48	178	122	124	137	55	48	182	61	50	30	114	141	86
CTC_RS11115	complement(2283729..2285648)	7	12	7	0	24	36	0	0	0	9	8	0	0	8	0	4	0	7	7	0	12	0	0	21	4	0	0	7	13	0
CTC_RS11120	complement(2285554..2286330)	0	0	34	39	30	22	36	0	0	8	0	61	0	0	0	9	32	33	11	0	0	16	144	52	0	29	0	0	0	0
CTC_RS11125	complement(2286391..2286957)	0	0	0	0	0	0	0	0	0	0	28	0	0	0	37	0	0	0	0	0	0	0	0	0	14	0	0	0	0	0
nagZ	2287150..2288367	11	0	55	19	0	28	0	26	22	70	0	0	6	12	17	6	21	10	17	0	59	31	31	33	0	28	0	11	0	47
CTC_RS11135	2288432..2288644	0	56	62	36	108	0	44	0	251	42	226	112	34	137	98	0	0	119	200	227	337	58	0	191	37	52	109	32	0	90
CTC_RS11140	2288796..2289107	0	0	0	0	0	0	0	0	0	0	52	0	23	0	0	22	0	41	0	0	0	0	0	0	0	0	0	0	0	0
CTC_RS11145	complement(2289135..2290328)	0	0	0	0	0	14	0	13	0	25	0	20	6	0	18	6	0	0	11	41	40	0	0	0	7	0	0	12	0	0
CTC_RS11150	2290499..2290882	0	1328	1212	0	686	224	0	1476	1809	0	2930	1057	0	3126	1202	0	920	1517	11	126	249	0	1166	530	0	2969	1449	0	4378	998
CTC_RS11155	2290959..2291240	0	0	189	0	163	61	0	56	95	0	627	592	0	208	0	0	89	0	0	343	85	0	132	289	0	79	164	0	0	0
CTC_RS11160	complement(2291297..2292139)	16	28	32	36	27	61	11	37	0	7	0	85	43	69	25	16	30	60	30	0	0	15	0	0	9	0	55	8	87	45
CTC_RS11165	complement(2292317..2292781)	0	51	0	0	0	37	0	0	0	0	35	0	16	0	0	0	0	0	0	104	0	0	0	0	17	0	0	0	0	0
eutH	complement(2292796..2293887)	0	22	0	0	42	32	9	14	24	5	29	44	0	13	0	0	0	12	0	0	44	0	0	37	0	31	0	0	0	0
CTC_RS11175	complement(2293929..2294471)	0	0	0	0	0	0	0	0	0	0	30	0	0	0	0	0	46	0	0	0	0	0	0	0	0	0	0	0	0	0
CTC_RS11180	complement(2294495..2294764)	0	0	0	0	0	0	0	0	0	0	0	0	0	0	0	0	93	0	0	0	0	0	0	0	29	0	0	0	0	0
CTC_RS11185	complement(2294797..2295543)	0	32	18	0	0	0	0	0	0	0	22	0	0	0	0	0	0	0	0	0	0	0	0	0	0	0	0	0	0	0
eutJ_2	complement(2295530..2296366)	0	0	0	0	0	21	11	0	0	21	19	29	0	0	0	8	30	15	0	0	29	0	89	49	9	0	0	16	0	23
CTC_RS11195	complement(2296378..2297019)	0	0	124	0	0	0	0	25	0	0	0	37	11	23	33	0	0	20	7	0	37	19	58	0	0	0	36	11	0	30
CTC_RS11200	complement(2297032..2297793)	0	0	17	10	30	0	0	0	0	0	42	94	0	0	0	0	33	17	0	0	0	0	0	0	0	0	0	0	0	0
eutM_2	complement(2297895..2298179)	0	0	47	0	0	0	33	0	0	0	0	84	0	0	0	0	0	0	0	0	0	0	0	0	0	0	0	0	172	0
CTC_RS11210	complement(2298235..2299707)	0	16	36	0	31	12	0	11	0	12	22	49	5	0	14	9	51	34	0	0	8	0	25	0	0	23	63	5	33	39
CTC_RS11215	complement(2299722..2300255)	0	0	0	0	0	32	0	0	75	0	60	45	0	27	0	0	0	0	0	0	0	0	0	0	0	0	0	0	0	0
eutL	complement(2300270..2300926)	0	36	30	0	35	52	0	0	20	27	24	36	0	67	96	0	77	19	6	0	0	0	0	0	0	17	35	10	0	29
eutC	complement(2300944..2301828)	0	0	23	0	0	19	0	0	0	13	36	27	0	0	47	0	57	14	0	0	0	0	0	0	0	13	0	0	0	0
CTC_RS11230	complement(2301843..2303210)	0	35	0	0	67	13	0	0	0	17	0	0	5	0	77	0	37	19	3	0	35	9	0	30	0	8	17	10	0	14
eutA	complement(2303249..2304682)	0	0	9	0	0	0	0	0	0	4	0	0	0	0	0	5	35	35	0	0	0	0	0	28	0	8	0	0	0	0
CTC_RS11240	complement(2304838..2306253)	67	84	85	92	210	243	40	56	189	94	216	202	51	41	104	125	89	107	57	68	253	61	79	115	39	47	98	102	17	108

CTC_RS11245	complement(2306270..2306848)	23	164	115	145	277	238	258	177	92	112	167	330	199	152	181	271	87	241	206	334	248	161	129	211	137	193	300	119	212	199
CTC_RS11250	complement(2306922..2307356)	0	0	0	0	158	79	0	36	0	54	0	0	0	0	0	0	0	0	0	0	0	0	0	94	0	26	0	16	0	0
CTC_RS11255	complement(2307367..2307711)	0	0	0	22	332	50	0	274	0	51	140	0	0	85	61	20	0	110	0	0	0	36	0	0	0	32	0	0	142	56
CTC_RS11260	complement(2307908..2309077)	12	0	34	7	39	0	0	0	0	15	0	41	6	0	18	0	0	22	4	0	0	0	32	0	0	0	0	6	0	0
CTC_RS11265	complement(2309254..2310060)	100	118	165	113	426	214	150	429	199	293	259	296	161	163	182	160	94	204	332	240	148	231	277	353	108	139	57	145	213	380
CTC_RS11270	2310305..2311417	24	21	12	27	0	46	0	14	24	56	43	43	6	26	0	12	45	23	11	0	0	33	0	0	0	40	31	56	44	121
CTC_RS11275	complement(2311504..2311908)	133	59	99	94	226	43	46	78	132	87	40	0	53	108	104	135	0	0	21	239	0	31	0	201	39	83	114	68	182	47
CTC_RS11280	complement(2311942..2312577)	64	0	63	12	72	0	29	25	42	9	0	0	11	23	33	32	119	119	40	0	38	20	0	128	25	53	36	108	39	30
CTC_RS11285	complement(2312598..2313269)	20	71	0	0	0	0	14	0	0	9	0	0	11	0	0	0	0	19	19	0	0	0	0	0	24	0	0	20	0	0
CTC_RS11290	complement(2313312..2313923)	0	0	0	12	0	0	61	77	0	48	0	0	47	24	0	78	0	83	28	0	39	20	0	0	0	0	76	45	40	94
CTC_RS11295	2314063..2315007	0	577	858	0	97	73	0	566	353	0	119	51	8	558	333	0	868	563	0	51	25	0	79	129	0	568	515	0	1195	284
CTC_RS11300	complement(2315042..2315980)	36	76	227	89	269	202	30	184	171	75	240	203	69	109	134	36	107	121	59	103	76	79	278	173	42	60	99	7	78	41
CTC_RS11305	complement(2316134..2316523)	69	365	205	59	294	44	48	121	0	45	453	367	139	263	108	87	65	97	158	248	184	64	191	0	61	258	178	35	189	147
brnQ_3	2316634..2317929	42	37	113	29	88	146	36	36	41	360	50	18	22	11	65	68	0	127	43	0	148	19	0	94	18	17	72	58	19	89
aspS	complement(2317995..2319773)	372	240	179	377	296	184	252	283	165	345	312	362	260	156	106	415	369	174	400	163	282	244	231	137	348	163	130	379	248	259
CTC_RS11320	complement(2319825..2321246)	38	33	37	72	129	121	79	89	0	96	11	67	51	51	59	48	0	107	99	34	177	87	52	57	72	24	65	58	104	67
CTC_RS11325	complement(2321283..2321888)	45	39	66	189	76	142	262	52	44	93	80	197	155	97	104	225	125	63	207	0	79	102	0	202	65	37	0	91	0	0
dtd	complement(2321899..2322348)	90	53	59	85	102	77	311	35	119	66	0	106	112	163	0	121	56	56	250	107	53	28	0	0	106	99	103	107	55	85
CTC_RS11335	complement(2322360..2324555)	86	70	91	87	84	133	119	65	61	143	88	120	59	73	86	81	161	156	131	132	109	68	85	93	105	71	84	103	101	79
CTC_RS11340	complement(2324667..2325185)	26	46	102	191	88	133	36	30	0	296	93	92	70	28	40	66	49	146	106	186	0	48	0	157	15	43	134	272	95	111
CTC_RS11345	complement(2325235..2326044)	17	0	0	9	28	21	23	0	0	131	0	0	18	0	0	17	0	31	37	0	0	0	46	0	0	14	0	51	0	0
secF	complement(2326223..2327098)	46	54	76	17	52	295	43	18	91	142	55	109	41	67	24	39	29	58	39	28	191	57	85	186	54	51	53	118	56	77
secD	complement(2327099..2328364)	192	300	200	193	91	170	236	236	211	336	178	264	182	335	348	237	458	350	262	153	472	137	147	161	182	150	348	383	291	326
scfB	complement(2328416..2329777)	159	87	88	129	118	202	188	185	78	182	112	88	191	193	123	285	296	363	307	107	325	128	110	239	157	172	170	232	126	260
scfA	complement(2329862..2329999)	0	0	0	0	0	0	0	0	0	0	0	0	0	0	0	0	0	0	0	0	173	0	0	0	0	0	0	0	0	0
CTC_RS11370	complement(2330250..2330615)	0	65	73	21	0	0	0	43	0	16	483	0	0	320	287	19	483	69	0	132	65	0	0	0	0	31	0	0	134	0
yajC	complement(2330676..2331053)	501	941	704	525	910	1505	420	1166	1201	930	1573	1263	439	407	832	865	400	670	450	128	759	558	691	1185	378	473	491	400	195	1014
tgt	complement(2331138..2332268)	24	63	47	61	61	137	58	56	0	146	142	21	32	39	37	84	67	106	139	0	85	11	99	216	63	109	102	103	109	203
queA	complement(2332315..2333340)	13	23	52	52	45	50	36	15	26	63	94	47	21	14	20	40	25	136	83	47	140	24	0	40	23	22	0	47	48	112
ruvB	complement(2333375..2334403)	13	23	13	37	45	17	27	61	78	17	31	46	28	28	20	26	0	123	50	0	70	36	0	40	4	11	45	47	95	112
ruvA	complement(2334417..2335010)	23	0	0	0	0	87	31	53	45	20	27	0	0	74	35	34	42	43	36	0	201	21	0	0	13	19	0	93	0	258
CTC_RS11400	2335144..2335761	0	0	22	0	0	0	0	0	0	0	156	0	0	190	34	0	41	0	0	0	39	0	0	0	0	54	75	0	159	31
CTC_RS11405	complement(2335843..2336583)	164	416	144	309	124	116	252	255	108	239	152	225	156	178	255	340	34	205	333	65	161	117	151	165	112	136	63	380	33	155
CTC_RS11410	complement(2336706..2337350)	0	37	62	12	36	0	29	73	41	18	0	37	45	23	33	48	0	118	26	0	74	38	0	63	25	52	0	32	38	0
CTC_RS11415	complement(2337355..2338032)	20	70	20	0	34	0	0	23	0	17	0	0	0	0	0	20	0	19	19	71	106	0	0	0	0	0	34	0	0	57
CTC_RS11420	complement(2338040..2339449)	10	0	19	16	81	24	13	11	0	17	23	17	10	0	15	14	0	36	6	0	85	18	53	0	6	0	33	10	35	0
CTC_RS11425	complement(2339519..2340415)	75	53	59	102	26	135	31	70	0	273	143	80	32	82	70	106	28	71	194	54	27	69	125	45	40	75	52	107	82	128
CTC_RS11430	complement(2340797..2341018)	0	0	0	0	0	0	0	0	0	0	0	0	0	0	0	0	0	0	0	0	0	0	0	0	0	0	0	0	0	0
CTC_RS11435	complement(2341118..2344552)	20	110	205	36	214	191	35	137	179	120	140	236	28	273	269	107	73	317	407	28	125	25	206	320	12	169	243	177	29	391
CTC_RS14610	complement(2344872..2345033)	83	293	0	141	0	106	58	389	165	0	496	0	223	90	388	210	156	156	52	597	0	153	461	0	98	138	429	0	455	355
CTC_RS11440	complement(2345138..2346427)	94	202	206	124	89	361	116	159	124	211	224	278	112	113	179	195	137	167	246	262	260	135	145	252	71	199	126	133	95	297
hflX	complement(2346446..2348227)	80	53	67	34	39	97	126	18	75	94	180	107	53	99	24	118	71	185	92	27	40	70	42	23	45	44	0	89	83	86
CTC_RS11450	complement(2348329..2349138)	0	0	33	9	0	64	35	39	0	22	0	0	9	0	0	34	62	47	21	0	59	0	0	0	20	14	0	0	30	47
CTC_RS11455	complement(2349174..2349833)	164	108	40	35	69	104	71	48	0	81	97	72	98	44	32	62	76	96	213	147	0	38	0	123	66	17	70	125	0	29
CTC_RS11460	2350016..2351665	8	0	0	0	42	10	0	0	0	14	19	0	4	0	0	4	15	15	8	0	14	0	0	0	0	7	0	8	0	0
CTC_RS11465	2351704..2352798	197	65	36	105	0	110	136	29	98	178	117	65	99	0	0	68	23	81	70	88	109	215	68	186	159	20	127	69	45	88
CTC_RS11470	complement(2352864..2353475)	927	581	695	885	768	1577	1136	463	611	1259	1103	1248	1097	454	137	1135	1319	993	799	711	704	1206	976	2195	842	219	455	1258	521	626
CTC_RS11475	complement(2353501..2354079)	1564	696	264	1160	831	714	2659	992	830	683	1041	742	1789	708	543	1629	2048	962	1663	835	868	1457	677	1125	1548	637	481	1294	1060	397
CTC_RS11480	complement(2354121..2354702)	650	489	274	590	473	711	1138	622	229	558	414	492	943	50	108	644	520	239	515	582	432	597	898	665	763	346	40	484	380	198
CTC_RS11485	complement(2354847..2355662)	17	29	33	19	56	21	11	19	65	29	39	0	27	0	26	42	0	124	36	0	59	0	0	50	19	0	0	34	0	23
CTC_RS11490	complement(2355662..2356774)	0	64	12	75	185	139	59	57	48	69	29	21	19	13	75	58	0	34	107	0	86	22	34	183	14	30	21	49	44	34
CTC_RS11495	complement(2356828..2358171)	10	35	20	28	51	64	35	12	40	31	12	18	16	11	0	41	56	94	35	0	18	9	28	61	12	0	9	61	0	14
CTC_RS11500	complement(2358326..2359498)	12	20	34	52	20	15	56	0	0	65	27	41	6	12	72	41	0	76	91	0	20	32	95	121	14	10	20	70	21	57
CTC_RS11505	complement(2359636..2360670)	91	69	128	59	199	316	9	91	103	414	124	69	0	28	61	33	24	98	74	93	277	60	108	551	23	32	90	126	71	185
hpt_2	2361133..2361657	206	0	76	116	87	131	213	90	0	349	31	0	344	56	40	298	48	241	154	92	137	118	142	155	166	85	132	314	47	110
yunB	complement(2361733..2362356)	0	0	0	0	0	0	0	25	0	0	103	0	0	0	0	0	0	20	0	0	0	0	60	0	0	36	37	0	39	0
CTC_RS11520	2362578..2364950	262	165	174	365	493	327	275	212	169	825	193	292	207	185	115	373	149	293	317	163	514	261	315	326	160	198	195	380	165	388
spoVAE	complement(2364987..2365346)	0	132	74	21	0	0	26	175	223	0	268	66	0	488	175	0	0	70	0	0	0	0	0	0	0	248	64	0	136	53
spoVAD	complement(2365353..2366375)	0	46	78	37	179	51	18	215	26	23	236	117	0	286	51	40	123	37	17	47	23	0	146	0	23	284	204	13	120	131
spoVAC	complement(2366408..2366866)	0	52	29	17	100	75	0	103	0	0	105	104	0	191	0	0	0	0	19	105	104	0	0	89	0	170	0	0	53	0
sigF	complement(2366980..2367738)	285	344	517	261	574	295	234	498	581	125	593	755	247	579	912	556	332	701	387	191	425	327	344	429	345	692	703	100	194	985
spoIIAB	complement(2367753..2368178)	63	223	219	233	969	162	109	739	439	42	641	392	152	550	739	288	237	594	349	227	505	175	263	191	74	630	980	0	173	1080
spoIIAA	complement(2368194..2368529)	40	212	40	136	409	308	222	468	636	53	431	213	215	305	375	284	225	226	63	288	0	258	111	121	47	366	414	41	0	228
speE	2368798..2369643	272	28	79	149	217	122	265	93	379	450	133	197	256	87	149	209	30	224	153	0	198	139	353	265	239	92	192	431	145	351
CTC_RS11560	complement(2369882..2371624)	8	0	0	4	13	10	0	9	31	3	9	0	8	8	12	0	0	7	7	0	0	0	0	0	0	13	27	4	0	0
pdaA	2371804..2372640	65	255	207	55	55	288	33	113	176	92	173	228	26	184	175	28	90	106	41	58	200	74	45	243	62	174	180	90	117	218
CTC_RS11570	complement(2372664..2373386)	0	66	0	21	0	0	13	44	0	65	0	0	10	20	0	0	35	18	35	0	0	0	0	0	11	0	32	10	0	53
CTC_RS11575	complement(2373401..2374054)	0	73	20	23	35	79	0	0	82	81	0	36	0	0	0	31	39	58	78	0	37	19	0	62	18	85	0	11	75	88
CTC_RS11580	complement(2374069..2375484)	0	17	9	16	0	12	7	0	0	75	0	0	0	0	0	0	0	45	9	34	17	18	26	29	0	0	16	19	0	41
murJ_1	complement(2375595..2377151)	0	0	0	10	29	22	0	20	17	64	10	8	0	0	0	0	0	0	5	0	0	8	0	0	0	0	0	13	0	18
CTC_RS11590	complement(2377151..2378263)	0	0	0	7	0	31	8	0	0	48	0	0	19	0	0	0	0	0	42	0	0	0	0	0	0	0	0	19	0	0
CTC_RS11595	complement(2378268..2379512)	11	19	21	31	55	55	0	0	0	71	0	0	17	0	17	11	0	20	10	0	19	20	30	33	19	9	37	33	0	62
CTC_RS11600	complement(2379611..2380642)	13	46	13	52	22	50	0	122	78	86	0	23	14	14	41	20	0	37	16	0	23	12	0	0	15	11	22	13	0	74
CTC_RS11605	complement(2380639..2382078)	47	33	9	16	32	60	0	0	0	37	0	17	10	0	7	19	0	18	21	0	0	0	0	0	6	8	0	19	17	0
CTC_RS11610	complement(2382111..2383457)	0	35	10	6	17	26	0	12	0	39	24	0	21	0	31	10	19	38	13	0	18	0	28	30	0	8	17	31	0	0
murJ_2	complement(2383473..2385017)	0	31	17	40	45	78	6	41	0	40	10	0	19	9	14	9	16	33	44	0	46	16	24	26	10	36	0	18	0	50
CTC_RS11620	complement(2385035..2386141)	0	107	60	131	166	140	25	100	145	112	29	65	78	159	0	68	46	137	92	87	43	78	0	37	36	61	42	81	67	173
CTC_RS11625	complement(2386178..2386918)	55	192	197	185	155	70	88	276	108	255	152	225	97	99	368	257	409	513	275	196	258	184	353	220	139	196	156	213	199	285
CTC_RS11630	complement(2386985..2388091)	134	150	198	290	166	156	143	199	181	341	174	474	241	212	171	240	217	274	419	218	378	224	472	74	154	268	272	236	177	294
CTC_RS11635	complement(2388149..2389465)	698	360	323	582	522	294	595	430	152	1022	98	526	512	356	80	491	268	461	588	184	472	372	680	371	536	263	141	564	503	626

CTC_RS11640	complement(2389559..2390764)	0	20	11	25	0	43	15	52	22	127	0	0	30	73	0	56	21	63	28	40	79	31	31	34	36	46	38	154	0	79
CTC_RS11645	complement(2390784..2392625)	7	103	22	37	50	94	15	26	36	77	52	26	24	24	80	63	27	62	60	0	91	7	61	22	4	55	13	78	27	73
galU	complement(2392753..2393634)	15	108	75	52	208	59	32	196	91	117	109	162	41	315	119	77	86	158	133	110	27	56	169	0	54	228	105	125	195	250
CTC_RS11655	complement(2393727..2394809)	0	44	12	28	0	16	60	0	0	44	15	22	7	0	58	38	47	23	27	0	22	0	0	38	0	41	0	38	0	0
CTC_RS11660	2395002..2396462	37	97	55	42	16	12	6	75	18	174	121	33	30	110	43	65	69	121	81	33	33	51	51	0	49	31	63	89	17	79
CTC_RS11665	complement(2396498..2396959)	29	103	29	83	99	37	40	102	58	51	0	52	94	127	182	15	109	55	110	0	0	81	0	88	0	97	251	119	106	41
CTC_RS11670	complement(2396980..2399340)	29	40	56	42	97	66	43	67	34	145	109	81	9	56	36	40	32	80	76	205	111	32	32	103	24	76	29	82	42	49
CTC_RS11675	complement(2399361..2401724)	17	0	0	6	10	15	24	0	0	50	20	20	0	12	9	19	21	32	47	0	61	0	16	52	18	19	10	35	10	8
zapA	complement(2401792..2402451)	195	252	484	220	590	548	85	358	364	523	438	217	77	266	222	103	306	556	129	293	689	150	170	370	108	102	176	177	409	494
pheT	complement(2402563..2404938)	159	50	17	96	77	0	149	20	11	159	7	50	185	31	18	195	32	59	188	20	50	115	63	0	187	66	10	127	21	32
pheS	complement(2404962..2405981)	106	70	13	67	22	34	155	46	26	197	32	23	191	29	62	107	0	25	213	47	23	85	0	0	163	88	0	98	24	94
CTC_RS11695	complement(2406303..2407085)	17	0	51	39	59	44	6	40	0	181	41	61	28	19	27	52	0	32	38	0	31	32	48	0	10	29	0	70	31	49
CTC_RS11700	complement(2407100..2407762)	61	179	80	150	104	0	84	95	121	249	0	180	163	132	63	113	76	115	298	146	108	19	56	61	72	135	70	135	74	202
CTC_RS11705	complement(2407777..2409123)	40	0	49	40	85	13	28	58	59	175	60	71	64	11	0	86	0	85	69	36	71	18	111	30	29	46	34	79	18	71
rplT	complement(2409276..2409635)	413	264	554	572	573	239	233	525	297	1271	446	332	581	366	408	719	561	211	1512	1478	465	517	933	679	639	342	193	1623	341	426
rpmI	complement(2409662..2409859)	614	359	134	540	463	174	283	318	270	1178	649	603	255	518	106	791	255	384	966	366	121	752	0	0	481	621	351	1910	124	581
infC	complement(2409898..2410392)	1283	1150	860	2243	1158	871	688	1304	1241	5870	1007	1157	1334	1124	1271	2257	663	1522	3951	1173	2175	2281	2036	1069	1162	1242	1124	6673	744	1665
thrS	complement(2410601..2412520)	422	741	637	338	657	269	377	1246	473	784	335	180	444	968	655	516	696	1210	880	454	343	475	641	339	310	1080	483	569	741	784
ytxC	complement(2412853..2413743)	0	53	104	0	26	58	0	18	60	7	72	54	0	33	47	0	170	156	0	0	0	0	42	0	0	75	156	0	55	108
CTC_RS11735	2413847..2414548	0	0	0	0	33	0	0	0	0	50	0	0	0	0	0	10	36	0	12	0	0	0	0	0	0	16	33	29	0	0
cobT	complement(2415139..2416215)	50	88	49	50	0	0	17	29	0	55	0	44	40	41	58	101	47	59	182	0	0	23	35	0	66	42	22	109	23	36
hslO	complement(2416712..2417596)	0	80	105	34	26	39	200	53	0	147	73	0	114	165	119	162	57	86	283	0	54	70	0	0	54	114	79	186	28	43
CTC_RS11765	complement(2417615..2418355)	18	0	0	41	0	23	13	21	0	68	65	0	10	59	28	37	0	34	52	0	32	17	101	0	0	0	31	9	0	52
CTC_RS11770	complement(2418392..2418646)	0	0	0	0	0	0	0	0	0	0	0	94	0	0	0	0	0	0	0	0	0	0	0	0	0	0	0	0	0	0
CTC_RS11775	2418875..2419867	735	645	536	661	831	851	944	555	336	651	534	361	665	531	338	645	330	752	366	609	1108	937	620	615	639	433	479	194	297	637
dapA	2419894..2420778	366	429	316	418	220	536	427	231	407	541	590	674	285	182	95	593	413	572	199	273	716	554	337	460	332	177	183	225	361	1105
dapB	2420808..2421560	323	221	494	471	365	778	601	690	284	463	576	634	379	214	111	529	620	572	243	193	890	560	496	487	416	304	277	155	652	738
CTC_RS11790	complement(2421607..2422251)	0	110	41	0	71	0	0	73	0	0	349	0	0	227	65	11	235	20	0	0	0	0	58	0	0	173	36	0	57	119
CTC_RS14345	complement(2422336..2422824)	0	0	0	0	35	0	0	0	0	0	131	49	15	60	86	0	52	0	0	0	0	0	76	0	0	0	0	0	0	78
pdaB	complement(2422893..2423639)	0	556	276	0	153	69	0	780	179	16	710	0	10	803	197	0	844	220	0	65	0	0	100	0	0	1085	217	0	986	26
CTC_RS11805	2423799..2424323	26	0	51	0	87	98	36	0	0	112	61	45	14	0	0	26	48	24	8	0	91	0	71	155	0	0	88	26	0	0
CTC_RS11810	complement(2424315..2425196)	0	0	15	0	0	0	0	18	30	0	18	0	0	50	0	0	114	29	0	0	0	0	0	0	0	25	53	0	28	0
CTC_RS11815	2425323..2425565	0	293	274	314	377	142	0	389	440	170	198	98	119	422	432	56	0	677	297	199	98	255	154	335	131	0	572	141	0	0
CTC_RS11820	complement(2425617..2426912)	42	0	10	24	0	13	32	12	0	91	50	0	28	23	0	42	0	20	44	0	0	10	0	0	31	0	0	74	0	15
CTC_RS11825	complement(2426980..2429622)	445	72	25	196	35	20	284	36	10	445	43	45	304	33	32	152	29	62	172	55	0	286	71	15	399	42	53	242	19	58
CTC_RS11830	complement(2429994..2430611)	3958	652	646	3173	631	697	2325	255	346	4463	585	1081	3691	308	339	1356	490	594	1290	783	465	3845	785	1021	4414	470	863	2353	238	310
CTC_RS11835	complement(2430642..2432321)	6866	819	649	4733	559	1047	3740	557	397	7697	622	1094	5456	549	674	1707	420	539	2565	720	1111	6200	889	1115	6753	566	442	4170	453	867
ftcD	complement(2432370..2433263)	5797	624	446	3474	897	925	3308	511	299	5903	602	547	5696	491	282	1817	522	566	2799	541	589	6370	877	546	6138	600	467	4537	329	450
gltS_1	complement(2433428..2434657)	670	116	32	1334	130	294	1179	77	43	38	78	0	1473	71	68	72	123	72	733	39	272	1987	243	232	1380	100	75	162	40	94
CTC_RS11850	complement(2434892..2436079)	1564	659	728	270	540	537	1127	689	517	149	541	221	231	672	212	206	913	682	168	204	443	386	471	206	320	734	488	104	702	871
CTC_RS11855	complement(2436120..2437391)	1211	224	199	78	108	108	851	247	126	51	215	103	170	391	247	16	674	542	114	152	207	146	205	192	168	404	255	22	425	301
CTC_RS11860	complement(2437706..2439004)	62	18	10	12	0	27	7	0	0	214	99	37	6	28	0	5	19	19	20	0	18	24	0	0	55	0	0	148	19	0
CTC_RS11865	complement(2439405..2440703)	1815	1296	635	1013	1182	703	596	1018	720	775	3328	919	1053	1499	1187	1067	1476	663	666	1564	727	1122	1293	1285	1215	1187	1339	468	1171	826
CTC_RS11870	complement(2440735..2441526)	478	120	168	347	347	131	177	179	51	567	649	60	419	129	106	292	207	224	263	61	272	392	283	103	330	127	234	182	465	242
CTC_RS11875	2441951..2442715	0	31	0	70	0	113	24	0	70	224	42	0	19	0	82	71	0	99	119	63	94	0	0	160	0	0	30	54	32	25
CTC_RS11880	2442723..2443196	86	0	196	97	48	255	157	66	56	473	34	50	76	31	0	57	0	80	112	102	151	79	236	172	33	24	0	160	0	40
CTC_RS11885	complement(2443234..2444697)	74	16	36	5	31	59	6	11	0	93	110	16	35	10	14	23	34	26	16	33	49	8	25	28	81	46	32	70	0	26
CTC_RS11890	complement(2445103..2446566)	28	65	55	10	63	141	13	22	46	230	231	147	54	50	43	23	103	78	44	165	147	25	25	167	43	15	127	103	84	13
hutH	complement(2446671..2448215)	3101	215	344	267	223	257	411	194	207	2309	333	803	2651	237	570	2555	604	254	658	501	279	470	48	356	4712	217	488	1947	191	372
CTC_RS11900	complement(2448553..2451588)	58	344	223	60	234	565	77	135	158	130	289	432	56	116	124	70	258	198	99	223	425	25	357	449	44	144	92	92	376	221
hutI	complement(2451744..2453003)	826	471	644	224	655	780	81	425	488	1280	995	1515	338	593	616	284	881	312	334	653	778	192	741	1260	626	506	570	998	584	472
CTC_RS11910	complement(2453097..2455121)	2963	568	644	196	351	885	203	428	317	3821	571	1243	413	333	373	567	511	438	614	884	945	196	986	975	924	304	378	3697	364	360
CTC_RS11915	complement(2455316..2456671)	20	17	0	6	0	25	21	12	0	44	24	18	11	0	0	15	19	19	13	36	18	18	0	30	6	8	17	10	0	14
CTC_RS11920	complement(2456659..2458662)	0	0	0	4	0	0	7	0	0	18	0	0	4	0	0	0	38	9	6	0	0	6	19	0	8	11	0	3	0	0
gltS_2	2459100..2460311	3178	2593	5788	1823	5182	7602	1462	1578	5102	1238	9137	4441	1221	1739	3522	2546	5953	3552	1731	8461	9948	1904	4373	7457	1495	1084	4400	1512	4779	4167
CTC_RS11930	complement(2460348..2464643)	53	204	229	135	336	321	122	269	196	70	572	739	77	232	225	154	376	337	114	270	579	72	195	294	87	260	135	48	223	486
CTC_RS11935	complement(2464936..2465547)	66	39	130	150	225	197	91	26	44	77	223	78	71	48	34	56	41	103	35	158	235	101	122	67	26	73	0	67	120	125
CTC_RS11940	complement(2465547..2466929)	401	343	135	419	265	274	411	228	155	149	424	190	261	222	91	325	438	256	221	105	190	458	189	206	287	137	101	65	373	277
CTC_RS11945	complement(2466946..2468655)	253	180	82	286	282	202	366	156	62	73	85	126	228	205	49	235	280	215	122	85	140	218	87	24	269	144	108	68	301	179
CTC_RS11950	complement(2468733..2469326)	23	160	67	180	193	290	126	79	90	30	27	121	109	25	0	46	170	85	43	81	40	104	314	0	93	75	0	23	145	32
CTC_RS11955	complement(2469338..2469646)	219	0	0	123	74	279	121	102	43	0	52	77	140	142	0	66	0	82	14	0	77	0	0	132	77	36	0	44	79	0
CTC_RS11960	complement(2469683..2470108)	349	724	468	555	969	627	657	351	376	236	377	448	390	241	98	336	415	208	140	341	505	568	263	573	335	210	218	81	346	630
CTC_RS11965	complement(2470134..2472101)	213	157	209	326	396	420	256	96	143	135	294	158	117	89	117	170	167	122	72	197	225	265	209	310	113	136	141	66	299	244
CTC_RS11970	complement(2472113..2473162)	90	23	76	145	142	82	98	30	25	73	107	136	96	56	40	71	72	84	36	92	68	83	142	78	60	43	66	65	94	110
CTC_RS11975	complement(2473165..2473488)	83	146	82	188	141	266	288	0	0	46	99	147	134	90	129	42	0	39	157	0	74	287	230	0	122	69	72	85	114	177
CTC_RS14350	complement(2473618..2474442)	16	0	161	55	167	366	34	57	32	283	97	145	0	35	0	17	61	107	10	117	174	90	181	247	29	54	56	25	0	256
CTC_RS11985	2474641..2475000	0	0	0	0	0	0	0	0	0	0	0	0	0	0	0	0	0	0	0	0	66	0	0	0	0	0	0	0	0	0
CTC_RS11990	complement(2475030..2476205)	46	141	226	65	205	73	48	161	363	105	164	122	31	162	276	52	86	118	36	41	92	42	95	104	7	209	237	82	198	407
recX	complement(2476240..2476890)	0	255	82	0	106	26	14	193	287	127	123	73	44	169	145	73	116	175	59	74	220	57	57	125	37	103	178	32	151	324
CTC_RS12000	complement(2476918..2478150)	0	0	11	12	56	28	8	51	43	48	26	39	0	36	60	17	41	21	21	0	58	0	30	0	6	18	0	11	80	93
CTC_RS12005	2478291..2478815	103	0	0	29	0	0	36	60	0	388	0	0	27	56	0	26	0	0	32	92	46	47	0	0	15	0	44	105	0	73
CTC_RS12010	complement(2478841..2479722)	31	81	30	61	52	20	42	71	121	492	91	81	57	100	143	85	114	29	58	0	0	56	85	46	45	38	79	195	111	43
CTC_RS12015	complement(2479734..2480561)	33	86	32	37	28	21	11	38	32	364	19	0	9	0	0	25	0	76	21	0	0	15	90	49	10	13	28	75	30	46
proV	complement(2480566..2481750)	34	20	11	26	39	44	8	27	0	272	41	20	55	0	18	34	43	32	18	41	40	42	0	0	27	19	98	116	21	32
CTC_RS12025	complement(2482000..2483352)	10	649	442	11	271	127	0	721	434	22	523	123	5	839	434	25	1081	444	22	72	88	0	152	120	6	859	497	10	871	368
CTC_RS12030	complement(2483418..2486852)	4	83	23	2	60	10	0	50	16	9	108	35	0	128	61	8	103	100	5	14	28	0	0	12	5	172	34	12	107	50
CTC_RS12035	complement(2487019..2488017)	0	24	27	8	46	35	0	0	0	53	16	48	0	0	0	34	25	38	47	0	96	0	37	41	0	0	0	48	123	38
rbsB	complement(2488108..2489028)	484	232	245	522	75	299	0	0	0	6	349	855	0	0	0	695	247	69	185	263	416	377	446	354	0	0	0	105	107	146
CTC_RS12045	complement(2489071..2490006)	101	0	142	65	98	166	0	0	0	6	52	306	0	0	0	146	108	95	127	155	128	119	40	130	0	0	0	81	52	41
CTC_RS12050	complement(2490034..2491551)	98	0	18	63	15	23	0	0	0	0	21	47	0	0	0	72	17	17	84	96	32	16	74	27	0	0	0	36	0	13

rbsD	complement(2491551..2491946)	34	0	0	39	58	44	0	0	0	15	81	121	0	0	0	52	0	0	75	0	0	94	0	103	0	0	0	35	0	0
rbsK_2	complement(2491949..2492878)	15	26	14	57	25	56	0	0	0	10	17	26	0	0	0	132	27	0	87	0	51	40	80	0	0	0	0	22	0	0
safA	2493120..2493731	22	155	43	12	37	56	15	51	44	10	53	39	24	24	0	33	124	21	14	0	39	0	61	67	13	37	0	67	80	31
CTC_RS12070	complement(2493799..2494296)	68	48	80	15	46	35	37	126	0	12	129	96	43	88	126	14	51	25	17	0	48	50	0	82	16	67	0	41	49	0
CTC_RS12075	complement(2494493..2495785)	42	202	72	59	106	80	72	97	124	137	174	55	45	68	178	79	195	88	90	187	167	29	58	31	86	95	54	85	57	252
CTC_RS12080	complement(2495809..2497128)	20	81	30	35	17	13	42	167	0	121	73	72	30	78	79	103	134	163	132	220	163	19	28	31	30	76	18	52	167	160
thrB	complement(2497131..2498033)	30	79	59	17	51	38	10	70	30	78	18	53	0	16	0	45	56	14	80	107	0	0	41	45	9	99	77	38	109	64
thrC_2	complement(2498043..2499527)	0	112	18	8	62	35	6	64	54	44	76	64	10	99	28	60	85	51	77	33	161	0	126	0	11	68	16	28	0	52
CTC_RS12095	2499675..2500088	163	115	96	147	111	167	0	38	0	442	272	115	70	212	51	132	122	153	123	0	116	150	90	393	77	108	112	116	59	46
CTC_RS12100	complement(2500132..2500533)	34	295	728	38	285	600	46	274	399	455	920	712	54	583	261	85	251	693	106	602	179	31	186	506	39	361	404	257	427	906
CTC_RS12105	complement(2500752..2501663)	0	78	160	17	101	180	41	35	88	39	176	707	24	80	0	45	332	278	47	530	1154	27	205	446	0	61	127	23	175	147
CTC_RS12110	2501817..2502476	0	0	0	0	0	0	14	0	40	0	0	0	0	0	0	0	0	19	0	0	0	0	0	0	0	0	0	0	0	29
CTC_RS12115	2502494..2503282	17	0	0	0	29	87	0	20	0	37	0	0	0	0	27	9	0	32	5	0	0	0	0	52	0	0	59	0	0	0
CTC_RS12120	2503382..2503804	703	505	660	1046	867	1630	485	1042	1421	3051	608	480	171	519	198	1393	954	1556	975	1029	1584	689	529	2599	375	476	438	1820	812	2719
CTC_RS12125	complement(2503842..2505068)	11	0	11	0	0	14	0	0	0	0	0	19	0	12	0	6	21	10	0	0	0	0	0	0	0	0	19	0	0	0
CTC_RS12130	complement(2505367..2505714)	0	273	382	0	132	50	0	0	77	0	139	69	0	126	60	0	145	291	0	0	0	0	0	234	0	225	67	0	212	0
CTC_RS12135	complement(2505772..2505960)	0	2824	16042	0	1213	3648	0	4081	7137	0	1191	1326	0	3331	4106	0	4004	4924	0	256	506	0	197	862	0	3667	3435	0	2662	3296
CTC_RS14805	complement(2506004..2506447)	0	34806	70055	0	5421	7454	21	50502	47766	0	10933	8870	0	65133	40722	0	52502	40849	0	3922	2640	0	9499	4493	0	73069	42661	0	38419	14938
CTC_RS12140	complement(2506592..2507299)	0	0	0	0	32	0	13	0	0	8	0	0	10	0	0	10	0	0	0	0	0	0	0	0	22	0	0	0	0	0
yihA	complement(2507390..2507980)	23	80	45	65	78	87	79	53	45	150	82	101	37	74	35	69	171	86	151	0	162	63	63	0	54	76	39	186	208	32
lon	complement(2507998..2510313)	23	164	52	66	99	82	133	102	58	157	83	72	62	107	109	91	174	120	161	84	72	27	16	70	65	111	50	148	223	66
lonB	complement(2510529..2512220)	16	56	24	0	41	20	6	37	0	14	161	28	0	78	12	4	45	22	3	0	28	15	22	0	0	73	0	0	116	34
clpX	complement(2512360..2513649)	733	736	670	1136	1244	828	597	1025	745	1596	411	759	901	726	374	1309	665	1188	1101	562	779	1077	608	694	879	680	665	1039	609	1367
clpP	complement(2513676..2514260)	393	365	341	463	509	442	670	673	320	1282	192	449	685	475	179	600	302	974	589	662	1063	604	574	278	447	554	396	576	336	786
tig	complement(2514332..2515627)	1204	2077	1319	1687	1875	1662	1627	1834	2009	2336	1718	2044	1682	1389	1602	2281	1265	2345	2231	1288	2150	1388	1944	1696	1414	1570	1771	2589	1477	2470
CTC_RS12175	complement(2515846..2516625)	69	30	85	39	59	88	12	131	171	144	41	92	65	56	81	35	0	146	27	62	31	16	0	0	10	29	0	71	31	74
pyrE	complement(2516812..2517393)	0	82	46	0	39	89	0	0	0	30	110	0	12	0	180	23	43	44	0	0	0	0	0	140	0	19	80	0	0	0
CTC_RS12185	complement(2517415..2518308)	0	106	104	17	179	77	10	18	30	33	54	53	24	65	164	8	56	57	33	54	27	0	42	0	27	63	52	8	55	107
CTC_RS12190	complement(2518310..2519038)	37	65	55	42	0	47	38	43	37	49	0	98	20	80	115	9	35	35	17	0	0	34	102	223	0	31	127	19	0	0
pyrF	complement(2519053..2519919)	31	27	0	0	26	20	0	18	31	7	19	83	0	17	121	16	145	29	10	56	28	0	0	0	27	13	27	0	28	88
CTC_RS12200	complement(2519937..2521127)	11	60	67	19	19	58	47	0	22	15	27	160	0	37	70	0	21	85	11	0	0	10	0	34	13	19	78	12	0	32
CTC_RS12205	complement(2521130..2521564)	0	0	61	0	0	79	0	109	61	81	74	55	0	34	145	0	0	29	20	0	137	0	0	94	0	26	53	16	0	0
pyrB	complement(2521566..2522489)	15	13	29	33	25	19	0	0	58	32	17	181	8	0	91	0	27	137	9	52	104	27	40	0	0	48	0	22	27	83
CTC_RS12215	complement(2522698..2523270)	0	0	0	0	0	90	0	0	0	93	0	42	13	0	0	12	0	22	22	0	0	43	0	71	14	0	0	24	0	0
CTC_RS12275	complement(2524438..2524911)	0	100	28	16	0	109	39	0	169	37	34	201	15	124	44	14	53	80	27	0	202	26	0	86	0	47	49	15	259	0
rdgB	complement(2525059..2525664)	89	274	307	82	151	284	231	156	176	263	451	354	202	314	138	214	167	397	189	0	355	266	185	336	131	166	230	204	243	221
CTC_RS12285	2525842..2527218	0	34	0	0	67	0	0	34	19	0	35	0	0	43	0	5	0	0	0	0	17	0	27	0	0	32	0	0	18	0
CTC_RS14810	2527307..2527465	0	0	0	0	0	0	0	0	0	0	0	0	0	0	0	0	0	0	0	0	0	0	0	0	0	0	0	0	0	0
CTC_RS12290	2527560..2527763	0	0	65	0	0	84	0	77	0	145	79	0	0	0	0	67	0	62	21	0	117	0	0	0	0	0	0	67	0	188
gatB	complement(2527787..2529220)	537	480	417	474	336	577	852	582	447	665	482	657	599	459	351	484	475	675	522	540	651	208	247	397	642	374	517	544	300	361
gatA	complement(2529251..2530708)	440	163	137	196	409	366	608	302	256	444	121	327	337	221	158	409	329	547	434	365	525	238	282	307	321	241	111	401	143	368
gatC	complement(2530739..2531029)	325	163	228	131	473	474	513	0	184	203	110	328	372	101	72	328	520	261	453	166	411	171	513	140	27	77	159	496	84	329
CTC_RS12310	complement(2531180..2531449)	0	176	49	113	170	64	0	58	0	66	60	0	107	0	155	76	93	94	63	179	89	0	138	0	59	41	0	76	0	497
ligA	complement(2531475..2533481)	74	130	123	183	126	103	93	169	106	206	112	238	97	113	84	151	75	303	165	169	143	130	149	101	99	167	162	130	61	229
pcrA	complement(2533510..2535732)	30	43	36	48	62	85	67	42	24	112	58	107	62	59	19	86	34	137	63	65	65	45	34	0	50	45	21	59	11	121
CTC_RS12325	complement(2535884..2537068)	57	681	370	232	464	320	87	491	361	30	665	322	171	1099	425	178	681	256	140	653	323	209	693	275	207	637	636	29	911	453
CTC_RS12330	complement(2537104..2538417)	82	72	121	29	122	131	82	96	102	13	183	109	115	89	160	93	365	135	36	110	237	113	28	0	115	132	150	26	168	204
CTC_RS12335	complement(2538476..2538778)	178	0	263	25	378	114	92	156	0	39	318	79	214	97	208	22	333	125	210	1118	1263	41	246	134	26	184	230	0	162	443
CTC_RS12340	complement(2539067..2539783)	94	99	167	75	128	216	130	176	335	124	112	366	131	123	410	104	317	283	178	135	167	138	104	568	100	265	453	355	103	267
CTC_RS12345	complement(2539867..2540493)	323	303	318	292	256	962	446	176	682	1083	256	647	656	163	703	402	563	646	231	309	610	495	238	974	582	160	518	1042	548	550
CTC_RS12350	complement(2540566..2542569)	111	71	80	116	126	103	102	47	147	24	96	131	117	73	115	122	101	95	73	145	155	130	19	102	123	78	104	7	61	120
CTC_RS12355	complement(2542794..2543234)	61	108	30	104	208	78	85	143	61	54	146	54	164	266	238	201	114	373	159	110	0	84	423	92	162	380	368	171	223	391
CTC_RS12360	complement(2543246..2544595)	60	149	113	153	323	128	83	198	79	109	143	141	171	325	264	232	262	333	132	251	159	276	249	332	206	331	292	117	200	284
CTC_RS12365	complement(2544707..2546065)	0	17	0	11	0	0	0	0	0	17	35	35	5	11	15	5	0	9	9	0	18	0	0	0	18	0	0	0	18	0
CTC_RS12370	complement(2546081..2546530)	0	0	30	0	0	0	0	35	0	0	0	0	16	0	0	0	0	0	0	0	0	0	0	0	0	0	0	0	0	0
CTC_RS12375	complement(2546762..2547208)	60	0	30	154	179	116	0	0	0	132	108	53	0	0	0	0	0	0	0	0	0	28	83	273	0	0	0	0	0	0
CTC_RS12380	complement(2547201..2547656)	30	52	29	84	151	189	0	0	0	104	211	52	0	0	0	0	0	0	0	0	0	82	82	179	0	0	0	0	0	0
CTC_RS12385	2547819..2548109	0	0	0	0	0	0	0	0	0	0	0	0	0	0	0	0	0	0	0	0	0	0	0	0	0	0	0	0	0	0
guaA	complement(2548185..2549717)	150	279	191	234	209	146	198	339	192	304	178	296	202	344	192	302	197	347	304	284	351	186	365	292	191	270	151	242	176	250
guaB	complement(2549749..2551203)	121	114	137	157	126	95	71	184	37	106	99	98	141	241	101	225	191	283	178	199	148	128	333	224	177	184	159	151	287	250
CTC_RS12400	complement(2551534..2552268)	18	0	18	10	31	23	0	0	0	24	0	0	0	20	0	9	0	0	0	0	0	0	51	0	0	30	0	9	0	26
groL	complement(2552418..2554043)	1562	6258	2368	1638	6590	2905	1492	5441	2858	1549	2214	2187	2601	2935	2593	2763	1652	2235	1680	2469	3457	2259	5050	2867	2514	3375	2495	1524	996	2588
groES	complement(2554084..2554368)	474	3246	1353	696	3458	1391	589	3590	1406	394	1495	586	1102	2568	3017	2007	797	1466	776	1188	1931	610	2750	2071	863	2628	2603	700	689	1143
CTC_RS12415	2554683..2555321	0	0	0	0	0	27	0	0	0	0	0	0	0	0	0	0	20	0	0	0	0	0	0	0	0	0	0	0	0	0
CTC_RS12420	complement(2555325..2556050)	19	33	18	11	0	119	26	22	0	98	89	0	50	0	0	19	69	17	35	67	66	17	0	56	0	15	0	28	34	0
CTC_RS12425	complement(2556219..2557703)	91	128	54	221	77	133	144	79	108	139	119	80	49	69	113	128	68	43	256	130	209	109	126	55	80	105	62	262	50	103
CTC_RS12430	complement(2557907..2558827)	15	13	43	8	0	19	41	0	0	19	35	0	0	0	46	7	0	0	23	53	0	81	0	0	0	0	0	37	0	21
gltA	complement(2558896..2560290)	852	383	415	1255	263	525	1374	271	134	169	392	992	2031	241	496	1604	669	227	1105	1838	703	1997	281	321	1774	248	415	286	387	323
CTC_RS12440	complement(2560290..2561180)	470	453	224	1148	412	658	1016	177	150	186	424	589	1580	345	471	1319	368	213	630	1086	376	1782	293	594	1816	257	338	247	303	387
CTC_RS12445	complement(2561339..2562508)	3280	1054	1330	4902	1440	1842	6203	404	719	828	1319	1918	6206	263	610	4349	787	173	1810	4217	1799	5949	1116	1009	6947	392	674	394	797	1606
CTC_RS12450	complement(2562718..2563566)	26598	9164	10150	24161	8640	10953	35107	6369	7268	5833	13205	14647	31485	3707	7992	24308	9048	2028	16088	30426	11637	33555	9979	11005	36093	4180	9203	5312	11578	10645
CTC_RS12455	complement(2563596..2564606)	5721	1267	1539	7152	2607	3086	65	16	0	12	16	24	12308	1216	2324	7748	2857	827	6104	7416	3904	10800	3322	2738	10193	1537	2660	2686	3981	4001
CTC_RS12460	complement(2564627..2565406)	10508	2859	2506	8473	2145	3812	16403	2119	1781	2779	2349	5447	13801	826	1318	9422	2749	698	7759	7876	5060	13744	2871	3001	11926	960	1754	2860	3367	2421
CTC_RS12465	complement(2565424..2566563)	4220	1040	817	2892	603	771	9975	1015	750	210	352	345	2624	257	368	1386	642	56	3586	3458	755	4609	786	411	2783	255	427	271	581	168
CTC_RS12470	complement(2566577..2567359)	13773	3741	3023	12753	2927	2994	19563	3749	2558	1712	4393	9267	20441	1589	3724	14118	4124	994	11550	14982	6981	15537	4004	3120	21618	2398	3686	3205	5595	4113
CTC_RS12475	complement(2567628..2568263)	1296	1380	941	1716	1297	1423	1555	1547	1302	1950	1668	2815	897	1312	1237	1580	952	896	2039	1901	2426	1873	939	1504	1228	1318	1531	1908	1042	1145
CTC_RS12480	2568433..2570346	49	62	28	50	60	95	58	58	56	136	101	150	34	23	11	36	53	53	87	51	62	39	97	64	29	35	36	90	13	20
CTC_RS12485	2570654..2571625	0	0	14	8	0	0	0	16	0	36	0	0	0	0	0	0	0	13	13	0	0	0	0	42	0	0	0	0	0	0
proC	complement(2571669..2572481)	233	496	262	235	733	424	115	387	197	182	198	352	249	279	232	335	248	296	306	298	338	412	275	200	195	261	314	389	181	259
tsaD	complement(2572498..2573520)	172	70	91	172	67	168	196	154	131	150	79	280	113	157	103	233	148	223	164	95	246	133	73	358	62	186	159	175	96	206

CTC_RS12500	2573705..2574319	22	0	22	12	75	56	15	51	87	144	78	39	12	48	136	33	0	21	0	0	39	0	0	66	13	0	0	56	0	0
CTC_RS12505	complement(2574363..2574734)	2743	1020	393	6482	1048	602	2708	762	359	1095	259	1027	4288	315	620	4687	882	68	3103	1235	643	5654	1405	1259	4306	300	187	1682	5608	721
CTC_RS12510	complement(2574737..2576002)	2855	656	431	5250	878	497	2785	361	148	1614	330	698	3589	266	215	3685	279	120	2421	879	397	4768	929	643	3373	115	284	1151	3034	742
CTC_RS12515	complement(2576021..2577460)	2534	362	351	4075	430	305	3307	361	185	1384	257	414	3078	91	248	3233	385	88	1817	1008	465	4364	752	481	3304	147	225	812	2258	439
tnpA_3	complement(2577894..2578350)	0	0	0	0	0	0	0	0	0	39	0	0	0	0	0	0	0	0	0	0	0	0	0	0	0	0	0	0	0	0
CTC_RS12530	complement(2578628..2579458)	0	0	0	0	0	0	0	0	0	0	0	0	0	0	0	0	0	0	0	0	0	0	0	0	0	0	0	0	0	0
CTC_RS12535	2579640..2580350	0	0	0	0	0	0	0	22	38	83	23	34	0	0	0	0	0	0	0	0	0	0	0	0	0	0	0	0	0	0
CTC_RS12540	complement(2580452..2580832)	35	62	35	40	0	90	73	41	140	171	42	0	19	0	0	89	0	33	22	0	0	0	0	0	42	0	0	72	129	50
CTC_RS12545	complement(2580851..2581711)	0	55	93	18	0	80	22	55	124	41	93	28	25	34	24	12	29	118	30	56	28	14	0	0	18	26	27	52	57	22
CTC_RS12550	complement(2581715..2582611)	15	26	0	0	0	38	0	0	0	7	0	53	16	0	0	8	0	28	9	0	27	0	0	0	9	0	0	23	0	64
tsaE	2582795..2583253	0	103	29	17	0	338	61	34	0	206	105	208	16	96	183	45	55	110	19	105	156	0	244	355	17	24	152	105	107	125
tsaB	2583250..2583963	76	66	93	43	32	314	78	22	75	174	23	234	0	0	29	86	106	355	48	136	268	17	105	342	33	31	32	135	69	483
rimI	2583956..2584414	147	0	319	200	150	225	61	69	233	206	228	104	94	191	46	193	220	193	93	422	261	27	325	177	52	73	202	135	214	251
tyrS	complement(2584461..2585684)	221	174	11	206	75	56	267	103	22	232	158	156	301	84	86	245	124	124	377	40	117	193	61	100	356	55	76	306	40	157
CTC_RS12575	complement(2585887..2586309)	351	280	1258	289	379	1956	287	186	1515	154	3914	1382	171	692	595	169	775	658	30	114	1018	220	1059	2599	225	581	658	16	696	1042
CTC_RS12580	complement(2586497..2587609)	12	43	24	0	0	0	8	28	24	11	14	21	0	66	0	0	91	34	0	0	0	0	0	0	0	35	0	0	0	52
CTC_RS12585	complement(2587590..2589002)	0	17	9	0	16	12	0	22	19	0	23	51	0	31	0	10	18	9	3	0	0	0	0	0	0	24	0	0	0	14
CTC_RS12590	2589120..2590199	0	0	0	0	0	0	0	0	0	0	0	0	0	0	0	0	0	0	0	0	0	0	0	0	0	0	0	0	0	0
CTC_RS12595	2590373..2590537	328	288	403	46	139	104	170	95	0	179	97	145	87	0	381	165	0	0	52	0	0	75	0	247	192	68	0	208	149	232
CTC_RS12600	complement(2590852..2592441)	34	104	25	149	108	87	35	59	67	342	40	60	170	74	53	356	63	191	257	30	45	101	117	0	120	98	58	411	46	157
yidA	2592616..2593425	0	0	0	0	14	0	0	0	0	7	0	0	0	0	0	8	0	0	0	0	0	0	0	0	0	0	0	0	0	0
trpS	complement(2593496..2594497)	297	71	93	228	92	86	326	63	187	377	96	71	339	146	63	204	151	114	284	193	310	223	186	122	269	112	162	226	49	191
CTC_RS12615	complement(2594633..2595007)	1514	2246	1738	3602	2934	1287	1828	2141	3633	512	4908	3755	3975	1874	2293	6275	3566	2076	1599	2838	2775	3507	3483	2171	3595	1788	2658	651	2422	2147
CTC_RS12620	2595195..2595455	0	0	0	0	0	0	0	181	0	0	185	0	0	168	0	0	145	0	0	0	0	0	0	0	0	171	89	0	188	0
CTC_RS12625	complement(2595481..2603958)	2	50	35	4	27	12	2	54	16	8	68	7	2	79	10	6	65	37	8	6	14	4	9	19	2	71	3	2	43	43
lysA	complement(2604062..2605360)	73	164	72	117	106	80	86	170	144	141	136	55	94	169	48	220	194	224	329	335	184	143	172	188	82	181	89	238	151	89
CTC_RS12635	complement(2605672..2606964)	31	18	31	30	71	53	22	12	21	50	37	111	39	45	81	16	59	59	39	37	74	19	58	63	31	43	0	35	19	15
CTC_RS12640	2607073..2608257	23	0	67	13	0	15	8	27	0	45	27	0	6	25	0	6	0	11	7	0	40	10	0	52	7	38	0	0	21	49
CTC_RS12645	2608309..2609397	0	44	98	35	42	47	26	43	25	22	30	22	7	27	58	6	69	93	8	0	44	0	0	37	0	31	43	13	90	141
CTC_RS12650	complement(2609418..2611550)	70	156	150	129	150	295	122	148	200	327	75	168	71	89	108	185	106	321	132	204	359	70	105	324	59	68	250	132	196	279
ftsH_2	complement(2611588..2613462)	72	158	113	171	147	147	219	269	256	340	86	255	121	141	157	389	188	385	288	258	447	66	219	261	165	119	111	172	223	388
glpK_2	complement(2613637..2615118)	661	0	18	1076	93	12	277	42	0	219	22	16	872	10	42	1020	0	0	473	65	81	1055	38	27	728	45	31	302	215	13
CTC_RS12665	complement(2615397..2616572)	11	0	11	0	19	15	24	0	23	20	14	20	18	50	0	12	43	86	11	0	0	0	0	35	20	10	0	12	0	16
CTC_RS12670	complement(2616689..2617864)	115	0	34	13	117	132	103	27	0	15	27	61	25	0	0	110	129	522	14	41	102	32	32	35	81	10	118	12	0	114
CTC_RS12675	complement(2617884..2619281)	126	17	38	5	16	12	47	23	0	21	0	34	41	21	0	34	36	136	9	0	34	18	0	29	23	8	17	15	88	14
citF_1	complement(2619395..2620948)	235	31	17	15	30	33	162	0	0	8	10	46	42	19	67	57	114	220	5	0	92	24	12	0	51	7	15	0	16	0
CTC_RS12685	complement(2620938..2621846)	208	0	0	0	50	19	205	0	29	13	35	184	87	16	46	112	83	320	19	0	79	14	41	90	52	12	51	8	0	0
citD_1	complement(2621861..2622145)	142	0	0	0	0	60	131	0	0	41	0	0	0	0	74	48	0	222	30	0	168	0	0	0	28	0	0	0	0	67
CTC_RS12695	complement(2622441..2623106)	0	71	80	69	69	129	28	95	0	124	24	143	43	22	94	51	38	76	19	0	72	37	56	61	36	34	104	72	0	29
CTC_RS12700	2623426..2623866	0	215	407	0	312	215	21	393	606	27	656	379	33	266	428	46	229	804	29	110	163	28	169	462	18	51	158	109	334	565
CTC_RS12705	complement(2623897..2624562)	0	0	0	0	34	0	0	0	0	0	0	0	0	0	0	0	0	0	0	0	0	0	0	0	0	0	0	10	0	0
CTC_RS12710	complement(2624638..2625207)	0	0	0	0	20	0	0	0	0	10	0	0	0	0	0	0	0	67	30	0	0	22	0	0	0	0	0	0	0	34
CTC_RS12715	complement(2625384..2625977)	0	0	0	0	39	0	0	0	45	0	27	0	0	0	0	0	0	0	0	0	0	0	0	69	0	19	0	0	41	32
CTC_RS12720	complement(2626016..2626699)	0	0	19	0	0	25	0	46	0	17	23	0	0	64	0	0	37	0	0	0	0	0	109	0	0	0	34	0	36	0
CTC_RS12725	complement(2626717..2627193)	0	199	0	0	48	0	0	33	112	12	135	0	0	614	88	43	53	0	27	0	0	0	0	0	17	258	146	0	103	40
CTC_RS12730	complement(2627186..2628034)	0	28	0	0	0	0	0	0	0	7	19	0	0	17	0	8	0	0	3	0	28	0	0	0	0	26	0	0	29	0
CTC_RS12735	complement(2628021..2630366)	0	71	34	3	0	37	0	34	0	3	89	41	6	62	9	0	32	11	7	0	20	0	0	0	0	91	10	4	31	8
CTC_RS12740	complement(2630599..2631234)	42	112	0	0	108	108	0	74	0	9	126	0	11	69	33	0	0	20	7	0	0	78	0	128	25	18	0	22	77	0
CTC_RS14355	complement(2631323..2631973)	42	0	61	35	0	0	14	73	0	27	0	0	28	45	0	21	39	19	3	0	0	38	0	0	49	17	71	11	38	59
CTC_RS14360	complement(2632084..2633337)	22	38	74	18	27	0	22	31	0	0	13	57	46	35	17	5	40	10	0	0	0	10	0	0	6	45	18	11	0	76
rlmD_2	complement(2633847..2635229)	0	17	10	11	17	12	13	23	19	56	23	35	10	21	30	89	0	27	75	0	17	0	27	0	0	0	50	122	18	42
CTC_RS12765	complement(2635254..2635955)	0	34	0	0	0	0	0	0	0	34	0	34	0	0	0	10	0	0	61	0	0	0	0	58	11	16	33	39	0	0
asrC	complement(2636237..2637187)	14	0	42	48	0	54	20	17	0	68	0	50	15	0	44	29	106	67	4	51	50	13	39	0	8	35	73	14	0	81
asrB	complement(2637198..2637992)	0	30	17	10	0	65	23	0	34	82	40	30	0	37	26	9	32	0	0	0	30	0	0	51	20	14	29	78	0	24
asrA	complement(2637994..2638983)	14	24	40	8	23	52	9	16	0	72	49	0	7	30	0	14	0	13	9	0	0	25	75	41	16	0	0	35	25	10
CTC_RS12785	complement(2639011..2639781)	18	0	17	20	0	0	12	20	0	46	21	62	19	0	0	27	0	16	6	0	124	0	0	53	0	0	30	27	32	0
CTC_RS12790	complement(2639861..2640550)	392	172	328	597	133	275	744	319	387	351	140	138	826	170	243	326	146	147	474	140	243	575	108	118	529	138	101	80	178	264
pyk	complement(2640562..2642316)	951	365	402	1015	287	108	1536	314	578	370	330	258	1717	350	299	867	316	353	1005	386	341	1237	255	116	1523	401	436	233	168	573
pfkA	complement(2642339..2643298)	507	247	194	374	48	90	651	262	278	68	100	174	485	229	590	451	210	264	354	302	125	336	350	85	570	245	507	236	64	160
CTC_RS12805	complement(2643543..2647034)	81	82	53	120	118	94	99	90	54	139	60	212	101	117	60	144	137	170	175	83	301	36	96	117	82	90	53	124	95	214
CTC_RS12810	complement(2647056..2648402)	70	35	10	40	85	64	42	47	0	35	24	89	38	43	0	25	0	94	88	36	71	18	0	60	82	50	17	36	91	43
whiA	complement(2648406..2649362)	28	99	28	64	96	126	88	49	181	68	50	125	68	76	0	50	79	159	49	101	200	52	78	43	50	35	48	79	51	160
CTC_RS12820	complement(2649374..2650705)	41	125	70	63	34	155	154	71	80	71	72	143	76	88	63	82	57	233	132	36	198	93	140	214	60	117	87	36	92	173
rapZ	complement(2650702..2651586)	31	54	30	60	130	234	221	53	30	67	145	162	122	17	47	92	0	157	151	219	243	56	84	92	125	76	79	70	111	282
murB	complement(2651725..2652648)	73	539	763	149	124	224	212	664	347	205	435	77	141	840	953	221	683	541	221	0	155	67	242	176	129	895	803	208	531	228
uvrC	complement(2652793..2654661)	7	51	50	33	61	74	5	8	57	41	52	77	8	16	0	15	27	68	27	0	51	0	0	22	13	27	25	29	13	31
CTC_RS12840	complement(2654686..2655294)	0	39	44	0	0	57	15	0	0	68	26	0	0	0	34	0	0	42	7	0	39	0	0	0	13	0	0	11	0	0
CTC_RS12845	complement(2655399..2656295)	136	159	119	145	204	202	42	123	119	184	269	53	137	212	23	159	197	198	180	162	160	41	0	182	71	162	129	161	137	107
CTC_RS12850	complement(2656320..2657750)	71	116	93	80	144	126	98	110	37	136	56	133	71	123	59	76	35	133	175	0	67	52	78	114	67	117	49	130	154	94
CTC_RS12855	complement(2657752..2658954)	22	0	44	25	57	100	16	13	0	113	40	20	30	85	0	17	42	42	39	0	119	21	0	135	13	56	19	80	122	32
CTC_RS12860	complement(2658977..2659411)	31	109	0	35	53	0	43	0	61	109	37	55	33	67	0	47	58	29	39	0	55	29	0	94	73	51	0	63	0	0
uvrA	complement(2659509..2662331)	110	235	188	99	162	226	152	190	142	176	239	254	118	197	134	169	349	253	215	274	415	92	132	187	98	178	148	153	165	149
uvrB	complement(2662421..2664406)	7	12	20	19	81	61	23	16	0	65	32	108	7	29	21	17	76	57	30	24	12	6	56	0	0	11	23	52	37	19
CTC_RS12875	complement(2664410..2665708)	21	55	20	41	18	13	0	12	0	59	62	73	22	56	16	26	39	49	43	0	0	0	57	31	6	43	0	32	19	30
CTC_RS12880	complement(2665739..2666938)	11	237	0	25	76	129	47	52	0	79	268	40	54	232	35	57	126	84	90	81	80	52	156	136	36	205	39	152	184	0
ftsX	complement(2667100..2667990)	91	80	90	137	77	426	230	124	210	603	90	241	73	99	47	214	198	369	293	0	201	56	335	640	80	119	78	405	220	237
ftsE	complement(2667980..2668666)	39	138	203	156	133	276	272	92	78	524	23	278	221	149	61	307	220	424	545	70	226	181	272	0	242	163	101	460	107	223
CTC_RS12895	complement(2668850..2669704)	0	111	16	0	0	0	22	110	31	21	19	0	8	137	74	16	118	30	10	0	0	0	0	48	0	196	54	16	57	0
CTC_RS12900	complement(2669761..2670117)	151	598	2384	257	385	1062	261	1102	1122	546	855	1003	162	1476	1293	229	2403	1880	393	271	603	139	836	114	155	1957	1429	578	2063	966
CTC_RS12905	complement(2670077..2670397)	0	443	621	71	143	107	44	785	166	331	200	149	22	1186	523	233	1690	394	344	151	224	39	465	254	99	1027	361	278	382	239

alr	complement(2670505..2671668)	35	224	57	52	59	89	96	257	69	81	400	144	112	346	90	152	379	120	128	0	195	64	64	70	48	298	20	112	274	198
CTC_RS12915	complement(2671687..2672295)	0	78	44	25	0	0	31	0	44	29	132	78	36	48	69	11	124	104	49	0	39	0	61	0	13	110	38	11	40	31
CTC_RS12920	complement(2672379..2673881)	18	63	97	36	214	92	6	84	178	39	75	111	34	68	140	68	34	118	25	0	175	41	99	108	11	164	123	37	16	255
acpS	complement(2673878..2674258)	35	62	209	0	60	181	0	83	491	16	211	439	0	346	440	179	66	199	33	0	251	65	196	0	42	205	304	36	64	604
CTC_RS12930	complement(2674332..2674685)	76	2010	1653	151	1166	1217	53	3468	3018	67	1453	3034	82	3598	4562	231	641	1574	108	1230	980	0	1054	345	45	2384	4388	58	1317	4630
CTC_RS12935	complement(2674919..2675374)	0	0	0	50	0	76	0	0	59	65	0	0	0	0	0	15	0	83	19	0	0	27	0	0	9	25	0	15	0	0
CTC_RS12940	complement(2675474..2676778)	0	36	10	41	35	40	29	24	41	77	86	37	19	22	0	16	19	0	10	74	18	19	0	125	12	34	53	26	19	29
CTC_RS12945	2677155..2678456	114	146	31	152	106	132	165	73	21	113	148	37	78	79	48	178	19	97	70	0	129	100	86	31	183	112	107	84	189	213
CTC_RS12950	complement(2678475..2679314)	0	28	0	55	0	41	11	56	0	63	38	0	9	17	25	24	60	15	35	0	0	30	0	0	0	27	0	25	29	46
CTC_RS12955	2679718..2679987	0	0	0	28	0	128	0	0	0	109	0	0	27	54	0	0	0	0	0	0	89	0	0	0	0	0	0	0	0	0
CTC_RS12960	complement(2680025..2681389)	0	0	19	11	0	13	0	0	0	0	12	0	0	0	0	10	55	28	9	0	35	0	0	0	0	8	0	5	0	28
arcC	complement(2681503..2682450)	86	125	140	258	169	73	79	183	85	221	34	151	160	263	155	230	585	414	242	153	151	170	472	172	217	554	171	268	337	182
CTC_RS12970	complement(2682604..2683554)	597	5288	587	2223	4459	852	579	3741	618	124	1099	1079	4413	1670	662	2195	1035	666	2305	610	604	2179	3552	385	3919	1440	963	260	1858	665
porA	complement(2683566..2684756)	851	5049	592	2146	4205	550	666	4421	493	124	931	641	4106	1610	757	2322	1038	941	2445	1015	944	2636	3510	444	3805	1164	662	214	2782	885
CTC_RS12980	complement(2684772..2685077)	132	3721	130	1110	2772	113	427	2521	262	19	630	234	4399	1818	960	2704	1154	372	1716	1107	235	1804	1464	133	4095	1205	682	315	3088	564
CTC_RS12985	complement(2685064..2685648)	323	2960	455	1318	2860	383	255	1910	91	131	769	286	3880	1176	861	1816	604	974	1388	496	164	1230	2042	209	3199	974	674	153	2433	623
CTC_RS12990	complement(2685673..2687031)	497	2217	264	1078	2648	355	254	1622	177	87	580	263	1888	458	201	990	316	261	715	463	370	968	1895	300	1549	395	290	167	984	367
megL	complement(2687117..2688313)	666	6738	1266	1881	7133	1152	655	6542	1026	49	5311	1635	4070	2189	1630	2157	2150	777	1140	364	500	2353	8029	918	3922	1657	2576	198	3875	993
CTC_RS13000	complement(2688513..2690072)	9	46	43	0	44	110	24	30	34	11	165	107	23	103	81	39	162	89	30	93	107	8	0	78	25	104	104	33	110	74
CTC_RS13005	complement(2690203..2691183)	331	387	380	218	444	474	528	481	272	280	606	949	221	269	278	295	437	258	353	641	341	240	609	498	222	182	213	179	550	449
CTC_RS13010	complement(2691412..2691783)	109	383	393	574	678	927	25	381	503	318	1318	1347	155	236	395	275	407	204	269	1560	1093	467	1605	1313	192	300	187	296	264	258
CTC_RS13015	2692017..2692418	0	0	17	0	0	0	0	0	0	59	0	0	0	0	0	0	0	0	0	0	0	0	0	0	0	0	0	0	0	0
CTC_RS13020	complement(2692424..2693209)	0	60	34	68	29	154	0	80	0	105	102	167	37	19	133	26	0	32	27	0	0	16	142	0	30	57	88	57	0	73
CTC_RS13025	complement(2693199..2693960)	18	0	17	10	0	45	0	0	0	47	84	157	19	19	0	0	0	33	17	0	31	0	0	53	42	0	0	18	0	0
CTC_RS13030	complement(2693957..2694808)	32	28	16	9	27	61	11	0	0	62	75	28	8	17	0	0	0	0	5	57	84	0	0	0	0	0	0	0	0	22
CTC_RS13035	complement(2694814..2696133)	31	0	0	6	52	26	0	12	0	40	12	54	5	11	0	5	19	10	0	0	0	9	28	0	6	8	0	0	0	0
CTC_RS13040	complement(2696155..2697003)	48	28	31	27	54	122	0	19	0	42	19	112	26	34	25	8	0	0	30	0	0	0	44	0	9	0	0	8	0	0
CTC_RS13045	complement(2697000..2698133)	12	0	0	20	0	46	0	14	0	68	28	105	0	0	0	6	0	11	7	0	21	0	0	0	0	10	0	6	0	17
CTC_RS13050	complement(2698135..2699019)	84	80	45	34	104	117	53	36	0	80	18	270	114	33	24	31	0	43	72	0	54	0	211	92	81	57	26	31	0	22
CTC_RS13055	complement(2699073..2700071)	0	0	0	0	0	0	0	0	27	0	0	0	0	0	0	0	0	25	0	0	0	0	0	0	0	0	0	0	0	38
glmS	complement(2700348..2702174)	377	610	309	950	502	302	536	728	322	504	326	281	555	609	241	1014	469	613	884	582	288	849	552	223	447	728	228	606	618	829
glmM	complement(2702536..2703882)	662	264	158	467	264	205	616	280	139	756	263	266	605	543	327	1047	243	479	1315	377	320	438	139	121	612	473	189	801	383	256
buk	complement(2704018..2705088)	1520	421	621	1033	878	676	1463	867	574	1255	825	2028	1577	273	392	1393	942	307	1366	2123	893	1077	732	627	1163	689	303	1377	825	483
ptb	complement(2705111..2706019)	847	365	249	529	177	341	842	312	59	942	566	972	631	242	162	772	361	167	676	1171	474	450	411	246	925	234	179	794	189	358
CTC_RS13080	complement(2706162..2707763)	42	44	42	143	29	54	105	79	50	133	125	238	70	91	13	174	94	119	231	30	269	23	93	0	94	98	58	116	8	36
CTC_RS13085	complement(2707744..2708994)	32	114	138	85	147	165	131	88	149	161	180	153	35	152	84	82	0	182	75	77	325	40	179	163	25	143	19	55	49	169
cdaA	complement(2708964..2709812)	96	56	172	45	216	102	11	19	31	195	189	84	26	52	0	64	89	89	80	57	56	58	132	96	9	39	27	16	0	158
CTC_RS13095	2709985..2710974	0	0	0	0	0	0	0	0	0	0	16	0	0	0	21	7	0	13	0	0	0	0	0	0	0	0	0	0	0	0
CTC_RS13100	2711141..2711668	0	45	50	0	130	0	0	119	51	0	365	0	0	55	0	0	48	0	0	0	0	0	0	0	0	85	0	0	70	73
CTC_RS13105	complement(2711702..2712568)	47	82	46	18	132	20	86	154	108	61	204	55	33	169	48	134	116	58	132	167	83	57	0	47	101	90	107	79	28	66
trxA	complement(2712638..2712955)	3315	3506	2676	1992	3316	2222	2435	3217	5376	2359	5863	3303	1929	4051	4551	1671	2618	2468	2888	3499	4438	1814	4225	5121	1796	3199	4885	2119	2238	3014
CTC_RS13115	complement(2713050..2714303)	54	0	21	24	0	41	22	25	0	61	51	38	17	35	67	11	20	71	44	39	57	0	0	65	13	18	0	16	39	31
citG	complement(2714432..2715343)	30	78	139	17	101	208	143	104	117	84	18	183	32	64	207	22	249	139	70	159	157	27	0	45	17	61	127	23	27	147
citX	complement(2715360..2715872)	26	0	0	45	45	67	18	31	0	35	94	140	14	57	0	27	98	74	33	94	0	24	0	0	0	0	0	27	0	37
citC	complement(2715874..2716917)	52	91	96	29	44	124	45	60	77	74	15	46	97	42	40	52	48	158	122	232	92	24	71	234	53	75	111	46	24	55
citF_2	complement(2717037..2718578)	1617	1861	1018	153	1962	1526	874	1501	624	1434	886	4009	300	636	599	607	1211	948	474	1443	1288	113	2130	1162	273	652	707	1788	1130	1380
CTC_RS13140	complement(2718628..2719503)	933	650	797	105	1217	944	618	1204	640	1072	569	2834	198	785	730	303	950	853	471	884	1092	42	1576	604	172	874	688	1161	504	810
citD_2	complement(2719541..2719804)	819	1438	756	116	1389	979	530	1222	809	716	670	1175	82	471	795	335	1051	432	403	1283	816	94	848	771	210	635	615	963	465	508
CTC_RS13150	complement(2719881..2720438)	1744	3146	1585	369	2793	1900	936	3230	1915	1249	1440	6117	414	2230	1955	708	1944	1475	1181	4595	1543	289	2809	1021	526	2364	1932	1848	2287	1718
CTC_RS13155	complement(2720452..2721294)	2565	3109	2335	453	4555	2760	1472	2960	2091	2396	1430	7814	805	2336	1717	945	2678	2140	915	3443	1674	545	2878	2342	569	2572	1678	2487	2475	3104
CTC_RS13160	complement(2721410..2722657)	14916	11556	6841	2048	11617	7313	8297	11465	6357	6140	7302	25322	2941	7144	5916	4636	12442	5950	5933	17074	6153	2749	12516	7454	3949	8276	5740	9665	10856	8409
CTC_RS13165	complement(2722798..2724249)	3202	4427	2798	594	5067	3591	1298	3567	2024	3205	2585	12353	597	2939	2376	1407	4239	3026	2350	7029	4004	496	3984	2776	672	2710	2938	4595	3879	4013
glmL	complement(2724265..2725653)	2340	3680	3351	687	4480	6372	766	3507	2423	4516	3228	13403	379	2266	2635	1187	4404	3951	2381	7818	6398	313	3990	4543	428	2475	2771	5672	4612	3533
mamA	complement(2725723..2726136)	1110	1891	867	350	2049	1957	394	2053	1032	1220	1631	5477	366	2581	2685	872	2224	1621	1833	5258	1329	210	1803	2065	306	1809	1848	2258	3290	1204
CTC_RS13180	complement(2726198..2727172)	28	170	259	16	259	265	19	371	164	200	99	392	7	60	258	98	233	493	96	99	589	0	191	42	8	92	119	113	176	236
cwlD	complement(2727372..2728076)	0	34	0	0	0	24	0	0	0	0	23	0	0	0	0	0	0	0	0	0	0	0	0	0	0	16	0	0	0	0
rpsI	complement(2728171..2728563)	499	1871	1421	1223	2362	1776	712	1322	1495	4546	859	972	514	968	747	1534	835	2158	1590	615	2861	916	1947	1554	605	910	590	3805	749	2658
rplM	complement(2728594..2729028)	652	2072	1192	1553	2371	1902	718	2099	2149	3435	1145	2305	1045	1918	1856	2114	1334	2794	4095	1334	1210	970	1544	1310	893	1760	1652	5294	1016	1939
truA_2	complement(2729164..2729910)	36	32	0	112	31	138	0	21	0	676	22	0	0	0	28	18	34	51	205	0	0	17	50	54	11	15	0	341	0	26
CTC_RS13205	complement(2729913..2730710)	68	30	33	163	86	108	12	138	67	533	0	120	32	147	53	213	0	111	591	0	90	124	47	0	30	112	58	603	92	144
CTC_RS13210	complement(2730723..2731589)	31	82	31	114	79	99	11	73	31	443	93	28	54	219	24	134	58	131	397	0	28	129	0	47	37	103	160	357	85	88
CTC_RS13215	complement(2731574..2732419)	144	112	189	262	190	163	22	149	32	880	95	85	145	69	25	161	89	150	475	57	141	249	221	0	0	79	82	666	145	136
rplQ	complement(2732496..2732837)	7152	3988	1011	4217	1877	1310	3369	2439	625	6252	658	628	1920	1948	1349	3366	811	1555	8404	707	420	3809	3056	119	1577	2713	678	12784	1364	1177
CTC_RS13225	complement(2732872..2733819)	2017	926	365	1320	991	636	984	565	113	5280	432	529	533	587	155	1200	160	628	3146	255	454	1224	827	215	335	672	220	4376	375	748
rpsD	complement(2733894..2734514)	3145	1719	632	1911	1587	833	1352	1293	387	5031	440	269	744	1037	372	1832	488	836	4344	1091	693	1419	1082	656	664	972	523	4948	553	803
rpsK	complement(2734545..2734943)	3150	2081	467	2563	1436	907	1683	1657	335	4152	645	479	1212	1798	946	2398	253	1301	7464	1091	689	2363	1684	408	1272	2577	697	7650	1107	1057
rpsM	complement(2734967..2735338)	1271	893	322	1518	1232	649	802	762	215	3207	346	449	660	748	338	1520	136	715	3137	260	514	1468	1505	766	384	811	249	3410	462	515
rpmJ	complement(2735445..2735558)	0	0	0	0	0	0	0	0	0	0	0	0	0	0	0	0	0	0	37	0	0	0	0	0	0	0	0	0	0	0
infA	complement(2735574..2735792)	710	650	61	418	209	394	256	359	244	1321	73	109	297	201	96	653	230	347	1728	442	328	283	170	0	435	766	106	1915	224	525
CTC_RS13255	complement(2735799..2736080)	288	168	141	487	244	428	33	167	0	1686	114	85	102	208	149	411	179	180	942	172	170	264	132	0	112	159	0	1804	174	136
map	complement(2736097..2736846)	1982	1550	736	2127	1742	724	871	798	392	7107	471	477	813	995	308	1389	135	1249	4323	838	861	1919	1393	271	592	984	433	5605	622	869
CTC_RS13265	complement(2736846..2737496)	996	692	388	1559	1426	794	444	774	451	5525	518	275	322	607	354	847	271	827	2221	297	735	1010	1003	563	512	695	178	3612	452	1325
secY	complement(2737511..2738788)	883	687	364	1140	924	964	398	456	21	6209	195	374	302	430	205	773	237	654	1432	265	767	782	672	319	347	507	209	2940	230	697
rplO	complement(2738790..2739230)	919	968	181	1211	624	704	381	714	303	3589	255	379	475	398	190	672	0	718	2102	439	271	1182	508	277	216	684	210	1988	390	565
rpmD	complement(2739252..2739431)	0	0	222	254	0	192	0	175	297	427	89	0	40	0	0	227	0	141	449	0	0	207	0	0	0	373	129	229	0	106
rpsE	complement(2739444..2739941)	950	953	134	1417	736	312	487	727	215	1654	452	240	1015	1293	421	1491	203	636	3855	583	432	1109	600	245	764	1436	559	2623	394	308
rplR	complement(2739960..2740319)	1839	2108	813	2544	2038	1029	829	1071	445	5923	357	729	942	1769	699	1684	210	1301	4346	134	1263	2499	1659	1018	815	1552	837	4010	545	639
rplF	complement(2740340..2740882)	1132	2708	735	2024	1393	1048	722	1594	689	5395	829	967	1196	1321	946	1662	372	863	3833	1425	925	2285	1684	600	993	1770	512	4703	497	1271
rpsH	complement(2740905..2741303)	1355	2675	533	2601	1551	648	620	1381	335	4307	725	1137	1230	1871	999	1647	253	1174	4220	1091	899	1835	1824	306	1103	2199	407	3963	123	817
CTC_RS13305	complement(2741334..2741519)	145	893	357	697	370	649	0	677	144	1397	259	0	233	394	226	403	0	613	731	0	257	300	803	0	213	210	499	517	132	103

rplE	complement(2741535..2742077)	772	1158	490	1869	1773	1175	481	957	394	4884	681	945	871	1348	657	1449	348	1212	2549	1203	1145	1588	2062	825	759	1225	897	2551	497	1094
rplX	complement(2742099..2742419)	968	2069	829	2318	2714	1396	610	1863	666	6624	551	855	1304	1733	653	1358	236	1420	3212	1733	1043	2358	1279	1522	1235	1602	1083	2891	382	1612
rplN	complement(2742438..2742806)	879	2571	613	2233	1739	888	480	1408	434	2273	479	647	1193	2658	1648	1652	479	1098	3992	655	1297	2118	1416	993	903	2151	1005	2608	665	1195
rpsQ	complement(2742837..2743091)	1378	3535	1669	2544	3416	2771	549	2037	2409	4957	1576	1685	1528	2296	2303	1870	1286	2557	3576	1707	2064	3430	2049	2395	964	3068	1818	3828	866	1729
rpmC	complement(2743115..2743327)	63	557	624	1326	323	728	219	517	125	970	226	560	339	756	591	480	415	416	1767	454	562	757	701	191	726	577	762	646	346	180
rplP	complement(2743317..2743760)	883	1816	629	1633	1600	893	378	1560	782	1623	796	941	1642	1780	1441	1381	511	1112	3543	763	916	1369	1093	825	1125	2014	783	2268	995	1036
rpsC	complement(2743778..2744446)	465	1135	537	1460	1131	1237	195	1435	639	2335	721	928	971	1488	1191	1324	528	1363	2962	578	1001	1206	1339	609	735	1437	901	2487	477	860
rplV	complement(2744463..2744798)	1086	2612	1108	3781	2865	1898	555	2577	1033	4834	909	2096	2148	2266	1810	1845	526	2148	4226	1872	2207	2659	2944	1939	1275	2528	1932	3253	584	1769
rpsS	complement(2744821..2745105)	285	832	140	843	483	544	196	552	94	1098	508	335	431	796	221	621	177	422	1455	509	420	827	917	571	431	961	488	1146	172	135
rplB	complement(2745172..2746002)	943	2526	1072	3021	2345	1877	561	2169	1093	8173	1102	1738	1928	2237	1552	2668	577	2133	6040	1455	1943	2703	2133	1102	1713	2347	1562	8438	738	2145
rplW	complement(2746084..2746377)	1195	1210	769	2933	1559	1290	254	857	636	4440	820	649	1412	1046	642	1923	429	1486	4584	1316	814	2110	1904	277	1214	1749	552	5752	501	1500
rplD	complement(2746377..2746997)	533	821	353	1591	867	611	270	1039	516	3742	466	577	802	684	439	1415	122	550	2711	701	655	1489	1022	557	945	558	485	3288	395	741
rplC	complement(2747023..2747652)	1030	2485	1045	4403	1674	1354	740	1674	636	7237	918	1515	3173	2022	1065	4297	721	2201	8210	1229	1956	3417	2310	1066	2593	2085	1582	10187	1208	1491
rpsJ	complement(2747743..2748051)	394	921	689	1976	1039	781	211	713	605	3880	624	695	1378	948	747	1763	82	1188	3110	313	774	2209	242	1054	1001	832	825	3938	397	1210
tuf_1	complement(2748397..2749590)	113	238	28	195	326	238	31	13	0	25	0	0	30	25	35	63	21	21	21	0	20	281	31	136	27	47	10	0	21	0
fusA_1	complement(2749640..2751709)	764	1713	591	2011	1146	849	460	1278	477	4993	474	657	1634	1238	497	1841	427	1187	3173	946	717	2062	1217	570	1435	1296	493	3959	848	1232
rpsG	complement(2751798..2752268)	1607	2770	593	3313	1655	769	941	1504	397	5574	546	1115	3057	1554	1002	3688	911	1694	7682	1027	863	2792	1902	519	2568	2160	689	8816	1146	1628
rpsL	complement(2752439..2752816)	804	784	352	1262	1031	958	420	541	212	5164	128	568	917	349	222	1252	200	754	2115	576	538	968	592	323	755	651	552	3628	325	938
CTC_RS13400	complement(2752872..2753117)	55	0	0	124	186	561	0	192	217	792	196	97	176	60	0	83	103	257	225	0	194	151	152	165	161	0	188	419	100	156
rpoC	complement(2753245..2756771)	284	928	541	1090	910	579	278	817	432	1809	401	1086	627	807	526	672	526	752	1196	494	705	936	884	369	625	849	500	986	772	859
rpoB	complement(2756794..2760504)	184	524	351	807	556	283	249	501	331	1280	273	650	487	505	407	507	204	641	787	417	490	488	342	263	447	587	300	658	410	537
rplL	complement(2760769..2761134)	4135	2852	2144	5515	3131	3155	2269	2623	2153	14547	2262	2804	3481	3840	2063	4569	758	1730	11477	2115	1895	5509	2345	2113	2882	3314	2724	17581	1073	3221
rplJ	complement(2761171..2761671)	3196	2273	1181	3937	1647	2202	1397	1131	1546	16946	1187	1596	2046	1110	1193	3086	302	1440	5652	1593	1241	4124	1564	1463	1836	1450	1111	12734	735	2296
rplA	complement(2761879..2762568)	2262	1616	944	2947	2159	1324	1217	1403	832	7143	815	761	2165	1591	942	3075	622	1321	6134	1087	1525	2625	1379	708	1977	2057	857	8294	1458	1945
rplK	complement(2762621..2763046)	4505	3981	2685	6932	4466	2954	2376	4009	3699	15632	2528	3138	4007	3780	2363	6092	1421	4800	9097	2158	4829	5316	4731	2771	3034	4119	3429	18647	1844	7379
nusG	complement(2763122..2763643)	1838	3544	1681	4685	3974	2229	1778	2247	2072	9793	1416	2240	2606	2748	1246	3882	1232	4123	6125	2965	4262	2924	3289	2262	1823	3137	1599	12472	1787	4884
secE	complement(2763720..2763956)	285	601	281	451	484	582	433	930	733	2068	339	504	426	741	177	632	319	908	915	1021	404	209	472	687	368	990	293	1566	0	1618
rpmG	complement(2763998..2764147)	0	158	0	203	0	0	187	105	178	157	0	0	241	98	0	136	168	84	510	0	0	0	0	271	159	224	155	321	0	128
tuf_2	complement(2764338..2765531)	300	834	434	358	365	130	367	791	582	460	377	400	632	1251	799	1107	803	732	1106	729	381	416	219	170	737	1292	883	1114	802	658
sigH	complement(2765774..2766367)	228	479	291	450	502	493	188	450	135	398	189	80	365	271	177	195	255	384	333	81	403	313	691	206	267	282	39	220	83	290
CTC_RS13465	complement(2766483..2766995)	237	370	337	431	715	437	409	368	260	535	282	558	310	457	82	432	443	370	365	283	466	629	437	238	448	436	362	228	239	710
rlmB	complement(2766998..2767735)	220	386	144	434	435	374	310	256	181	280	414	420	313	258	114	341	103	472	202	131	454	412	683	110	258	470	188	149	100	468
thyX	complement(2767750..2768484)	257	290	199	260	249	446	292	364	218	213	284	292	275	279	171	241	275	207	179	66	228	371	102	277	216	350	63	168	200	548
CTC_RS13480	complement(2768468..2768923)	237	416	204	586	251	529	604	535	410	259	282	366	744	449	184	433	277	500	336	636	682	939	491	179	435	735	153	181	484	420
cysS	complement(2768956..2770353)	271	288	219	355	467	284	307	265	267	228	195	615	413	346	255	385	180	235	228	190	291	364	374	262	434	316	265	103	176	398
ilvA	complement(2770368..2771579)	446	920	647	636	889	526	901	896	485	307	1101	1123	917	761	727	888	687	522	582	758	947	758	1355	269	874	775	440	250	466	474
CTC_RS13495	complement(2771630..2773342)	339	249	132	183	428	267	321	193	172	229	56	188	324	145	98	203	177	229	243	198	265	123	414	309	215	192	149	134	86	369
ispD	complement(2773637..2774344)	57	67	169	129	97	414	92	67	0	209	204	371	51	41	178	87	143	179	144	68	135	140	211	345	45	189	33	146	243	325
CTC_RS13505	complement(2774370..2775473)	49	236	277	124	145	265	34	214	121	393	248	195	92	159	152	160	617	396	139	307	412	56	135	406	65	127	210	196	378	417
CTC_RS13510	2775763..2776161	0	119	333	0	0	346	23	39	67	15	242	60	0	37	210	17	190	254	32	364	120	31	187	0	20	28	116	0	123	384
disA	complement(2776202..2777275)	63	177	50	28	64	144	100	147	99	82	224	311	60	68	78	146	235	177	150	135	200	35	70	190	89	62	108	141	137	71
radA	complement(2777291..2778655)	30	70	97	45	118	177	185	58	59	91	82	157	42	43	92	147	74	176	170	106	263	36	137	209	52	98	17	106	144	84
nagB	complement(2778788..2779513)	74	65	110	95	95	166	141	108	37	195	89	164	139	101	173	319	104	105	164	200	66	137	103	168	87	92	128	85	237	79
CTC_RS13530	complement(2779669..2782104)	338	1071	759	816	1313	1309	172	520	647	470	1824	2391	151	631	517	233	476	764	147	318	678	507	1295	1053	104	505	590	71	474	936
CTC_RS13535	complement(2782133..2783155)	198	672	364	351	1064	623	128	477	261	393	1123	1400	71	343	308	87	197	285	58	331	327	267	511	955	70	361	363	20	312	525
CTC_RS13540	complement(2783167..2783685)	156	503	205	338	1060	531	144	152	515	205	1084	828	56	197	566	105	194	195	57	93	507	383	647	235	61	431	179	40	473	295
CTC_RS13545	complement(2783742..2784200)	0	52	116	100	300	188	102	34	291	51	140	52	31	64	91	59	0	193	28	0	52	0	163	266	17	49	0	15	160	42
CTC_RS13550	complement(2784424..2785059)	21	261	42	12	0	0	44	99	84	74	51	0	0	92	66	54	79	60	80	0	0	0	0	64	0	70	109	65	116	60
CTC_RS13555	complement(2785118..2785342)	0	0	0	0	0	0	0	0	119	0	0	0	0	130	0	0	0	0	0	0	0	0	0	0	0	0	0	0	109	0
CTC_RS13560	2785527..2786114	2448	3994	5722	3738	6159	10201	2856	3681	9517	1075	10715	7692	2449	3573	5030	3527	5127	4920	1320	5018	7527	3598	6792	12705	2549	3317	5540	959	2045	5428
fusA_2	complement(2786171..2788255)	3575	1945	1167	4044	2133	893	4949	1744	1390	85	2725	1099	5537	2528	1926	2231	1821	1245	922	1369	1251	4871	1709	391	5245	2383	1434	23	1524	1200
CTC_RS14370	join(73941..74082,1..140)	0	0	0	0	0	0	0	0	0	0	0	0	0	0	0	0	0	0	0	0	0	0	0	0	0	0	0	0	0	0
CTC_RS13635	441..1826	29	51	86	135	281	261	44	74	0	43	116	129	96	37	106	25	100	78	84	384	337	54	229	117	23	56	59	92	142	90
CTC_RS13640	1959..2228	0	0	0	0	0	0	0	0	0	77	0	0	0	0	0	0	0	0	0	0	0	0	0	0	0	41	0	0	0	0
CTC_RS13650	complement(2876..3286)	0	0	0	0	0	0	0	0	0	0	20	0	0	0	0	0	0	0	341	11652	35214	0	0	0	0	0	0	0	0	0
CTC_RS13655	complement(3428..3646)	0	0	30	0	0	0	0	0	0	54	0	0	0	0	0	0	0	0	0	0	328	0	0	0	0	0	0	0	0	0
CTC_RS13660	complement(3671..3886)	0	0	0	0	0	0	0	0	0	0	0	0	0	0	0	0	0	0	0	0	222	0	0	0	0	0	0	0	0	0
CTC_RS13665	complement(4065..4406)	0	0	0	0	0	0	0	0	0	0	0	0	0	0	0	0	0	0	0	0	210	0	0	0	0	0	0	0	0	0
CTC_RS13670	4870..5223	0	0	0	0	0	0	0	0	0	0	0	0	0	0	0	0	0	0	12	0	0	0	0	0	0	0	0	0	0	27
CTC_RS13675	5316..5738	0	0	0	0	0	0	0	0	0	21	0	0	0	0	0	0	0	0	0	0	0	0	0	0	9	0	0	0	0	0
CTC_RS14815	5780..5950	0	0	0	0	0	0	0	0	0	0	0	0	0	0	0	0	0	0	0	0	0	0	0	0	0	0	0	0	0	0
CTC_RS13685	6216..6398	0	0	0	0	0	0	25	0	0	0	0	0	0	0	0	0	0	0	23	0	131	0	0	0	0	0	0	0	0	0
CTC_RS13690	6534..6860	0	0	0	23	0	105	0	0	0	45	0	0	0	0	0	0	0	0	0	0	73	0	0	0	0	0	0	0	0	0
CTC_RS13700	complement(7121..7327)	0	0	0	0	0	0	0	0	129	29	155	231	0	0	0	0	0	0	62	1636	231	0	0	0	0	0	0	0	0	0
CTC_RS13710	7792..8292	0	189	0	61	0	103	56	0	53	0	0	0	29	29	0	0	0	0	0	0	48	0	74	81	0	0	46	41	0	0
CTC_RS13715	complement(8469..9008)	250	44	25	35	170	32	130	87	74	0	0	0	13	14	0	69	164	70	24	179	44	11	104	75	7	0	0	13	0	0
CTC_RS13720	complement(9010..9564)	0	0	0	0	0	0	0	0	0	0	0	0	0	0	0	0	0	0	8	0	86	0	0	0	0	0	0	0	0	0
CTC_RS13725	complement(9833..10255)	0	0	31	0	54	0	0	0	0	0	0	0	0	0	0	8	60	0	101	286	1923	0	0	0	0	26	0	8	0	45
CTC_RS14895	complement(10398..10553)	0	0	0	0	0	0	0	0	0	0	0	0	0	0	538	0	0	0	0	0	0	0	0	0	0	0	0	0	0	0
CTC_RS13730	10828..11817	191	96	107	551	208	122	320	278	270	137	341	386	306	104	191	406	127	166	331	147	387	551	113	206	216	203	164	431	74	290
CTC_RS13735	11832..12173	0	0	0	67	0	50	27	0	0	52	0	140	0	0	61	80	74	0	37	71	0	0	0	0	0	0	0	0	0	0
CTC_RS13740	complement(12293..12571)	242	1615	1287	465	2054	896	234	1495	1532	85	115	43	259	735	601	49	90	91	114	173	343	400	1873	1021	313	681	748	49	176	137
CTC_RS13745	12873..13541	0	0	0	0	0	0	0	0	0	0	0	0	0	0	0	0	0	0	13	145	36	0	0	0	0	0	0	0	0	0
CTC_RS13750	13558..16011	0	0	0	0	0	0	0	0	0	0	0	0	0	0	0	0	0	0	14	39	68	0	0	0	0	0	0	0	0	0
CTC_RS13760	complement(16369..17391)	0	35	0	22	22	51	14	8	13	43	16	35	4	14	0	0	0	0	0	0	12	24	18	99	8	11	23	0	0	0
tnpA_4	complement(17429..17836)	0	58	0	0	0	0	0	0	0	0	0	0	18	36	103	0	0	0	0	0	0	0	0	0	0	110	85	0	0	0
CTC_RS13770	18195..18539	0	0	0	0	0	0	0	0	0	0	47	0	0	0	0	10	0	0	0	280	0	0	0	0	0	0	0	30	36	56
CTC_RS13775	18829..19047	0	0	0	0	0	0	0	0	0	0	0	0	0	0	0	0	0	0	0	0	0	0	0	0	0	0	0	0	0	0
CTC_RS13780	complement(19636..19851)	0	0	0	0	0	0	0	0	0	0	0	0	0	0	0	0	0	29	0	0	0	0	0	0	0	0	0	0	0	0
CTC_RS13785	20256..21941	48	183	166	118	367	337	33	56	63	70	38	14	30	26	87	28	75	68	33	0	85	52	89	338	42	80	110	35	15	91

CTC_RS13790	complement(21961..23022)	38	22	88	14	76	162	9	44	75	44	0	11	7	14	59	26	24	155	0	0	0	23	70	77	0	21	98	45	92	451
CTC_RS13795	23261..23458	0	0	0	0	0	0	0	0	0	0	0	0	0	0	0	0	0	0	0	0	0	0	0	0	0	0	0	0	0	0
CTC_RS13800	complement(23519..23737)	0	0	0	0	0	0	0	0	0	0	0	0	0	0	0	0	0	0	0	0	0	0	0	0	0	0	0	0	0	0
CTC_RS13805	23809..24495	0	0	0	0	0	0	0	0	0	0	0	0	0	0	0	0	0	0	105	0	487	0	0	0	0	0	0	0	0	0
CTC_RS13810	24499..25473	0	0	0	0	0	0	0	0	0	0	0	0	0	0	0	0	0	0	17	50	74	0	0	0	0	0	0	0	0	0
CTC_RS13815	25671..26078	0	0	0	0	0	0	0	0	0	0	0	0	0	0	0	0	0	0	0	0	0	0	0	0	0	0	0	0	0	0
CTC_RS13820	26108..26866	0	0	0	0	0	0	0	0	0	0	0	0	0	0	0	0	0	0	6	0	32	0	0	0	0	0	0	0	0	0
CTC_RS13825	26853..28781	0	0	0	0	0	0	0	0	0	0	0	0	0	0	0	2	0	0	4	0	0	0	0	0	0	0	0	0	0	0
CTC_RS13830	complement(29178..29375)	0	0	0	0	0	0	0	0	0	0	0	0	0	0	0	0	0	0	21	0	121	0	0	0	0	0	0	0	0	0
CTC_RS13835	29542..30111	0	0	0	0	0	0	0	0	0	0	0	0	0	0	0	0	44	44	15	0	42	0	0	0	0	0	0	12	43	101
CTC_RS13840	30338..31174	161	255	183	182	329	381	123	291	96	152	154	128	52	122	75	73	60	182	66	0	114	89	178	413	47	154	111	33	0	298
CTC_RS15035	31193..31504	0	0	0	0	0	0	0	0	0	0	0	38	0	0	0	22	0	0	164	0	345	0	0	0	0	0	0	0	0	0
CTC_RS15040	31639..31890	0	0	0	0	0	0	0	0	0	0	0	47	0	0	0	27	100	100	34	0	95	0	0	0	0	0	0	191	97	228
CTC_RS13850	32303..32668	0	0	0	0	0	0	0	0	0	0	0	0	0	0	0	0	0	0	192	793	588	0	0	0	0	0	0	0	0	0
CTC_RS13855	32685..32948	0	0	0	0	0	0	0	0	0	0	0	0	0	0	0	0	0	0	32	183	181	0	0	0	0	0	0	0	0	0
CTC_RS13860	complement(33162..34718)	0	0	0	0	0	0	0	0	0	0	0	0	0	0	0	0	0	8	0	0	0	0	0	0	0	0	0	0	0	0
CTC_RS13865	complement(35102..35482)	0	0	0	0	0	0	0	0	0	0	0	0	0	0	0	0	0	0	0	0	63	0	0	0	0	0	0	0	0	0
CTC_RS13870	complement(35521..36153)	0	0	0	0	0	0	0	0	0	0	0	0	0	0	0	0	0	0	0	0	0	0	0	0	0	0	0	0	0	0
CTC_RS13875	36699..37001	0	0	0	0	0	0	0	0	0	0	0	0	0	0	0	259	749	1630	0	0	0	0	0	403	0	0	0	352	972	2530
CTC_RS13880	complement(37332..37562)	59	103	230	66	695	1492	81	273	578	409	70	207	62	253	182	0	0	0	138	314	828	107	808	881	103	97	0	0	0	0
CTC_RS15045	complement(37559..37690)	0	0	0	0	0	0	0	0	0	0	0	0	0	0	0	0	0	0	0	0	0	0	0	0	0	0	0	0	0	0
CTC_RS13890	complement(37990..38466)	0	0	0	0	0	0	0	66	0	0	0	50	0	0	0	0	53	0	0	0	0	13	0	0	0	0	0	0	0	0
CTC_RS14820	complement(38488..38646)	0	0	0	0	0	0	0	0	0	0	0	0	0	0	0	0	0	0	0	0	0	0	0	0	0	0	0	0	0	0
CTC_RS14825	complement(38709..38882)	0	0	0	0	0	0	0	0	0	0	0	0	0	0	0	0	0	0	0	0	0	0	0	0	0	0	0	0	0	0
CTC_RS13895	complement(38919..39206)	0	0	0	0	0	0	0	0	0	0	0	0	0	0	0	0	0	0	0	0	0	0	0	0	0	0	0	24	0	67
colT	complement(39438..42413)	0	16	27	3	31	41	5	5	0	4	32	32	0	10	14	7	8	26	3	0	0	0	0	0	0	11	0	16	8	19
CTC_RS13905	42788..43603	0	0	0	0	0	0	0	0	0	0	0	0	0	0	0	0	0	0	0	0	0	0	0	0	0	0	0	0	0	0
CTC_RS13910	43596..44405	0	0	0	0	0	0	0	0	0	0	0	0	0	0	0	0	0	0	0	0	0	0	0	0	0	0	0	0	0	0
CTC_RS13915	44407..45192	0	0	0	0	0	0	0	0	0	0	0	0	0	0	0	0	0	0	0	0	0	0	0	0	0	0	0	0	0	0
CTC_RS13920	45189..45830	0	0	0	0	0	0	0	0	0	5	0	0	0	0	0	0	0	0	0	0	0	10	0	0	0	0	0	0	0	0
CTC_RS13925	complement(46073..46402)	0	0	0	0	0	0	0	0	0	0	0	0	0	0	0	0	0	19	0	0	0	0	0	0	0	0	0	156	74	378
CTC_RS13930	complement(46386..46895)	0	0	0	0	0	0	0	0	0	0	0	0	0	0	0	0	0	0	0	0	0	0	0	0	0	0	0	13	0	75
CTC_RS13935	complement(47307..48329)	13	58	39	0	22	51	5	8	0	72	16	82	4	43	0	0	0	0	17	0	129	12	18	60	0	0	11	0	0	0
tnpA_5	complement(48367..48801)	31	164	61	158	343	79	107	36	0	204	74	165	33	34	193	0	0	0	108	111	110	86	0	94	0	51	266	0	0	0
CTC_RS13945	complement(48826..49323)	54	0	53	77	230	138	19	32	0	95	32	0	0	59	0	0	0	0	0	97	192	0	75	82	0	0	0	69	0	38
CTC_RS13950	complement(49310..50104)	68	60	17	125	245	195	12	40	101	238	40	90	36	0	26	0	0	8	70	0	150	47	0	51	10	14	0	43	0	121
CTC_RS13955	50368..50796	0	0	0	36	53	0	22	37	0	69	0	0	0	0	49	0	0	0	0	0	0	0	0	0	0	0	0	0	0	0
CTC_RS13960	50975..51895	176	927	643	443	1070	1469	223	966	638	359	820	959	125	453	455	0	55	27	120	341	623	310	1459	1149	125	473	403	19	53	0
CTC_RS13965	52344..53732	39	205	196	49	206	366	44	176	192	134	619	498	52	216	204	103	536	355	129	592	896	45	309	234	57	48	142	94	371	193
CTC_RS14380	complement(54773..55213)	0	161	513	0	0	0	85	535	606	415	182	758	0	183	95	185	1030	1335	67	329	732	28	0	92	18	177	578	0	0	0
CTC_RS13975	56391..56750	75	198	222	233	637	1197	0	219	148	180	89	0	40	41	0	76	280	317	142	0	465	103	829	905	22	62	64	344	614	426
CTC_RS13980	56761..57186	32	56	62	107	269	263	44	0	0	14	38	0	0	0	0	16	59	149	50	0	0	29	263	573	19	0	0	48	0	270
CTC_RS13985	complement(57241..57954)	0	0	19	11	0	72	0	0	37	0	0	0	0	0	29	0	0	9	6	0	34	0	0	0	0	0	0	0	34	13
CTC_RS13990	complement(58025..58633)	0	0	66	0	38	283	0	0	44	29	53	39	0	24	69	0	41	52	21	0	39	0	0	0	0	18	38	56	0	63
CTC_RS13995	58890..59366	0	0	28	0	0	0	0	0	0	12	34	50	0	31	0	0	0	0	0	0	25	0	0	0	0	0	0	0	0	0
CTC_RS14000	complement(59451..60158)	0	134	188	54	32	73	0	22	0	25	23	0	0	83	119	0	36	45	24	68	101	0	105	0	0	134	131	0	35	81
CTC_RS14005	complement(60161..61354)	0	0	0	13	38	72	8	0	22	40	0	0	0	12	0	6	0	11	7	0	20	0	0	0	0	0	0	6	0	32
CTC_RS14010	complement(61356..62486)	0	0	0	7	41	30	0	0	0	3	14	0	0	0	0	12	22	56	15	0	21	11	0	0	0	0	0	0	22	34
CTC_RS15050	complement(62491..62748)	0	0	0	0	0	67	0	0	0	0	0	46	0	0	81	26	0	0	0	0	0	0	0	0	0	0	90	0	0	37
CTC_RS14020	complement(62882..63565)	0	225	0	22	235	38	0	46	0	17	23	0	0	0	31	119	332	167	0	0	0	0	55	0	17	49	0	20	0	14
CTC_RS14025	63985..65337	0	88	260	17	186	134	21	209	375	0	0	0	0	97	171	0	9	0	13	143	106	0	28	45	0	29	188	0	27	0
CTC_RS14030	complement(65527..65829)	0	0	0	0	0	0	0	0	0	0	0	0	0	0	0	0	0	0	0	0	0	0	0	0	0	0	0	0	0	0
CTC_RS14035	complement(66192..66410)	0	1029	61	0	628	0	0	72	0	0	0	0	0	0	0	0	4377	463	0	221	0	0	511	0	0	102	0	0	0	0
CTC_RS14040	complement(66434..66655)	0	6731	300	137	9499	233	126	2127	722	0	0	0	0	1022	94	245	5000	855	19	0	0	0	7397	367	0	1410	104	0	1106	173
CTC_RS14045	complement(66743..66922)	0	395	0	148	1019	0	0	0	0	0	45	0	0	0	0	0	0	0	24	0	0	0	1244	0	0	0	0	0	0	0
CTC_RS14050	67415..68161	362	2096	2848	960	2716	2919	674	2350	3486	530	2776	1406	594	1832	2569	538	1503	1661	1107	3626	5316	1196	3073	2125	446	1347	3693	405	1084	2438
CTC_RS14055	68165..68461	182	639	1321	334	1235	2728	126	874	1529	507	541	482	182	616	777	149	212	639	100	1792	3020	209	1068	1371	27	263	781	23	248	1226
tetX	complement(68640..72587)	135	11121	9975	1370	20353	25530	142	8325	6901	18	751	399	168	3170	3897	36	179	632	43	1280	1460	632	17645	21665	98	3376	4487	136	2499	4967
botR	complement(72818..73354)	0	177	446	0	1195	1573	0	29	473	0	0	0	0	82	195	0	0	24	0	0	0	0	1390	1289	0	62	130	0	0	286
