# Supplementary material for: Comparative pathogenomic analysis reveals a highly tetanus toxin-producing clade of Clostridium tetani isolates in Japan
Source: mSphere. 2023 Nov 27;8(6):e00369-23. doi: 10.1128/msphere.00369-23 (PMC10732020; doi:10.1128/msphere.00369-23)
Supplement: Table S3 — Pan-genome regions and coding sequence list in the high toxin-producing strain KHSU-254310-026. [file msphere.00369-23-s0003.pdf]

Table S3 Pangenome regions and coding sequence list in the high toxin-producing strain KHSU-254310-026

Region	Locus tag	Product	TetX high producing isolates (n=21)	Others (n=200)
1	K254310026_09040	hypothetical protein	100%	21.00%
1	K254310026_09050	hypothetical protein	95.20%	12.50%
1	K254310026_09060	putative exonuclease	95.20%	12.50%
1	K254310026_09070	putative recombinase	95.20%	22.00%
1	K254310026_09080	hypothetical protein	95.20%	20.50%
1	K254310026_09090	transcriptional regulator	95.20%	23.00%
1	K254310026_09100	hypothetical protein	90.50%	11.50%
1	K254310026_09110	hypothetical protein	100%	2.00%
1	K254310026_09120	hypothetical protein	100%	0.50%
1	K254310026_09130	hypothetical protein	100%	0.50%
1	K254310026_09140	hypothetical protein	100%	0.50%
1	K254310026_09150	methyltransferase	100%	4.50%
1	K254310026_09160	hypothetical protein	100%	5.00%
1	K254310026_09170	hypothetical protein	95.20%	0.50%
1	K254310026_09180	hypothetical protein	100%	2.50%
1	K254310026_09250	hypothetical protein	100%	22.00%
1	K254310026_09260	putative membrane protein	100%	22.00%
1	K254310026_09270	putative membrane protein	100%	20.00%
1	K254310026_09280	hypothetical protein	95.20%	22.50%
1	K254310026_09290	hypothetical protein	100%	13.00%
1	K254310026_09300	hypothetical protein	100%	8.50%
1	K254310026_09640	hypothetical protein	100%	4.50%
1	K254310026_09660	mannosyl-glycoprotein endo-beta-N-acetylglucosaminidase	100%	3.50%
1	K254310026_09670	putative membrane protein	100%	7.00%
1	K254310026_09680	hypothetical protein	100%	2.00%
1	K254310026_09690	Acyl-CoA N-acyltransferase	100%	2.00%
1	K254310026_09700	hypothetical protein	100%	2.00%
1	K254310026_09710	P-loop containing nucleoside triphosphate hydrolase	100%	2.00%
2	K254310026_12910	cytosine permease CodB	100%	16.00%
2	K254310026_12920	cytosine deaminase CodA	100%	16.00%
3	K254310026_21670	DEAD/DEAH box helicase HsdR	100%	14.00%
3	K254310026_21680	type I restriction endonuclease MjaXP subunit S	100%	14.00%
3	K254310026_21690	restriction endonuclease subunit M	100%	14.00%
4	K254310026_24820	putative tRNA nuclease	71.40%	11.00%
4	K254310026_24830	putative nucleotidyl transferase	71.40%	11.00%
4	K254310026_24840	DNA repair ATPase	71.40%	11.00%
4	K254310026_24850	hypothetical protein	71.40%	11.00%
4	K254310026_24860	hypothetical protein	71.40%	11.00%
4	K254310026_24870	hypothetical protein	71.40%	11.00%
4	K254310026_24880	type I restriction endonuclease subunit R	71.40%	11.00%
4	K254310026_24890	hypothetical protein	71.40%	11.00%
4	K254310026_24900	hypothetical protein	71.40%	11.00%
4	K254310026_24910	hypothetical protein	71.40%	11.00%
4	K254310026_24920	putative restriction endonuclease	71.40%	11.00%
4	K254310026_24930	restriction modification system related protein	71.40%	11.00%
4	K254310026_24940	putative S-adenosyl-L-methionine-dependent methyltransferase	71.40%	11.00%
